# Catalytic enantioselective arylative cyclizations of alkynyl 1,3-diketones by 1,4-rhodium(i) migration†

**DOI:** 10.1039/c9sc06309a

**Published:** 2020-02-06

**Authors:** Alistair Groves, Jinwei Sun, Hal R. I. Parke, Michael Callingham, Stephen P. Argent, Laurence J. Taylor, Hon Wai Lam

**Affiliations:** The GlaxoSmithKline Carbon Neutral Laboratories for Sustainable Chemistry, University of Nottingham Jubilee Campus, Triumph Road Nottingham NG7 2TU UK hon.lam@nottingham.ac.uk; School of Chemistry, University of Nottingham University Park Nottingham NG7 2RD UK; Jiangsu Collaborative Innovation Center of Atmospheric Environment and Equipment Technology, Jiangsu Key Laboratory of Atmospheric Environment Monitoring and Pollution Control, School of Chemistry and Materials Science, Nanjing University of Information Science and Technology Nanjing Jiangsu 210044 China

## Abstract

The enantioselective synthesis of densely functionalized polycarbocycles by the rhodium(i)-catalyzed reaction of arylboronic acids with 1,3-diketones is described. The key step in these desymmetrizing domino addition–cyclization reactions is an alkenyl-to-aryl 1,4-Rh(i) migration, which enables arylboronic acids to function effectively as 1,2-dimetalloarene surrogates.

## Abstract 2

The enantioselective synthesis of densely functionalized polycarbocycles by the rhodium(i)-catalyzed reaction of arylboronic acids with alkynyl 1,3-diketones is described. The key step in these reactions is an alkenyl-to-aryl 1,4-Rh(i) migration..

## Graphical abstract

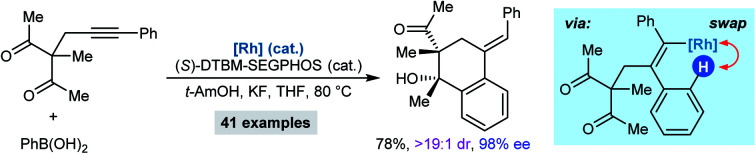

The functionalization of remote C–H bonds offers a powerful method to develop new synthetic methods and achieve transformations that would otherwise be highly challenging.^1^ Within this field, 1,4-migration of rhodium(i) between two carbon centers^2–4^ has proven to be highly effective for the catalytic functionalization of remote C–H bonds, which has been used to impressive effect in a range of valuable synthetic methods.^4^

We have described catalytic arylative cyclizations from the reaction of alkynyl ketones with arylboronic acids, which produce densely functionalized polycarbocycles through a key step involving an alkenyl-to-aryl 1,4-metal migration (Scheme 1A).^5^ This desymmetrization reaction forms two new carbon–carbon bonds with complete diastereocontrol over two new stereocenters and a trisubstituted alkene. In the non-enantioselective variant of this process, rhodium(i) catalysis was only moderately successful because of the formation of significant quantities of side-products, and the highest yields were obtained using iridium(i) catalysis.^5,6^ Although preliminary attempts towards an enantioselective variant using chiral bisphosphine–iridium complexes successfully gave products in high enantioselectivities, only modest catalytic activities were observed.^5^ Furthermore, only cyclic ketones were employed in that study.^5^ Yan and Yoshikai have reported related cobalt-catalyzed arylative cyclizations of acyclic 1,3-diketones with diarylzinc reagents; however, enantioselective reactions were not described (Scheme 1B).^7^ Therefore, to increase synthetic utility, there remains a need to discover more effective chiral catalysts that address these limitations by promoting high-yielding and highly enantioselective arylative cyclizations of a wider range substrates,^8^ including acyclic 1,3-diketones. Here, we report that a chiral bisphosphine–rhodium complex promotes the diastereo- and enantioselective reaction of arylboronic acids with alkynyl 1,3-diketones, for which both acyclic and cyclic 1,3-diketones are effective substrates.

**Scheme 1:**
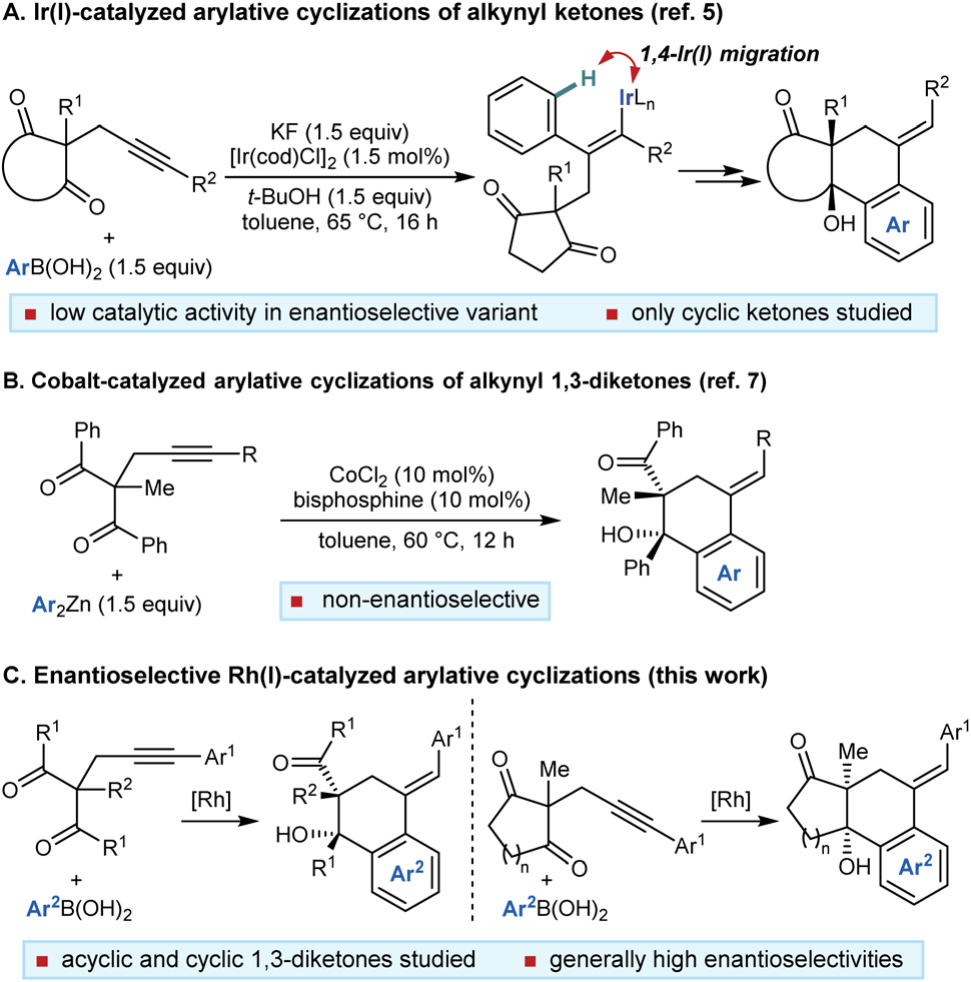
Catalytic arylative cyclizations involving 1,4-metal migration.

Our experiments began with the arylative cyclization of alkynyl 1,3-diketone **1a** with PhB(OH)_2_ (eqn (1) and Table 1). Application of conditions identical to those described in our1
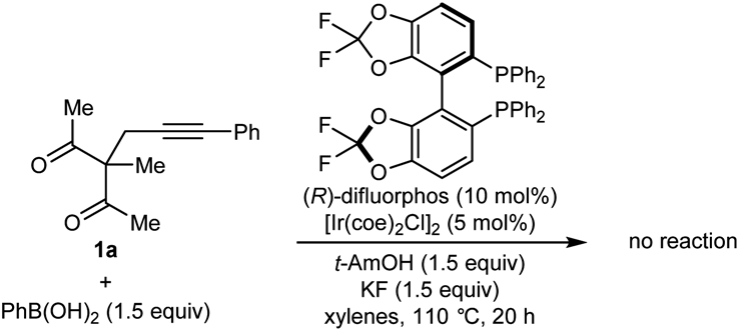
previous study,^5^ using an iridium–(*R*)-difluorphos complex, led to no reaction and only unreacted starting material was recovered (eqn (1)). Pleasingly, however, reaction of **1a** with PhB(OH)_2_ (1.5 equiv.) in the presence of [Rh(C_2_H_4_)_2_Cl]_2_ (5 mol%), (*R*)-BINAP (**L1**, 10 mol%), and KF (1.5 equiv.) in THF/H_2_O (9 : 1) at 70 °C for 24 h gave arylative cyclization product *ent*-**2a** in 54% yield (as determined by ^1^H NMR analysis using 1,4-dimethoxybenzene as an internal standard) as a single diastereomer (>19 : 1 dr) in 80% ee (Table 1, entry 1).^9^ Higher enantioselectivity was obtained using (*S*)-DTBM-SEGPHOS (**L2**), which gave **2a** in 52% NMR yield and 91% ee (entry 2). Changing the protic additive from H_2_O to *t*-AmOH (1.5 equiv.) further increased the enantioselectivity (entry 3). The yield of **2a** was increased further by raising the temperature to 80 °C (entry 4) and using 2.0 equivalents of PhB(OH)_2_ (entry 5). Conducting the reaction on a larger scale using 0.30 mmol of **1a** gave **2a** in 78% yield and 98% ee (entry 6). This experiment also gave a side-product **3a** in 5% yield.^10^ It should be noted that the use of PhB(OH)_2_ free from triphenylboroxine is very important for good results, as otherwise lower enantioselectivities are observed.^11^ Finally, repeating the conditions of entry 5 but using [Ir(coe)_2_Cl]_2_ in place of [Rh(C_2_H_4_)Cl_2_] led to no reaction, and only unreacted starting material was recovered (entry 7).

**Table 1:** Evaluation of reaction conditions^a^

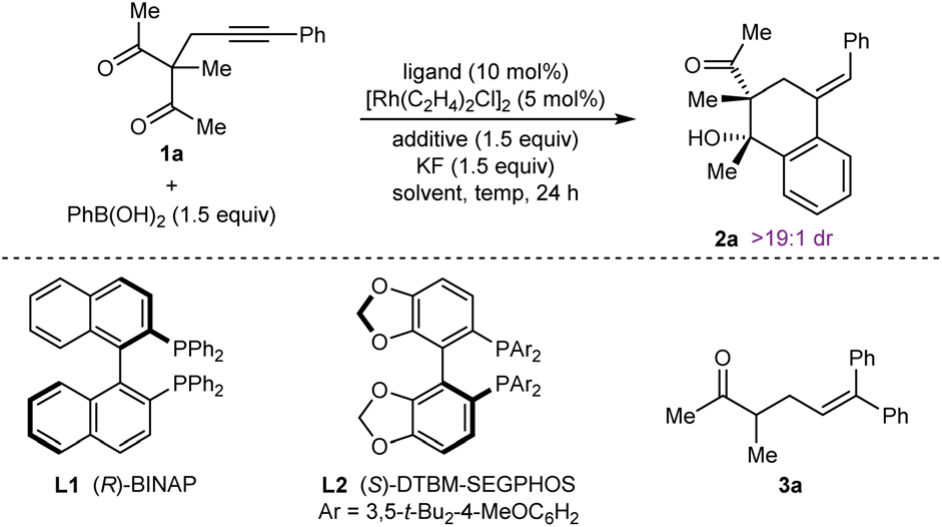						
Entry	Ligand	Additive	Solvent	Temp. (°C)	Yield^b^ (%)	ee^c^ (%)
1	**L1**	—	THF : H_2_O (9 : 1)	70	54	−80^d^
2	**L2**	—	THF : H_2_O (9 : 1)	70	52	91
3	**L2**	*t*-AmOH	THF	70	54	95
4	**L2**	*t*-AmOH	THF	80	67	96
5^e^	**L2**	*t*-AmOH	THF	80	70	96
6^e^^,^^f^	**L2**	*t*-AmOH	THF	80	78 (5)^g^	98
7^e^^,^^h^	**L2**	*t*-AmOH	THF	80	n.r.^i^	—

a Reactions were conducted with 0.05 mmol of **1a** in 1 mL of solvent.

b Determined by ^1^H NMR analysis using 1,4-dimethoxybenzene as an internal standard.

c Determined by HPLC analysis on a chiral stationary phase.

d The major enantiomer was *ent*-**2a**.

e Using 2.0 equivalents of PhB(OH)_2_.

f Using 0.30 mmol of **1a** in THF (6 mL).

g Value in parentheses refers to the yield of side-product **3a**, which was also isolated from this experiment.

h Using [Ir(coe)Cl_2_]_2_ in place of [Rh(C_2_H_4_)_2_Cl_2_].

i n.r. = no reaction.

With effective conditions identified (Table 1, entry 6), the scope of this process with respect to the alkynyl acyclic 1,3-diketone **1** was investigated in reactions with PhB(OH)_2_ (Table 2). Arylative cyclization products **2a–2r** were obtained as single observable diastereomers (>19 : 1 dr as determined by ^1^H NMR analysis of the crude reaction mixtures) in 27–82% yield and 56–99% ee. Side-products analogous to **3a** (see Table 1) were generally detected but not isolated. Changing the α-substituent R^2^ between the two ketones from methyl (**2a**) to ethyl (**2b**), *n*-butyl (**2c**), benzyl (**2d**), or 4-methoxybenzyl (**2e**) is tolerated. The low yield of **2c** results from a low conversion as significant unreacted starting material was observed. The process is also compatible with a range (hetero)aryl groups Ar^1^ at the alkynyl position, such as 4-substituted phenyl (**2f–2i**), 2-fluorophenyl (**2j**), 3,5-dimethylphenyl (**2k**), 3,4-(methylenedioxy)phenyl (**2l**), 2-naphthyl (**2m**), 1-naphthyl (**2n**), 2-thienyl (**2o**), and 2-pyridyl (**2p**) groups. Finally, the ketone substituents can also be varied from methyl to ethyl (**2q**) or phenyl groups (**2r**), although the enantioselectivity dropped substantially in the latter case.

**Table 2:** Evaluation of alkynyl acyclic 1,3-diketones **1**^a^

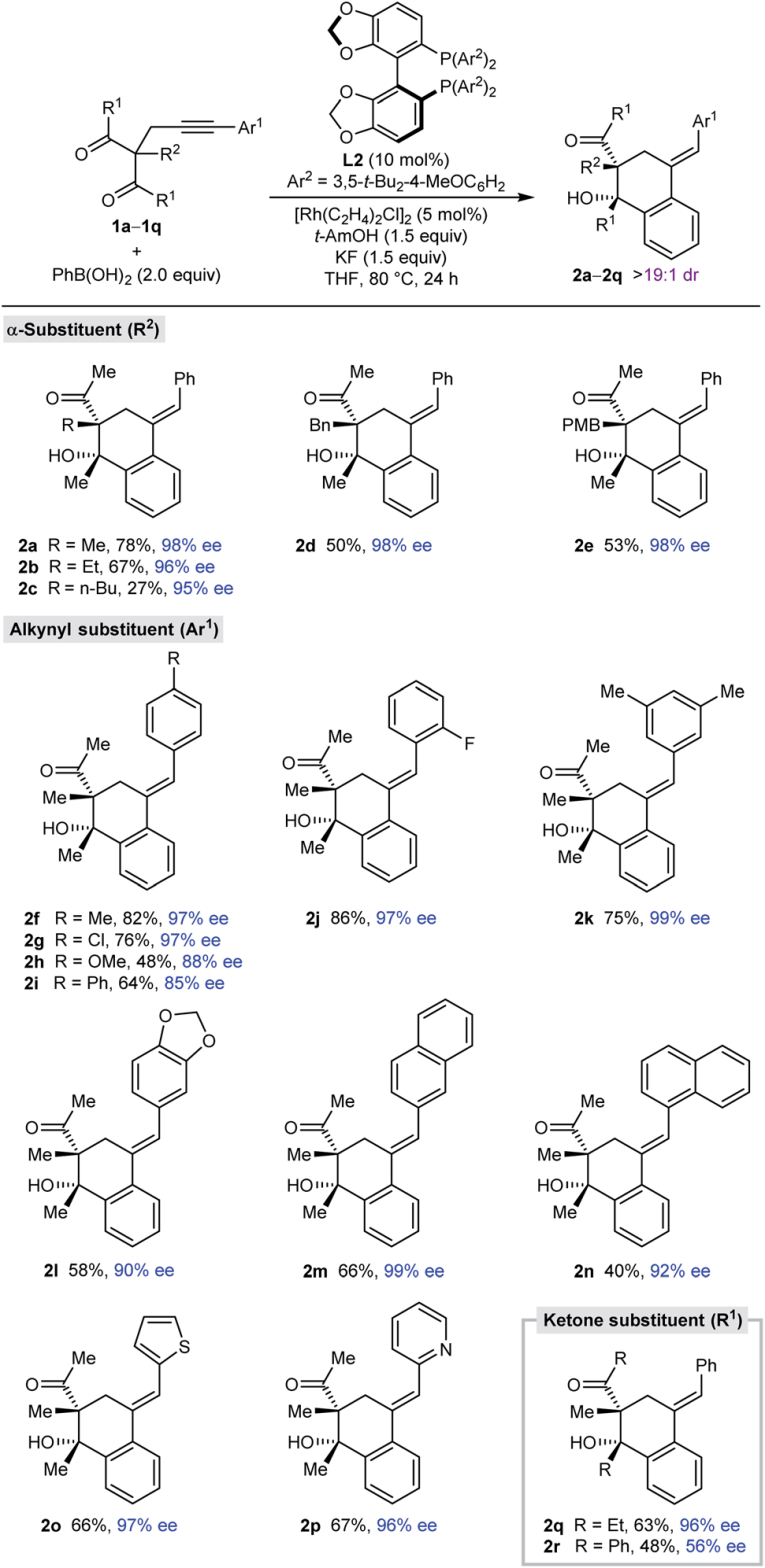

a Reactions were conducted with 0.30 mmol of **1** in THF (6 mL). Yields are of isolated products. Enantiomeric excesses were determined by HPLC analysis on a chiral stationary phase. PMB = *para*-methoxybenzyl.

The process is not limited to the use of PhB(OH)_2_, as shown by the arylative cyclizations of **1a** with different arylboronic acids to give **2s–2x** in 88–96% ee (Table 3). Various 4-substituted phenylboronic acids containing methyl (**2s**), methoxy (**2t**), fluoro (**2u**), or chloro groups (**2v**) reacted successfully. When 3-methylphenylboronic acid was employed, 1,4-Rh(i) migration occurred to the least sterically hindered site, *para* to the methyl group (**2x**). However, 2-methylphenylboronic acid did not provide any of the arylative cyclization product **2y**, and returned mainly unreacted starting material along with what appeared to be small quantities of alkyne hydroarylation products.

**Table 3:** Evaluation of arylboronic acids with alkynyl acyclic 1,3-diketone **1a**^a^

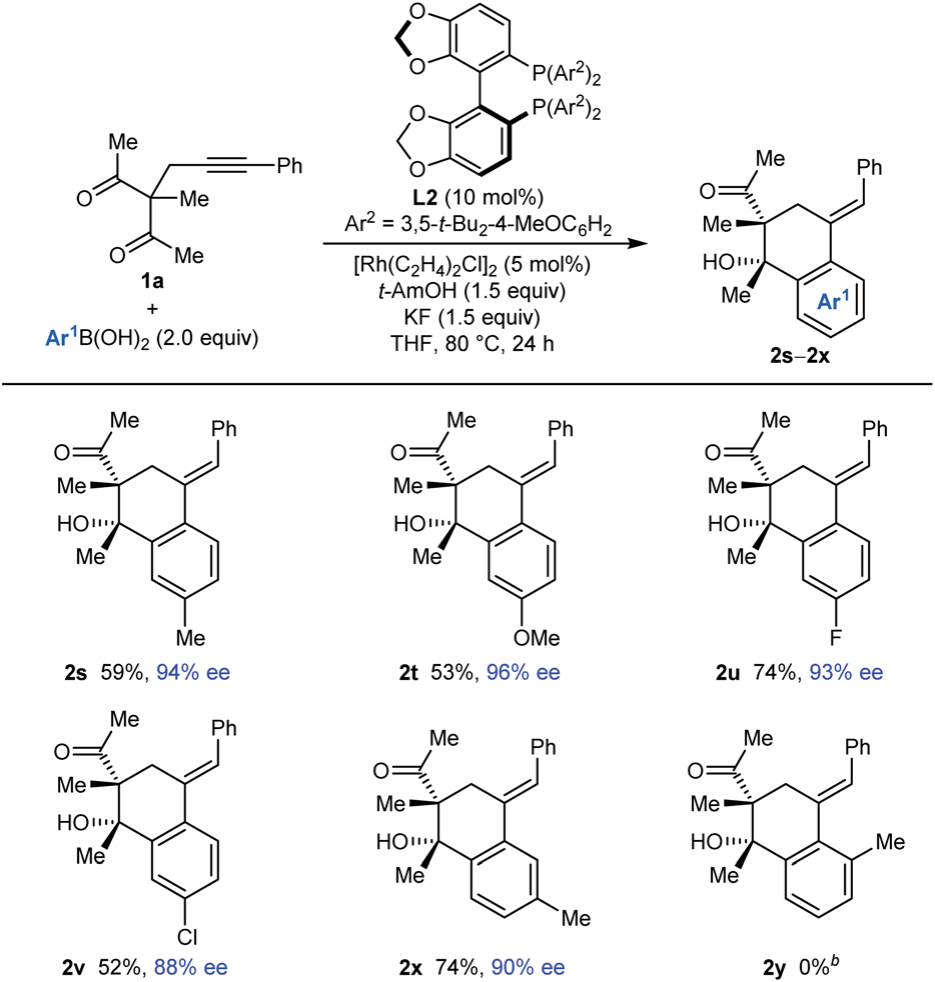

a Reactions were conducted with 0.30 mmol of **1a** in THF (3 mL). Yields are of isolated products. Enantiomeric excesses were determined by HPLC analysis on a chiral stationary phase.

b Unreacted starting material was returned along with a trace of what appeared to be alkyne hydroarylation products.

Our attention then turned to the reaction of alkynyl cyclic 1,3-diketones **4**, substrates employed in our prior study using iridium catalysis (Tables 4 and 5).^5^ With toluene as the solvent, these more reactive substrates generally allowed the use of a decreased catalyst loading of 5 mol% and a lower temperature of 50 °C. Furthermore, in most cases, acceptable results were obtained using only 1.5 equivalents of the arylboronic acid. Various substrates **4a–4f** underwent arylative cyclization with PhB(OH)_2_ to give products **5a–5f** in 39–74% yield and 61–99% ee (Table 4). Small quantities of side-products resulting from arylrhodation of the alkyne with the regioselectivity opposite to that seen in the formation of products **5** were also observed but generally not isolated (see ESI† for details). As with the acylic 1,3-diketones (Table 2), a range of aryl substituents at the alkyne are tolerated, including phenyl (**5a** and **5f**), 4-methoxyphenyl (**5b**), 4-chlorophenyl (**5c**), and 3-methylphenyl (**5d**). The lower yield of **5b** results from the formation of products of alkyne hydroarylation without cyclization. The cyclization of a 2-cyanophenyl-containing substrate **4e** proceeded smoothly using a 10 mol% catalyst loading but the product **5e** was formed in a modest 61% ee. A six-membered cyclic 1,3-diketone also underwent arylative cyclization with PhB(OH)_2_ to give **5f** in 60% and 97% ee.

**Table 4:** Evaluation of alkynyl cyclic 1,3-diketones **4**^a^

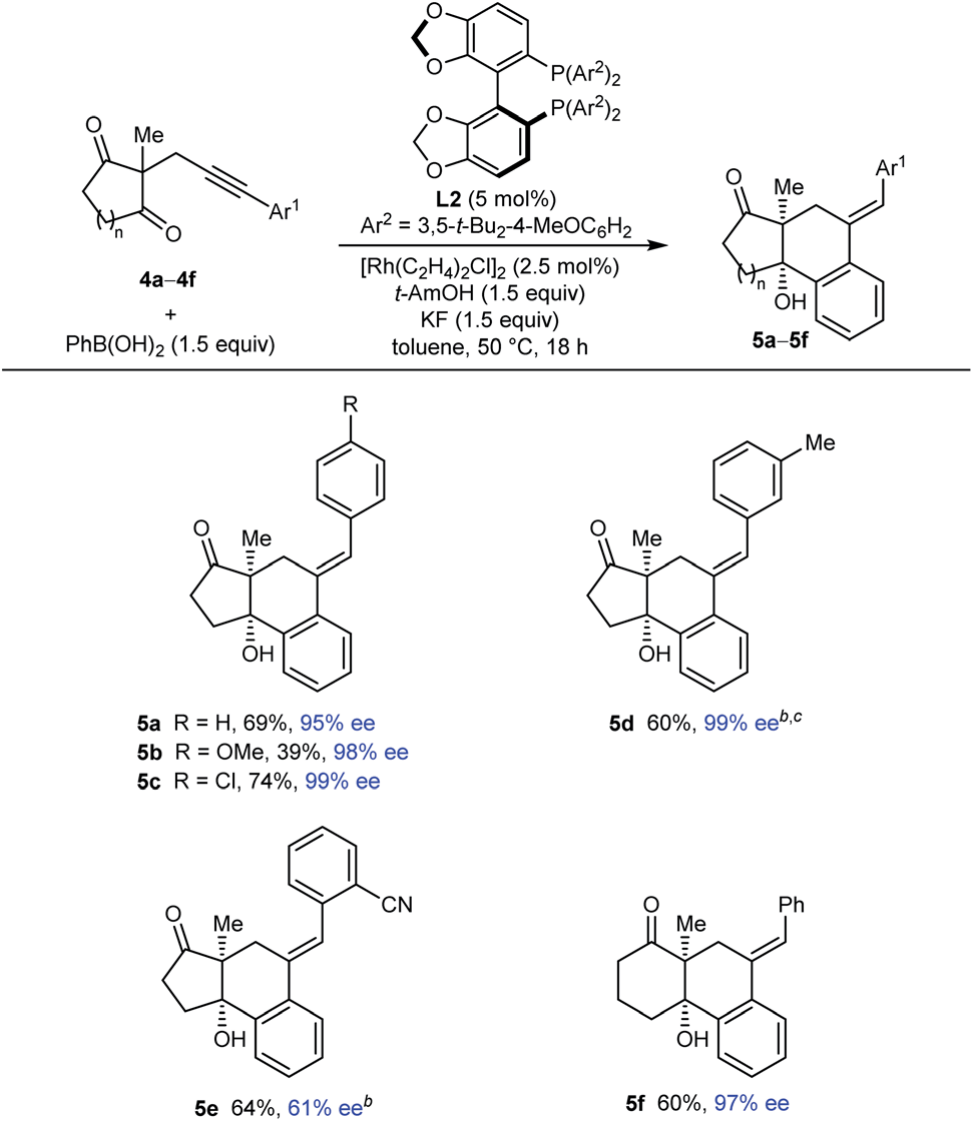

a Reactions were conducted with 0.30 mmol of **4** in toluene (3 mL). Yields are of isolated products. Enantiomeric excesses were determined by HPLC analysis on a chiral stationary phase.

b Using 5 mol% of [Rh(C_2_H_4_)_2_Cl]_2_, 10 mol% of **L2**.

c Using 2.0 equiv. of PhB(OH)_2_.

**Table 5:** Evaluation of arylboronic acids with alkynyl cyclic 1,3-diketone **4a**^a^

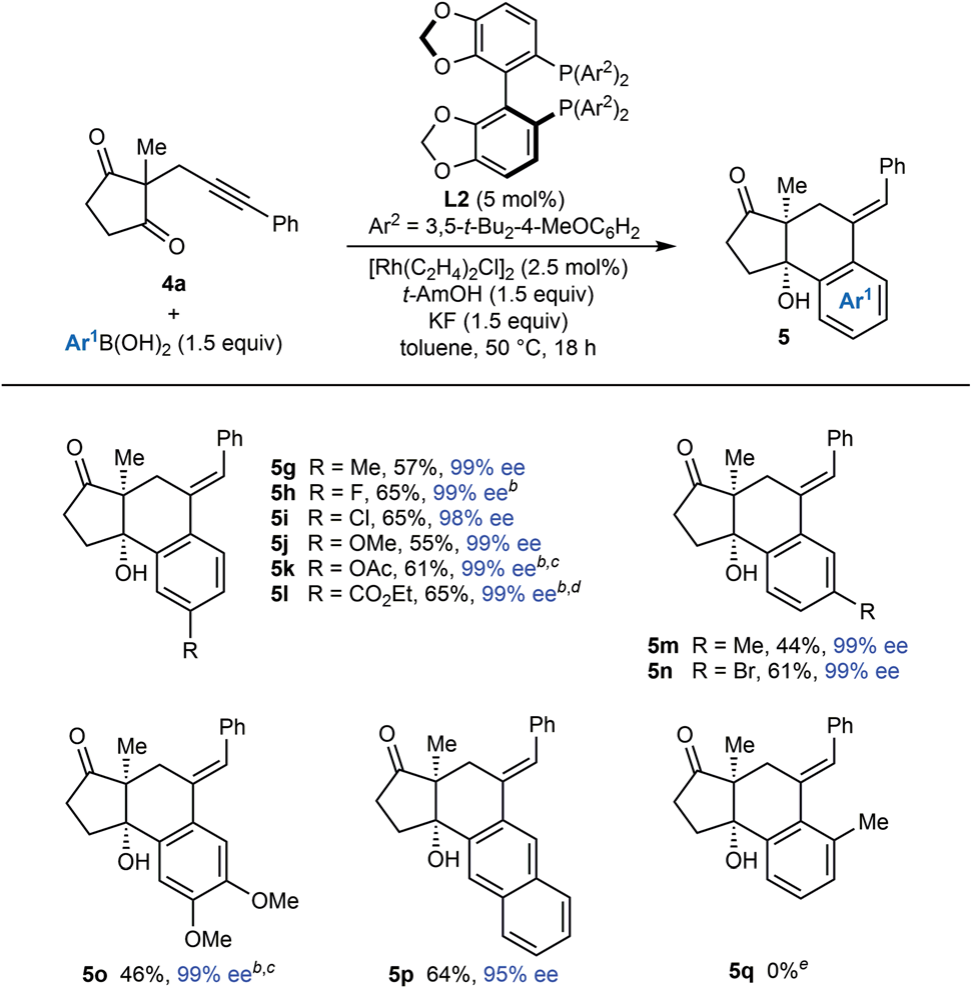

a Reactions were conducted with 0.30 mmol of **4** in toluene (3 mL). Yields are of isolated products. Enantiomeric excesses were determined by HPLC analysis on a chiral stationary phase.

b Using 5 mol% of [Rh(C_2_H_4_)_2_Cl]_2_ and 10 mol% of **L2**.

c Using 2.0 equivalents of the arylboronic acid.

d Using 2.4 equivalents of the arylboronic acid.

e Unreacted starting material was returned.

The scope of the arylative cyclization of alkynyl cyclic 1,3-diketones with respect to the arylboronic acid was then explored in reactions with substrate **4a** (Table 5). These reactions proceeded in 44–69% yield and gave products **5g–5p** in 95–99% ee. The process tolerates diverse 4-substituted phenylboronic acids containing methyl (**5g**), halide (**5h** and **5i**), methoxy (**5j**), acetoxy (**5k**), or carboethoxy groups (**5l**). 3-Substituted phenylboronic acids (**5m** and **5n**), 3,4-dimethoxyphenylboronic acid (**5o**), and 2-naphthylboronic acid (**5p**) also react effectively. Again, where 1,4-Rh(i) migration could occur to two different positions, migration to the sterically less-hindered side was observed (**5m–5p**). An attempt to form **5q** with 2-methylphenylboronic acid was unsuccessful, and returned only unreacted starting material.

Interestingly, the reaction of substrate **4a** with 3-thienylboronic acid gave two products **5ra** and **5rb** resulting from 1,4-Rh(i) migration to different positions of the thienyl ring before cyclization (eqn (2)).2
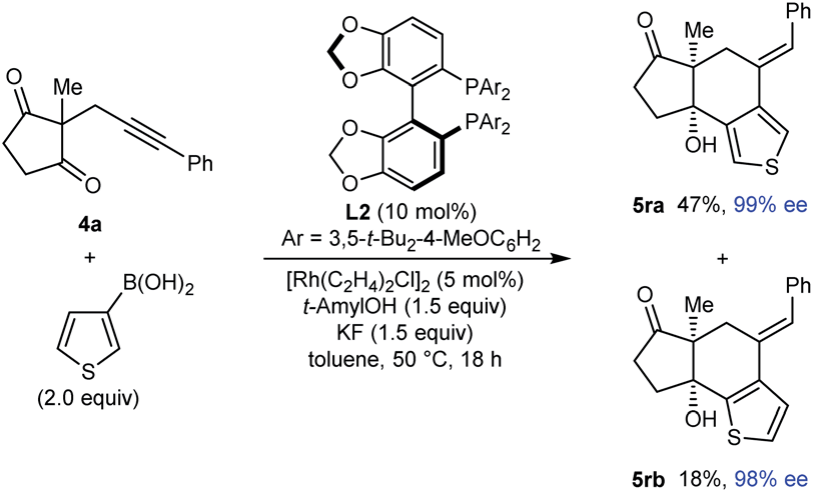


Finally, this method is not restricted to the use of 1,3-diketone-containing substrates; substrate **6** containing a single methyl ketone also underwent arylative cyclization to give **7** in 94% yield and 85% ee (eqn (3)).3
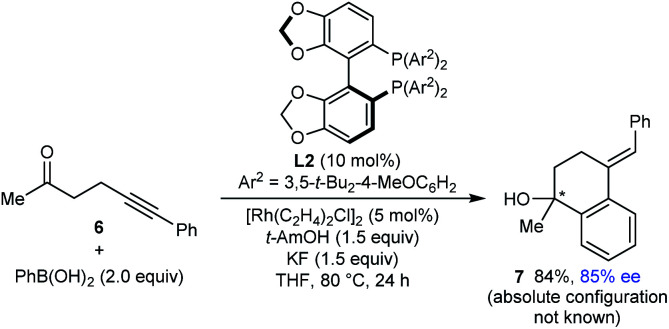


Scheme 2 illustrates a possible catalytic cycle for these reactions, using **1a** and PhB(OH)_2_ as example substrates. First, upon mixing [Rh(C_2_H_4_)Cl]_2_, **L2**, KF, and *t*-AmOH, a chiral complex **8** consisting of one bisphosphine bound to one rhodium atom is formed, which could have a chloride, fluoride, or *tert*-amyl counterion. Transmetalation of **8** with PhB(OH)_2_ gives an arylrhodium species **9**, which can then undergo migratory insertion with the alkyne of **1a** to give alkenylrhodium intermediate **10**. Alkenyl-to-aryl 1,4-rhodium(i) migration of **10** then provides arylrhodium species **11**. The relative configuration of products **2** can be explained by a tentative stereochemical model where cyclization proceeds through a conformation similar to **12**, in which: (i) rhodium(i) has a square pyramidal coordination geometry; (ii) the ketone undergoing nucleophilic attack is coordinated to rhodium such that the carbonyl group is aligned with the arylrhodium bond to enable subsequent migratory insertion; and (iii) the second ketone is coordinated to rhodium in an axial position. The relative configuration of products **5** (Tables 4 and 5) is more straightforward to rationalize; because of geometric constraints, nucleophilic addition of the arylrhodium group must occur to the same face of the cyclic 1,3-diketone as that from which the tether connecting the two reacting components projects (as in **14** to give representative product **5a**, for example). However, as to exactly how the chiral ligand controls the absolute configuration of the products is not clear at the present time.

**Scheme 2:**
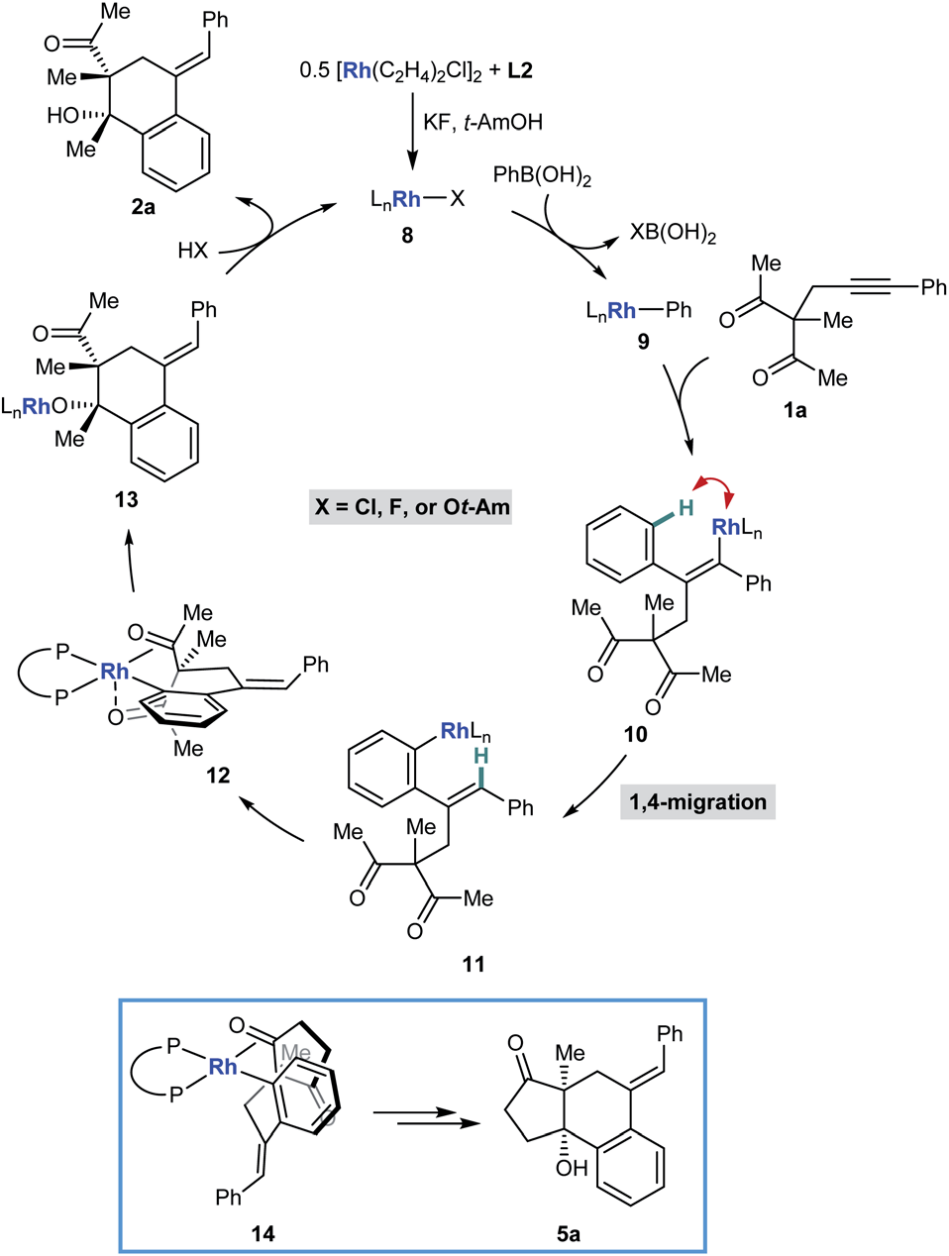
Possible catalytic cycle and rationalization of diastereochemical outcomes.

In conclusion, we have reported rhodium(i)-catalyzed arylative cyclizations of alkynyl 1,3-diketones with arylboronic acids, which involve an alkenyl-to-aryl 1,4-Rh(i) migration as a key step. By using a chiral rhodium(i) complex based upon (*S*)-DTBM-SEGPHOS, the formation of side-products observed previously^5^ with [Rh(cod)Cl]_2_ is significantly reduced, and catalytic activity is greatly increased compared with chiral iridium complexes.^5^ These desymmetrization reactions provide densely functionalized polycarbocycles with high diastereo- and enantioselectivities, and notably, both acyclic and cyclic 1,3-ketones are effective substrates.^12^

## Conflicts of interest

There are no conflicts to declare.

## Supplementary Material

SC-011-C9SC06309A-s001

SC-011-C9SC06309A-s002
