# Supplementary material for: Catalytic enantioselective arylative cyclizations of alkynyl 1,3-diketones by 1,4-rhodium(i) migration
Source: Chem Sci. 2020 Feb 6;11(10):2759–64. doi: 10.1039/c9sc06309a (PMC8157494; doi:10.1039/c9sc06309a)
Supplement: SC-011-C9SC06309A-s001 [file SC-011-C9SC06309A-s001.pdf]

Electronic Supplementary Material (ESI) for Chemical Science.
This journal is © The Royal Society of Chemistry 2020

Catalytic enantioselective arylative cyclizations of alkynyl 1,3-diketones by 1,4-rhodium(I) migration

Alistair Groves,[a,b] Jinwei Sun,[a,b,c] Hal R. I. Parke,[a,b] Michael Callingham,[a,b] Stephen P. Argent,[b] Laurence J. Taylor,[b] and Hon Wai Lam*,[a,b]

[a] *The GlaxoSmithKline Carbon Neutral Laboratories for Sustainable Chemistry, University of Nottingham, Jubilee Campus, Triumph Road, Nottingham, NG7 2TU, United Kingdom*

and

[b] *School of Chemistry, University of Nottingham, University Park, Nottingham, NG7 2RD, United Kingdom*

and

[c] *Jiangsu Collaborative Innovation Center of Atmospheric Environment and Equipment Technology, Jiangsu Key Laboratory of Atmospheric Environment Monitoring and Pollution Control, School of Chemistry and Materials Science, Nanjing University of Information Science and Technology, Nanjing, Jiangsu 210044 (China)*

Supplementary Information

General Information 2

Preparation of Substrates 3

Enantioselective Arylative Cyclizations of Alkynyl 1,3-Diketones 15

NMR Spectra of New Compounds 40

HPLC Traces 89

References 131

General Information

All air–sensitive reactions were carried out under an inert atmosphere using oven–dried apparatus. THF and toluene were dried and purified by passage through activated alumina columns using a solvent purification system. All commercially available reagents were used as received unless otherwise stated. Arylboronic acids were used as received unless the sample contained >10% boroxine as determined by ^{1}H NMR analysis. In this case, the boronic acid was stirred in a mixture of Et_2O and water for 30 min. The organic phase was separated, dried (Na_2SO_4), filtered, and concentrated *in vacuo* to give the corresponding boronic acid which was used without further purification. Petroleum ether refers to Sigma-Aldrich product 24587 (petroleum ether boiling point 40–60 °C). Thin layer chromatography (TLC) was performed on Merck DF Alufoilien 60F254 0.2 mm precoated plates. Compounds were visualized by exposure to UV light or by dipping the plates into solutions of potassium permanganate or vanillin followed by gentle heating. FlAS-H column chromatography was carried out using silica gel (Fisher Scientific 60 Å particle size 35–70 micron or Fluorochem 60 Å particle size 40–63 micron). Automated column chromatography was conducted using PuriFlash instrument from Interchim. Melting points were recorded on a Gallenkamp melting point apparatus and are uncorrected. The solvent of recrystallization is reported in parentheses. Infrared (IR) spectra were recorded on a Bruker platinum alpha FTIR spectrometer on the neat compound using the attenuated total refraction technique. NMR spectra were acquired on Bruker Ascend 400 or Ascend 500 spectrometers. ^{1}H and ^{13}C NMR spectra were referenced to external tetramethylsilane *via* the residual protonated solvent (^{1}H) or the solvent itself (^{13}C). ^{19}F NMR spectra were referenced through the solvent lock (^{2}H) signal according to the IUPAC-recommended secondary referencing method following Bruker protocols. All chemical shifts are reported in parts per million (ppm). For $CDCl_3$, the shifts are referenced to 7.27 ppm for ^{1}H NMR spectroscopy and 77.0 ppm for ^{13}C NMR spectroscopy. Abbreviations used in the description of resonances are: s (singlet), d (doublet), t (triplet), q (quartet), quint (quintet), br (broad) and m (multiplet). Coupling constants (*J*) are quoted to the nearest 0.1 Hz. ^{13}C NMR assignments were made using the DEPT sequence with secondary pulses at 90° and 135°. High–resolution mass spectra were recorded using electrospray ionization (ESI). X–ray diffraction data were collected at 120 K on an Agilent SuperNova diffractometer using CuKα radiation. Chiral HPLC analysis was performed on Agilent 1200 Infinity series instruments using 4.6 × 250 mm columns. $[Rh(cod)Cl]_2$ was used as an achiral rhodium complex to obtain authentic racemic compounds.

Preparation of Substrates

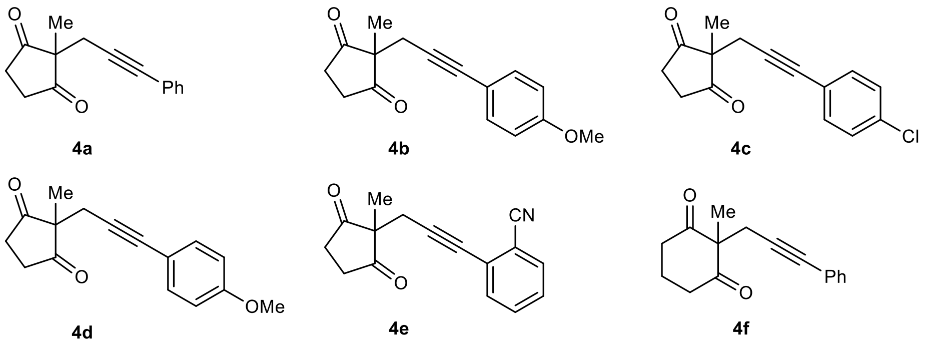

Substrates **4a**–**4f** were prepared as described previously.[1]

3-Methyl-3-(3-phenylprop-2-yn-1-yl)pentane-2,4-dione (1a)[2]

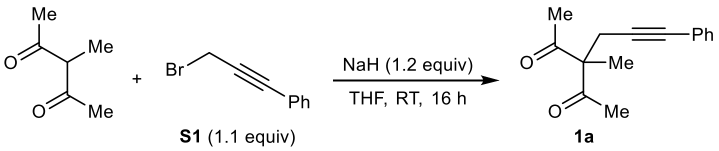

3-Methyl-2,4-pentanedione (1.75 mL, 15.0 mmol) was added dropwise to a solution of NaH (60% in mineral oil, 720 mg, 18.0 mmol) in THF (50 mL) at 0 °C under argon and the resulting mixture was stirred for 1 h. Alkynyl bromide **S1**[3] (80% wt. solution in toluene, 4.00 g, 16.5 mmol) was added dropwise and the solution was stirred at room temperature for 16 h, quenched with saturated aqueous NH_4Cl solution (50 mL), and extracted into Et_2O (3 × 50 mL). The combined organic layers were washed with brine (50 mL), dried ($MgSO_4$), filtered, and concentrated *in vacuo*. Purification of the residue by column chromatography (100% petroleum ether to 5% EtOAc/petroleum ether) gave alkynyl 1,3-diketone **1a** as an orange oil (1.64 g, 48%) whose spectroscopic data were consistent with those reported previously.[2]

3-Ethyl-3-(3-phenylprop-2-yn-1-yl)pentane-2,4-dione (1b)

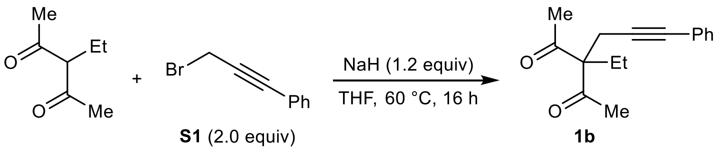

3-Ethylpentane-2,4-dione (4.03 mL, 3.00 mmol) was added dropwise to a solution of NaH (60% in mineral oil, 144 mg, 3.60 mmol) in THF (30 mL) at 0 °C under argon and the resulting mixture was stirred at room temperature for 1 h. Alkynyl bromide **S1**[3] (80% wt. solution in toluene, 1.46 g, 6.00 mmol) was added dropwise *via* syringe (using 2 mL of THF as a rinse) and the solution was stirred at 60 °C for 16 h. The reaction was cooled to room temperature, quenched with saturated aqueous NH_4Cl solution (50 mL), and extracted into Et_2O (3 × 50 mL). The combined organic layers were washed with brine (50 mL), dried ($MgSO_4$), filtered, and concentrated *in vacuo*. Purification of the

residue by column chromatography (100% petroleum ether to 10% EtOAc/petroleum ether) gave *alkynyl 1,3-diketone* **1b** as a yellow oil (580 mg, 82%). R_f = 0.35 (10% EtOAc/pentane); IR (ATR) 2971, 1696 (C=O), 1491, 1356, 1157, 1107, 937, 755, 691 cm^{-1}; ^{1}H NMR (400 MHz, $CDCl_3$) δ 7.36–7.32 (2H, m, Ar**H**), 7.30–7.26 (3H, m, Ar**H**), 2.98 (2H, s, ≡CC$\mathbf{H}_2$), 2.22–2.16 (2H, m, C$\mathbf{H}_2$$CH_3$), 2.18 (6H, s, 2 × C$\mathbf{H}_3$C=O), 0.82 (3H, t, *J* = 7.6 Hz, CH_2C$\mathbf{H}_3$); ^{13}C NMR (101 MHz, $CDCl_3$) δ 205.5 (2 × C), 131.6 (2 × CH), 128.2 (2 × CH), 128.1 (CH), 123.0 (C), 84.7 (C), 83.7 (C), 70.7 (C), 26.9 (2 × CH_3), 23.7 (CH_2), 20.1 (CH_2), 8.1 (CH_3); HRMS (ESI) Exact mass calculated for $[C_{16}H_{18}NaO_2]^+$ $[M+Na]^+$: 265.1199, found 265.1199.

3-Butyl-3-(3-phenylprop-2-yn-1-yl)pentane-2,4-dione (1c)

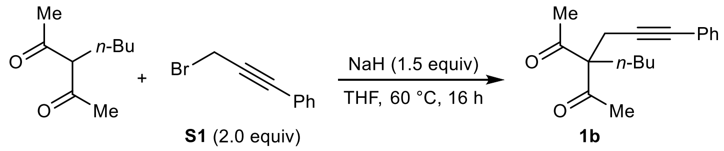

3-Butyl-2,4-pentanedione (506 µL, 3.00 mmol) was added dropwise to a solution of NaH (60% in mineral oil, 180 mg, 4.50 mmol) in THF (30 mL) at 0 °C under argon and the resulting mixture was stirred at room temperature for 1 h. Alkynyl bromide **S1**[3] (80% wt. solution in toluene, 1.46 g, 6.00 mmol) was added dropwise *via* syringe (using 2 mL of THF as a rinse) and the solution was stirred at 60 °C for 16 h. The reaction was cooled to room temperature, quenched with NH_4Cl (50 mL), and extracted into Et_2O (3 × 50 mL). The combined organic layers were washed with brine (50 mL), dried ($MgSO_4$), filtered, and concentrated *in vacuo*. Purification of residue by column chromatography (100% petroleum ether to 10% EtOAc/petroleum ether) gave *alkynyl 1,3-diketone* **1c** as a yellow oil (713 mg, 88%). R_f = 0.35 (10% EtOAc/pentane); IR (ATR) 2958, 2933, 2873, 1698 (C=O), 1356, 1185, 1158, 1116, 756, 691 cm^{-1}; ^{1}H NMR (500 MHz, $CDCl_3$) δ 7.37–7.33 (2H, m, Ar**H**), 7.30–7.26 (3H, m, Ar**H**), 2.99 (2H, s, ≡CC$\mathbf{H}_2$), 2.18 (6H, s, 2 × C$\mathbf{H}_3$C=O), 2.17–2.10 (2H, m, C$\mathbf{H}_2$$CH_2CH_2CH_3$), 1.39 (2H, sext, *J* = 7.4 Hz, CH_2C$\mathbf{H}_2$$CH_3$), 1.12–1.06 (2H, m, C$\mathbf{H}_2$$CH_2CH_3$), 0.93 (3H, t, *J* = 7.4 Hz, CH_2C$\mathbf{H}_3$); ^{13}C NMR (101 MHz, $CDCl_3$) δ 205.5 (2 × C), 131.6 (2 × CH), 128.2 (2 × CH), 128.0 (CH), 123.0 (C), 84.8 (C), 83.7 (C), 70.4 (C), 30.5 (CH_2), 26.9 (2 × CH_3), 25.9 (CH_2), 23.1 (CH_2), 21.6 (CH_2), 13.8 (CH_3); HRMS (ESI) Exact mass calculated for $C_{18}H_{22}NaO_2^+$ $[M+Na]^+$: 293.1512, found: 293.1504.

Synthesis of Substrate 1d

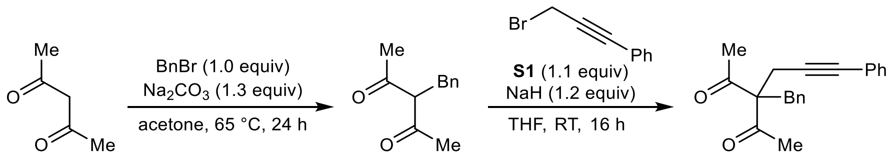

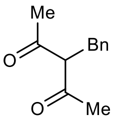

3-Benzylpentane-2,4-dione (S2).[4] Na_2CO_3 (6.86 g, 65.0 mmol) was added portionwise over 15 min to a solution of pentane-2,4-dione (5.10 mL, 50.0 mmol) in acetone (25 mL) at 0 °C and the solution was stirred for 15 min. A solution of benzyl bromide (5.95 mL, 50.0 mmol) in acetone (25 mL) was added dropwise over 15 min and the solution was heated at reflux for 24 h. The reaction was cooled to room temperature, filtered, and the filtrate was concentrated *in vacuo*. Purification of the residue by column chromatography (10% Et_2O/petroleum ether) gave diketone **S2** as a yellow oil (5.16 g, 54%, 24:76 ratio of keto:enol tautomers) whose spectroscopic data were consistent with those reported previously.[4]

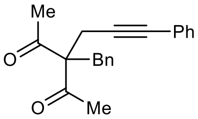

3-Benzyl-3-(3-phenylprop-2-yn-1-yl)pentane-2,4-dione (1d). 3-Benzylpentane-2,4-dione (**S2**, 1.90 g, 10.0 mmol) was added dropwise *via* syringe (using 2 mL of THF as a rinse) to a solution of NaH (60% in mineral oil, 480 mg, 12.0 mmol) in THF (30 mL) at 0 °C under argon and the resulting mixture was stirred at room temperature for 1 h. Alkynyl bromide **S1**[3] (80% wt. solution in toluene, 2.67 g, 11.0 mmol) *via* syringe (using 2 mL of THF as a rinse) was added dropwise and the solution was stirred at room temperature for 16 h, quenched with saturated aqueous NH_4Cl solution (50 mL), and extracted into Et_2O (3 × 50 mL). The combined organic layers were washed with brine (50 mL), dried ($MgSO_4$), filtered, and concentrated *in vacuo*. Purification of the residue by column chromatography (100% petroleum ether to 10% EtOAc/petroleum ether) gave *alkynyl 1,3-diketone* **1d** as a yellow oil (1.16 g, 38%). R_f = 0.19 (10% EtOAc/pentane); IR (ATR) 3069, 3029, 2956, 1695 (C=O), 1489, 1441, 1359, 1172, 1148, 755, 689 cm^{-1}; ^1H NMR (500 MHz, $CDCl_3$) δ 7.46–7.40 (2H, m, Ar**H**), 7.36–7.26 (6H, m, Ar**H**), 7.19–7.14 (2H, m, Ar**H**), 3.47 (2H, s, PhC**H**$_2$), 2.89 (2H, s, ≡CC**H**$_2$), 2.27 (6H, s, 2 × C**H**$_3$); ^{13}C NMR (101 MHz, $CDCl_3$) δ 204.7 (2 × C), 135.6 (C), 131.6 (2 × CH), 129.7 (2 × CH), 128.5 (2 × CH), 128.3 (2 × CH), 128.2 (CH), 127.1 (CH), 122.9 (C), 84.8 (C), 84.7 (C), 71.3 (C), 36.9 (CH_2), 27.4 (2 × CH_3), 21.7 (CH_2); HRMS (ESI) Exact mass calculated for $C_{21}H_{20}NaO_2^+$ $[M+Na]^+$: 327.1356, found: 327.1355.

Synthesis of Substrate 1e

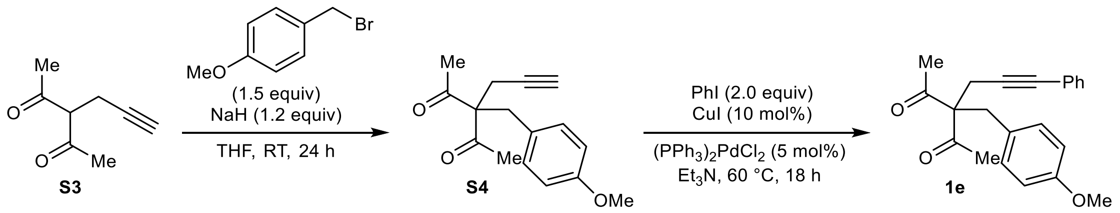

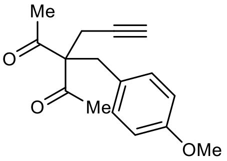

3-(4-Methoxybenzyl)-3-(prop-2-yn-1-yl)pentane-2,4-dione (S4). 3-(Prop-2-yn-1-yl)pentane-2,4-dione (**S3**,[5] 690 mg, 5.00 mmol) was added dropwise *via* syringe (using 2 mL of THF as a rinse) to a solution of NaH (60% in mineral oil, 240 mg, 6.00 mmol) in THF (30 mL) at 0 °C under argon and the resulting mixture was stirred at room temperature for 1 h. 4-Methoxybenzyl bromide[6] (1.08 mL, 7.50 mmol) was added dropwise and the solution was stirred at room temperature for 24 h, quenched with saturated aqueous NH_4Cl solution (50 mL), and extracted into Et_2O (3 × 50 mL). The combined organic layers were washed with brine (50 mL), dried ($MgSO_4$), filtered, and concentrated *in vacuo.* Purification of the residue by column chromatography (20% EtOAc/petroleum ether) gave *alkynyl 1,3-diketone* **S4** as a yellow oil (771 mg, 60%). R_f = 0.33 (20% EtOAc/pentane); IR (ATR) 2959, 1696 (C=O), 1513, 1360, 1266, 1171, 1151, 1027, 755, 690 cm^{-1}; ^{1}H NMR (500 MHz, $CDCl_3$) δ 7.03 (2H, d, *J* = 8.6 Hz, Ar**H**), 6.80 (2H, d, *J* = 8.3 Hz, Ar**H**), 3.78 (2H, s, OC$\mathbf{H}_3$), 3.34 (2H, s, ArC$\mathbf{H}_2$), 2.64 (2H, d, *J* = 2.7 Hz, ≡CC$\mathbf{H}_2$), 2.20 (6H, s, 2 × C$\mathbf{H}_3$C=O), 2.14 (1H, t, *J* = 2.7 Hz, C≡C**H**); ^{13}C NMR (101 MHz, $CDCl_3$) δ 204.6 (2 × C), 158.7 (C), 130.6 (2 × CH), 127.3 (C), 113.9 (2 × CH), 79.7 (C), 72.6 (C), 71.1 (CH), 55.2 (CH_3), 35.8 (CH_2), 27.4 (2 × CH_2), 20.7 (CH_2); HRMS (ESI) Exact mass calculated for $C_{16}H_{18}NaO_3^+$ $[M+Na]^+$: 281.1148, found: 281.1149.

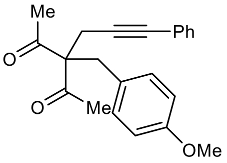

3-(4-Methoxybenzyl)-3-(3-phenylprop-2-yn-1-yl)pentane-2,4-dione (1e). A degassed solution of terminal alkyne **S4** (516 mg, 2.00 mmol), iodobenzene (448 µL, 4.00 mmol), $(PPh_3)_2PdCl_2$ (70.2 mg, 0.10 mmol), and CuI (38.1 mg, 0.20 mmol) in Et_3N (20 mL) was heated at 60 °C for 18 h. The reaction was cooled to room temperature, quenched with 1.0 M aqueous HCl solution (25 mL), and extracted into EtOAc (3 × 25 mL). The combined organic layers were washed with brine, dried ($MgSO_4$), filtered, and concentrated *in vacuo*. Purification of the residue by automated column chromatography (5% EtOAc/petroleum ether to 10% EtOAc/petroleum ether) gave *alkynyl 1,3-diketone* **1e** as a brown oil (525 mg, 79%). R_f = 0.20 (20% EtOAc/pentane); IR (ATR) 2997, 2958, 2933, 1720 (C=O), 1512, 1356, 1246, 1176, 1032, 756, 691 cm^{-1}; ^{1}H NMR (500 MHz, $CDCl_3$) δ 7.46–7.37 (2H, m, Ar**H**), 7.36–7.30 (3H, m, Ar**H**), 7.07 (2H, d, *J* = 8.7 Hz, Ar**H**), 6.81 (2H, d, *J* = 8.7 Hz, Ar**H**), 3.78 (3H, s, OC$\mathbf{H}_3$), 3.40 (2H, s, ArC$\mathbf{H}_2$), 2.87 (2H,

s, ≡CC**H**$_2$), 2.24 (6H, s, 2 × C**H**$_3$C=O); ^{13}C NMR (101 MHz, CDCl$_3$) δ 204.9 (2 × C), 158.7 (C), 131.6 (2 × CH), 130.7 (2 × CH), 128.3 (2 × CH), 128.2 (CH), 127.4 (C), 123.0 (C), 114.0 (2 × CH), 84.9 (C), 84.6 (C), 71.4 (C), 55.2 (CH$_3$), 36.1 (CH$_2$), 27.5 (2 × CH$_3$), 21.7 (CH$_2$); HRMS (ESI) Exact mass calculated for $C_{22}H_{22}NaO_3^+$ [M+Na]$^+$: 357.1461, found: 357.1460.

3-Methyl-3-[3-(*p*-tolyl)prop-2-yn-1-yl]pentane-2,4-dione (1f)

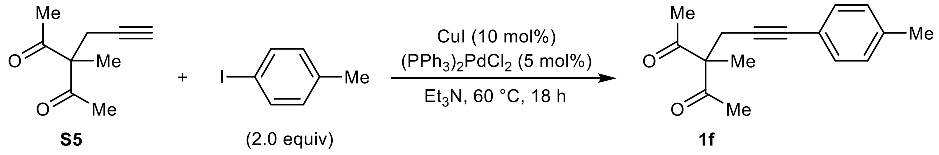

A degassed solution of terminal alkyne **S5**[2] (608 mg, 4.00 mmol), 4-iodotoluene (1.74 g, 8.00 mmol), (PPh$_3$)$_2$PdCl$_2$ (140 mg, 0.20 mmol), and CuI (76.2 mg, 0.40 mmol) in Et$_3$N (40 mL) was heated at 60 °C for 18 h. The reaction was cooled to room temperature, quenched with 1.0 M aqueous HCl solution (50 mL), and extracted into EtOAc (3 × 50 mL). The combined organic layers were washed with brine, dried (MgSO$_4$), filtered, and concentrated *in vacuo*. Purification of the residue by automated column chromatography (5% EtOAc/petroleum ether to 10% EtOAc/petroleum ether) gave *alkynyl 1,3-diketone* **1f** as an orange oil (415 mg, 43%). R_f = 0.21 (10% EtOAc/petroleum ether); IR (ATR) 3031, 2977, 2960, 2915, 1694 (C=O), 1509, 1355, 1160, 1097, 814, 589, 526 cm^{-1}; ^{1}H NMR (500 MHz, CDCl$_3$) δ 7.25 (2H, d, J = 8.2 Hz, Ar**H**), 7.09 (2H, d, J = 8.3 Hz, Ar**H**), 2.96 (2H, s, ≡CC**H**$_2$), 2.34 (3H, s, ArC**H**$_3$), 2.20 (6H, s, 2 × C**H**$_3$C=O), 1.54 (3H, s, C**H**$_3$CC=O); ^{13}C NMR (101 MHz, CDCl$_3$) δ 205.6 (2 × C), 138.2 (C), 131.4 (2 × CH), 129.0 (2 × CH), 119.9 (C), 84.0 (C), 83.9 (C), 66.4 (C), 26.6 (2 × CH$_3$), 25.4 (CH$_2$), 21.4 (CH$_3$), 18.7 (CH$_3$); HRMS (ESI) Exact mass calculated for $C_{16}H_{18}NaO_2^+$ [M+Na]$^+$: 265.1199, found: 265.1196.

3-[3-(4-Chlorophenyl)prop-2-yn-1-yl]-3-methylpentane-2,4-dione (1g)

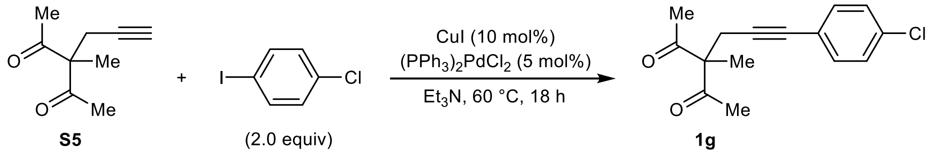

A degassed solution of terminal alkyne **S5**[2] (456 mg, 3.00 mmol), 4-chloro-1-iodobenzene (1.43 g, 6.00 mmol), (PPh$_3$)$_2$PdCl$_2$ (105 mg, 0.15 mmol), and CuI (57.1 mg, 0.30 mmol) in Et$_3$N (30 mL) was heated at 60 °C for 18 h. The reaction was cooled to room temperature, quenched with 1.0 M aqueous HCl (50 mL) solution, and extracted into EtOAc (3 × 50 mL). The combined organic layers were washed with brine, dried (MgSO$_4$), filtered, and concentrated *in vacuo*. Purification of the residue by automated column chromatography (5% EtOAc/petroleum ether to 10% EtOAc/petroleum ether) gave *alkynyl 1,3-diketone* **1g** as a yellow oil (373 mg, 47%). R_f = 0.20 (10% EtOAc/petroleum ether);

IR (ATR) 2980, 2937, 1698 (C=O), 1489, 1356, 1089, 827, 526 cm^{-1}; ^{1}H NMR (500 MHz, $CDCl_3$) δ 7.32–7.25 (4H, m, Ar**H**), 2.97 (2H, s, ≡CC**H**$_2$), 2.21 (6H, s, 2 × C**H**$_3$C=O), 1.54 (3H, s, C**H**$_3$CC=O); ^{13}C NMR (101 MHz, $CDCl_3$) δ 205.3 (2 × C), 134.1 (C), 132.8 (2 × CH), 128.5 (2 × CH), 121.4 (C), 85.9 (C), 82.7 (C), 66.3 (C), 26.5 (2 × CH_3), 25.3 (CH_2), 18.6 (CH_3); HRMS (ESI) Exact mass calculated for $C_{15}ClH_{15}NaO_2^+$ $[M+Na]^+$: 285.0653, found: 285.0645.

3-[3-(4-Methoxyphenyl)prop-2-yn-1-yl]-3-methylpentane-2,4-dione (1h)

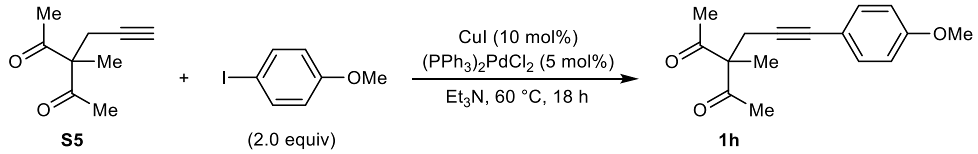

A degassed solution of terminal alkyne **S5**[2] (456 mg, 3.00 mmol), 4-iodoanisole (1.40 g, 6.00 mmol), $(PPh_3)_2PdCl_2$ (105 mg, 0.15 mmol), and CuI (57.1 mg, 0.30 mmol) in Et_3N (30 mL) was heated at 60 °C for 18 h. The reaction was cooled to room temperature, quenched with 1.0 M aqueous HCl solution (50 mL), and extracted into EtOAc (3 × 50 mL). The combined organic layers were washed with brine, dried ($MgSO_4$), filtered, and concentrated *in vacuo*. Purification of the residue by automated column chromatography (5% EtOAc/petroleum ether to 10% EtOAc/petroleum ether) gave *alkynyl 1,3-diketone* **1h** as a colorless oil (500 mg, 65%). R_f = 0.10 (10% EtOAc/petroleum ether); IR (ATR) 3097, 3031, 2993, 2959, 1721 (C=O), 1693 (C=O), 1509, 1245, 1091, 839, 679 cm^{-1}; ^{1}H NMR (500 MHz, $CDCl_3$) δ 7.29 (2H, d, J = 8.0 Hz, Ar**H**), 6.80 (2H, d, J = 8.0 Hz, Ar**H**), 3.80 (3H, s, OC**H**$_3$), 2.94 (2H, s, ≡CC**H**$_2$), 2.20 (6H, s, 2 × C**H**$_3$C=O), 1.53 (3H, s, C**H**$_3$CC=O); ^{13}C NMR (101 MHz, $CDCl_3$) δ 205.7 (2 × C), 159.4 (C), 132.9 (2 × CH), 115.1 (C), 113.8 (2 × CH), 83.6 (C), 83.2 (C), 66.4 (C), 55.3 (CH_3), 26.6 (2 × CH_3), 25.4 (CH_2), 18.7 (CH_3); HRMS (ESI) Exact mass calculated for $C_{15}ClH_{15}NaO_3^+$ $[M+Na]^+$: 281.1148, found: 281.1148.

3-{3-[(1,1'-Biphenyl)-4-yl]prop-2-yn-1-yl}-3-methylpentane-2,4-dione (1i)

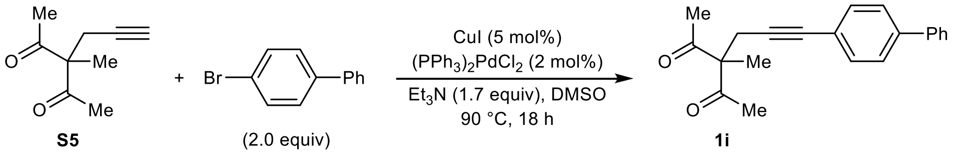

A degassed solution of terminal **S5**[2] (456 mg, 3.00 mmol), 4-bromo-1,1'-biphenyl (762 mg, 3.30 mmol), $(PPh_3)_2PdCl_2$ (42.1 mg, 0.06 mmol), CuI (29 mg, 0.15 mmol), and Et_3N (0.71 mL, 5.1 mmol) in DMSO (10 mL) was heated at 90 °C for 18 h. The reaction was cooled to room temperature, quenched with 1.0 M aqueous HCl solution (50 mL), and extracted into EtOAc (3 × 10 mL). The combined organic layers were washed with brine, dried ($MgSO_4$), filtered, and concentrated *in vacuo*. Purification of the residue by automated column chromatography (5% EtOAc/petroleum ether to 10%

EtOAc/petroleum ether) gave *alkynyl 1,3-diketone* **1i** as a white soild (711 mg, 78%). R*f* = 0.21 (10% EtOAc/petroleum ether); m.p. (Et_2O) 78–79 ˚C; IR (ATR) 1690 (C=O), 1485, 1358, 1213, 1159, 1097, 962, 839, 686 cm^{-1}; ^{1}H NMR (400 MHz, $CDCl_3$) δ 7.59–7.54 (2H, m, Ar**H**), 7.54–7.48 (2H, m, Ar**H**), 7.46–7.39 (4H, m, Ar**H**), 7.38–7.32 (1H, m, Ar**H**), 2.99 (2H, s, ≡CC**H**$_2$), 2.21 (6H, s, 2 × C**H**$_3$C=O), 1.56 (3H, s, C**H**$_3$CC=O); ^{13}C NMR (101 MHz, $CDCl_3$) δ 205.6 (2 × C), 140.9 (C), 140.3 (C), 132.0 (2 × CH), 128.8 (2 × CH), 127.6 (2 × CH), 127.0 (2 × CH), 126.9 (C), 121.9 (C), 110.0 (C), 85.5 (C), 83.7 (C), 66.4 (C), 26.6 (2 × CH_3), 25.4 (CH_2), 18.7 (CH_3); HRMS (ESI) Exact mass calculated for $[C_{21}H_{20}NaO_2]^+$ $[M+Na]^+$: 327.1356, found 327.1347.

3-[3-(2-Fluorophenyl)prop-2-yn-1-yl]-3-methylpentane-2,4-dione (1j)

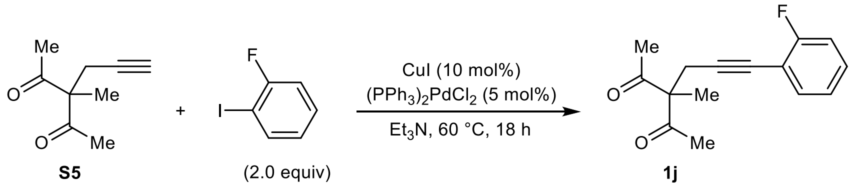

A degassed solution of terminal **S5**[2] (456 mg, 3.00 mmol), 1-fluoro-2-iodobenzene (700 µL, 6.00 mmol), $(PPh_3)_2PdCl_2$ (105 mg, 0.15 mmol), and CuI (57.1 mg, 0.30 mmol) in Et_3N (30 mL) was heated at 60 °C for 18 h. The reaction was cooled to room temperature, quenched with 1.0 M aqueous HCl (50 mL) solution, and extracted into EtOAc (3 × 50 mL). The combined organic layers were washed with brine, dried ($MgSO_4$), filtered, and concentrated *in vacuo*. Purification of the residue by automated column chromatography (5% EtOAc/petroleum ether to 10% EtOAc/petroleum ether) gave *alkynyl 1,3-diketone* **1j** as a colorless oil (526 mg, 71%). R*f* = 0.12 (10% EtOAc/petroleum ether); IR (ATR) 2980, 2937, 1698 (C=O), 1492, 1357, 1215, 757, 694 cm^{-1}; ^{1}H NMR (500 MHz, $CDCl_3$) δ 7.38–7.32 (1H, m, Ar**H**), 7.31–7.24 (1H, m, Ar**H**), 7.11–7.00 (2H, m, Ar**H**), 3.01 (2H, s, ≡CC**H**$_2$), 2.22 (6H, s, 2 × C**H**$_3$C=O), 1.57 (3H, s, C**H**$_3$CC=O); ^{13}C NMR (101 MHz, $CDCl_3$) δ 205.5 (2 × C), 162.9 (C, J_{CF} = 251 Hz), 133.4 (CH), 129.8 (CH, J_{CF} = 7.5 Hz), 123.8 (CH, J_{CF} = 3.7 Hz), 115.4 (CH, J_{CF} = 20.7 Hz), 111.5 (CH, J_{CF} = 16.0 Hz), 90.4 (C), 77.2 (C), 66.2 (C), 26.6 (2 × C), 25.6 (CH_2), 18.7 (CH_3); ^{19}F NMR (376 MHz, $CDCl_3$) δ –111.5; HRMS (ESI) Exact mass calculated for $C_{15}FH_{15}NaO_2^+$ $[M+Na]^+$: 269.0948, found: 269.0947.

3-[3-(3,5-Dimethylphenyl)prop-2-yn-1-yl]-3-methylpentane-2,4-dione (1k)

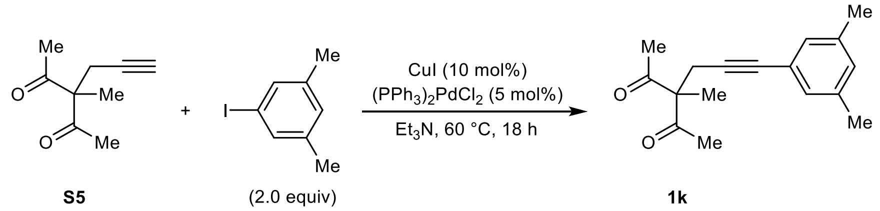

A degassed solution of terminal alkyne **S5**[2] (456 mg, 3.00 mmol), 1-iodo-3,5-dimethylbenzene (0.87 mL, 6.00 mmol), $(PPh_3)_2PdCl_2$ (105 mg, 0.15 mmol), and CuI (57.1 mg, 0.30 mmol) in Et_3N (30 mL) was heated at 60 °C for 18 h. The reaction was cooled to room temperature, quenched with 1.0 M aqueous HCl solution (50 mL), and extracted into EtOAc (3 × 50 mL). The combined organic layers were washed with brine, dried ($MgSO_4$), filtered, and concentrated *in vacuo*. Purification of the residue by automated column chromatography (5% EtOAc/petroleum ether to 10% EtOAc/petroleum ether) gave *alkynyl 1,3-diketone* **1k** as a yellow oil (537 mg, 70%). R_f = 0.21 (10% EtOAc/petroleum ether); IR (ATR) 3031, 2978, 2919, 2867, 1699 (C=O), 1598, 1355, 1162, 1092, 849, 690, cm^{-1}; ^1H NMR (500 MHz, $CDCl_3$) δ 6.99 (2H, s, Ar**H**), 6.93 (1H, s, Ar**H**), 2.95 (2H, s, ≡CC**H**$_2$), 2.27 (6H, s, 2 × ArC**H**$_3$), 2.21 (6H, s, 2 × C**H**$_3$C=O), 1.54 (3H, s, C**H**$_3$CC=O); ^{13}C NMR (101 MHz, $CDCl_3$) δ 205.6 (2 × C), 137.8 (2 × C), 130.0 (CH), 129.2 (2 × CH), 122.6 (C), 84.1 (C), 84.0 (C), 66.4 (C), 26.6 (2 × CH_3), 25.3 (CH_2), 21.0 (2 × CH_3), 18.7 (CH_3); HRMS (ESI) Exact mass calculated for $C_{17}H_{20}NaO_2^+$ $[M+Na]^+$: 279.1356, found: 279.1349.

3-[3-(Benzo[*d*][1,3]dioxol-5-yl)prop-2-yn-1-yl]-3-methylpentane-2,4-dione (1l)

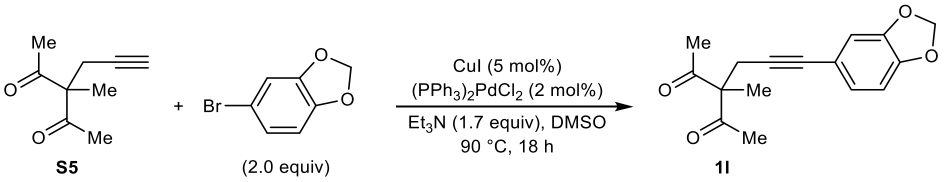

A degassed solution of terminal alkyne **S5**[2] (456 mg, 3.00 mmol), 5-bromobenzo[*d*][1,3]dioxole (660 mg, 3.30 mmol), $(PPh_3)_2PdCl_2$ (42.1 mg, 0.06 mmol), CuI (29 mg, 0.15 mmol), and Et_3N (0.71 mL, 5.1 mmol) in DMSO (10 mL) was heated at 90 °C for 18 h. The reaction was cooled to room temperature, quenched with 1.0 M aqueous HCl solution (50 mL), and extracted into EtOAc (3 × 10 mL). The combined organic layers were washed with brine, dried ($MgSO_4$), filtered, and concentrated *in vacuo*. Purification of the residue by automated column chromatography (5% EtOAc/petroleum ether to 10% EtOAc/petroleum ether) gave *alkynyl 1,3-diketone* **1l** as a yellow solid (481 mg, 75%). R_f = 0.21 (10% EtOAc/petroleum ether); m.p. (Et_2O) 69–70 ˚C IR (ATR) 2918, 1697 (C=O), 1502, 1481, 1361, 1251, 1210, 1031, 929, 872, 814, 698 cm^{-1}; ^1H NMR (400 MHz, $CDCl_3$) δ 6.87 (1H, dd, J = 8.0, 1.6 Hz, Ar**H**), 6.79 (1H, d, J = 1.5 Hz, Ar**H**), 6.71 (1H, d, J = 8.0 Hz, Ar**H**), 5.94 (2H, s, OC**H**$_2$O), 2.93 (2H, s, ≡CC**H**$_2$), 2.19 (6H, s, 2 × C**H**$_3$C=O), 1.52 (3H, s, C**H**$_3$CC=O); ^{13}C

NMR (101 MHz, $CDCl_3$) δ 205.6 (2 × C), 147.7 (C), 147.3 (C), 126.1 (C), 116.2 (CH), 111.6 (CH), 108.3 (CH), 101.2 (OCH_2), 83.6 (C), 83.1 (C), 66.4 (C), 26.6 (2 × CH_3), 25.3 (CH_2), 18.7 (CH_3); HRMS (ESI) Exact mass calculated for $[C_{16}H_{17}O_4]^+$ $[M+H]^+$: 273.1121, found 273.1116.

3-Methyl-3-[3-(naphthalen-2-yl)prop-2-yn-1-yl]pentane-2,4-dione (1m)

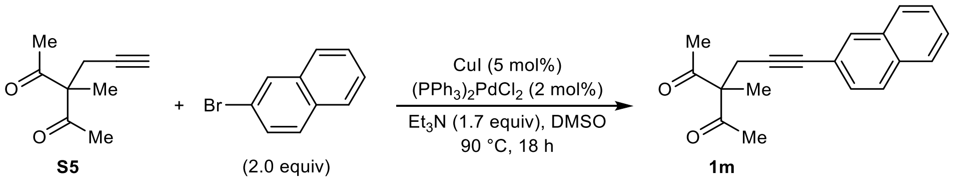

A degassed solution of terminal alkyne **S5**[2] (456 mg, 3.00 mmol), 2-bromonaphthalene (677 mg, 3.3 mmol), $(PPh_3)_2PdCl_2$ (42.1 mg, 0.06 mmol), CuI (29 mg, 0.15 mmol), and Et_3N (0.71 mL, 5.1 mmol) in DMSO (10 mL) was heated at 90 °C for 18 h. The reaction was cooled to room temperature, quenched with 1.0 M aqueous HCl solution (50 mL), and extracted into EtOAc (3 × 10 mL). The combined organic layers were washed with brine, dried ($MgSO_4$), filtered, and concentrated *in vacuo*. Purification of the residue by automated column chromatography (5% EtOAc/petroleum ether to 10% EtOAc/petroleum ether) gave *alkynyl 1,3-diketone* **1m** as a yellow soild (711 mg, 78%). R_f = 0.21 (10% EtOAc/petroleum ether); m.p. (Et_2O) 42–43 ˚C; IR (ATR) 1698 (C=O), 1356, 1210, 1163, 959, 858, 815, 750 cm^{-1}; ^{1}H NMR (400 MHz, $CDCl_3$) δ 7.87 (1H, s, Ar**H**), 7.78–7.72 (2H, m, Ar**H**), 7.50–7.45 (2H, m, Ar**H**), 7.39 (1H, dd, J = 8.4, 1.4 Hz, Ar**H**), 3.02 (2H, s, ≡CC**H**$_2$), 2.23 (6H, s, 2 × C**H**$_3$C=O), 1.56 (3H, s, C**H**$_3$CC=O); ^{13}C NMR (101 MHz, $CDCl_3$) δ 205.6 (2 × C), 132.7 (C), 131.3 (C), 128.4 (CH), 127.9 (CH), 127.7 (CH), 127.6 (CH), 126.6 (CH), 126.5 (CH), 120.2 (CH), 110.0 (C), 85.2 (C), 84.2 (C), 66.4 (C), 26.6 (2 × CH_3), 25.4 (CH_2), 18.7 (CH_3); HRMS (ESI) Exact mass calculated for $[C_{19}H_{18}NaO_2]^+$ $[M+Na]^+$: 301.1199, found 301.1194.

3-Methyl-3-[3-(naphthalen-1-yl)prop-2-yn-1-yl]pentane-2,4-dione (1n)

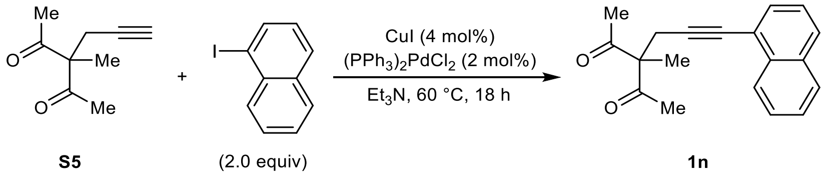

A degassed solution of terminal alkyne **S5**[2] (456 mg, 3.00 mmol), 1-iodonaphthalene (0.87 mL, 6.00 mmol), $(PPh_3)_2PdCl_2$ (42.6 mg, 0.06 mmol), and CuI (22.9 mg, 0.12 mmol) in Et_3N (30 mL) was heated at 60 °C for 18 h. The reaction was cooled to room temperature, quenched with saturated aqueous NH_4Cl solution (50 mL), and extracted into EtOAc (3 × 50 mL). The combined organic layers were washed with brine, dried ($MgSO_4$), filtered, and concentrated *in vacuo*. Purification of the residue by automated column chromatography (5% EtOAc/petroleum ether to 10% EtOAc/petroleum ether) gave *alkynyl 1,3-diketone* **1n** as a brown oil (423 mg, 51%). R_f = 0.19 (10%

EtOAc/petroleum ether); IR (ATR) 3058, 2978, 2932, 1698 (C=O), 1355, 910, 799, 773 cm^{-1}; ^{1}H NMR (500 MHz, $CDCl_3$) δ 8.20 (1H, dd, *J* = 8.3, 1.1 Hz, Ar**H**), 7.97-7.72 (2H, m, Ar**H**), 7.65-7.50 (3H, m, Ar**H**), 7.40 (1H, dd, *J* = 8.3, 7.1 Hz, Ar**H**), 3.14 (2H, s, ≡CC**H**$_2$), 2.25 (6H, s, 2 × ArC**H**$_3$), 1.64 (3H, s, C**H**$_3$CC=O); ^{13}C NMR (101 MHz, $CDCl_3$) δ 205.5 (2 × C), 133.3 (C), 133.1 (C), 130.5 (CH), 128.5 (CH), 128.2 (CH), 126.8 (CH), 126.3 (CH), 125.9 (CH), 125.1 (CH), 120.6 (C), 89.7 (C), 81.9 (C), 66.5 (C), 26.6 (2 × CH_3), 25.7 (CH_2), 18.8 (CH_3); HRMS (ESI) Exact mass calculated for $C_{19}H_{18}NaO_2^+$ [M+Na]$^+$: 301.1199, found: 301.1192.

3-Methyl-3-[3-(thiophen-2-yl)prop-2-yn-1-yl]pentane-2,4-dione (1o)

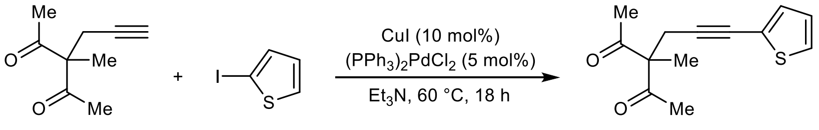

A degassed solution of terminal alkyne **S5**[2] (456 mg, 3.00 mmol), 2-iodothiophene (663 µL, 6.00 mmol), $(PPh_3)_2PdCl_2$ (105 mg, 0.15 mmol), and CuI (57.1 mg, 0.30 mmol) in Et_3N (30 mL) was heated at 60 °C for 18 h. The reaction was cooled to room temperature, quenched with 1.0 M aqueous HCl solution (50 mL), and extracted into EtOAc (3 × 50 mL). The combined organic layers were washed with brine, dried ($MgSO_4$), filtered, and concentrated *in vacuo*. Purification of the residue by automated column chromatography (5% EtOAc/petroleum ether to 10% EtOAc/petroleum ether) gave *alkynyl 1,3-diketone* **1o** as a yellow oil (445 mg, 63%). R_f = 0.17 (10% EtOAc/petroleum ether); IR (ATR) 2979, 2936, 1698 (C=O), 1304, 1163, 1091, 848, 701 cm^{-1}; ^{1}H NMR (500 MHz, $CDCl_3$) δ 7.20 (1H, dd, *J* = 5.2, 1.2 Hz, Ar**H**), 7.12 (1H, dd, *J* = 3.6, 1.2 Hz, Ar**H**), 6.94 (1H, dd, *J* = 5.2, 3.6 Hz, Ar**H**), 2.99 (2H, s, ≡CC**H**$_2$), 2.20 (6H, s, 2 × C**H**$_3$C=O), 1.54 (3H, s, C**H**$_3$CC=O); ^{13}C NMR (101 MHz, $CDCl_3$) δ 205.4 (2 × C), 131.7 (CH), 126.8 (CH), 126.6 (CH), 122.9 (C), 88.9 (C), 77.0 (C), 66.2 (C), 26.6 (2 × CH_3), 25.6 (CH_2), 18.7 (CH_3); HRMS (ESI) Exact mass calculated for $C_{13}H_{14}NaO_2S^+$ [M+Na]$^+$: 257.0607, found: 257.0599.

3-Methyl-3-[3-(pyridin-2-yl)prop-2-yn-1-yl]pentane-2,4-dione (1p)

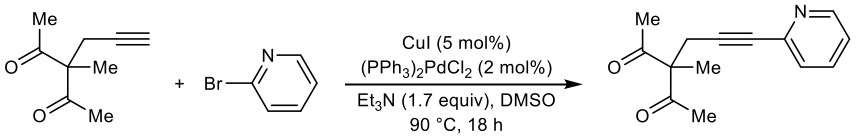

A degassed solution of terminal alkyne **S5**[2] (456 mg, 3.00 mmol), 2-bromopyridine (518 mg, 3.30 mmol), $(PPh_3)_2PdCl_2$ (42.1 mg, 0.06 mmol), CuI (29 mg, 0.15 mmol), and Et_3N (0.71 mL, 5.1 mmol) in DMSO (10 mL) was heated at 90 °C for 18 h. The reaction was cooled to room temperature, quenched with 1.0 M aqueous HCl solution (50 mL), and extracted into EtOAc (3 × 10 mL). The

combined organic layers were washed with brine, dried ($MgSO_4$), filtered, and concentrated *in vacuo*. Purification of the residue by automated column chromatography (5% EtOAc/petroleum ether to 10% EtOAc/petroleum ether) gave *alkynyl 1,3-diketone* **1p** as a yellow oil (481 mg, 70%). R_f = 0.21 (10% EtOAc/petroleum ether); IR (ATR) 2976, 1698 (C=O), 1582, 1464, 1428, 1357, 1165, 1092, 957, 778, 740 cm^{-1}; ^{1}H NMR (400 MHz, $CDCl_3$) δ 8.53 (1H, s, Ar**H**), 7.61 (1H, tt, *J* = 7.8, 1.7 Hz, Ar**H**), 7.34 (1H, t, *J* = 9.8 Hz, Ar**H**), 7.23–7.17 (1H, m, Ar**H**), 3.00 (2H, d, *J* = 2.2 Hz, ≡CCH_2), 2.20 (6H, d, *J* = 2.0 Hz, 2 × CH_3C=O), 1.56 (3H, d, *J* = 2.0 Hz, CH_3CC=O); ^{13}C NMR (101 MHz, $CDCl_3$) δ 205.3 (2 × C), 149.9 (CH), 143.0 (C), 136.1 (CH), 127.1 (CH), 122.8 (CH), 85.4 (C), 83.4 (C), 66.2 (C), 26.5 (2 × CH_3), 25.2 (CH_2), 18.7 (CH_3); HRMS (ESI) Exact mass calculated for $[C_{14}H_{15}NNaO_2]^+$ $[M+Na]^+$: 252.0995, found 252.0990.

4-Methyl-4-(3-phenylprop-2-yn-1-yl)heptane-3,5-dione (1q)

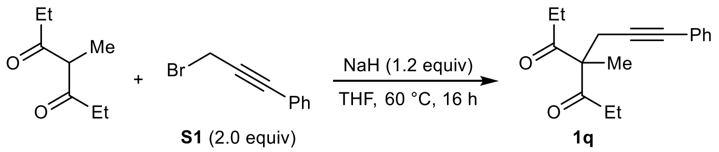

4-Methylheptane-3,5-dione (**S6**,[7] 710 mg, 5.00 mmol) was added dropwise *via* syringe (using 2 mL of THF as a rinse) to a solution of NaH (60% in mineral oil, 240 mg, 6.00 mmol) in THF (30 mL) at 0 °C under argon and the resulting mixture was stirred at room temperature for 1 h. Alkynyl bromide **S1**[3] (80% wt. solution in toluene, 2.44 g, 10.0 mmol) was added dropwise *via* syringe (using 2 mL of THF as a rinse) and the solution was stirred at 60 °C for 16 h. The reaction was cooled to room temperature, quenched with saturated aqueous NH_4Cl solution (50 mL), and extracted into Et_2O (3 × 50 mL). The combined organic layers were washed with brine (50 mL), dried ($MgSO_4$), filtered, and concentrated *in vacuo.* Purification of the residue by column chromatography (100% petroleum ether to 10% EtOAc/petroleum ether) gave *alkynyl 1,3-diketone* **1q** as a yellow oil (1.03 g, 78%). R_f = 0.35 (10% EtOAc/pentane); IR (ATR) 2977, 1698 (C=O), 1490, 1347, 1202, 1088, 964, 755, 690 cm^{-1}; ^{1}H NMR (400 MHz, $CDCl_3$) δ 7.35–7.32 (2H, m, Ar**H**), 7.29–7.26 (3H, m, Ar**H**), 2.98 (2H, s, ≡CCH_2), 2.52–2.45 (4H, m, 2 × CH_2CH_3), 1.54 (3H, s, CH_3CC=O), 1.06 (6H, t, *J* = 7.2 Hz, 2 × CH_2CH_3); ^{13}C NMR (101 MHz, $CDCl_3$) δ 208.4 (2 × C), 131.6 (CH), 128.2 (CH), 128.0 (CH), 123.1 (C), 85.2 (C), 83.7 (C), 65.7 (C), 32.1 (CH_2), 25.5 (CH_2), 22.4 (CH_2), 18.8 (CH_3), 8.0 (2 × CH_3); HRMS (ESI) Exact mass calculated for $[C_{17}H_{20}NaO_2]^+$ $[M+Na]^+$: 279.1356, found 279.1356.

2-Methyl-1,3-diphenyl-2-(3-phenylprop-2-yn-1-yl)propane-1,3-dione (1r)

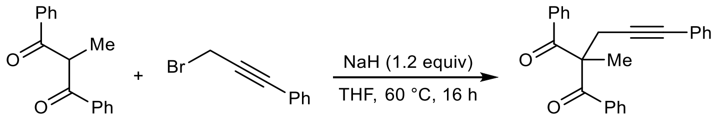

2-Methyl-1,3-diphenylpropane-1,3-dione (**S7**,[8] 1.19 g, 5.00 mmol) was added dropwise *via* syringe (using 2 mL of THF as a rinse) to a solution of NaH (60% in mineral oil, 240 mg, 6.00 mmol) in THF (30 mL) at 0 °C under argon and the resulting mixture was stirred at room temperature for 1 h. Alkynyl bromide **S1**[3] (80% wt. solution in toluene, 2.43 g, 10.0 mmol) was added dropwise *via* syringe (using 2 mL of THF as a rinse) and the solution was stirred at 60 °C for 16 h. The reaction was cooled to room temperature, quenched with saturated aqueous NH_4Cl solution (50 mL), and extracted into Et_2O (3 × 50 mL). The combined organic layers were washed with brine (50 mL), dried ($MgSO_4$), filtered, and concentrated *in vacuo*. Purification of the residue by column chromatography (100% petroleum ether to 10% EtOAc/petroleum ether) gave *alkynyl 1,3-diketone* **1r** as a yellow solid (1.41 g, 80%) whose spectroscopic data were consistent with those reported previously.[11]

6-Phenylhex-5-yn-2-one (6)

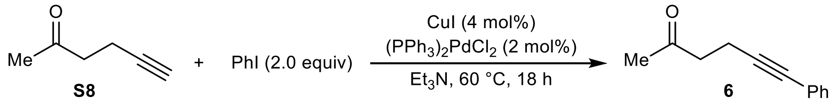

A degassed solution of hex-5-yn-2-one[9] (**S8**, 288 mg, 3.00 mmol), iodobenzene (669 µL, 6.00 mmol), $(PPh_3)_2PdCl_2$ (42.6 mg, 0.06 mmol), and CuI (22.9 mg, 0.12 mmol) in Et_3N (30 mL) was heated at 60 °C for 18 h. The reaction was cooled to room temperature, quenched with saturated aqueous NH_4Cl solution (25 mL), and extracted into EtOAc (3 × 25 mL). The combined organic layers were washed with brine, dried ($MgSO_4$), filtered, and concentrated *in vacuo*. Purification of the residue by column chromatography (5% EtOAc/petroleum ether to 10% EtOAc/petroleum ether) gave alkynyl ketone **6** as a yellow oil (194 mg, 38%) whose spectroscopic data were consistent with those reported previously.[10]

Enantioselective Arylative Cyclizations of Alkynyl 1,3-Diketones

General Procedure A

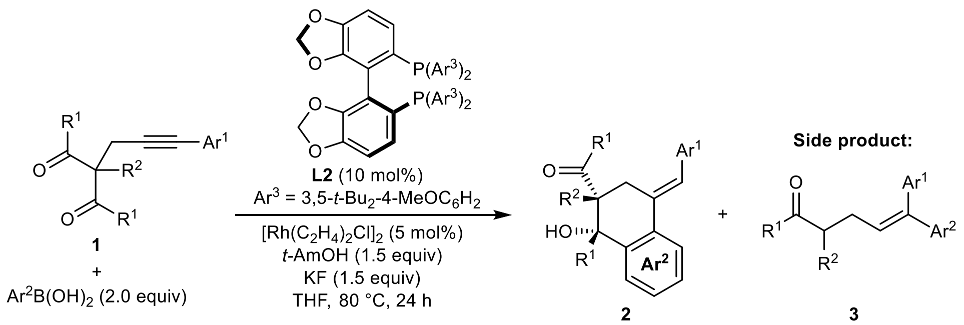

$[Rh(C_2H_4)_2Cl]_2$ (5.8 mg, 0.015 mmol) and (*S*)-DTBM-SEGPHOS (**L2**, 35.4 mg, 0.03 mmol) were added to an oven-dried microwave vial, which was sealed with a septum and purged with argon for 30 min. Degassed THF (5.5 mL) was added and the mixture was stirred at room temperature for 30 min under argon. In a separate oven-dried microwave vial the alkynyl 1,3-diketone **1** (0.30 mmol), boronic acid (0.60 mmol), and KF (26.1 mg, 0.45 mmol) were added. A septum was fitted and the vial was purged with argon for 30 min. The catalyst solution was added to the vial containing the alkynyl 1,3-diketone using additional THF (0.5 mL) as a rinse. *t*-Amyl alcohol (49 µL, 0.45 mmol) was added and the mixture was stirred at 80 °C for 24 h. The reaction was cooled to room temperature, diluted with H_2O and saturated aqueous NH_4Cl solution and extracted with EtOAc. The combined organic layers were washed with brine, dried (Na_2SO_4), filtered, and concentrated *in vacuo*. Purification of the residue by column chromatography gave the arylative cyclization product. In most cases, small quantities of minor products **3** were also observed in the ^{1}H NMR spectra of the crude material, but with the exception of the reaction forming arylative cyclization product **2a** (*vide infra*), they were not isolated.

1-{(1*S*,2*R*)-4-[(*E*)-Benzylidene]-1-hydroxy-1,2-dimethyl-1,2,3,4-tetrahydronaphthalen-2-yl}ethanone (1a) and 3-methyl-6,6-diphenylhex-5-en-2-one (2a) and 3-methyl-6,6-diphenylhex-5-en-2-one (3a)

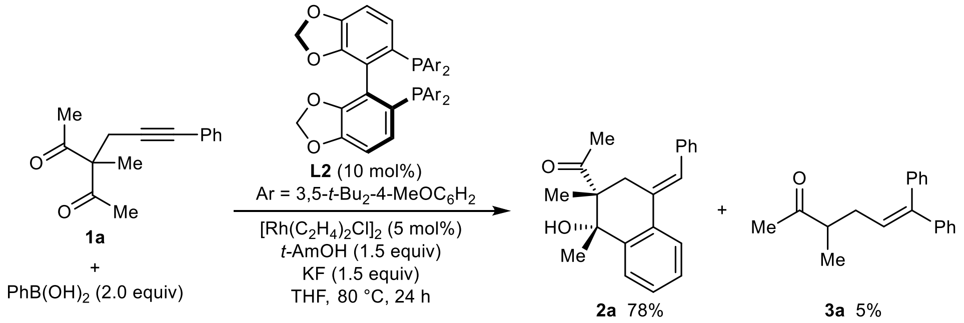

General Procedure A was followed using alkynyl 1,3-diketone **1a** (68.5 mg, 0.30 mmol) and phenylboronic acid (73.2 mg, 0.60 mmol). Purification by column chromatography (5% to 10% EtOAc/pentane) gave *ketone* **3a** as a colorless oil (4.3 mg, 5%) followed by *arylative cyclization product* **2a** as a yellow solid (71.5 mg, 78%).

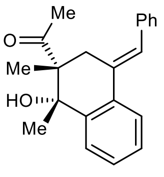

1-{(1*S*,2*R*)-4-[(*E*)-Benzylidene]-1-hydroxy-1,2-dimethyl-1,2,3,4-tetrahydronaphthalen-2-yl}ethanone (2a). R_f = 0.12 (10% EtOAc/pentane); m.p. 128–129 °C (MeOH); $[\alpha]_D^{20.5}$ –244 (*c* 1.00, $CHCl_3$); IR (ATR) 3476 (OH), 1682 (C=O), 1454, 1376, 1353, 1076, 929, 960, 694, 580, 538 cm^{-1}; ^1H NMR (500 MHz, $CDCl_3$) δ 7.80 (1H, dd, *J* = 7.8, 1.4 Hz, Ar**H**), 7.65 (1H, d, *J* = 7.9 Hz, Ar**H**), 7.44–7.41 (2H, m, Ar**H**), 7.38–7.37 (2H, m, Ar**H**), 7.35–7.29 (2H, m, Ar**H**), 7.25–7.21 (1H, m, Ar**H**), 7.17 (1H, d, *J* = 2.6 Hz, C=C**H**), 4.87 (1H, d, *J* = 1.4 Hz, O**H**), 3.41 (1H, dd, *J* = 16.5, 1.2 Hz, C$\mathbf{H}_a$$H_b$), 2.94 (1H, dd, *J* = 16.5, 2.6 Hz, C$H_a$$\mathbf{H}_b$), 1.89 (3H, s, C$\mathbf{H}_3$C=O), 1.41 (3H, d, *J* = 1.4 Hz, C$\mathbf{H}_3$COH), 1.36 (3H, s, C$\mathbf{H}_3$CC=O); ^{13}C NMR (101 MHz $CDCl_3$) δ 217.5 (C), 145.9 (C), 137.3 (C), 132.9 (C), 132.9 (C), 129.3 (2 × CH), 128.6 (2 × CH), 128.5 (CH), 127.1 (CH), 127.0 (CH), 125.7 (CH), 125.3 (CH), 123.3 (CH), 75.0 (C), 54.7 (C), 36.9 (CH_2), 26.8 (CH_3), 26.1 (CH_3), 18.8 (CH_3); HRMS (ESI) Exact mass calculated for $[C_{21}H_{22}NaO_2]^+$ $[M+Na]^+$: 329.1512, found 329.1510; Enantiomeric excess was determined by HPLC with a Chiralpak AS-H column (80:20 isohexane:*i*-PrOH, 0.8 mL/min, 280 nm, 25 °C): t_r (minor) = 6.6 min, t_r (major) = 7.6 min, 98% ee.

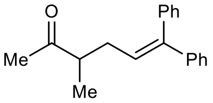

3-Methyl-6,6-diphenylhex-5-en-2-one (3a). R_f = 0.30 (10% EtOAc/pentane); IR (ATR) 2964, 2924, 2852, 1709 (C=O), 1494, 1359, 1074, 758, 698 cm^{-1}; ^1H NMR (400 MHz, $CDCl_3$) δ 7.46–7.29 (4H, m, Ar**H**), 7.26–7.15 (6H, m, Ar**H**), 6.01 (1H, t, *J* = 7.4 Hz, CH_2C**H**=), 2.63 (1H, quin, *J* = 6.8 Hz, O=CC**H**), 2.47 (1H, dt, *J* = 14.8, 6.8 Hz, C$\mathbf{H}_a$$H_b$CH=), 2.23 (1H, dt, *J* = 14.8, 7.5 Hz, C$H_a$$\mathbf{H}_b$CH=), 2.09 (3H, s, C$\mathbf{H}_3$C=O), 1.11 (3H, d, *J* = 7.0 Hz, CHC$\mathbf{H}_3$); δ

^{13}C NMR (101 MHz, $CDCl_3$) δ 211.8 (C), 143.6 (C), 142.4 (C), 139.8 (C), 129.8 (2 × CH), 128.3 (2 × CH), 128.1 (2 × CH), 127.2 (2 × CH), 127.1 (2 × CH), 126.2 (CH), 47.6 (CH), 32.7 (CH_2), 28.1 (CH_3), 15.9 (CH_3); HRMS (ESI) Exact mass calcd for $C_{19}H_{20}NaO^+$ $[M+Na]^+$: 287.1406, found: 287.1395.

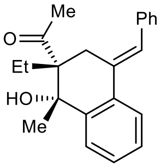

1-[(1*S*,2*R*,*E*)-4-Benzylidene-2-ethyl-1-hydroxy-1-methyl-1,2,3,4-tetrahydronaphthalen-2-yl]ethanone (2b). The title compound was prepared according to General Procedure A using alkynyl 1,3-diketone **1b** (72.6 mg, 0.30 mmol) and phenylboronic acid (73.2 mg, 0.60 mmol), and purified by column chromatography (5% to 10% EtOAc/*n*-pentane) to give a yellow solid (64.2 mg, 67%). R_f = 0.10 (10% EtOAc/pentane); m.p. (Et_2O) 96–97 °C; $[\alpha]_D^{20.5}$ –60 (*c* 1.00, $CHCl_3$); IR (ATR) 3437 (OH), 1687 (C=O), 1493, 1354, 1220, 1110, 911, 789, 757 cm^{-1}; ^1H NMR (400 MHz, $CDCl_3$) δ 7.75 (1H, dd, *J* = 7.8, 1.3 Hz, Ar**H**), 7.63 (1H, dd, *J* = 7.9, 1.2 Hz, Ar**H**), 7.43–7.40 (4H, m, Ar**H**), 7.34–7.29 (2H, m, Ar**H**), 7.25–7.21 (1H, m, Ar**H**), 7.12 (1H, s, C=C**H**), 4.84 (1H, d, *J* = 1.2 Hz, O**H**), 3.50 (1H, dd, *J* = 17.0, 1.4 Hz, C**H**$_a$H$_b$C=), 2.85 (1H, dd, *J* = 17.0, 2.6 Hz, CH$_a$**H**$_b$C=O), 2.28–2.18 (1H, m, C**H**$_a$H$_b$CH$_3$), 1.88 (3H, s, C**H**$_3$C=O), 1.72–1.62 (1H, 2H, m, CH$_a$**H**$_b$CH$_3$), 1.39 (3H, d, *J* = 1.2 Hz, C**H**$_3$COH), 1.00 (3H, t, *J* = 7.7 Hz, CH$_2$C**H**$_3$); ^{13}C NMR (101 MHz $CDCl_3$) δ 218.6 (C), 145.9 (C), 137.3 (C), 133.6 (C), 133.1 (C), 129.3 (2 × CH), 128.5 (2 × CH), 127.2 (CH), 127.1 (CH), 126.0 (CH), 124.9 (CH), 123.4 (CH), 75.8 (C), 57.5 (C), 35.4 (CH_2), 28.9 (CH_3), 28.0 (CH_3), 26.1 (CH_2), 10.1 (CH_3); HRMS (ESI) Exact mass calculated for $[C_{22}H_{24}NaO_2]^+$ $[M+Na]^+$: 343.1669, found 343.1666; Enantiomeric excess was determined by HPLC with a Chiralpak AD-H column (90:10 isohexane:*i*-PrOH, 1.0 mL/min, 280 nm, 25 °C): t_r (minor) = 5.8 min, t_r (major) = 6.5 min; 96% ee.

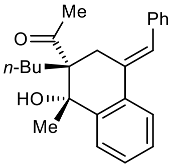

1-{(1*S*,2*R*)-4-[(*E*)-Benzylidene]-2-butyl-1-hydroxy-1-methyl-1,2,3,4-tetrahydronaphthalen-2-yl}ethan-1-one (2c). The title compound was prepared according to General Procedure A using alkynyl 1,3-diketone **1c** (81.0 mg, 0.30 mmol) and phenylboronic acid (73.2 mg, 0.60 mmol), and purified by column chromatography (10% EtOAc/pentane) to give a yellow oil (28.2 mg, 27%). R_f = 0.17 (10% EtOAc/pentane); $[\alpha]_D^{20.1}$ –64.0 (*c* 1.00, $CHCl_3$); IR (ATR) 3443 (OH), 2956, 2929, 2870, 1684 (C=O), 1354, 1038, 758, 697 cm^{-1}; ^1H NMR (400 MHz, $CDCl_3$) δ 7.75 (1H, dd, *J* = 7.8, 1.5 Hz, Ar**H**), 7.62 (1H, dd, *J* = 8.0, 1.3 Hz, Ar**H**), 7.47–7.38 (4H, m, Ar**H**), 7.36–7.28 (2H, m, Ar**H**), 7.29–7.19 (1H, m, Ar**H**), 7.12 (1H, t, *J* = 1.9 Hz, C=C**H**), 4.81 (1H, d, *J* = 1.3 Hz, O**H**), 3.49 (1H, dd, *J* = 17.0, 1.5 Hz, C**H**$_a$H$_b$C=), 2.87 (1H, dd, *J* = 17.0, 2.6 Hz, CH$_a$**H**$_b$C=), 2.16 (1H, td, *J* = 13.5, 4.3 Hz, C**H**$_a$H$_b$CH$_2$CH$_2$CH$_3$), 1.87 (3H, s, C**H**$_3$C=O), 1.61–1.57 (1H, m, CH$_a$**H**$_b$CH$_2$CH$_2$CH$_3$), 1.50–1.21 (7H, m, C**H**$_3$COH and C**H**$_2$C**H**$_2$CH$_3$), 0.91 (3H, t, *J* = 7.1 Hz, CH$_2$C**H**$_3$); ^{13}C NMR (101 MHz, $CDCl_3$) δ

218.5 (C), 145.9 (C), 137.4 (C), 133.6 (C), 133.2 (C), 129.3 (2 × CH), 128.54 (2 × CH), 128.53 (CH), 127.2 (CH), 127.1 (CH), 126.0 (CH), 125.0 (CH), 123.4 (CH), 75.7 (C), 57.4 (C), 35.8 (CH_2), 35.3 (CH_2), 28.7 (CH_3), 27.6 (CH_2), 26.2 (CH_3), 23.7 (CH_2), 13.9 (CH_3); HRMS (ESI) Exact mass calculated for $C_{24}H_{28}NaO_2^+$ $[M+Na]^+$: 371.1982, found: 371.1973; Enantiomeric excess was determined by HPLC with a Chiralpak AS-H column (80:20 isohexane:*i*-PrOH, 1.0 mL/min, 280 nm, 23 °C): t_r (minor) = 4.2 min, t_r (major) = 5.8 min, 95% ee.

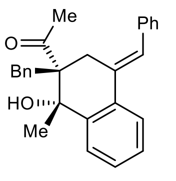

1-{(1*S*,2*R*)-2-Benzyl-4-[(*E*)-benzylidene]-1-hydroxy-1-methyl-1,2,3,4-tetrahydronaphthalen-2-yl}ethanone (2d). The title compound was prepared according to General Procedure A using alkynyl 1,3-diketone **1d** (91.2 mg, 0.30 mmol) and phenylboronic acid (73.2 mg, 0.60 mmol), and purified by column chromatography (5% to 10% EtOAc/pentane) to give a yellow solid (57.6 mg, 50%). R_f = 0.13 (10% EtOAc/pentane); m.p. 144–145 °C (Et_2O); $[\alpha]_D^{20.5}$ +96.0 (*c* 1.00, $CHCl_3$); IR (ATR) 3502 (OH), 1681 (C=O), 1492, 1362, 1229, 1069, 937, 867, 796, 699 cm^{-1}; ^1H NMR (400 MHz, $CDCl_3$) δ 7.76 (1H, dd, *J* = 7.7, 1.1 Hz, Ar**H**), 7.63 (1H, d, *J* = 7.8 Hz, Ar**H**), 7.48–7.41 (4H, m, Ar**H**), 7.36–7.21 (6H, m, Ar**H**), 7.16 (2H, d, *J* = 7.1 Hz, Ar**H**), 7.09 (1H, s, C=C**H**), 5.29 (1H, d, *J* = 1.2 Hz, O**H**), 3.65 (1H, d, *J* = 13.1 Hz, PhC**H**$_a$H$_b$), 3.51 (1H, d, *J* = 18.2 Hz, C**H**$_a$H$_b$C=), 3.05 (1H, dd, *J* = 18.2, 2.5 Hz, CH$_a$**H**$_b$C=), 2.88 (1H, d, *J* = 13.1 Hz, PhCH$_a$**H**$_b$), 1.44 (3H, s, C**H**$_3$), 1.37 (3H, s, C**H**$_3$); ^{13}C NMR (101 MHz $CDCl_3$) δ 220.8 (C), 145.3 (C), 137.3 (C), 136.6 (C), 134.3 (C), 132.7 (2 × CH), 130.1 (2 × CH), 129.3 (2 × CH), 128.48 (2 × CH), 128.46 (CH), 127.4 (CH), 127.2 (CH), 126.9 (CH), 126.6 (CH), 124.3 (CH), 123.6 (CH), 75.6 (C), 57.2 (C), 40.4 (CH_2), 35.0 (CH_2), 31.4 (CH_3), 25.1 (CH_3); HRMS (ESI) Exact mass calculated for $[C_{27}H_{26}NaO_2]^+$ $[M+Na]^+$: 405.1825, found 405.1821; Enantiomeric excess was determined by HPLC with a Chiralpak AS-H column (80:20 isohexane:*i*-PrOH, 0.8 mL/min, 280 nm, 25 °C): t_r (major) = 5.7 min, t_r (minor) = 7.2 min, 98% ee.

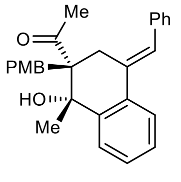

1-{(1*S*,2*R*)-4-[(*E*)-Benzylidene]-1-hydroxy-2-(4-methoxybenzyl)-1-methyl-1,2,3,4-tetrahydronaphthalen-2-yl}ethan-1-one (2e). The title compound was prepared according to General Procedure A using alkynyl 1,3-diketone **1e** (100 mg, 0.30 mmol) and phenylboronic acid (73.2 mg, 0.60 mmol), and purified by column chromatography (10% EtOAc/pentane) to give a yellow oil (65.6 mg, 53%). R_f = 0.15 (10% EtOAc/pentane); $[\alpha]_D^{20.3}$ +184 (*c* 1.00, $CHCl_3$); IR 3433 (OH), 2956, 2934, 1678 (C=O), 1511, 1353, 1246, 1032, 759, 693 (ATR) cm^{-1}; ^1H NMR (400 MHz, $CDCl_3$) δ 7.76 (1H, dd, *J* = 7.9, 1.4 Hz, Ar**H**), 7.64 (1H, dd, *J* = 7.9, 1.2 Hz, Ar**H**), 7.48–7.40 (4H, m, Ar**H**), 7.40–7.22 (3H, m, Ar**H**), 7.12–7.04 (3H, m, Ar**H** and C=C**H**), 6.84 (2H, d, *J* = 8.6 Hz, Ar**H**), 5.30 (1H, m, O**H**), 3.80 (3H, s, OC**H**$_3$), 3.61 (1H, d, *J* = 13.3 Hz, ArC**H**$_a$H$_b$), 3.54 (1H, dd, *J* = 18.2, 2.1 Hz, C**H**$_a$H$_b$C=), 3.04 (1H, dd, *J* = 18.2,

2.6 Hz, $CH_a\mathbf{H}_bC$=), 2.84 (1H, d, *J* = 13.3 Hz, $ArCH_a\mathbf{H}_b$), 1.43 (3H, d, *J* = 1.4 Hz, $C\mathbf{H}_3COH$), 1.41 (3H, s, $C\mathbf{H}_3C$=O); ^{13}C NMR (101 MHz, $CDCl_3$) δ 221.0 (C), 158.5 (C), 145.4 (C), 137.3 (C), 134.3 (C), 132.8 (C), 131.0 (2 × CH), 129.3 (2 × CH), 128.5 (2 × CH), 128.4 (CH), 128.3 (C), 127.4 (CH), 127.1 (CH), 126.5 (CH), 124.3 (CH), 123.6 (CH), 113.8 (2 × CH), 75.6 (C), 57.2 (C), 55.2 (CH_3), 39.5 (CH_2), 35.0 (CH_2), 31.5 (CH_3), 25.1 (CH_3); HRMS (ESI) Exact mass calculated for $C_{28}H_{28}NaO_3^+$ $[M+Na]^+$: 435.1931, found: 435.1927; Enantiomeric excess was determined by HPLC with a Chiralpak AS-H column (80:20 isohexane:*i*-PrOH, 0.8 mL/min, 280 nm, 23 °C): t_r (major) = 6.7 min, t_r (minor) = 8.2 min, 98% ee.

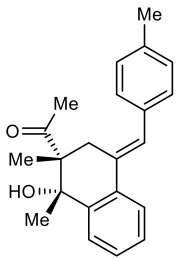

1-{1*S*,2*R*)-1-Hydroxy-1,2-dimethyl-4-[(*E*)-4-methylbenzylidene]-1,2,3,4-tetrahydronaphthalen-2-yl}ethan-1-one (2f). The title compound was prepared according to General Procedure A using alkynyl 1,3-diketone **1f** (72.6 mg, 0.30 mmol) and phenylboronic acid (73.2 mg, 0.60 mmol), and purified by column chromatography (10% EtOAc/pentane) to give a yellow solid (78.9 mg, 82%). R_f = 0.12 (10% EtOAc/pentane); m.p. 89–91 °C (Et_2O); $[\alpha]_D^{20.4}$ –216 (c 1.00, $CHCl_3$); IR (ATR) 3442 (OH), 3002, 2918, 2871, 1683 (C=O), 1510, 1354, 1072, 817, 699 cm^{-1}; ^1H NMR (400 MHz, $CDCl_3$) δ 7.81 (1H, dd, *J* = 7.8, 1.5 Hz, Ar**H**), 7.65 (1H, dd, *J* = 8.1, 1.3 Hz, Ar**H**), 7.35–7.31 (1H, m, Ar**H**) 7.30–7.27 (3H, m, Ar**H**), 7.27–7.22 (3H, Ar**H**), 7.15 (1H, br s, C=C**H**), 4.89 (1H, d, *J* = 1.3 Hz, O**H**), 3.43 (1H, dd, *J* = 16.6, 1.2 Hz, $C\mathbf{H}_aH_b$), 2.94 (1H, dd, *J* = 16.6, 2.6 Hz, $CH_a\mathbf{H}_b$), 2.42 (3H, s, $ArC\mathbf{H}_3$), 1.91 (3H, s, $C\mathbf{H}_3C$=O), 1.42 (3H, d, *J* = 1.3 Hz, $C\mathbf{H}_3COH$), 1.38 (3H, s, $C\mathbf{H}_3CC$=O); ^{13}C NMR (101 MHz, $CDCl_3$) δ 217.6 (C), 145.8 (C), 137.0 (C), 134.4 (C), 133.1 (C), 132.2 (C), 129.24 (2 × CH), 129.22 (2 × CH), 128.4 (CH), 127.0 (CH), 125.8 (CH), 125.3 (CH), 123.2 (CH), 75.1 (C), 54.7 (C), 37.0 (CH_2), 26.7 (CH_3), 26.1 (CH_3), 21.3 (CH_3), 18.9 (CH_3); HRMS (ESI) Exact mass calculated for $C_{22}H_{24}NaO_2^+$ $[M+Na]^+$: 343.1669, found: 343.1668; Enantiomeric excess was determined by HPLC with a Chiralpak AS-H column (80:20 isohexane:*i*-PrOH, 1.0 mL/min, 280 nm, 23 °C): t_r (minor) = 4.9 min, t_r (major) = 5.9 min, 97% ee.

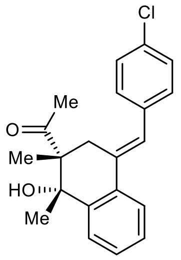

1-{(1*S*,2*R*)-4-[(*E*)-4-Chlorobenzylidene]-1-hydroxy-1,2-dimethyl-1,2,3,4-tetrahydronaphthalen-2-yl}ethan-1-one (2g). The title compound was prepared according to General Procedure A using alkynyl 1,3-diketone **1g** (81.0 mg, 0.30 mmol) and phenylboronic acid (73.2 mg, 0.60 mmol), and purified by column chromatography (10% EtOAc/pentane) to give a yellow solid (77.4 mg, 76%). R_f = 0.13 (10% EtOAc/pentane); m.p. 144–146 °C (Et_2O); $[\alpha]_D^{20.1}$ –228 (*c* 1.00, $CHCl_3$); IR (ATR) 3448 (OH), 2971, 2936, 1681 (C=O), 1562, 1354, 1073, 825, 691 cm^{-1}; ^1H NMR (400 MHz, $CDCl_3$) δ 7.80 (1H, dd, *J* = 8.0, 1.4 Hz, Ar**H**), 7.63 (1H, dd, *J* = 8.0, 1.2 Hz, Ar**H**), 7.41–7.37 (2H, m, Ar**H**), 7.36–

7.28 (3H, m, Ar**H**), 7.23 (1H, ddd, *J* = 8.6, 7.3, 1.4 Hz, Ar**H**), 7.10 (1H, app d, *J* = 1.8 Hz, C=C**H**), 4.84 (1H, d, *J* = 1.3 Hz, O**H**), 3.34 (1H, dd, *J* = 16.5, 1.1 Hz, C**H**$_a$H$_b$), 2.91 (1H, dd, *J* = 16.5, 2.7 Hz, CH$_a$**H**$_b$), 1.89 (3H, s, C**H**$_3$C=O), 1.41 (3H, d, *J* = 1.4 Hz, C**H**$_3$COH), 1.37 (3H, s, C**H**$_3$CC=O); ^{13}C NMR (101 MHz, $CDCl_3$) δ 217.2 (C), 146.0 (C), 135.7 (C), 133.6 (C), 132.9 (C), 132.6 (C), 130.5 (2 × CH), 128.9 (CH), 128.7 (2 × CH), 127.1 (CH), 125.4 (CH), 124.4 (CH), 123.3 (CH), 75.0 (C), 54.8 (C), 36.9 (CH_2), 26.8 (CH_3), 26.0 (CH_3), 18.9 (CH_3); HRMS (ESI) Exact mass calculated for $C_{21}ClH_{21}NaO_2^+$ $[M+Na]^+$: 363.1122, found: 363.1114; Enantiomeric excess was determined by HPLC with a Chiralpak AS-H column (80:20 isohexane:*i*-PrOH, 1.0 mL/min, 280 nm, 23 °C): t_r (minor) = 5.3 min, t_r (major) = 7.3 min, 97% ee.

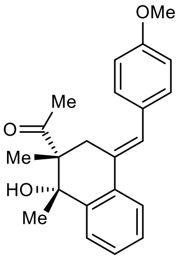

1-{(1*S*,2*R*)-1-Hydroxy-4-[(*E*)-4-methoxybenzylidene]-1,2-dimethyl-1,2,3,4-tetrahydronaphthalen-2-yl}ethan-1-one (2h). The title compound was prepared according to General Procedure A using alkynyl 1,3-diketone **1h** (77.4 mg, 0.30 mmol) and phenylboronic acid (73.2 mg, 0.60 mmol), and purified by column chromatography (10% EtOAc/pentane) to give a yellow oil (48.7 mg, 48%). R_f = 0.15 (10% EtOAc/pentane); $[\alpha]_D^{20.2}$ –204 (*c* 1.00, $CHCl_3$); IR (ATR) 3454 (OH), 2971, 2934, 1686 (C=O), 1604, 1509, 1175, 1073, 826, 701 cm^{-1}; ^{1}H NMR (400 MHz, $CDCl_3$) δ 7.79 (1H, dd, *J* = 7.8, 1.4 Hz, Ar**H**), 7.62 (1H, dd, *J* = 8.0, 1.3 Hz, Ar**H**), 7.36–7.28 (3H, m, Ar**H**), 7.24–7.19 (1H, m, Ar**H**), 7.10 (1H, app d, *J* = 2.5 Hz, C=C**H**), 6.99–6.93 (2H, m, Ar**H**), 4.88 (1H, d, *J* = 1.4 Hz, O**H**), 3.87 (3H, s, OC**H**$_3$), 3.41 (1H, dd, *J* = 16.5, 1.1 Hz, C**H**$_a$H$_b$), 2.92 (1H, dd, *J* = 16.5, 2.6 Hz, CH$_a$**H**$_b$), 1.90 (3H, s, C**H**$_3$C=O), 1.41 (3H, d, *J* = 1.3 Hz, C**H**$_3$COH), 1.37 (3H, s, C**H**$_3$CC=O); ^{13}C NMR (101 MHz, $CDCl_3$) δ 217.6 (C), 158.6 (C), 145.6 (C), 133.2 (C), 131.3 (C), 130.6 (2 × CH), 129.9 (C), 128.3 (CH), 127.0 (CH), 125.4 (CH), 125.2 (CH), 123.1 (CH), 113.9 (2 × CH), 75.1 (C), 55.3 (CH_3), 54.6 (C), 37.0 (CH_2), 26.7 (CH_3), 26.1 (CH_3), 18.9 (CH_3); HRMS (ESI) Exact mass calculated for $C_{22}H_{24}NaO_3^+$ $[M+Na]^+$: 359.1618, found: 359.1617; Enantiomeric excess was determined by HPLC with a Chiralpak AS-H column (80:20 isohexane:*i*-PrOH, 1.0 mL/min, 280 nm, 23 °C): t_r (minor) = 6.3 min, t_r (major) = 8.3 min, 88% ee.

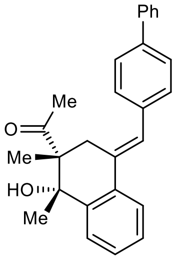

1-{(1*S*,2*R*,*E*)-4-[(1,1'-Biphenyl)-4-ylmethylene]-1-hydroxy-1,2-dimethyl-1,2,3,4-tetrahydronaphthalen-2-yl}ethanone (2i). The title compound was prepared according to General Procedure A using alkynyl 1,3-diketone **1i** (91.2 mg, 0.30 mmol) and phenylboronic acid (73.2 mg, 0.60 mmol), and purified by column chromatography (5% to 10% EtOAc/pentane) to give a yellow solid (73.8 mg, 64%). R_f = 0.14 (10% EtOAc/pentane); m.p. (Et_2O) 151–152 ˚C; IR (ATR) 3463 (OH), 1686 (C=O), 1454, 1356, 1227, 1073, 930, 835, 765, 695 cm^{-1}; $[\alpha]_D^{20.6}$ –228 (c 1.00, $CHCl_3$); ^{1}H NMR (400 MHz, $CDCl_3$)

δ 7.81 (1H, dd, *J* = 7.8, 1.2 Hz, Ar**H**), 7.70–7.60 (5H, m, Ar**H**), 7.49–7.45 (4H, m, Ar**H**), 7.42–7.30 (2H, m, Ar**H**), 7.26–7.21 (1H, m, Ar**H**), 7.19 (1H, app d, *J* = 1.6 Hz, C=C**H**), 4.89 (1H, s, O**H**), 3.48 (1H, dd, *J* = 16.6, 0.9 Hz, C**H**$_a$H$_b$), 2.99 (1H, dd, *J* = 16.6, 2.6 Hz, CH$_a$**H**$_b$), 1.93 (3H, s, C**H**$_3$C=O), 1.42 (3H, s, C**H**$_3$), 1.39 (3H, s, C**H**$_3$); ^{13}C NMR (101 MHz CDCl$_3$) δ 217.6 (C), 145.9 (C), 140.4 (C), 139.8 (C), 136.3 (C), 133.04 (C), 132.98 (C), 129.8 (CH), 128.9 (2 × CH), 128.6 (2 × CH), 127.5 (2 × CH), 127.12 (2 × CH), 127.05 (CH), 126.9 (CH), 125.4 (CH), 125.3 (CH), 123.3 (CH), 75.1 (C), 54.7 (C), 37.1 (CH$_2$), 26.7 (CH$_3$), 26.2 (CH$_3$), 18.9 (CH$_3$); HRMS (ESI) Exact mass calculated for [$C_{27}H_{26}NaO_2$]$^+$ [M+Na]$^+$: 405.1825, found 405.1822; Enantiomeric excess was determined by HPLC with Chiralpak AS-H column (80:20 isohexane:*i*-PrOH, 0.8 mL/min, 280 nm, 25 °C): t$_r$ (minor) = 6.4 min, t$_r$ (major) = 8.3 min; 85% ee.

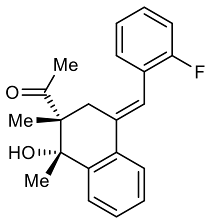

1-{(1*S*,2*R*)-4-[(*E*)-2-Fluorobenzylidene]-1-hydroxy-1,2-dimethyl-1,2,3,4-tetrahydronaphthalen-2-yl}ethan-1-one (2j). The title compound was prepared according to General Procedure A using alkynyl 1,3-diketone **1j** (73.8 mg, 0.30 mmol) and phenylboronic acid (73.2 mg, 0.60 mmol), and purified by column chromatography (10% EtOAc/pentane) to give a yellow solid (83.8 mg, 86%). R$_f$ = 0.15 (10% EtOAc/pentane); m.p. 126–128 °C (Et$_2$O); $[\alpha]_D^{20.3}$ –232 (*c* 1.00, CHCl$_3$); IR (ATR) 3472 (OH), 2993, 2971, 2875, 1685 (C=O), 1485, 1352, 1229, 763, 694 cm^{-1}; ^{1}H NMR (400 MHz, CDCl$_3$) δ 7.81 (1H, dd, *J* = 7.9, 1.4 Hz, Ar**H**), 7.67 (1H, dd, *J* = 8.0, 1.2 Hz, Ar**H**), 7.39–7.28 (3H, m, Ar**H**), 7.26–7.13 (3H, m, Ar**H**), 7.12 (1H, app d, *J* = 1.8 Hz, C=C**H**), 4.90 (1H, d, *J* = 1.5 Hz, O**H**), 3.18 (1H, dd, *J* = 16.6, 1.1 Hz, C**H**$_a$H$_b$), 2.88 (1H, dd, *J* = 16.6, 2.7 Hz, CH$_a$**H**$_b$), 1.92 (3H, s, C**H**$_3$C=O), 1.42 (3H, d, *J* = 1.0 Hz, C**H**$_3$COH), 1.35 (3H, s, C**H**$_3$CC=O); ^{13}C NMR (101 MHz, CDCl$_3$) δ 217.4 (C), 160.2 (C, J_{CF} = 247.9 Hz), 146.1 (C), 135.1 (C), 132.3 (C), 130.7 (CH), 129.1 (CH, J_{CF} = 8.3 Hz), 128.9 (CH), 127.0 (CH), 125.5 (CH), 125.0 (C, J_{CF} = 14.6 Hz), 124.0 (CH, J_{CF} = 3.6 Hz), 123.5 (CH), 118.2 (CH), 115.8 (CH, J_{CF} = 22.0 Hz), 75.1 (C), 54.8 (C), 36.8 (CH$_2$), 26.9 (CH$_3$), 25.8 (CH$_3$), 18.7 (CH$_3$); ^{19}F NMR (376 MHz, CDCl$_3$) δ –114.1; HRMS (ESI) Exact mass calculated for $C_{21}H_{21}FNaO_2^+$ [M+Na]$^+$: 347.1418, found: 347.1413; Enantiomeric excess was determined by HPLC with a Chiralpak AS-H column (80:20 isohexane:*i*-PrOH, 1.0 mL/min, 280 nm, 25 °C): t$_r$ (minor) = 5.3 min, t$_r$ (major) = 6.2 min; 97% ee.

Slow diffusion of pentane into a solution of **2j** in CH_2Cl_2 gave crystals that were suitable for X-ray crystallography:

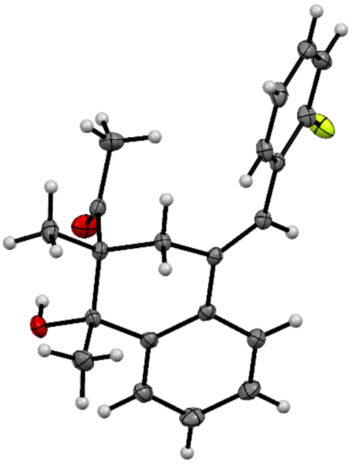

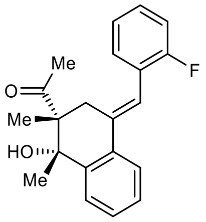

2j
CCDC 1959877
Flack parameter = 0.028(19)

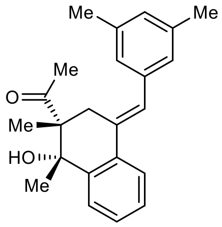

1-{(1*S*,2*R*)-4-[(*E*)-3,5-Dimethylbenzylidene]-1-hydroxy-1,2-dimethyl-1,2,3,4-tetrahydronaphthalen-2-yl}ethan-1-one (2k). The title compound was prepared according to General Procedure A using alkynyl 1,3-diketone **1k** (76.8 mg, 0.30 mmol) and phenylboronic acid (73.2 mg, 0.60 mmol), and purified by column chromatography (10% EtOAc/pentane) to give a yellow oil (75.2 mg, 75%). R_f = 0.14 (10% EtOAc/pentane); $[\alpha]_D^{20.4}$ –196 (*c* 1.00, $CHCl_3$); IR (ATR) 3461 (OH), 2977, 2918, 2865, 1687 (C=O), 1596, 1353, 1073, 841, 683 cm^{-1}; ^1H NMR (400 MHz, $CDCl_3$) δ 7.80 (1H, dd, *J* = 7.8, 1.4 Hz, Ar**H**), 7.63 (1H, dd, *J* = 8.0, 1.2 Hz, Ar**H**), 7.32 (1H, td, *J* = 7.5, 1.3 Hz, Ar**H**), 7.22 (1H, ddd, *J* = 8.5, 7.3, 1.5 Hz, Ar**H**), 7.11 (1H, app d, *J* = 2.5 Hz, C=C**H**), 6.98 (2H, s, Ar**H**), 6.95 (1H, s, Ar**H**), 4.89 (1H, d, *J* = 1.3 Hz, O**H**), 3.41 (1H, dd, *J* = 16.5, 1.1 Hz, C$\mathbf{H}_a$$H_b$), 2.93 (1H, dd, *J* = 16.5, 2.6 Hz, C$H_a$$\mathbf{H}_b$), 2.38 (6H, s, 2 × ArC$\mathbf{H}_3$), 1.92 (3H, s, C$\mathbf{H}_3$C=O), 1.42 (3H, d, *J* = 1.3 Hz, C$\mathbf{H}_3$COH), 1.37 (3H, s C$\mathbf{H}_3$CC=O); ^{13}C NMR (101 MHz, $CDCl_3$) δ 217.7 (C), 145.8 (C), 137.9 (2 × C), 137.2 (C), 133.1 (C)¸132.4 (C), 128.9 (CH), 128.4 (CH), 127.1 (2 × CH), 127.0 (CH), 126.0 (CH), 125.3 (CH), 123.2 (CH), 75.1 (C), 54.7 (C), 36.9 (CH_2), 26.7 (CH_3), 26.2 (CH_3), 21.4 (CH_3), 18.9 (CH_3); HRMS (ESI) Exact mass calculated for $C_{23}H_{26}NaO_2^+$ $[M+Na]^+$: 357.1825, found: 357.1821; Enantiomeric excess was determined by HPLC with a Chiralpak AS-H column (80:20 isohexane:*i*-PrOH, 1.0 mL/min, 280 nm, 23 °C): t_r (minor) = 4.2 min, t_r (major) = 4.7 min, 99% ee.

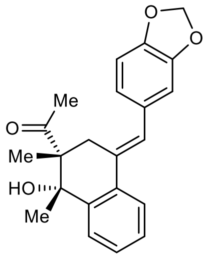

1-[(1*S*,2*R*,*E*)-4-(Benzo[*d*][1,3]dioxol-5-ylmethylene)-1-hydroxy-1,2-dimethyl-1,2,3,4-tetrahydronaphthalen-2-yl]ethanone (2l). The title compound was prepared according to General Procedure A using alkynyl 1,3-diketone **1l** (81.6 mg, 0.30 mmol) and phenylboronic acid (73.2 mg, 0.60 mmol), and purified by column chromatography (5% to 10% EtOAc/pentane) to give a yellow oil (61.0 mg, 58%). R_f = 0.10 (10% EtOAc/pentane); $[\alpha]_D^{20.3}$ –220 (*c* 1.00, $CHCl_3$); IR (ATR) 3455 (OH), 1687 (C=O), 1488, 1354, 1249, 1246, 1102, 1036, 922, 783, 730 cm^{-1}; ^1H NMR (400 MHz, $CDCl_3$) δ 7.78 (1H,

dd, *J* = 7.8, 1.3 Hz, Ar**H**), 7.59 (1H, d, *J* = 7.4 Hz, Ar**H**), 7.34–7.27 (1H, m, Ar**H**), 7.24–7.16 (1H, m, Ar**H**), 7.05 (1H, app d, *J* = 1.7 Hz, C=C**H**), 6.88–6.82 (3H, m, Ar**H**), 6.05–5.98 (2H, m, OC**H**$_2$O), 4.86 (1H, d, *J* = 1.1 Hz, O**H**), 3.39 (1H, d, *J* = 16.4 Hz, CC**H**$_a$H$_b$), 2.89 (1H, dd, *J* =16.4, 2.5 Hz, CCH$_a$**H**$_b$), 1.91 (3H, s, C**H**$_3$C=O), 1.39 (3H, d, *J* = 1.0 Hz, C**H**$_3$COH), 1.37 (3H, s, C**H**$_3$CC=O); ^{13}C NMR (101 MHz CDCl$_3$) δ 217.5 (C), 147.7 (C), 146.6 (C), 145.8 (C), 133.0 (C), 131.9 (C), 131.4 (C), 128.4 (CH), 127.0 (CH), 125.5 (CH), 125.3 (CH), 123.4 (CH), 123.1 (CH), 109.3 (CH), 108.5 (CH), 101.2 (CH$_2$), 75.0 (C), 54.7 (C), 37.0 (CH$_2$), 26.7 (CH$_3$), 26.1 (CH$_3$), 18.9 (CH$_3$); HRMS (ESI) Exact mass calculated for [$C_{22}H_{22}NaO_4$]$^+$ [M+Na]$^+$: 373.1410, found 373.1411; Enantiomeric excess was determined by HPLC with a Chiralpak AS-H column (80:20 isohexane:*i*-PrOH, 1.0 mL/min, 230 nm, 25 °C): t$_r$ (minor) = 8.5 min, t$_r$ (major) = 11.2 min, 90% ee.

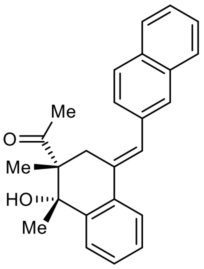

1-[(1*S*,2*R*,*E*)-1-Hydroxy-1,2-dimethyl-4-(naphthalen-2-ylmethylene)-1,2,3,4-tetrahydronaphthalen-2-yl]ethanone (2m). The title compound was prepared according to General Procedure A using alkynyl 1,3-diketone **1m** (83.4 mg, 0.30 mmol) and phenylboronic acid (73.2 mg, 0.60 mmol), and purified by column chromatography (5% to 10% EtOAc/pentane) to give a yellow solid (70.3 mg, 66%). R$_f$ = 0.11 (10% EtOAc/pentane); m.p. 110–111 °C (Et$_2$O); $[\alpha]_D^{20.4}$ –248 (*c* 1.00, CHCl$_3$); IR (ATR) 3449 (OH), 1686 (C=O), 1454, 1353, 1168, 1073, 949, 866, 789, 665 cm^{-1}; ^{1}H NMR (400 MHz, CDCl$_3$) δ 7.90–7.86 (3H, m, Ar**H**), 7.84–7.82 (2H, m, Ar**H**), 7.71 (1H, d, *J* = 7.6 Hz, Ar**H**), 7.57–7.46 (3H, m, Ar**H**), 7.39–7.30 (2H, m, Ar**H** and C=C**H**), 7.28–7.21 (1H, m, ArC**H**), 4.89 (1H, d, *J* = 1.0 Hz, O**H**), 3.50 (1H, d, *J* = 16.5 Hz, C**H**$_a$H$_b$), 3.04 (1 H, dd, *J* = 16.5, 2.5 Hz, CH$_a$**H**$_b$), 1.88 (3H, s, C**H**$_3$C=O), 1.45 (3H, s, C**H**$_3$), 1.36 (3H, s, C**H**$_3$); ^{13}C NMR (101 MHz CDCl$_3$) δ 217.4 (C), 146.0 (C), 134.8 (C), 133.3 (C), 132.8 (C), 132.3 (2 × C), 128.7 (C), 128.13 (CH), 128.08 (CH), 128.0 (CH), 127.7 (CH), 127.4 (CH), 127.0 (CH), 126.4 (CH), 126.2 (CH), 125.7 (CH), 125.4 (CH), 123.3 (CH), 75.1 (C), 54.9 (C), 37.0 (CH$_2$), 26.9 (CH$_3$), 26.0 (CH$_3$), 18.8 (CH$_3$); HRMS (ESI) Exact mass calculated for [$C_{25}H_{24}NaO_2$]$^+$ [M+Na]$^+$: 379.1669, found 379.1664; Enantiomeric excess was determined by HPLC with a Chiralpak AS-H column (80:20 isohexane:*i*-PrOH, 1.0 mL/min, 280 nm, 25 °C): t$_r$ (minor) = 5.9 min, t$_r$ (major) = 6.9 min, 99% ee.

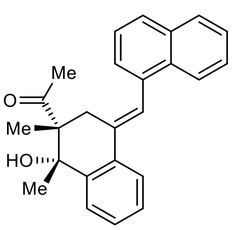

1-[(1S,2*R*,*E*)-1-Hydroxy-1,2-dimethyl-4-(naphthalen-1-ylmethylene)-1,2,3,4-tetrahydronaphthalen-2-yl]ethan-1-one (2n). The title compound was prepared according to General Procedure A using alkynyl 1,3-diketone **1n** (83.4 mg, 0.30 mmol) and phenylboronic acid (73.2 mg, 0.60 mmol), and purified by column chromatography (5% Et$_2$O/CHCl$_3$) to give a yellow solid (42.7 mg, 40%). R$_f$ = 0.35 (5% Et$_2$O/CHCl$_3$); m.p. 72-74 °C (Et$_2$O); $[\alpha]_D^{19.9}$ –224 (c 1.00, CHCl$_3$); IR (ATR)

3453 (OH), 3057, 2979, 2937, 1687 (C=O), 1353, 1106, 788, 763, 515 cm^{-1}; ^{1}H NMR (400 MHz, $CDCl_3$) δ 7.96 (1H, dd, *J* = 8.4, 1.3 Hz, Ar**H**), 7.92 (1H, dd, *J* = 7.8, 1.6 Hz, Ar**H**), 7.88-7.83 (2H, m, Ar**H**), 7.80 (1H, dd, *J* = 8.1, 1.3 Hz, Ar**H**), 7.61 (1H, t, *J* = 1.6 Hz, C=C**H**), 7.56-7.47 (3H, m, Ar**H**), 7.44-7.35 (2H, m, Ar**H**), 7.32-7.28 (1H, m, Ar**H**), 4.85 (1H, d, *J* = 1.4 Hz, O**H**), 3.14 (1H, dd, *J* = 16.6, 1.0 Hz, C**H**$_a$H$_b$), 2.83 (1H, dd, *J* = 16.6, 2.6 Hz, CH$_a$**H**$_b$), 1.80 (3H, s, C**H**$_3$C=O), 1.44 (3H, d, *J* = 1.3 Hz, C**H**$_3$), 1.26 (3H, s, C**H**$_3$); ^{13}C NMR (101 MHz, $CDCl_3$) δ 217.3 (C), 146.0 (C), 134.5 (C), 134.3 (C), 133.7 (C), 132.4 (C), 132.0 (C), 128.8 (CH), 128.6 (CH), 127.9 (CH), 127.1 (CH), 126.7 (CH), 126.3 (CH), 126.1 (CH), 125.6 (CH), 125.4 (CH), 124.7 (CH), 123.5 (CH), 123.3 (CH), 75.1 (C), 55.0 (C), 37.0 (CH_2), 27.1 (CH_3), 25.9 (CH_3), 18.7 (CH_3); HRMS (ESI) Exact mass calculated for $C_{25}H_{24}NaO_2^+$ [M+Na]$^+$: 379.1669, found: 379.1661; Enantiomeric excess was determined by HPLC with a Chiralpak AS-H column (80:20 isohexane:*i*-PrOH, 0.5 mL/min, 210 nm, 23 °C): t_r (minor) = 11.4 min, t_r (major) = 12.7 min, 92% ee.

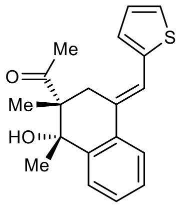

1-{(1S,2*R*,*E*)-1-Hydroxy-1,2-dimethyl-4-(thiophen-2-ylmethylene)-1,2,3,4-tetrahydronaphthalen-2-yl}ethan-1-one (2o). The title compound was prepared according to General Procedure A using alkynyl 1,3-diketone **1o** (70.2 mg, 0.30 mmol) and phenylboronic acid (73.2 mg, 0.60 mmol), and purified by column chromatography (10% EtOAc/pentane) to give a yellow oil (61.5 mg, 66%). R$_f$ = 0.11 (10% EtOAc/pentane); $[\alpha]_D^{20.3}$ +12 (*c* 1.00, $CHCl_3$); IR (ATR) 3461 (OH), 3064, 2974, 2928, 1684 (C=O), 1352, 1073, 758, 693 cm^{-1}; ^{1}H NMR (400 MHz, $CDCl_3$) δ 7.77 (1H, dd, *J* = 7.8, 1.4 Hz, Ar**H**), 7.62 (1H, dd, *J* = 8.0, 1.3 Hz, Ar**H**), 7.41 (1H, dd, *J* = 5.2, 1.0 Hz, Ar**H**), 7.34–7.28 (2H, m, Ar**H** and C=C**H**), 7.25–7.19 (2H, m, Ar**H**), 7.14 (1H, dd, *J* = 5.1, 3.6 Hz, Ar**H**), 4.92 (1H, s, O**H**), 3.55 (1H, dd, *J* = 17.9, 1.7 Hz, C**H**$_a$H$_b$), 2.91 (1H, dd, *J* = 17.9, 2.6 Hz, CH$_a$**H**$_b$), 2.01 (3H, s, C**H**$_3$C=O), 1.47 (3H, s, C**H**$_3$COH), 1.35 (3H, s, C**H**$_3$CC=O); ^{13}C NMR (101 MHz, $CDCl_3$) δ 218.4 (C), 145.8 (C), 140.7 (C), 133.2 (C), 130.3 (C), 128.6 (CH), 128.3 (CH), 127.4 (CH), 127.2 (CH), 126.2 (CH), 124.6 (CH), 123.2 (CH), 119.1 (CH), 75.0 (C), 53.7 (C), 37.5 (CH_2), 27.2 (CH_3), 25.3 (CH_3), 19.3 (CH_3); HRMS (ESI) Exact mass calculated for $C_{19}H_{20}NaO_2S^+$ [M+Na]$^+$: 335.1076, found: 335.1072; Enantiomeric excess was determined by HPLC with a Chiralpak AS-H column (80:20 isohexane:*i*-PrOH, 1.0 mL/min, 280 nm, 23 °C): t_r (minor) = 5.9 min, t_r (major) = 7.1 min, 97% ee.

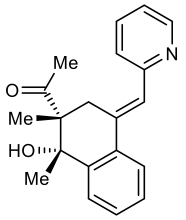

1-[(1*S*,2*R*,*E*)-1-Hydroxy-1,2-dimethyl-4-(pyridin-2-ylmethylene)-1,2,3,4-tetrahydronaphthalen-2-yl]ethanone (2p). The title compound was prepared according to General Procedure A using alkynyl 1,3-diketone **1p** (68.7 mg, 0.30 mmol) and phenylboronic acid (73.2 mg, 0.60 mmol), and purified by column chromatography (5% to 10% EtOAc/pentane) to give a yellow oil (62.1 mg, 67%).

R_f = 0.14 (10% EtOAc/pentane); $[\alpha]_D^{20.4}$ –12 (*c* 1.00, $CHCl_3$); IR (ATR) 3443 (OH), 1686 (C=O), 1456, 1377, 1149, 1073, 929, 865, 756, 674 cm^{-1}; ^1H NMR (400 MHz, $CDCl_3$) δ 8.69 (1H, dd, *J* = 4.8, 1.0 Hz, Ar**H**), 7.81 (1H, dd, *J* = 7.8, 1.2 Hz, Ar**H**), 7.71–7.67 (2H, m, Ar**H**), 7.37–7.29 (2H, m, Ar**H**), 7.25–7.20 (1H, m, Ar**H**), 7.14 (1H, ddd, *J* = 7.5, 4.8, 1.0 Hz, Ar**H**), 7.07 (1H, br s, C=C**H**), 4.95 (1H, d, *J* = 1.2 Hz, O**H**), 4.31 (1H, dd, *J* = 18.3, 1.2 Hz, C**H**$_a$H$_b$), 3.10 (1H, dd, *J* = 18.3, 2.6 Hz, CH$_a$**H**$_b$), 1.98 (3H, s, C**H**$_3$C=O), 1.43 (3H, s, C**H**$_3$CC=O), 1.39 (3H, d, *J* = 1.2 Hz, C**H**$_3$COH); ^{13}C NMR (101 MHz $CDCl_3$) δ 218.6 (C), 156.7 (C), 149.2 (C), 146.9 (C), 137.5 (C), 136.2 (CH), 133.2 (CH), 129.1 (CH), 127.0 (CH), 125.5 (CH), 125.1 (CH), 123.8 (CH), 123.5 (CH), 121.1 (CH), 75.0 (C), 54.4 (C), 36.9 (CH_2), 26.8 (CH_3), 26.1 (CH_3), 19.1 (CH_3); HRMS (ESI) Exact mass calculated for $[C_{20}H_{21}NNaO_2]^+$ $[M+Na]^+$: 330.1464, found 330.1459; Enantiomeric excess was determined by HPLC with a Chiralpak AS-H column (80:20 isohexane:*i*-PrOH, 0.8 mL/min, 280 nm, 25 °C): t_r (minor) = 8.2 min, t_r (major) = 11.3 min, 96% ee.

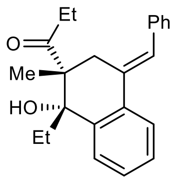

1-{(1*S*,2*R*)-4-[(*E*)-Benzylidene]-1-ethyl-1-hydroxy-2-methyl-1,2,3,4-tetrahydronaphthalen-2-yl}propan-1-one (2q). The title compound was prepared according to General Procedure A using alkynyl 1,3-diketone **1q** (76.8 mg, 0.30 mmol) and phenylboronic acid (73.2 mg, 0.60 mmol), and purified by column chromatography (5% to 10% EtOAc/pentane) to give a yellow solid (62.9 mg, 63%). R_f = 0.14 (10% EtOAc/pentane); m.p. 107–108 °C (Et_2O); $[\alpha]_D^{20.3}$ +60 (*c* 1.00, $CHCl_3$); IR (ATR) 3409 (OH), 1679 (C=O), 1460, 1372, 1216, 1141, 1098, 997, 870, 763 cm^{-1}; ^1H NMR (400 MHz, $CDCl_3$) δ 7.67 (1H, dd, *J* = 7.7, 1.4 Hz, Ar**H**), 7.63 (1H, dd, *J* = 7.8, 1.2 Hz, Ar**H**), 7.48–7.40 (4H, m, Ar**H**), 7.33–7.26 (2H, m, Ar**H**), 7.22 (1H, ddd, *J* = 6.8, 6.2, 2.4 Hz, Ar**H**), 7.06 (1H, app d, *J* = 2.1 Hz, C=C**H**), 3.49 (1H, dd, *J* =18.2, 1.8, C**H**$_a$H$_b$C=), 2.99 (1H, dd, *J* =18.2, 2.6 Hz, C**H**$_a$H$_b$C=), 2.48 (1H, dq, *J* =18.6, 7.1 Hz, CH$_3$C**H**$_2$C=O), 2.14 (1H, dq, *J* =18.6, 7.1 Hz, CH$_3$C**H**$_2$C=O), 1.88 (1H, dq, *J* =14.6, 7.3 Hz, CH$_3$C**H**$_2$COH), 1.57–1.46 (1H, m, CH$_3$C**H**$_2$COH), 1.35 (3H, s, C**H**$_3$CC=O), 0.77 (3H, t, *J* = 7.3 Hz, C**H**$_3$CH$_2$COH), 0.72 (3H, t, *J* = 7.1 Hz, CH$_3$C**H**$_2$C=O); ^{13}C NMR (101 MHz $CDCl_3$) δ 221.1 (C), 143.3 (C), 137.5 (C), 134.2 (C), 133.1 (C), 129.3 (2 × CH), 128.5 (2 × CH), 127.4 (CH), 127.04 (CH), 127.01 (CH), 126.3 (CH), 126.0 (CH), 123.9 (CH), 76.6 (C), 54.2 (C), 36.1 (CH_2), 32.0 (CH_2), 27.8 (CH_2), 19.4 (CH_3), 7.4 (CH_3), 7.1 (CH_3); HRMS (ESI) Exact mass calculated for $[C_{23}H_{26}NaO_2]^+$ $[M+Na]^+$: 357.1825, found 357.1815; Enantiomeric excess was determined by HPLC with a Chiralpak AD-H column (97:03 isohexane:*i*-PrOH, 1.0 mL/min, 280 nm, 25 °C): t_r (minor) = 6.2 min, t_r (major) = 7.6 min, 96% ee.

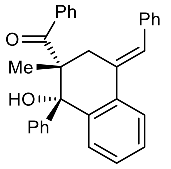

{(1*S*,2*R*)-4-[(*E*)-Benzylidene]-1-hydroxy-2-methyl-1-phenyl-1,2,3,4-tetrahydronaphthalen-2-yl}(phenyl)methanone (2r).[11] The title compound was prepared according to General Procedure A using alkynyl 1,3-diketone **1r** (105.6 mg, 0.30 mmol) and phenylboronic acid (73.2 mg, 0.60 mmol), and purified by column chromatography (5% to 10% EtOAc/pentane) to give a white solid (62.2 mg, 48%) whose spectroscopic data were consistent with those reported previously.[11] R_f = 0.14 (10% EtOAc/pentane); $[\alpha]_D^{20.5}$ –160 (*c* 0.50, $CHCl_3$); IR (ATR) 3423 (OH), 1655 (C=O), 1445, 1368, 1179, 1041, 906, 758 cm^{-1}; m.p. 149–150 °C (Et_2O); ^1H NMR (400 MHz, $CDCl_3$) δ 7.77–7.65 (2H, m, Ar**H**), 7.37–7.28 (6H, m, Ar**H**), 7.25 (1H, s, C=C**H**), 7.24–7.14 (6H, m, Ar**H**), 7.04 (3H, m, Ar**H**), 6.91 (2H, d, *J* =6.8 Hz, Ar**H**), 5.88 (1H, s, O**H**), 3.48 (1H, d, *J* =16.7 Hz, C$\mathbf{H}_a$$H_b$), 2.74 (1H, dd, *J* = 16.7, 2.6 Hz, C$H_a$$\mathbf{H}_b$), 1.46 (3H, s, C$\mathbf{H}_3$); ^{13}C NMR (101 MHz $CDCl_3$) δ 213.5 (C), 144.5 (C), 142.7 (C), 138.8 (C), 137.0 (C), 134.4 (C), 132.7 (C), 130.9 (CH), 129.2 (2 × CH), 129.1 (2 × CH), 128.8 (CH), 128.0 (2 × CH), 127.6 (2 × CH), 127.4 (CH), 127.2 (3 × CH), 127.1 (CH), 127.0 (2 × CH), 126.7 (CH), 125.8 (CH), 122.7 (CH), 80.5 (C), 54.9 (C), 36.4 (CH_2), 20.1 (CH_3); HRMS (ESI) Exact mass calculated for $[C_{31}H_{26}NaO_2]^+$ $[M+Na]^+$: 453.1825, found 453.1814; Enantiomeric excess was determined by HPLC with a Chiralpak AD-H column (97:3 isohexane:*i*-PrOH, 1.0 mL/min, 280 nm, 25 °C): t_r (major) = 9.1 min, t_r (minor) = 19.8 min, 56% ee.

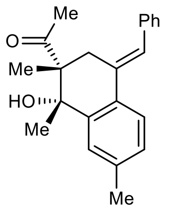

1-[(1*S*,2*R*,*E*)-4-Benzylidene-1-hydroxy-1,2-dimethyl-1,2,3,4-tetrahydronaphthalen-2-yl]ethanone (2s). The title compound was prepared according to General Procedure A using alkynyl 1,3-diketone **1a** (68.5 mg, 0.30 mmol) and phenylboronic acid (73.2 mg, 0.60 mmol), and purified by column chromatography (5% to 10% EtOAc/pentane) to give a yellow solid (57 mg, 59%). R_f = 0.12 (10% EtOAc/pentane); m.p. 126–127 °C (Et_2O); $[\alpha]_D^{25.0}$ –252 (*c* 1.00, $CHCl_3$); IR (ATR) 3465 (OH), 1683 (C=O), 1495, 1377, 1353, 1351, 1077, 929, 767, 696, 581 cm^{-1}; ^1H NMR (400 MHz, $CDCl_3$) δ 7.60 (1H, d, *J* = 0.8 Hz, Ar**H**), 7.54 (1H, t, *J* = 6.0 Hz, Ar**H**), 7.44–7.33 (4H, m, Ar**H**), 7.28 (1H, ddd, *J* = 7.3, 3.2, 1.8 Hz, Ar**H**), 7.11 (1H, app d, *J* = 1.9 Hz, C=C**H**), 7.03 (1H, ddd, *J* = 8.1, 1.9, 0.6 Hz, Ar**H**), 4.86 (1H, d, *J* = 1.2 Hz, O**H**), 3.38 (1H, dd, *J* = 16.6, 1.0 Hz, C$\mathbf{H}_a$$H_b$), 2.91 (1H, dd, *J* = 16.6, 2.6 Hz, C$H_a$$\mathbf{H}_b$), 2.37 (3H, s, ArC$\mathbf{H}_3$), 1.88 (3H, s, C$\mathbf{H}_3$C=O), 1.39 (3H, d, *J* = 1.2 Hz, C$\mathbf{H}_3$COH), 1.35 (3H, s, C$\mathbf{H}_3$CC=O); ^{13}C NMR (101 MHz $CDCl_3$) δ 217.7 (C), 145.8 (C), 138.5 (C), 137.4 (C), 132.8 (C), 130.1 (C), 129.3 (2 × CH), 128.5 (2 × CH), 128.0 (CH), 126.9 (CH), 125.8 (CH), 124.6 (CH), 123.3 (CH), 75.0 (C), 54.7 (C), 37.0 (CH_2), 26.7 (CH_3), 26.1 (CH_3), 21.4 (CH_3), 18.9 (CH_3); HRMS (ESI) Exact mass calculated for $[C_{22}H_{22}NaO_2]^+$ $[M+Na]^+$: 343.1669, found 343.1665; Enantiomeric excess

was determined by HPLC with a Chiralpak AS-H column (80:20 isohexane:*i*-PrOH, 0.8 mL/min, 280 nm, 25 °C): t_r (minor) = 5.9 min, t_r (major) = 7.1 min, 94% ee.

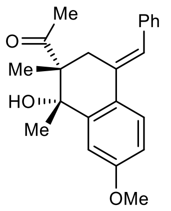

1-{(1*S*,2*R*)-4-[(*E*)-Benzylidene]-1-hydroxy-7-methoxy-1,2-dimethyl-1,2,3,4-tetrahydronaphthalen-2-yl-ethan-1-one (2t). The title compound was prepared according to General Procedure A using alkynyl 1,3-diketone **1a** (68.4 mg, 0.30 mmol) and 4-methoxyphenylboronic acid (91.8 mg, 0.60 mmol), and purified by column chromatography (10% EtOAc/pentane) to give a yellow oil (53.2 mg, 53%). R_f = 0.15 (10% EtOAc/pentane); $[\alpha]_D^{20.1}$ –200 (*c* 1.00, $CHCl_3$); IR (ATR) 3450 (OH), 2965, 2935, 1686 (C=O), 1600, 1354, 1068, 1030, 768, 697 cm^{-1}; ^1H NMR (400 MHz, $CDCl_3$) δ 7.60 (1H, d, *J* = 8.7 Hz, Ar**H**), 7.43–7.40 (2H, m, Ar**H**), 7.38–7.32 (3H, m, Ar**H**), 7.31–7.28 (1H, m, Ar**H**), 7.04 (1H, app d, *J* = 2.5 Hz, C=C**H**), 6.79 (1H, dd, *J* = 8.8, 2.8 Hz, Ar**H**), 4.96 (1H, d, *J* = 1.4 Hz, O**H**), 3.86 (3H, s, OC**H**$_3$), 3.38 (1H, dd, *J* = 16.7, 1.2 Hz, C**H**$_a$H$_b$), 2.92 (1H, dd, *J* = 16.7, 2.7 Hz, CH$_a$**H**$_b$), 1.90 (3H, s, C**H**$_3$C=O), 1.41 (3H, d, *J* = 1.4 Hz, C**H**$_3$COH), 1.36 (3H, s, C**H**$_3$CC=O); ^{13}C NMR (101 MHz, $CDCl_3$) δ 217.8 (C), 160.1 (C), 147.8 (C), 137.5 (C), 132.4 (C), 129.2 (2 × CH), 128.5 (2 × CH), 126.8 (CH), 125.6 (C), 124.9 (CH), 123.5 (CH), 114.1 (CH), 109.2 (CH), 75.2 (C), 55.3 (CH_3), 54.6 (C), 37.0 (CH_2), 26.6 (CH_3), 26.1 (CH_3), 18.9 (CH_3); HRMS (ESI) Exact mass calculated for $C_{22}H_{24}NaO_3^+$ $[M+Na]^+$: 359.1618, found: 359.1614; Enantiomeric excess was determined by HPLC with a Chiralpak AS-H column (80:20 isohexane:*i*-PrOH, 1.0 mL/min, 280 nm, 23 °C): t_r (minor) = 6.1 min, t_r (major) = 8.5 min, 96% ee.

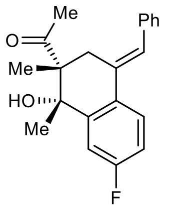

1-{(1*S*,2*R*)-4-[(*E*)-Benzylidene]-7-fluoro-1-hydroxy-1,2-dimethyl-1,2,3,4-tetrahydronaphthalen-2-yl}ethan-1-one (2u). The title compound was prepared according to General Procedure A using alkynyl 1,3-diketone **1a** (68.4 mg, 0.30 mmol) and 4-fluorophenylboronic acid (84.0 mg, 0.60 mmol), and purified by column chromatography (10% EtOAc/pentane) to give a yellow solid (72.3 mg, 74%). R_f = 0.11 (10% EtOAc/pentane); m.p. 129–131 °C (Et_2O); $[\alpha]_D^{20.1}$ –228 (*c* 1.00, $CHCl_3$); IR (ATR) 3475 (OH), 2973, 2919, 1686 (C=O), 1479, 1374, 1064, 769, 696 cm^{-1}; ^1H NMR (400 MHz, $CDCl_3$) δ 7.62 (1H, dd, *J* = 8.8, 5.5 Hz, Ar**H**), 7.50 (1H, dd, *J* = 10.0, 2.8 Hz, Ar**H**), 7.46–7.40 (2H, m, Ar**H**), 7.38–7.29 (3H, m, Ar**H**), 7.08 (1H, app d, *J* = 1.7 Hz, C=C**H**), 6.92 (1H, ddd, *J* = 8.8, 7.9, 2.8 Hz, Ar**H**), 4.91 (1H, d, *J* = 1.4 Hz, O**H**), 3.39 (1H, dd, *J* = 16.6, 1.1 Hz, C**H**$_a$H$_b$), 2.91 (1H, dd, *J* = 16.6, 2.7 Hz, CH$_a$**H**$_b$), 1.89 (3H, s, C**H**$_3$C=O), 1.40 (3H, d, *J* = 1.4 Hz, C**H**$_3$COH), 1.36 (3H, s, C**H**$_3$CC=O); ^{13}C NMR (101 MHz, $CDCl_3$) δ 217.3 (C), 163.3 (C, J_{CF} = 248.0 Hz), 148.6 (C), 137.1 (C), 131.9 (C), 129.2 (2 × CH), 129.0 (C), 128.6 (2 × CH), 127.2 (CH), 125.4 (CH), 125.3 (CH, J_{CF} = 7.9 Hz), 114.4 (CH, J_{CF} = 22.0 Hz), 112.1 (CH, J_{CF} = 22.7 Hz), 74.9 (C), 54.7 (CH), 36.8 (CH_2), 26.7 (CH_3), 25.9

(CH_3), 18.8 (CH_3); ^{19}F NMR (376 MHz, $CDCl_3$) δ –113.1; HRMS (ESI) Exact mass calculated for $C_{21}H_{21}FNaO_2^+$ [M+Na]$^+$: 347.1418, found: 347.1416; Enantiomeric excess was determined by HPLC with a Chiralpak AD-H column (90:10 isohexane:*i*-PrOH, 1.0 mL/min, 280 nm, 23 °C): t_r (minor) = 6.3 min, t_r (major) = 6.7 min, 93% ee.

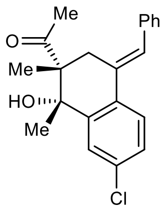

1-[(1*S*,2*R*,*E*)-4-Benzylidene-7-chloro-1-hydroxy-1,2-dimethyl-1,2,3,4-tetrahydronaphthalen-2-yl]ethanone (2v). The title compound was prepared according to General Procedure A using alkynyl 1,3-diketone **1a** (68.5 mg, 0.30 mmol) and phenylboronic acid (73.2 mg, 0.60 mmol), and purified by column chromatography (5% to 10% EtOAc/pentane) to give a yellow solid (53 mg, 52%). R_f = 0.16 (10% EtOAc/pentane); m.p. 109–110 °C (Et_2O); $[\alpha]_D^{24.9}$ –224 (*c* 1.00, $CHCl_3$); IR (ATR) 3471 (OH), 1684 (C=O), 1492, 1353, 1351, 1075, 931, 873, 759, 695 cm^{-1}; ^{1}H NMR (400 MHz, $CDCl_3$) δ 7.77 (1H, dd, *J* = 16.8, 4.0 Hz, Ar**H**), 7.55 (1H, t, *J* = 11.5 Hz, Ar**H**), 7.45–7.39 (2H, m, Ar**H**), 7.38–7.29 (3H, m, Ar**H**), 7.18 (1H, dd, *J* = 8.6, 2.3 Hz, Ar**H**), 7.12 (1H, app d, *J* = 2.1 Hz, C=C**H**), 4.88 (1H, d, *J* = 1.3 Hz, O**H**), 3.39 (1H, dd, *J* = 16.6, 0.8 Hz, C**H**$_a$H$_b$), 2.90 (1H, dd, *J* = 16.6, 2.6 Hz, C**H**$_a$H$_b$), 1.88 (3H, s, C**H**$_3$C=O), 1.39 (3H, d, *J* = 1.3 Hz, C**H**$_3$COH), 1.35 (3H, s, C**H**$_3$CC=O); ^{13}C NMR (101 MHz $CDCl_3$) δ 217.2 (C), 147.7 (C), 136.9 (C), 134.6 (C), 131.8 (C), 131.4 (C), 129.3 (2 × CH), 128.6 (2 × CH), 127.32 (CH), 127.26 (CH), 126.2 (CH), 125.6 (CH), 124.8 (CH), 74.9 (C), 54.6 (C), 36.7 (CH_2), 26.7 (CH_3), 25.9 (CH_3), 18.7 (CH_3); HRMS (ESI) Exact mass calculated for $[C_{21}H_{21}ClNaO_2]^+$ [M+Na]$^+$: 363.1122, found 363.1119; Enantiomeric excess was determined by HPLC with a Chiralpak AS-H column (93:7 isohexane:*i*-PrOH, 0.8 mL/min, 280 nm, 25 °C): t_r (minor) = 13.2 min, t_r (major) = 14.1 min, 88% ee.

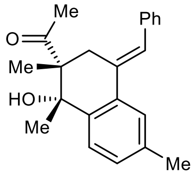

1-{(1*S*,2*R*)-4-[(*E*)-Benzylidene]-1-hydroxy-1,2,6-trimethyl-1,2,3,4-tetrahydronaphthalen-2-yl}ethan-1-one (2x). The title compound was prepared according to General Procedure A using alkynyl 1,3-diketone **1a** (68.4 mg, 0.30 mmol) and 3-methylphenylboronic acid (81.6 mg, 0.60 mmol), and purified by column chromatography (10% EtOAc/pentane) to give a yellow oil (70.8 mg, 74%). R_f = 0.14 (10% EtOAc/pentane); $[\alpha]_D^{20.1}$ –208 (*c* 1.00, $CHCl_3$); IR (ATR) 3471 (OH), 3021, 2979, 1681 (C=O), 1352, 1073, 768, 699 cm^{-1}; ^{1}H NMR (400 MHz, $CDCl_3$) δ 7.69 (1H, d, *J* = 8.0 Hz, Ar**H**), 7.46 (1H, s, Ar**H**), 7.44–7.41 (2H, m, Ar**H**), 7.39–7.35 (2H, m, Ar**H**), 7.34–7.28 (1H, m, Ar**H**), 7.19–7.12 (2H, m, C=C**H** and Ar**H**), 4.85 (1H, d, *J* = 1.3 Hz, O**H**), 3.40 (1H, dd, *J* = 16.6, 1.2 Hz, C**H**$_a$H$_b$), 2.92 (1H, dd, *J* = 16.5, 2.7 Hz, CH$_a$**H**$_b$), 2.36 (3H, s, ArC**H**$_3$), 1.89 (3H, s, C**H**$_3$C=O), 1.40 (1H, d, *J* = 1.4 Hz, C**H**$_3$COH), 1.35 (3H, s, C**H**$_3$CC=O); ^{13}C NMR (101 MHz, $CDCl_3$) δ 217.5 (C), 143.1 (C), 137.3 (C), 136.4 (C), 132.9 (C), 132.7 (C), 129.6 (CH), 129.3 (2 × CH), 128.5 (2 × CH), 127.0 (CH), 125.5

(CH), 125.3 (CH), 123.7 (CH), 75.0 (C), 54.8 (C)¸ 36.9 (CH_2), 26.8 (CH_3), 26.1 (CH_3), 21.2 (CH_3), 18.8 (CH_3); HRMS (ESI) Exact mass calculated for $C_{22}H_{24}NaO_2^+$ $[M+Na]^+$: 343.1669, found: 343.1663; Enantiomeric excess was determined by HPLC with a Chiralpak AS-H column (95:5 isohexane:*i*-PrOH, 1.0 mL/min, 280 nm, 23 °C): t_r (minor) = 7.1 min, t_r (major) = 7.8 min, 90% ee.

General Procedure B

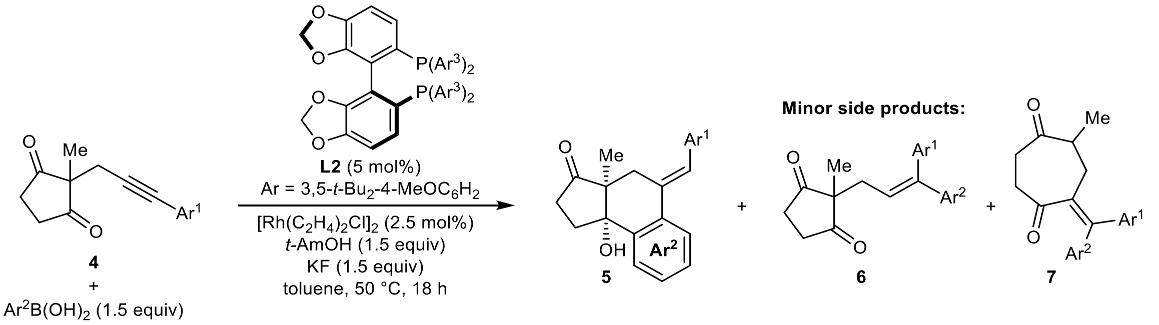

$[Rh(C_2H_4)_2Cl]_2$ (2.9 mg, 0.0075 mmol) and (*S*)-DTBM-SEGPHOS (**L2**, 17.7 mg, 0.015 mmol) were added to an oven-dried microwave vial, which was sealed with a septum and purged with argon for 30 min. Degassed toluene (2.5 mL) was added and the mixture was stirred at room temperature for 30 min under argon. In a separate oven-dried microwave vial the alkynyl 1,3-diketone (0.30 mmol), boronic acid (0.45 mmol), and KF (26.1 mg, 0.45 mmol) were added. A septum was fitted and the vial was purged with argon for 30 min. The catalyst solution was added to the vial containing the alkynyl 1,3-diketone using additional toluene (0.5 mL) as a rinse. *t*-Amyl alcohol (49 µL, 0.45 mmol) was added and the mixture was stirred at 50 °C for 18 h. The reaction was cooled to room temperature, diluted with H_2O and saturated aqueous NH_4Cl solution, and extracted with EtOAc. The combined organic layers were washed with brine, dried (Na_2SO_4), filtered, and concentrated *in vacuo*. Purification of the residue by column chromatography gave the arylative cyclization product **5**. In most cases, small quantities of minor products **6** and **7** were also observed in the ^{1}H NMR spectra of the crude material, but with the exception of the reaction forming arylative cyclization product **5g** (*vide infra*), these were not isolated.

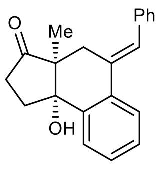

(3a*S*,9b*S*)-5-[(*E*)-Benzylidene)-9b-hydroxy-3a-methyl-1,2,3a,4,5,9b-hexahydro–3*H*-cyclopenta[*a*]naphthalen-3-one (5a).[1] The title compound was prepared according to General Procedure B using alkynyl 1,3-diketone **4a** (67.8 mg, 0.30 mmol) and phenylboronic acid (54.9 mg, 0.45 mmol), and purified by column chromatography (10% EtOAc/petroleum ether) to give a white solid (63 mg, 69%) whose spectroscopic data matched those reported previously.[1] m.p. 145–147 °C (MeOH); $[\alpha]_D^{20.1}$ –164 (*c* 1.00, CH_2Cl_2); Enantiomeric excess was determined by HPLC with a Chiralpak IA-3 column (80:20

isohexane:*i*-PrOH, 1.0 mL/min, 230 nm, 23 °C): t_r (minor) = 13.5 min, t_r (major) = 15.3 min, 95% ee.

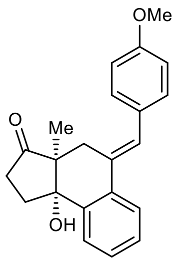

(3a*S*,9b*S*)-9b-Hydroxy-5-[(*E*)-4-methoxybenzylidene]-3a-methyl-1,2,3a,4,5,9b-hexahydro-3*H*-cyclopenta[*a*]naphthalene-3-one (5b).[1] The title compound was prepared according to General Procedure B using alkynyl 1,3-diketone **4b** (76.8 mg, 0.30 mmol) and phenylboronic acid (54.8 mg, 0.45 mmol), and purified by column chromatography (20% EtOAc/pentane) to give a white solid (37.7 mg, 39%) whose spectroscopic data matched those reported previously.[1] m.p. 173–175 °C (Et_2O); $[\alpha]_D^{20.1}$ –172 (*c* 1.00, $CHCl_3$); Enantiomeric excess was determined by HPLC with a Chiralpak AD-H column (80:20 isohexane:*i*-PrOH, 1.0 mL/min, 280 nm, 23 °C): t_r (minor) = 9.6 min, t_r (major) = 17.3 min, 98% ee.

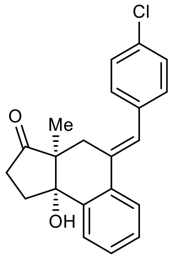

(3a*S*,9b*S*)-9b-Hydroxy-5-[(*E*)-4-chlorobenzylidene]-3a-methyl-1,2,3a,4,5,9b-hexahydro-3*H*-cyclopenta[*a*]naphthalen-3-one (5c).[1] The title compound was prepared according to General Procedure B using alkynyl 1,3-diketone **4c** (78.2 mg, 0.30 mmol) and phenylboronic acid (54.8 mg, 0.45 mmol), and purified by column chromatography (10:8:1 petroleum ether/CH_2Cl_2/EtOAc) to give a pale yellow solid (75.3 mg, 74%) whose spectroscopic data matched those reported previously.[1] m.p. 207–208 °C (MeOH); $[\alpha]_D^{22.0}$ –236 (*c* 1.00, CH_2Cl_2); Enantiomeric excess was determined by HPLC with a Chiralpak IA-3 column (80:20 isohexane:*i*-PrOH, 1.0 mL/min, 230 nm, 23 °C): t_r (minor) = 13.7 min, t_r (major) = 16.4 min, 99% ee.

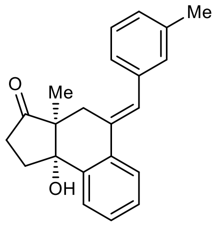

(3a*S*,9b*S*)-9b-Hydroxy-3a-methyl-5-[(*E*)-3-methylbenzylidene]-1,2,3a,4,5,9b-hexahydro-3*H*-cyclopenta[*a*]naphthalen-3-one (5d).[1] The title compound was prepared according to a modification of General Procedure B (in that the quantities of the rhodium precatalyst, ligand, and boronic acid were increased) using $[Rh(C_2H_4)_2Cl]_2$ (5.8 mg, 0.015 mmol), (*S*)-DTBM-SEGPHOS (**L2**, 35.4 mg, 0.03 mmol), alkynyl 1,3-diketone **4d** (81.3 mg, 0.30 mmol), and phenylboronic acid (73.2 mg, 0.60 mmol), and purified by column chromatography (10% Et_2O/CH_2Cl_2) to give a white solid (57.0 mg, 60%) whose spectroscopic data matched those reported previously.[1] m.p. 144–146 °C (Et_2O); $[\alpha]_D^{20.1}$ –204 (*c* 1.00, $CHCl_3$); Enantiomeric excess was determined by HPLC with a Chiralpak OD-H column (95:5 isohexane:*i*-PrOH, 0.8 mL/min, 254 nm, 23 °C): t_r (minor) = 18.8 min, t_r (major) = 22.7 min, 99% ee.

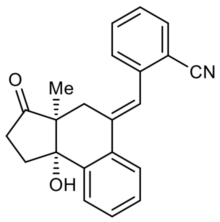

2-{(*E*)-[(3a*S*,9b*S*)-9b-Hydroxy-3a-methyl-3-oxo-1,2,3,3a,4,9b-hexahydro-5*H*-cyclopenta[*a*]naphthalen-5-ylidene]methyl}benzonitrile (5e).[1] The title compound was prepared according to a modification of General Procedure B (in that the quantities of the rhodium precatalyst and ligand were increased) using $[Rh(C_2H_4)_2Cl]_2$ (5.8 mg, 0.015 mmol), (*S*)-DTBM-SEGPHOS (**L2**, 35.4 mg, 0.03 mmol), alkynyl 1,3-diketone **4e** (75.3 mg, 0.30 mmol), and phenylboronic acid (54.9 mg, 0.45 mmol), and purified by column chromatography (10% Et_2O/CH_2Cl_2) to give a white solid (63.0 mg, 64%) whose spectroscopic data matched those reported previously.[1] R_f = 0.23 (10% Et_2O/CH_2Cl_2); m.p. 153–155 °C (Et_2O); $[\alpha]_D^{20.1}$ –116 (*c* 1.00, $CHCl_3$); Enantiomeric excess was determined by HPLC with a Chiralpak AD-H column (80:20 isohexane:*i*-PrOH, 0.8 mL/min, 254 nm, 23 °C): t_r (minor) = 12.7 min, t_r (major) = 29.6 min, 61% ee.

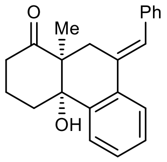

(4a*S*,10a*S*)-9-[(*E*)-Benzylidene]-4a-hydroxy-10a-methyl-3,4,4a,9,10,10a-hexahydrophenanthren-1(2*H*)-one (5f).[1] The title compound was prepared according to General Procedure B using alkynyl 1,3-diketone **4f** (72.1 mg, 0.30 mmol) and phenylboronic acid (54.9 mg, 0.45 mmol), and purified by column chromatography (10:8:1 petroleum ether/CH_2Cl_2/EtOAc) to give a pale yellow solid (57.6 mg, 60%) whose spectroscopic data matched those reported previously.[1] R_f = 0.27 (5:4:1 petroleum ether/CH_2Cl_2/EtOAc); $[\alpha]_D^{20.8}$ –96 (*c* 1.00, CH_2Cl_2); Enantiomeric excess was determined by HPLC with a Chiralpak IB-3 column (80:20 isohexane:*i*-PrOH, 1.0 mL/min, 230 nm, 23 °C): t_r (minor) = 14.0 min, t_r (major) = 15.5 min, 97% ee.

(3a*S*,9b*S*)-5-[(*E*)-Benzylidene]-9b-hydroxy-3a,8-dimethyl-1,2,3a,4,5,9b-hexahydro-3*H*-cyclopenta[*a*]naphthalen-3-one (5g), (*E*)-2-methyl-2-[3-phenyl-3-(*p*-tolyl)allyl]cyclopentane-1,3-dione (6g), and (*Z*)-5-methyl-7-[phenyl(*p*-tolyl)methylene]cycloheptane-1,4-dione (7g)

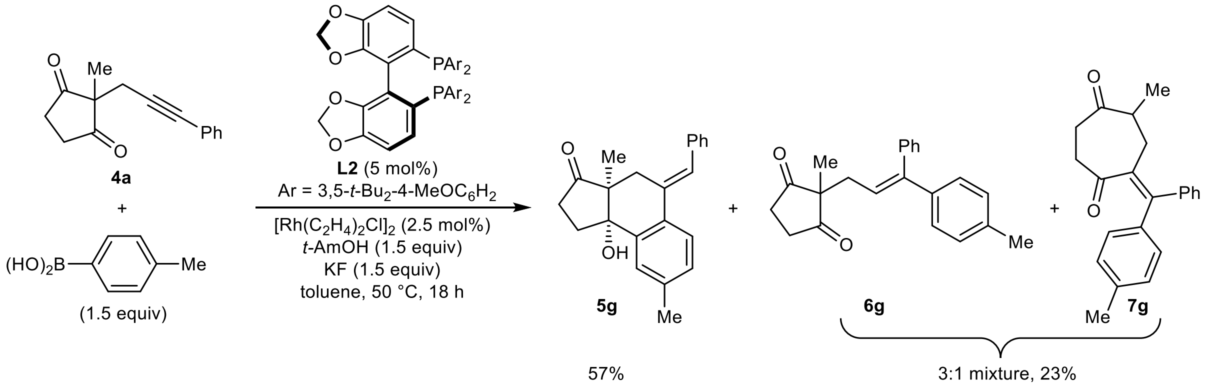

General Procedure B was followed using alkynyl 1,3-diketone **4a** (67.8 mg, 0.30 mmol) and 4-methylphenylboronic acid (61.2 mg, 0.45 mmol). Purification by column chromatography (20%

EtOAc/pentane) gave a 3:1 inseparable mixture of *products* **6g** and **7g** (22.7 mg, 23%) as a colorless oil followed by *arylative cyclization product* **5g** as a yellow solid (54.5 mg, 57%).

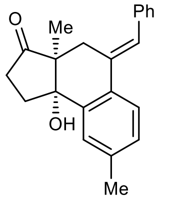

(3a*S*,9b*S*)-5-[(*E*)-Benzylidene]-9b-hydroxy-3a,8-dimethyl-1,2,3a,4,5,9b-hexahydro-3*H*-cyclopenta[*a*]naphthalen-3-one (5g). R*f* = 0.25 (30% EtOAc/pentane); m.p. 152–154 °C (Et_2O); $[\alpha]_D^{20.1}$ –172 (*c* 1.00, $CHCl_3$); IR (ATR) 3414 (OH), 2922, 1720 (C=O), 1446, 1200, 1069, 841, 705 cm^{-1}; ^{1}H NMR (400 MHz, $CDCl_3$) δ 7.58 (1H, d, *J* = 8.1 Hz, Ar**H**), 7.52 (1H, s, Ar**H**), 7.37–7.34 (2H, m, Ar**H** and C=C**H**), 2.77 (1H, d, *J* = 14.1 Hz, C**H**$_a$H$_b$C=C), 2.68–2.57 (2H, m, CH$_a$**H**$_b$C=C and C**H**$_a$H$_b$C=O), 2.54–2.27 (3H, m, CH$_a$**H**$_b$C=O and C**H**$_2$COH), 2.42 (3H, s, ArC**H**$_3$), 1.80 (1H, d, *J* = 2.2 Hz, O**H**), 1.00 (3H, s, 3H, s, C**H**$_3$CC=O); ^{13}C NMR (101 MHz, $CDCl_3$) δ 220.0 (C), 139.9 (C), 138.8 (C), 137.2 (C), 132.0 (C), 131.5 (C), 129.14 (CH), 129.09 (2 × CH), 128.4 (2 × CH), 127.0 (CH), 126.9 (CH), 124.1 (CH), 80.1 (C), 54.2 (C), 35.9 (CH_2), 35.2 (CH_2), 34.3 (CH_2), 21.3 (CH_3), 13.8 (CH_3); HRMS (ESI) Exact mass calculated for $C_{22}H_{22}NaO_2^+$ $[M+Na]^+$: 341.1517, found: 341.1506; Enantiomeric excess was determined by HPLC with a Chiralpak AD-H column (80:20 isohexane:*i*-PrOH, 1.0 mL/min, 280 nm, 23 °C): t$_r$ (minor) = 7.0 min, t$_r$ (major) = 10.6 min, 99% ee.

Slow diffusion of pentane into a solution of **5g** in CH_2Cl_2 gave crystals that were suitable for X-ray crystallography:

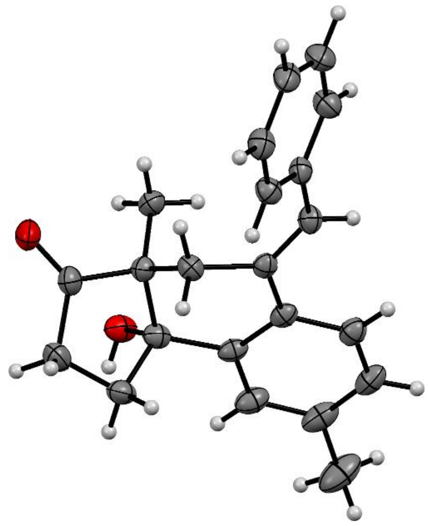

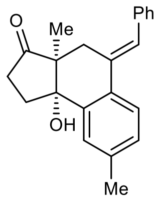

5g
CCDC 1959877
Flack parameter = 0.04(15)

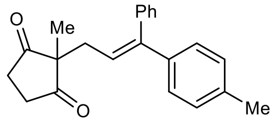

(*E*)-2-Methyl-2-[3-phenyl-3-(*p*-tolyl)allyl]cyclopentane-1,3-dione (6g). R*f* = 0.28 (30% EtOAc/pentane); Characteristic NMR signals of **6g**: ^{1}H NMR (400 MHz, $CDCl_3$) δ 5.87 (1H, t, *J* = 7.6 Hz, =C**H**), 2.80–2.65 (4H, m, C**H**$_2$C**H**$_2$), 2.43 (2H, d, *J* = 7.6 Hz, =CHC**H**$_2$), 2.32 (3H, s, ArC**H**$_3$), 1.12 (3H, s, C**H**$_3$CCH$_2$); ^{13}C NMR (101 MHz, $CDCl_3$) δ 215.5 (2 × C), 139.2 (C), 139.1 (C), 137.3 (C), 131.6 (C), 129.7 (2 × CH),

128.8 (2 × CH), 128.3 (2 × CH), 127.3 (CH), 127.2 (2 × CH), 120.3 (CH), 57.0 (C), 35.8 (CH_2), 35.1 (2 × CH_2), 21.1 (CH_3), 17.1 (CH_3).

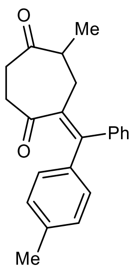

(*Z*)-5-Methyl-7-[phenyl(*p*-tolyl)methylene]cycloheptane-1,4-dione (7g). R*f* = 0.28 (30% EtOAc/pentane); Characteristic NMR signals of **7g**: ^{1}H NMR (400 MHz, $CDCl_3$) δ 2.80–2.65 (6H, C**H**$_2$C**H**$_2$ and CHC**H**$_2$), 2.31 (3H, s, ArC**H**$_3$), 2.28–2.19 (1H, m, C**H**CH$_3$), 0.99 (3H, d, *J* = 6.4 Hz, CHC**H**$_3$); ^{13}C NMR (101 MHz, $CDCl_3$) δ 211.7 (C), 207.8 (C), 144.7 (C), 140.1 (C), 45.3 (CH), 39.0 (CH_2), 37.6 (CH_2), 34.4 (CH_2), 21.2 (CH_3), 15.6 (CH_3).

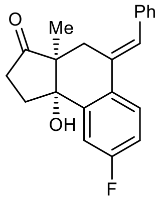

(3a*S*,9b*S*)-5-[(*E*)-Benzylidene]-8-fluoro-9b-hydroxy-3a-methyl-1,2,3a,4,5,9b-hexahydro-3*H*-cyclopenta[*a*]naphthalen-3-one (5h). The title compound was prepared according to a modification of General Procedure B (in that the quantities of the rhodium precatalyst and ligand were increased) using $[Rh(C_2H_4)_2Cl]_2$ (5.8 mg, 0.015 mmol), (*S*)-DTBM-SEGPHOS (**L2**, 35.4 mg, 0.03 mmol), alkynyl 1,3-diketone **4a** (67.8 mg, 0.30 mmol), and 4-fluorophenylboronic acid (63.0 mg, 0.45 mmol), and purified by column chromatography (20% EtOAc/pentane) to give an off-white solid (63.4 mg, 65%). R*f* = 0.42 (40% EtOAc/pentane); m.p. 70–72 °C (Et_2O); $[\alpha]_D^{20.1}$ –200 (*c* 1.00, $CHCl_3$); IR (ATR) 3447 (OH), 2966, 1729 (C=O), 1605, 1582, 1446, 1239, 838, 697 cm^{-1}; ^{1}H NMR (400 MHz, $CDCl_3$) δ 7.67 (1H, dd, *J* = 8.8, 5.5 Hz, Ar**H**), 7.43 (1H, dd, *J* = 9.6, 2.8 Hz, Ar**H**), 7.40–7.37 (2H, m, Ar**H**), 7.31–7.25 (4H, m, Ar**H**), 7.17 (1H, s, C=C**H**), 7.06 (1H, ddd, *J* = 8.8, 8.0, 2.8 Hz, Ar**H**), 2.80 (1H, d, *J* = 14.1 Hz, C**H**$_a$H$_b$C=C), 2.68–2.58 (2H, m, CH$_a$**H**$_b$C=C and C**H**$_a$H$_b$C=O), 2.52 (1H, ddd, *J* = 19.1, 9.0, 2.5 Hz, CH$_a$**H**$_b$C=O), 2.45–2.29 (2H, m, C**H**$_2$COH), 1.84 (1H, d, *J* = 2.3 Hz, O**H**), 1.02 (3H, s, C**H**$_3$); ^{13}C NMR (101 MHz, $CDCl_3$) δ 219.3 (C), 163.2 (C, J_{CF} = 248.0 Hz), 142.4 (C, J_{CF} = 6.5 Hz), 136.9 (C), 131.2 (C), 130.4 (C, J_{CF} = 3.6 Hz), 129.1 (2 × CH), 128.5 (2 × CH), 127.7 (CH), 127.2 (CH), 126.3 (CH, J_{CF} = 8.1 Hz), 115.6 (CH, J_{CF} = 21.8 Hz), 113.1 (CH, J_{CF} = 22.0 Hz), 80.0 (C), 54.0 (C), 35.8 (CH_2), 35.0 (CH_2), 34.4 (CH_2), 13.7 (CH_3); ^{19}F NMR (376 MHz, $CDCl_3$) δ –113.2; HRMS (ESI) Exact mass calculated for $C_{21}F_1H_{19}NaO_2^+$ [M+Na]$^+$: 345.1261, found: 345.1259; Enantiomeric excess was determined by HPLC with a Chiralpak AD-H column (80:20 isohexane:*i*-PrOH, 1.0 mL/min, 254 nm, 23 °C): t_r (minor) = 6.5 min, t_r (major) = 10.5 min, 99% ee.

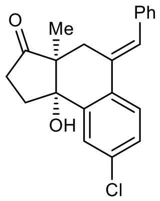

(3a*S*,9b*S*)-5-[(*E*)-Benzylidene]-8-chloro-9b-hydroxy-3a-methyl-1,2,3a,4,5,9b-hexahydro-3*H*-cyclopenta[*a*]naphthalen-3-one (5i).[1] The title compound was prepared according to General **Procedure B** using alkynyl 1,3-diketone **4a** (67.8 mg, 0.30 mmol) and 4-chlorophenylboronic acid (70.4 mg, 0.45 mmol), and purified by

column chromatography (20% EtOAc/pentane) to give an off-white solid (66.1 mg, 65%) whose spectroscopic data matched those reported previously.[1] R_f = 0.43 (40% EtOAc/pentane); m.p. 78–80 °C (Et_2O); $[\alpha]_D^{20.1}$ –224 (*c* 1.00, $CHCl_3$); Enantiomeric excess was determined by HPLC with a Chiralpak AD-H column (80:20 isohexane:*i*-PrOH, 1.0 mL/min, 280 nm, 23 °C): t_r (minor) = 6.9 min, t_r (major) = 12.3 min, 98% ee.

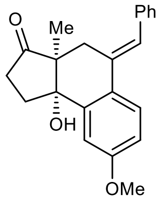

(3a*S*,9b*S*)-5-[(*E*)-Benzylidene]-9b-hydroxy-8-methoxy-3a-methyl-1,2,3a,4,5,9b–hexahydro-3*H*-cyclopenta[*a*]naphthalen-3-one (5j).[1] The title compound was prepared according to General Procedure B using alkynyl 1,3-diketone **4a** (67.8 mg, 0.30 mmol) and 4-methoxyphenylboronic acid (68.5 mg, 0.45 mmol), and purified by column chromatography (10:8:1 petroleum ether/CH_2Cl_2/EtOAc) to give a pale yellow solid (55 mg, 55%) whose spectroscopic data matched those reported previously.[1] m.p. 166–168 °C; $[\alpha]_D^{20.0}$ –188 (*c* 1.00, CH_2Cl_2); Enantiomeric excess was determined by HPLC with a Chiralpak IA-3 column (80:20 isohexane:*i*-PrOH, 1 mL/min, 230 nm, 23 °C): t_r (minor) = 16.0 min, t_r (major) = 21.3 min, 99% ee.

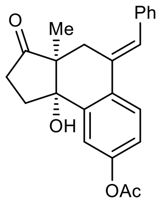

(3a*S*,9b*S*)-5-[(*E*)-Benzylidene]-9b-hydroxy-3a-methyl-3-oxo-2,3,3a,4,5,9b-hexahydro-1*H*-cyclopenta[*a*]naphthalen-8-yl acetate (5k). The title compound was prepared according to a modification of General Procedure B (in that the quantities of the rhodium precatalyst, ligand, and boronic acid were increased) using $[Rh(C_2H_4)_2Cl]_2$ (5.8 mg, 0.015 mmol), (*S*)-DTBM-SEGPHOS (**L2**, 35.4 mg, 0.03 mmol), alkynyl 1,3-diketone **4a** (67.8 mg, 0.30 mmol), and 4-acetoxyphenylboronic acid (108 mg, 0.60 mmol), and purified by column chromatography (10% Et_2O/CH_2Cl_2) to give a yellow solid (66.0 mg, 61%). R_f = 0.30 (10% Et_2O/CH_2Cl_2); m.p. 95–97 °C (Et_2O); $[\alpha]_D^{20.1}$ –208 (*c* 1.00, $CHCl_3$); IR (ATR) 3466 (OH), 2955, 1735 (C=O), 1493, 1368, 1200, 815, 698 cm^{-1}; ^1H NMR (400 MHz, $CDCl_3$) δ 7.69 (1H, d, *J* = 8.7 Hz, Ar**H**), 7.44 (1H, d, *J* = 2.5 Hz, Ar**H**), 7.38–7.35 (2H, m, Ar**H**), 7.29–7.25 (3H, m, Ar**H**), 7.19 (1H, app d, *J* = 2.1 Hz, C=C**H**), 7.08 (1H, dd, *J* = 8.6, 2.5 Hz, Ar**H**), 2.79 (1H, d, *J* = 14.2 Hz, C**H**$_a$H$_b$C=), 2.67–2.57 (2H, m, CH$_a$**H**$_b$C= and C**H**$_a$H$_b$C=O), 2.50 (1H, ddd, *J* = 19.1, 8.8, 2.6 Hz, CH$_a$**H**$_b$C=O), 2.42–2.29 (2H, m, C**H**$_2$COH), 2.35 (3H, s, C**H**$_3$C=O), 1.86 (1H, d, *J* = 2.3 Hz, O**H**), 1.00 (3H, s, C**H**$_3$CC=O); ^{13}C NMR (101 MHz, $CDCl_3$) δ 219.4 (C), 169.5 (C), 151.1 (C), 141.6 (C), 136.9 (C), 132.0 (C), 131.3 (C), 129.1 (2 × CH), 128.4 (2 × CH), 128.1 (CH), 127.2 (CH), 125.5 (CH), 121.7 (CH), 119.5 (CH), 80.0 (C), 54.0 (C), 35.8 (CH_2), 35.1 (CH_2), 34.4 (CH_2), 21.1 (CH_3), 13.7 (CH_3); HRMS (ESI) Exact mass calculated for $C_{23}H_{22}NaO_4^+$ $[M+Na]^+$: 385.1410, found:

385.1411; Enantiomeric excess was determined by HPLC with a Chiralpak AD-H column (80:20 isohexane:*i*-PrOH, 1.0 mL/min, 280 nm, 23 °C): t_r (minor) = 8.2 min, t_r (major) = 10.6 min, 99% ee.

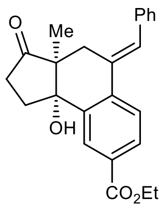

Ethyl (3a*S*,9b*S*)-5-[(*E*)-benzylidene]-9b-hydroxy-3a-methyl-3-oxo–2,3,3a,4,5,9b-hexahydro-1*H*-cyclopenta[*a*]naphthalene-8-carboxylate (5l).[1] The title compound was prepared according to a modification of General Procedure B (in that the quantities of the rhodium precatalyst, ligand, and boronic acid were increased) using $[Rh(C_2H_4)_2Cl]_2$ (5.8 mg, 0.015 mmol), (*S*)-DTBM-SEGPHOS (**L2**, 35.4 mg, 0.03 mmol), alkynyl 1,3-diketone **4a** (67.8 mg, 0.30 mmol), and 4-ethoxycarbonylphenylboronic acid (140 mg, 0.72 mmol), and purified by column chromatography (10% Et_2O/CH_2Cl_2) to give a yellow oily solid (73.4 mg, 65%) whose spectroscopic data matched those reported previously.[1] $[\alpha]_D^{20.1}$ –180 (*c* 1.00, $CHCl_3$); Enantiomeric excess was determined by HPLC with a Chiralpak OD-H column (90:10 isohexane:*i*-PrOH, 1.0 mL/min, 280 nm, 23 °C): t_r (minor) = 20.9 min, t_r (major) = 24.6 min, 99% ee.

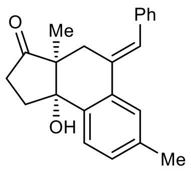

(3a*S*,9b*S*)-5-[(*E*)-Benzylidene]-9b-hydroxy-3a,7-dimethyl-1,2,3a,4,5,9b-hexahydro-3*H*-cyclopenta[*a*]naphthalen-3-one (5m).[1] The title compound was prepared according to General Procedure B using alkynyl 1,3-diketone **4a** (67.8 mg, 0.30 mmol) and 3-methylphenylboronic acid (61.2 mg, 0.45 mmol), and purified by column chromatography (20% EtOAc/pentane) to give a white solid (42.4 mg, 44%) whose spectroscopic data matched those reported previously.[1] m.p. 142–143°C (Et_2O); $[\alpha]_D^{20.1}$ –180 (*c* 1.00, $CHCl_3$); Enantiomeric excess was determined by HPLC with a Chiralpak AD-H column (80:20 isohexane:*i*-PrOH, 1.0 mL/min, 280 nm, 23 °C): t_r (minor) = 7.3 min, t_r (major) = 10.4 min, 99% ee.

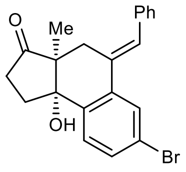

(3a*S*,9b*S*)-5-[(*E*)-Benzylidene]-7-bromo-9b-hydroxy-3a-methyl-1,2,3a,4,5,9b-hexahydro-3*H*-cyclopenta[*a*]naphthalen-3-one (5n).[1] The title compound was prepared according to General Procedure B using alkynyl 1,3-diketone **4a** (67.8 mg, 0.30 mmol) and 3-bromophenylboronic acid (90.5 mg, 0.45 mmol), and purified by column chromatography (10:8:1 petroleum ether/CH_2Cl_2/EtOAc) to give a white solid (71 mg, 61%) whose spectroscopic data matched those reported previously.[1] m.p. 173–174 °C (MeOH); $[\alpha]_D^{20.4}$ –60 (*c* 1.00, CH_2Cl_2); Enantiomeric excess was determined by HPLC with a Chiralpak IA-3 column (80:20 isohexane:*i*-PrOH, 0.8 mL/min, 230 nm, 23 °C): t_r (minor) = 16.1 min, t_r (major) = 18.0 min, 99% ee.

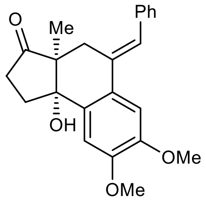

(3a*S*,9b*S*)-5-[(*E*)-Benzylidene]-9b-hydroxy-7,8-dimethoxy-3a-methyl-1,2,3a,4,5,9b-hexahydro-3*H*-cyclopenta[*a*]naphthalen-3-one (5o). The title compound was prepared according to a modification of General Procedure B (in that the quantities of the rhodium precatalyst, ligand, and boronic acid were increased) using $[Rh(C_2H_4)_2Cl]_2$ (5.8 mg, 0.015 mmol), (*S*)-DTBM-SEGPHOS (**L2**, 35.4 mg, 0.03 mmol), alkynyl 1,3-diketone **4a** (67.8 mg, 0.30 mmol), and 3,4-dimethoxyphenylboronic acid (96.1 mg, 0.60 mmol), and purified by column chromatography (10% Et_2O/CH_2Cl_2) to give a yellow solid (50.7 mg, 46%). R_f = 0.33 (10% Et_2O/CH_2Cl_2); m.p. 91–93 °C (Et_2O); $[\alpha]_D^{20.1}$ –164 (*c* 1.00, $CHCl_3$); IR (ATR) 3467 (OH), 2959, 2835, 1731 (C=O), 1446, 1200, 1052, 845, 698 cm^{-1}; ^1H NMR (400 MHz, $CDCl_3$) δ 7.40–7.35 (2H, m, Ar**H**), 7.30–7.26 (3H, m, Ar**H**), 7.21 (1H, s, Ar**H**), 7.14 (1H, s, Ar**H**), 7.12 (1H, app d, *J* = 2.0 Hz, C=C**H**), 3.98 (6H, s, 2 × OC**H**3), 2.77 (1H, d, *J* = 14.0 Hz, C**H**$_a$H$_b$C=), 2.68–2.57 (2H, m, CH$_a$**H**$_b$C= and C**H**$_a$H$_b$C=O), 2.54–2.29 (3H, m, CH$_a$**H**$_b$C=O and C**H**2COH), 1.79 (1H, d, *J* = 2.2 Hz, O**H**), 1.02 (3H, s, C**H**3CC=O); ^{13}C NMR (101 MHz, $CDCl_3$) δ 219.8 (C), 150.1 (C), 149.0 (C), 137.2 (C), 132.9 (C), 132.0 (C), 129.1 (2 × CH), 128.4 (2 × CH), 126.9 (CH), 126.1 (CH), 108.6 (CH), 106.5 (CH), 80.1 (C), 56.05 (CH_3), 55.98 (CH_3), 54.2 (C), 35.7 (CH_2), 35.3 (CH_2), 34.5 (CH_2), 13.9 (CH_3); HRMS (ESI) Exact mass calculated for $C_{23}H_{24}NaO_4^+$ $[M+Na]^+$: 387.1567, found: 387.1573; Enantiomeric excess was determined by HPLC with a Chiralpak OD-H column (85:15 isohexane:*i*-PrOH, 0.3 mL/min, 210 nm, 23 °C): t_r (minor) = 40.9 min, t_r (major) = 42.4 min, 99% ee.

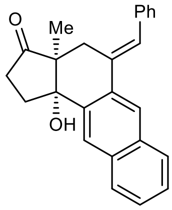

(3a*S*,11b*S*)-5-[(*E*)-Benzylidene]-11b-hydroxy-3a-methyl-1,2,3a,4,5,11b-hexahydro-3*H*-cyclopenta[*a*]anthracen-3-one (5p).[1] The title compound was prepared according to General Procedure B using alkynyl 1,3-diketone **4a** (67.8 mg, 0.30 mmol) and 2-naphthylboronic acid (77.4 mg, 0.45 mmol), and purified by column chromatography (20% EtOAc/pentane) to give a white solid (66.0 mg, 64%) whose spectroscopic data matched those reported previously.[1] m.p. 179–180 °C (Et_2O); $[\alpha]_D^{20.1}$ –212 (*c* 1.00, $CHCl_3$); Enantiomeric excess was determined by HPLC with a Chiralpak OD-H column (95:5 isohexane:*i*-PrOH, 1.0 mL/min, 210 nm, 23 °C): t_r (minor) = 30.8 min, t_r (major) = 33.7 min, 95% ee.

(5a*S*,8a*R*)-4-[(*E*)-Benzylidene]-8a-hydroxy-5a-methyl-4,5,5a,7,8,8a-hexahydro-6*H*-indeno[4,5-*c*]thiophen-6-one (5ra) and (5a*S*,8a*R*)-4-[(*E*)-benzylidene]-8a-hydroxy-5a-methyl-4,5,5a,7,8,8a-hexahydro-6*H*-indeno[4,5-*b*]thiophen-6-one (5rb)

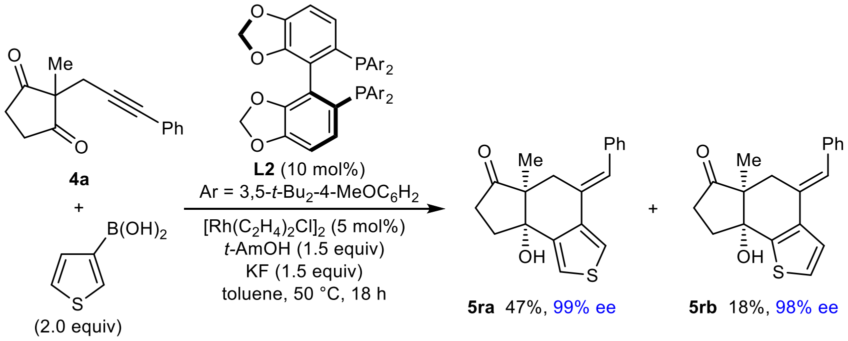

A modification of General Procedure B (in that the quantities of the rhodium precatalyst, ligand, and boronic acid were increased) was followed using $[Rh(C_2H_4)_2Cl]_2$ (5.8 mg, 0.015 mmol), (*S*)-DTBM-SEGPHOS (**L2**, 35.4 mg, 0.03 mmol), alkynyl 1,3-diketone **4a** (67.8 mg, 0.30 mmol) and 3-thienylboronic acid (76.8 mg, 0.60 mmol), and purified by column chromatography (10% Et_2O/CH_2Cl_2) to give *arylative cyclizaton product* **5ra** (43.5 mg, 47%) as a yellow solid followed by *arylative cyclizaton product* **5rb** (16.7 mg, 18%) as a yellow solid.

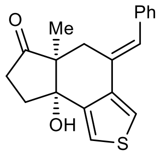

(5a*S*,8a*R*)-4-[(*E*)-Benzylidene]-8a-hydroxy-5a-methyl-4,5,5a,7,8,8a-hexahydro-6*H*-indeno[4,5-*c*]thiophen-6-one (5ra). R_f = 0.19 (10% Et_2O/CH_2Cl_2); m.p. 126–128 °C (Et_2O); $[\alpha]_D^{20.1}$ –196 (*c* 1.00, $CHCl_3$); IR (ATR) 3434 (OH), 2958, 2924, 2852, 1730, 1442, 1050, 962, 792, 696 cm^{-1}; ^1H NMR (400 MHz, $CDCl_3$) δ 7.50 (1H, d, *J* = 3.1 Hz, Ar**H**), 7.48 (1H, d, *J* = 3.1 Hz, Ar**H**), 7.38–7.35 (2H, m, Ar**H**), 7.29–7.27 (3H, m, Ar**H**), 7.16 (1H, app d, *J* = 2.3 Hz, C=C**H**), 2.75 (1H, dd, *J* = 14.8, 1.0 Hz, C**H**$_a$H$_b$C=), 2.68–2.56 (2H, m, CH$_a$**H**$_b$C= and C**H**$_a$H$_b$C=O), 2.52–2.36 (3H, m, CH$_a$**H**$_b$C=O and C**H**$_2$COH), 1.90 (1H, d, *J* = 2.0 Hz, O**H**), 0.99 (3H, s, C**H**$_3$); ^{13}C NMR (101 MHz, $CDCl_3$) δ 219.2 (C), 142.9 (C), 137.5 (C), 136.6 (C), 129.1 (2 × CH), 128.4 (2 × CH), 127.0 (CH), 126.5 (CH), 122.3 (CH), 118.5 (CH), 79.4 (C), 55.1 (C), 35.3 (CH_2), 35.0 (CH_2), 34.8 (CH_2), 13.7 (CH_3); HRMS (ESI) Exact mass calculated for $C_{19}H_{18}NaO_2S^+$ [M+Na]$^+$: 333.0920, found: 333.0920; Enantiomeric excess was determined by HPLC with a Chiralpak OD-H column (90:10 isohexane:*i*-PrOH, 1.0 mL/min, 230 nm, 23 °C): t_r (major) = 12.5 min, t_r (minor) = 14.1 min, 99% ee.

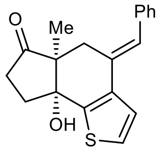

(5a*S*,8a*R*)-4-[(*E*)-Benzylidene]-8a-hydroxy-5a-methyl-4,5,5a,7,8,8a-hexahydro-6*H*-indeno[4,5-*b*]thiophen-6-one (5rb). R_f = 0.14 (10% Et_2O/CH_2Cl_2); m.p. 157–159 °C (Et_2O); $[\alpha]_D^{20.1}$ –8.0 (*c* 0.20, $CHCl_3$); IR (ATR) 3362 (OH), 2948, 2928, 1721 (C=O), 1395, 1126, 1073, 755, 698 cm^{-1}; ^1H NMR (400 MHz, $CDCl_3$) δ 7.41–7.28 (6H, m, Ar**H**),

7.24 (1H, d, *J* = 5.3 Hz, Ar**H**), 7.01 (1H, s, C=C**H**), 2.92 (dd, *J* = 15.2, 1.9 Hz, C**H**$_a$H$_b$C=), 2.63 (1H, dd, *J* = 15.2, 1.6 Hz, CH$_a$**H**$_b$C=), 2.59–2.41 (3H, m, C**H**$_2$C=O and C**H**$_a$H$_b$COH), 2.35–2.25 (1H, m, CH$_a$**H**$_b$COH), 2.09 (1H, br s, O**H**), 1.10 (3H, s, C**H**$_3$); ^{13}C NMR (101 MHz, CDCl$_3$) δ 217.9 (C), 141.9 (C), 138.1 (C), 136.7 (C), 129.1 (2 × CH), 129.0 (C), 128.4 (2 × CH), 127.0 (CH), 126.3 (CH), 125.9 (CH), 123.4 (CH), 78.2 (C), 54.9 (C), 35.8 (CH$_2$), 35.2 (CH$_2$), 33.2 (CH$_2$), 15.8 (CH$_3$); HRMS (ESI) Exact mass calculated for $C_{19}H_{18}NaO_2S^+$ [M+Na]$^+$: 333.0920, found: 333.0927; Enantiomeric excess was determined by HPLC with a Chiralpak OD-H column (95:5 isohexane:*i*-PrOH, 1.0 mL/min, 280 nm, 23 °C): t$_r$ (major) = 23.0 min, t$_r$ (minor) = 29.2 min, 98% ee.

The structural similarity of the two products **5ra** and **5rb** make confirmation of their exact structure challenging. However, in the major product **5ra**, the coupling constant observed between the two thiophene protons are indicative of a thiophene 2,5-relationship (^3J = 3.1 Hz vs expected range 0–4 Hz), whereas in the minor product **5rb**, the coupling constant observed between the two thiophene protons are indicative of a thiophene 2,3-relationship (^2J = 5.3 Hz vs expected range 4–8 Hz).

(*E*)-4-Benzylidene-1-methyl-1,2,3,4-tetrahydronaphthalen-1-ol (7)

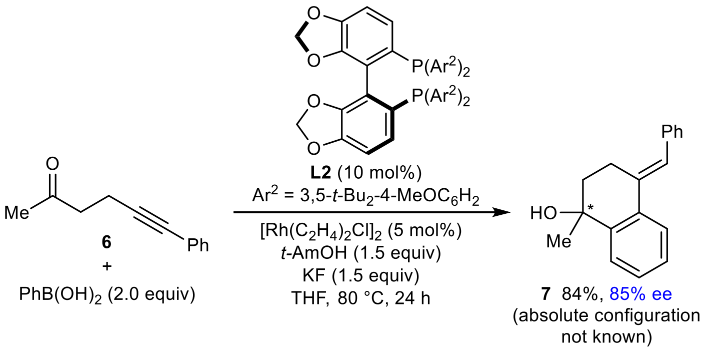

The title compound was prepared according to General Procedure A using alkynyl diketone **6** (51.6 mg, 0.30 mmol) and phenylboronic acid (73.2 mg, 0.60 mmol), and purified by column chromatography (5% to 10% EtOAc/pentane) to give an orange oil (62.8 mg, 84%). R$_f$ = 0.30 (30% EtOAc/pentane); $[\alpha]_D^{25.0}$ –20.0 (*c* 1.00, CHCl$_3$); IR (ATR) 3303 (OH), 3027, 2941, 2851, 1596, 1443, 1030, 922, 780, 695 cm^{-1}; ^{1}H NMR (500 MHz, CDCl$_3$) δ 7.68 (2H, ddd, *J* = 9.7, 7.7, 1.5 Hz, Ar**H**), 7.41–7.39 (4H, m, Ar**H**), 7.36–7.25 (3H, m, Ar**H**), 7.06 (1H, t, *J* = 1.7 Hz, C=C**H**), 3.01 (1H, dddd, *J* = 15.3, 6.5, 4.8, 1.7 Hz, =CC**H**$_a$H$_b$), 2.81 (1H, dddd, *J* = 15.3, 10.3, 4.8, 2.0 Hz, =CCH$_a$**H**$_b$), 2.04 (1H, ddd, *J* = 12.9, 6.1, 1.4 Hz, HOCC**H**$_a$H$_b$), 1.95 (1H, ddd, *J* = 12.9, 10.2, 4.8 Hz, HOCCH$_a$**H**$_b$), 1.79 (1H, br s, O**H**), 1.57 (3H, s, C**H**$_3$); ^{13}C NMR (126 MHz, CDCl$_3$) δ 143.3 (C), 137.8 (C), 136.4 (C), 135.2 (C), 129.3 (2 × CH), 128.2 (2 × CH), 128.1 (CH), 127.6 (CH), 126.7 (CH), 125.3 (CH), 124.9 (CH), 124.4 (CH), 70.8 (C), 39.2 (CH$_2$), 29.5 (CH$_3$), 25.3 (CH$_2$); HRMS (ESI) Exact mass calculated for $[C_{18}H_{18}NaO]^+$ [M+Na]$^+$: 273.1250, found 273.1246; Enantiomeric excess was

determined by HPLC with a Chiralpak AS-H column (90:10 isohexane:*i*-PrOH, 0.5 mL/min, 280 nm, 25 °C): t_r (minor) = 14.0 min, t_r (major) = 15.0 min, 85% ee.

NMR Spectra of New Compounds

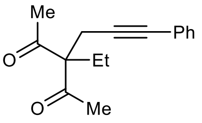

1b

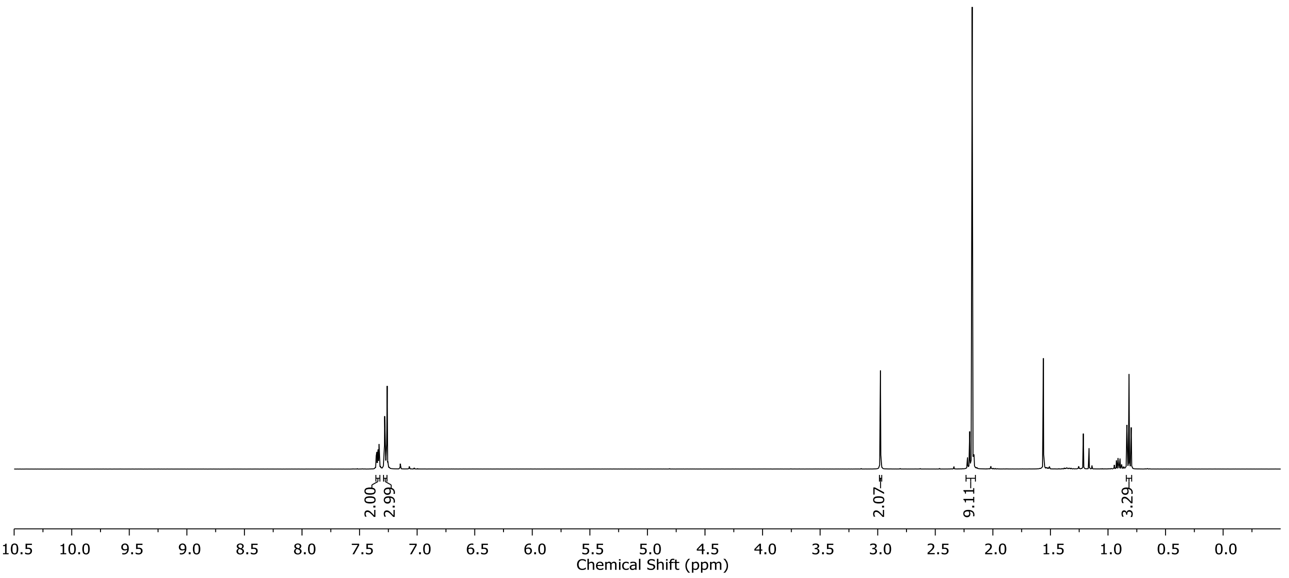

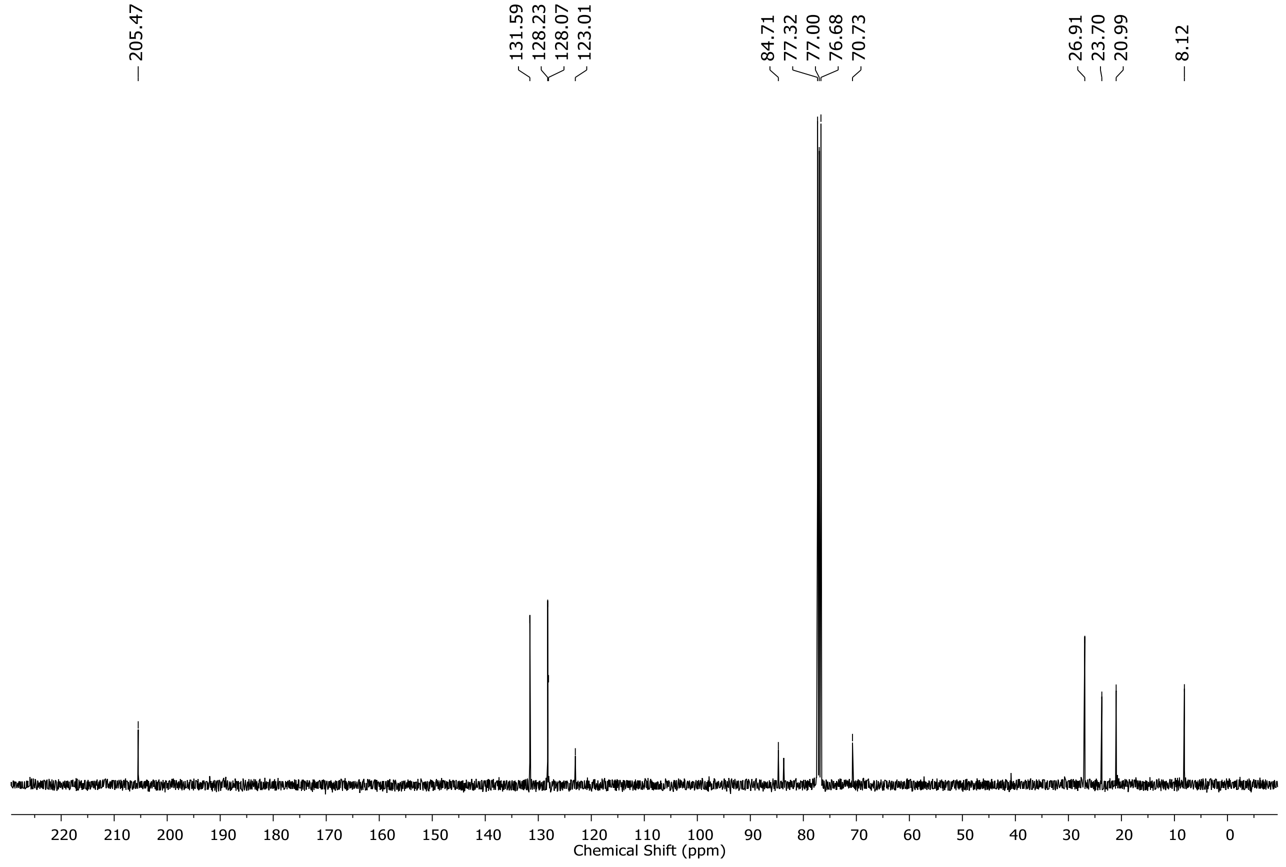

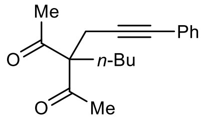
Me
Ph
O
n-Bu
O
Me

1c

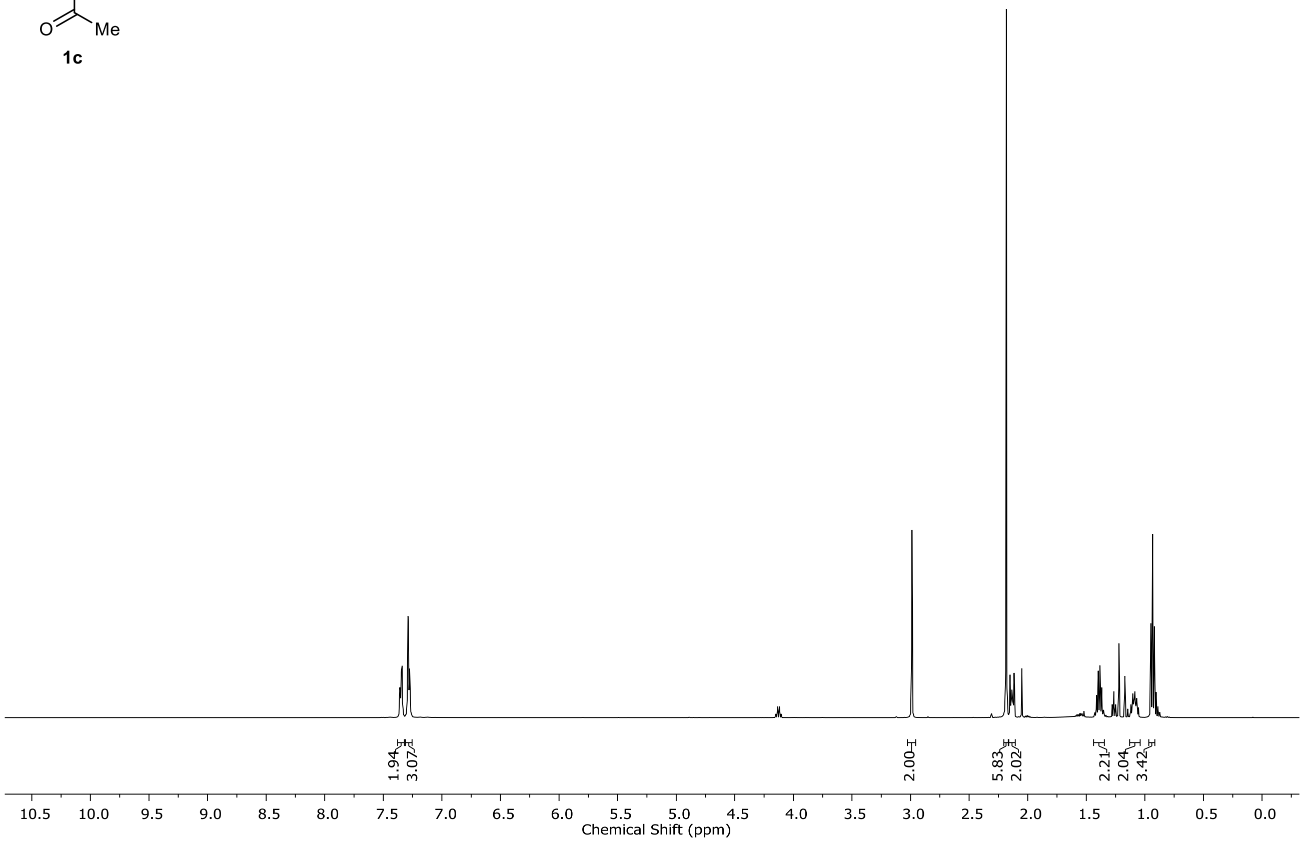
1.94
3.07
2.00
5.83
2.02
2.21
2.04
3.42
10.5 10.0 9.5 9.0 8.5 8.0 7.5 7.0 6.5 6.0 5.5 5.0 4.5 4.0 3.5 3.0 2.5 2.0 1.5 1.0 0.5 0.0
Chemical Shift (ppm)

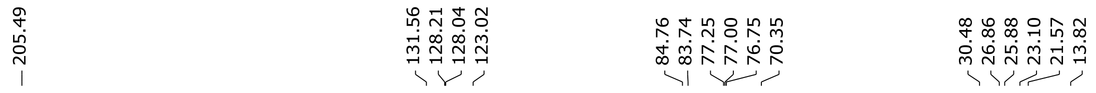
205.49
131.56
128.21
128.04
123.02
84.76
83.74
77.25
77.00
76.75
70.35
30.48
26.86
25.88
23.10
21.57
13.82

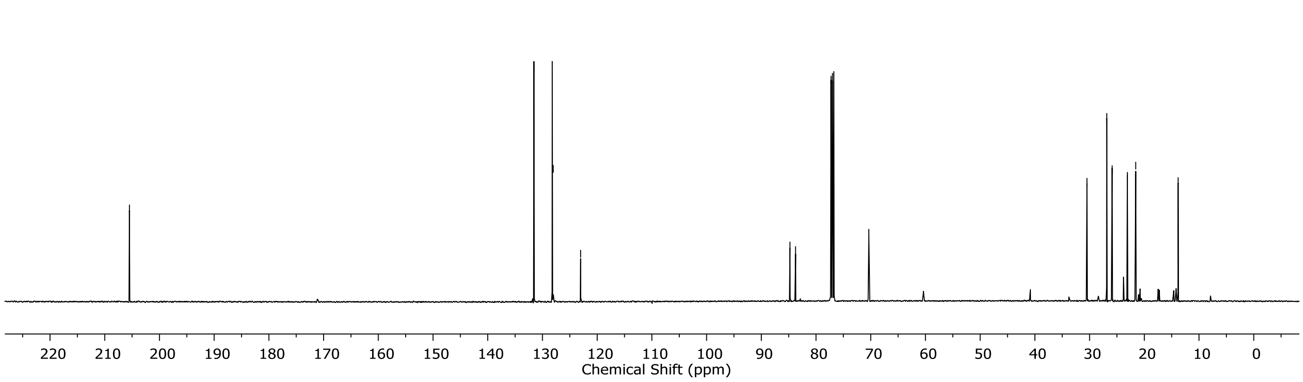
220 210 200 190 180 170 160 150 140 130 120 110 100 90 80 70 60 50 40 30 20 10 0
Chemical Shift (ppm)

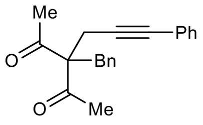
Me
Ph
O
Bn
O
Me

1d

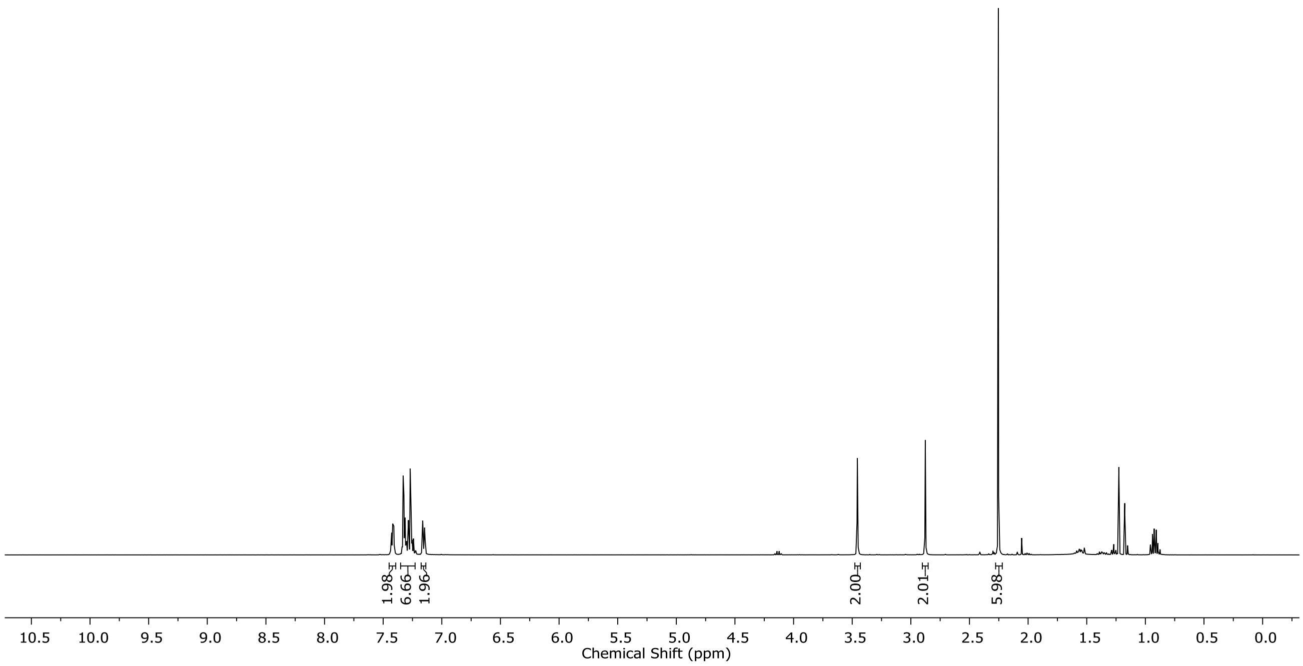
1.98
6.66
1.96
2.00
2.01
5.98
10.5 10.0 9.5 9.0 8.5 8.0 7.5 7.0 6.5 6.0 5.5 5.0 4.5 4.0 3.5 3.0 2.5 2.0 1.5 1.0 0.5 0.0
Chemical Shift (ppm)

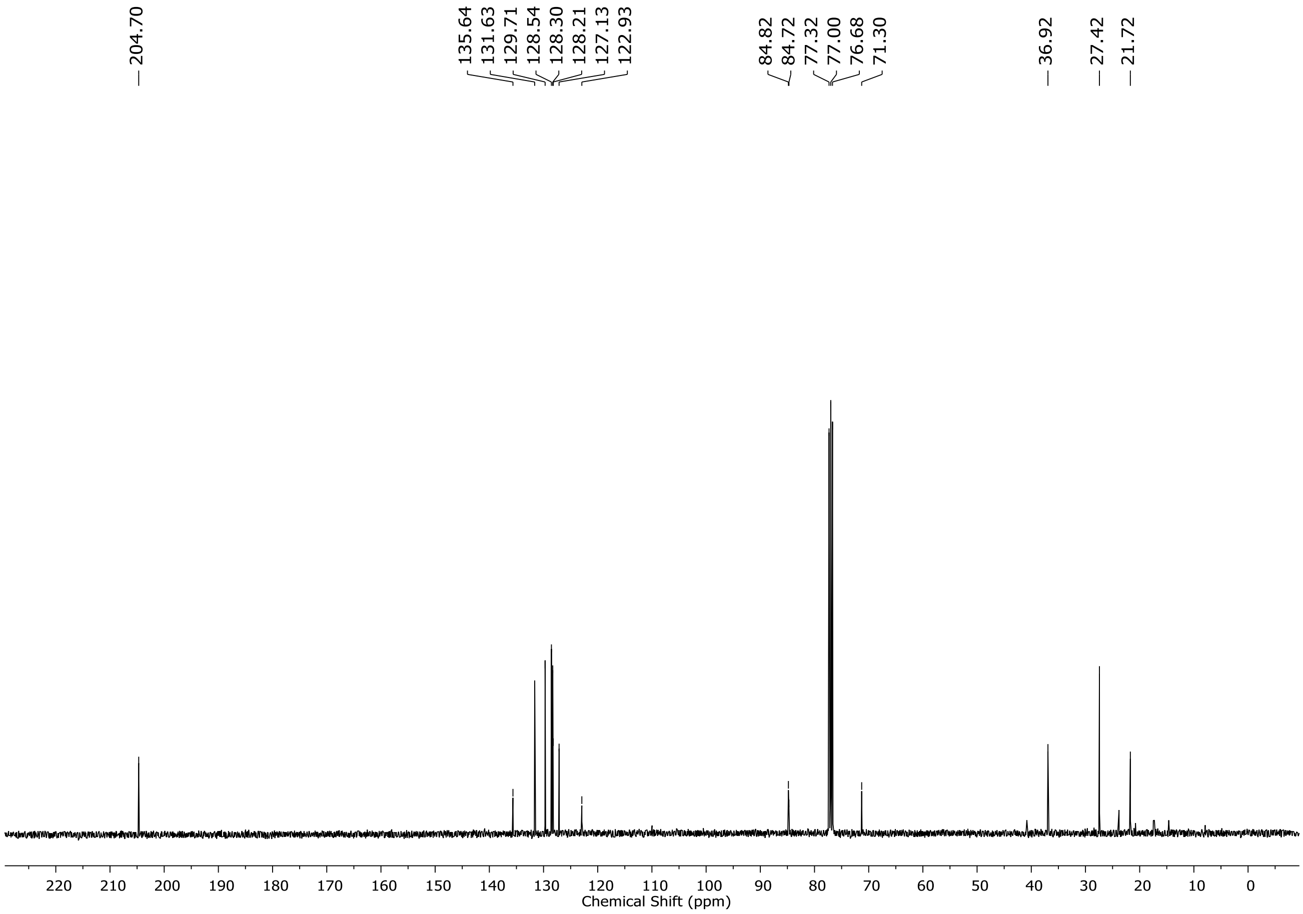
204.70
135.64
131.63
129.71
128.54
128.30
128.21
127.13
122.93
84.82
84.72
77.32
77.00
76.68
71.30
36.92
27.42
21.72
220 210 200 190 180 170 160 150 140 130 120 110 100 90 80 70 60 50 40 30 20 10 0
Chemical Shift (ppm)

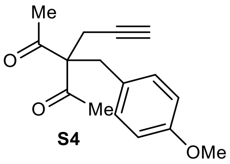
Me
O
O
Me
OMe

S4

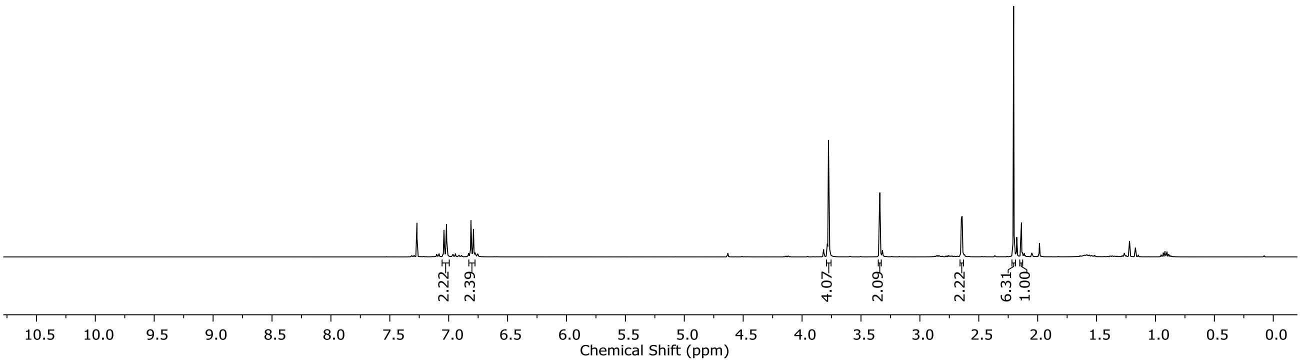
2.22
2.39
4.07
2.09
2.22
6.31
1.00
Chemical Shift (ppm)

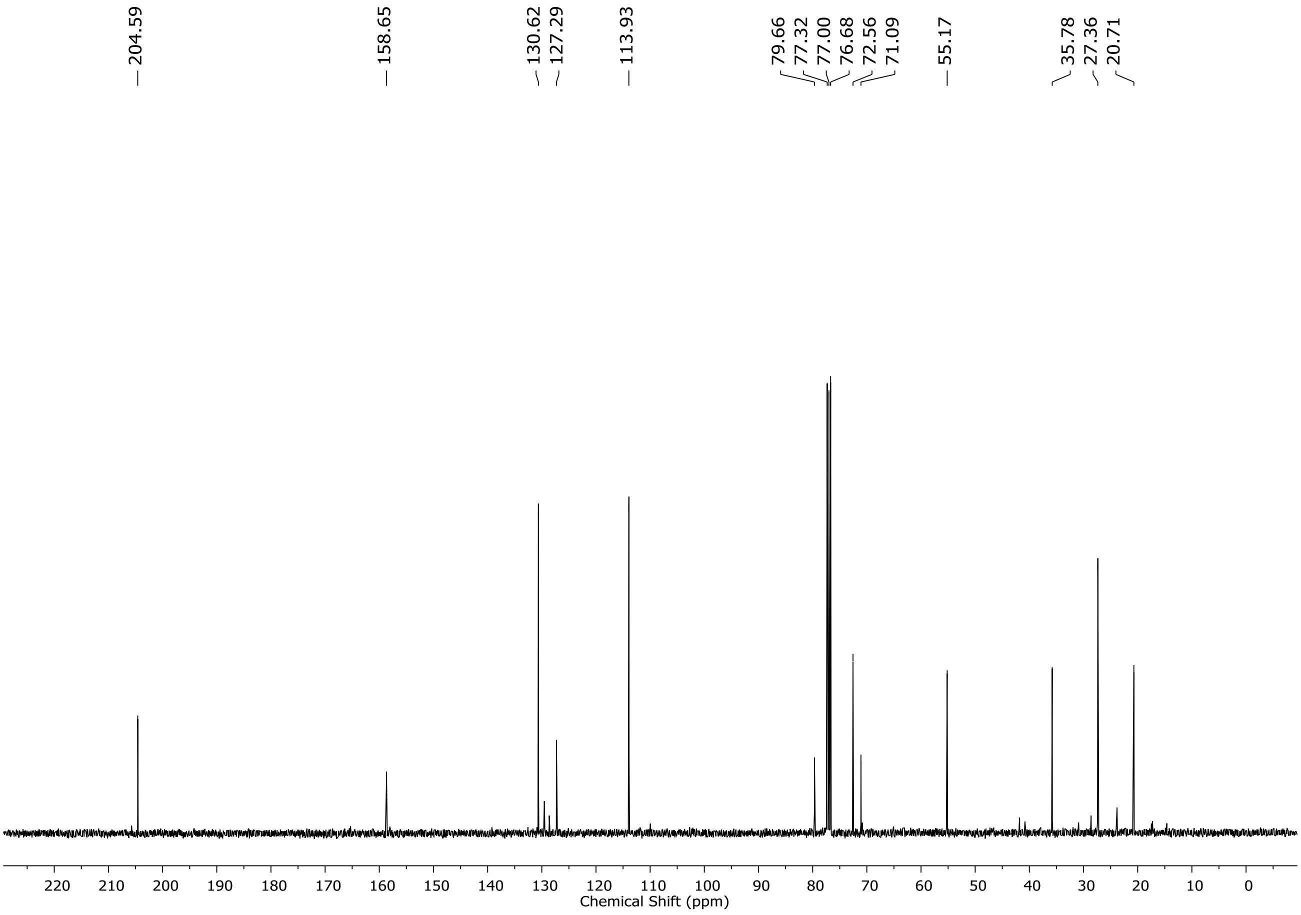
204.59
158.65
130.62
127.29
113.93
79.66
77.32
77.00
76.68
72.56
71.09
55.17
35.78
27.36
20.71
Chemical Shift (ppm)

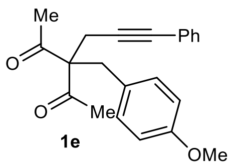
Me
Ph
O
O
Me
OMe

1e

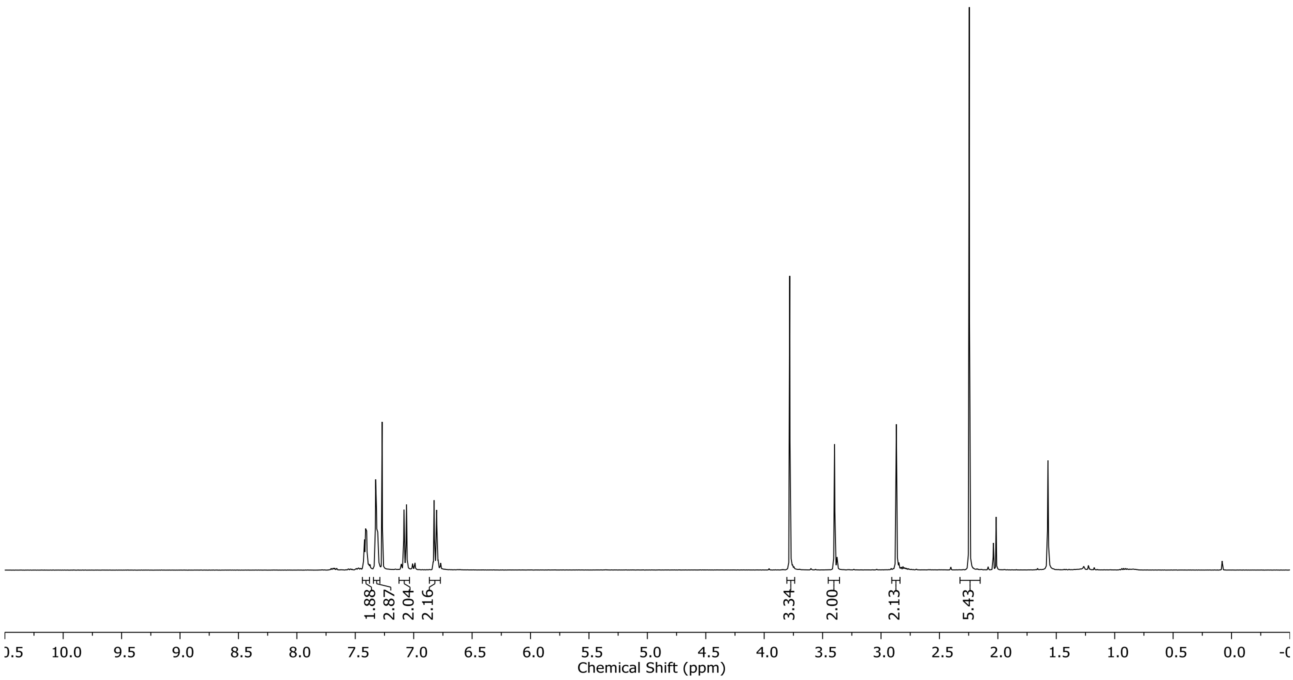
1.88
2.87
2.04
2.16
3.34
2.00
2.13
5.43
Chemical Shift (ppm)

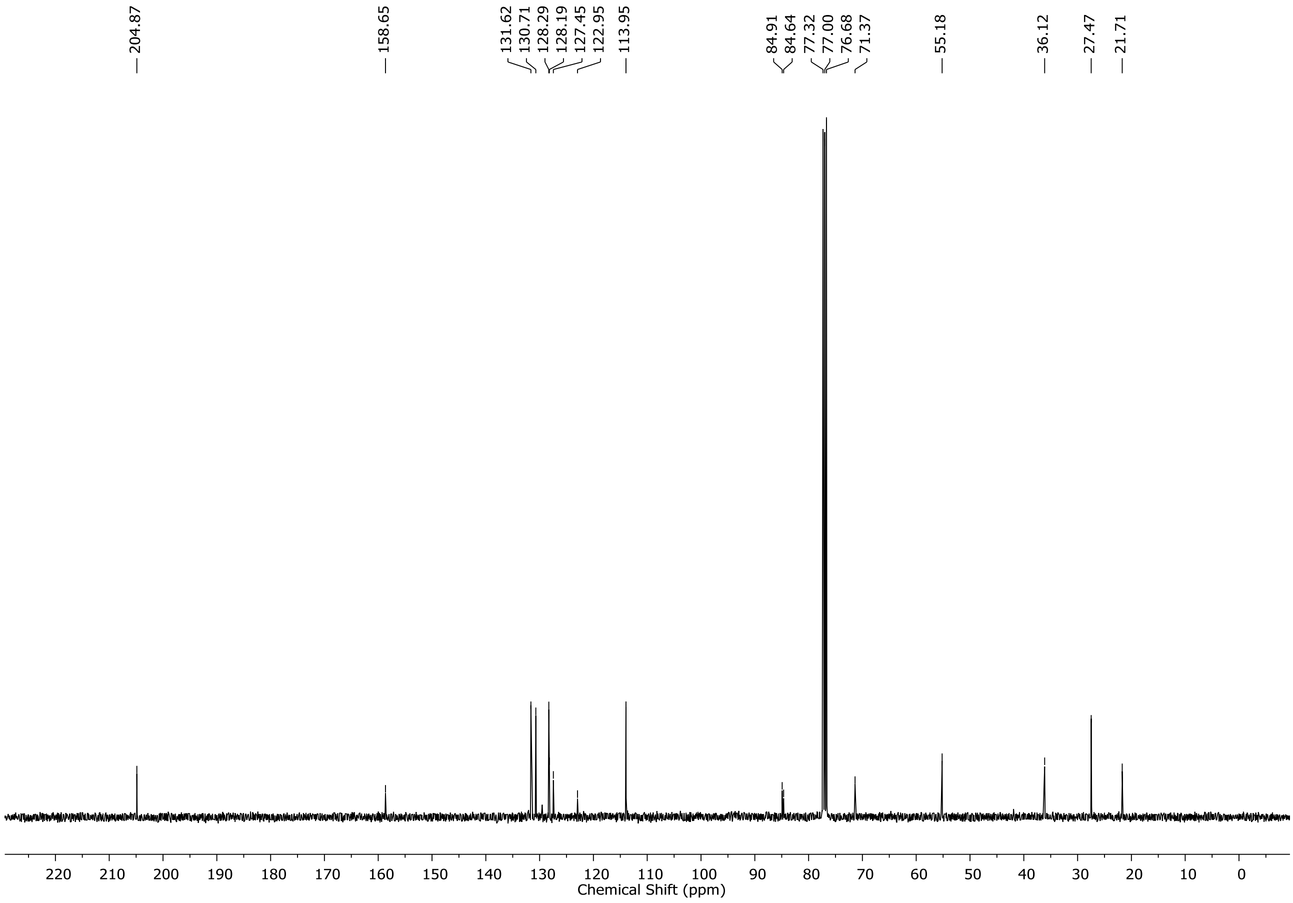
204.87
158.65
131.62
130.71
128.29
128.19
127.45
122.95
113.95
84.91
84.64
77.32
77.00
76.68
71.37
55.18
36.12
27.47
21.71
Chemical Shift (ppm)

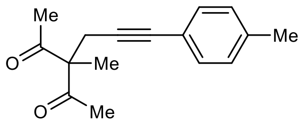
Me
O
Me
O
Me
Me

1f

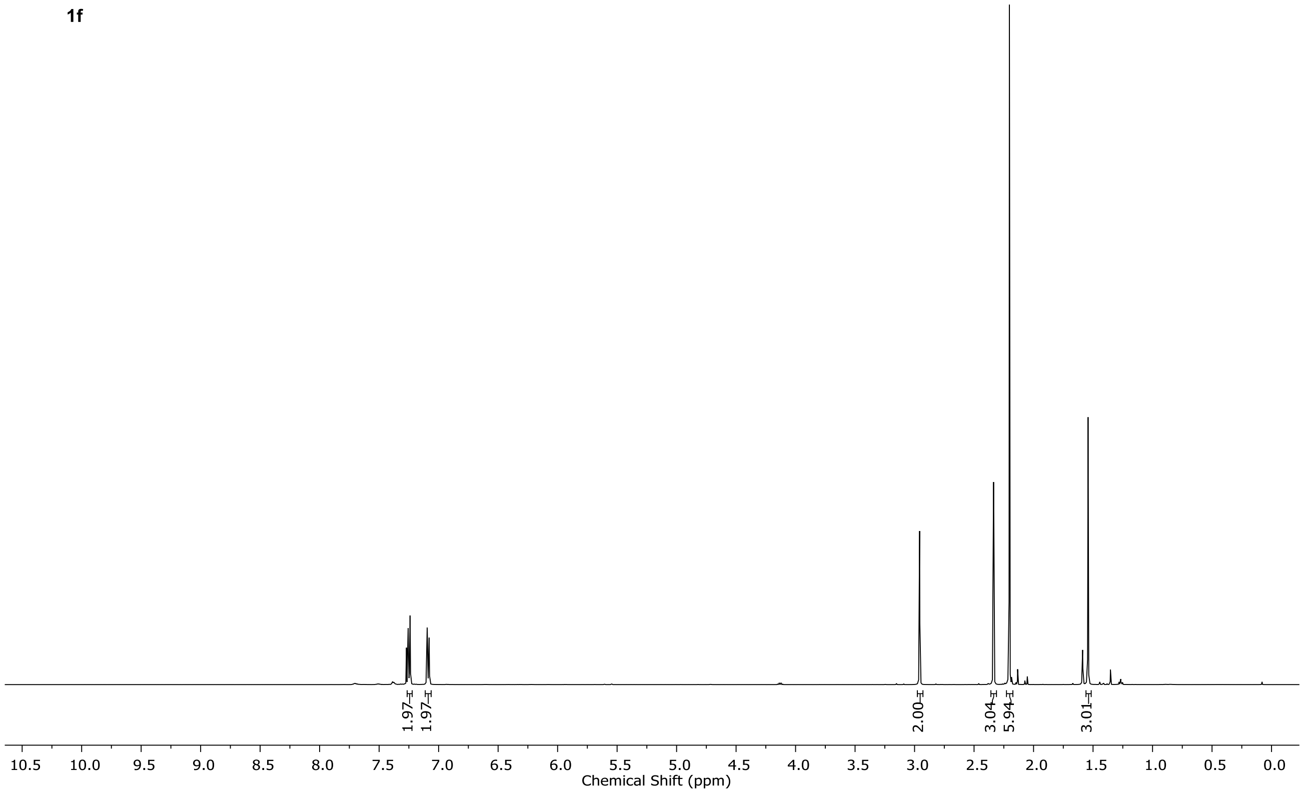
1.97
1.97
2.00
3.04
5.94
3.01
10.5 10.0 9.5 9.0 8.5 8.0 7.5 7.0 6.5 6.0 5.5 5.0 4.5 4.0 3.5 3.0 2.5 2.0 1.5 1.0 0.5 0.0
Chemical Shift (ppm)

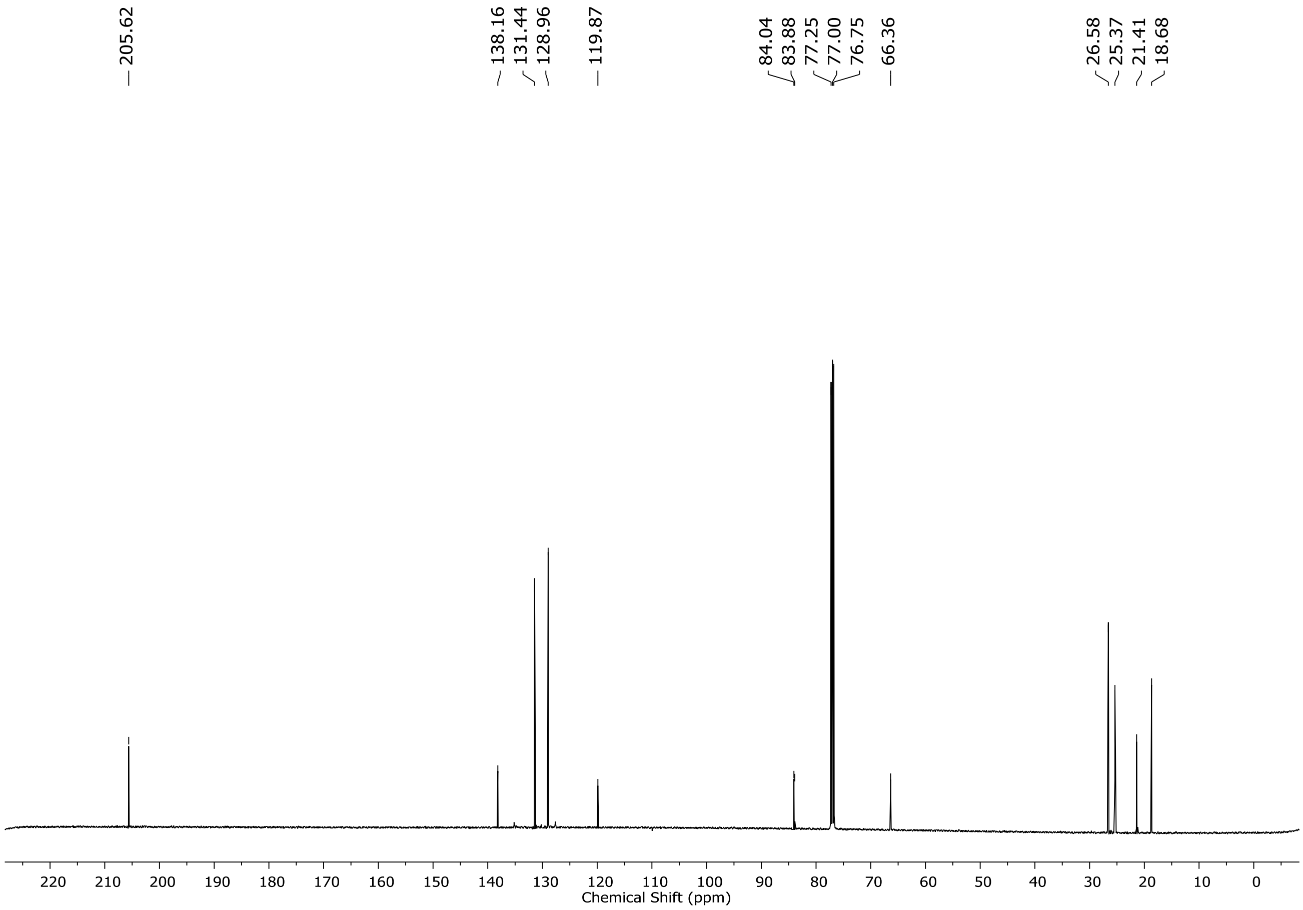
205.62
138.16
131.44
128.96
119.87
84.04
83.88
77.25
77.00
76.75
66.36
26.58
25.37
21.41
18.68
220 210 200 190 180 170 160 150 140 130 120 110 100 90 80 70 60 50 40 30 20 10 0
Chemical Shift (ppm)

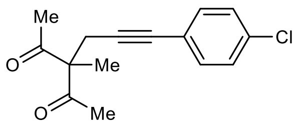
Me
O
Me
O
Me
Cl

1g

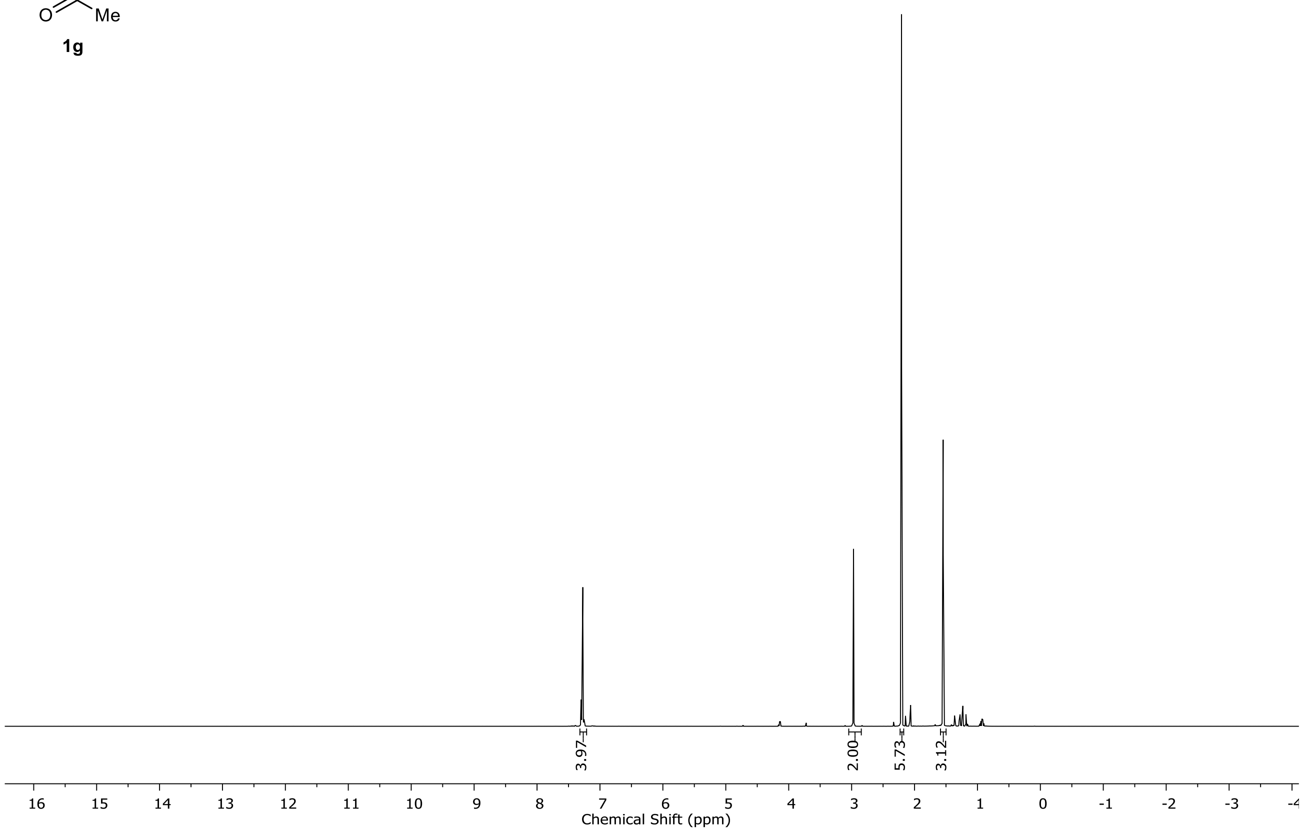
3.97
2.00
5.73
3.12
16
15
14
13
12
11
10
9
8
7
6
5
4
3
2
1
0
-1
-2
-3
Chemical Shift (ppm)

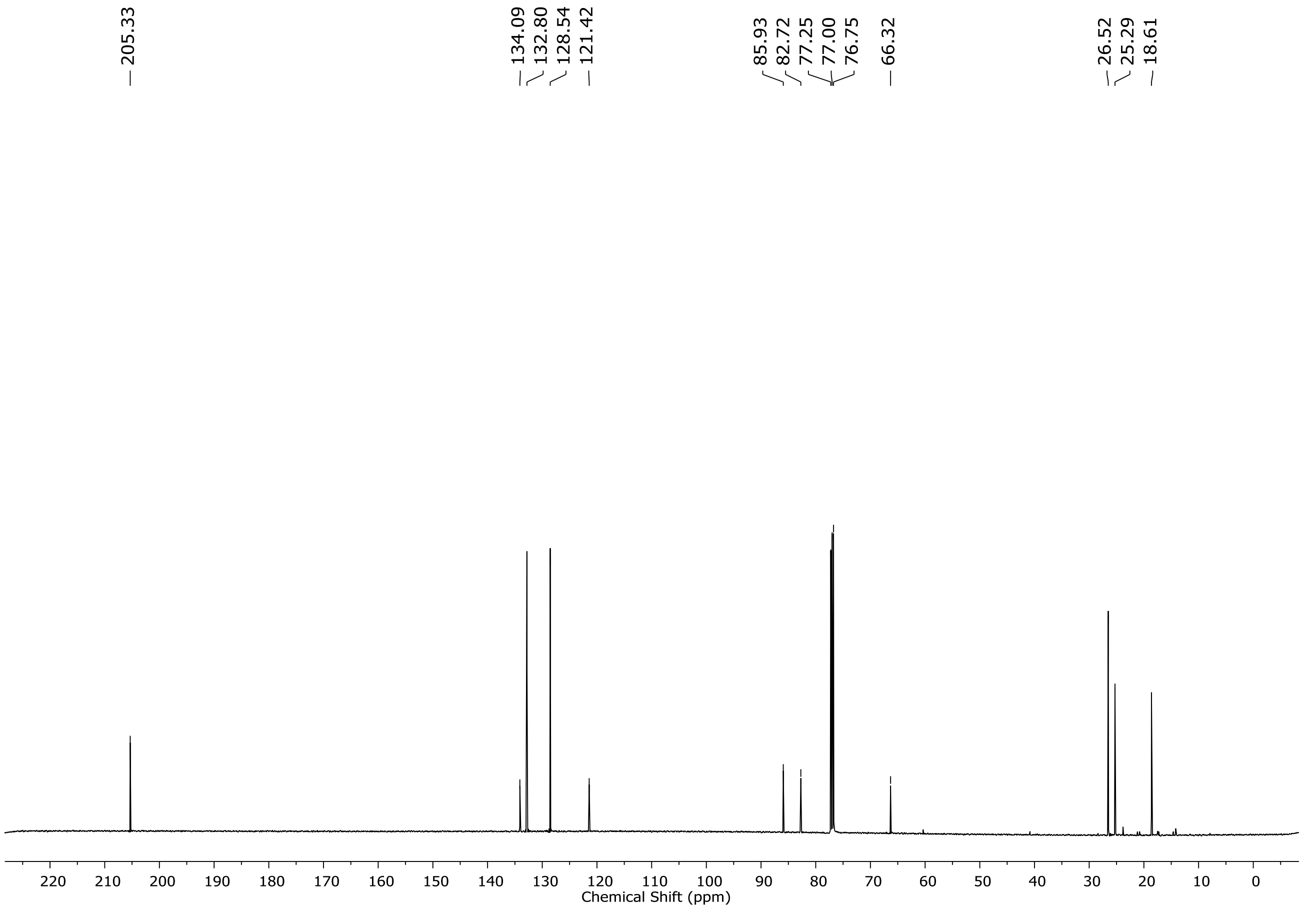
205.33
134.09
132.80
128.54
121.42
85.93
82.72
77.25
77.00
76.75
66.32
26.52
25.29
18.61
220
210
200
190
180
170
160
150
140
130
120
110
100
90
80
70
60
50
40
30
20
10
0
Chemical Shift (ppm)

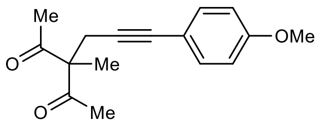
Me
O
Me
O
Me
OMe

1h

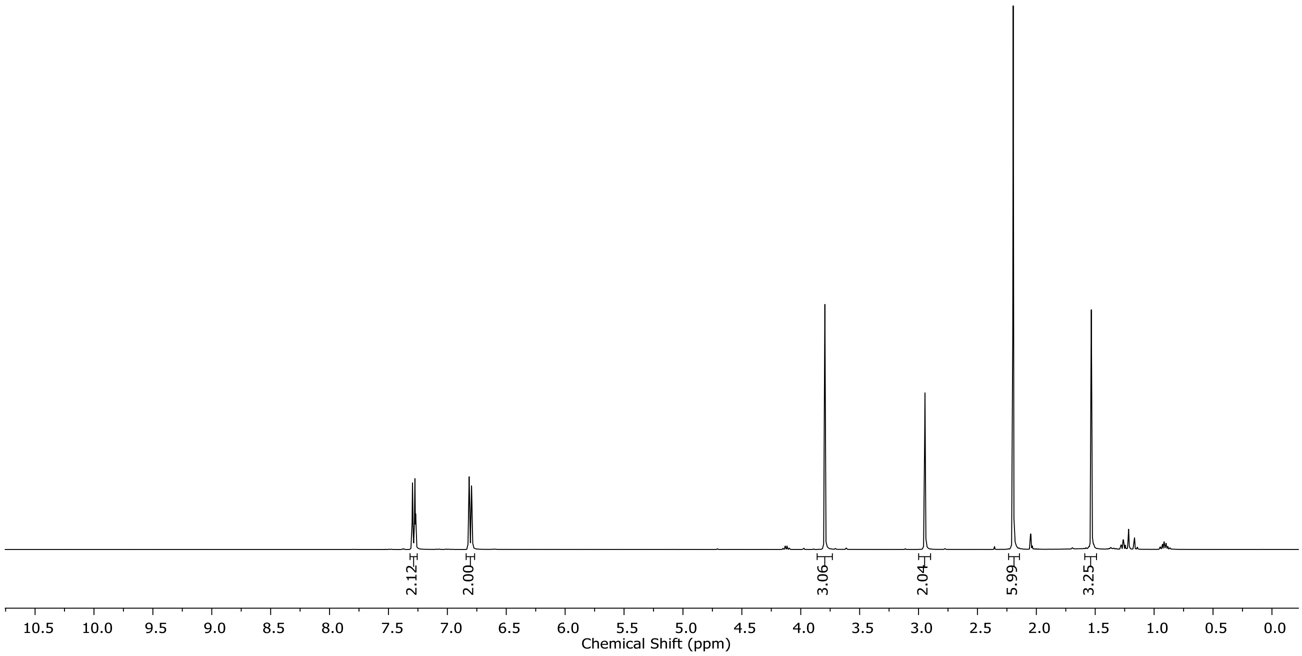
2.12
2.00
3.06
2.04
5.99
3.25
10.5 10.0 9.5 9.0 8.5 8.0 7.5 7.0 6.5 6.0 5.5 5.0 4.5 4.0 3.5 3.0 2.5 2.0 1.5 1.0 0.5 0.0
Chemical Shift (ppm)

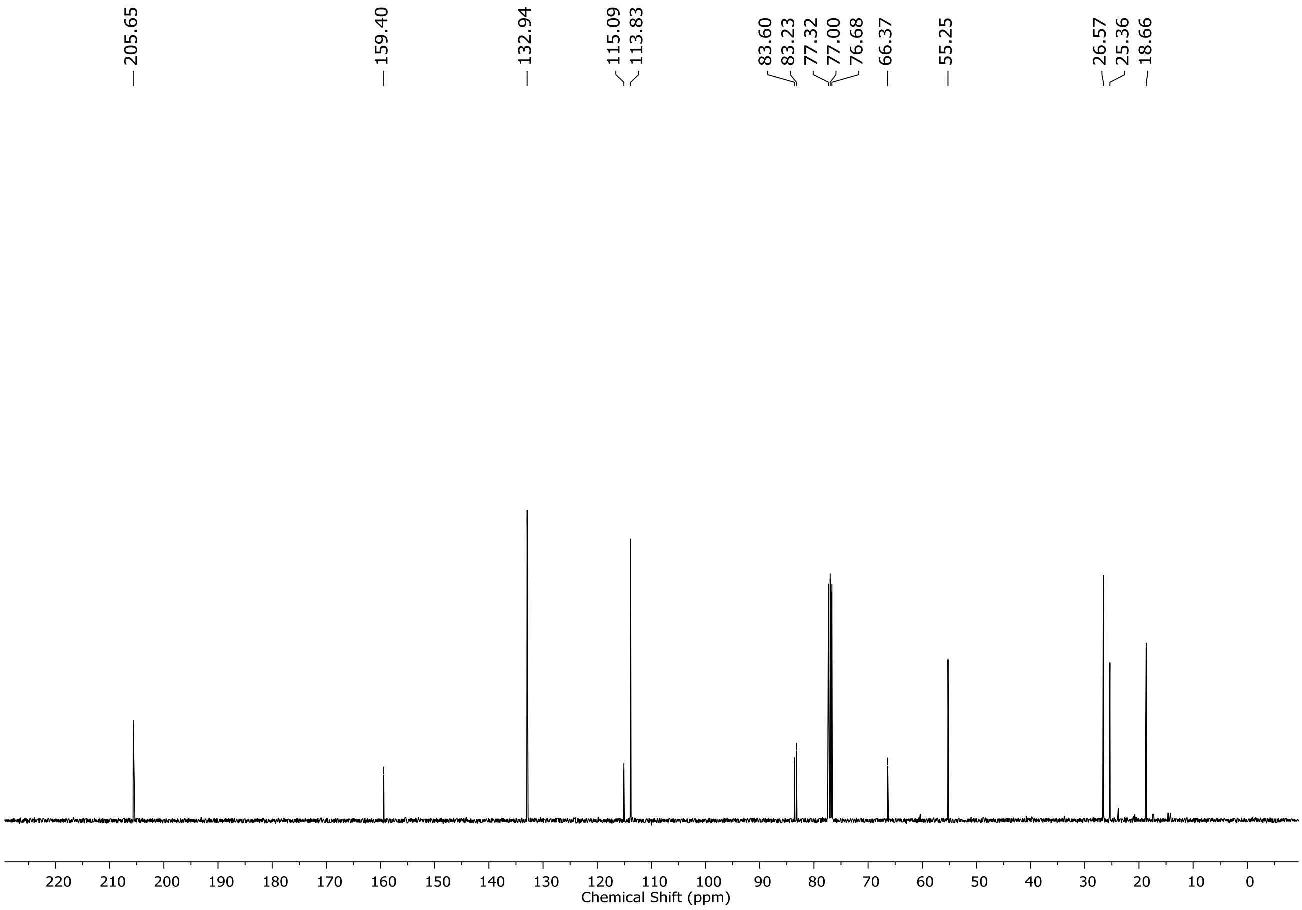
205.65
159.40
132.94
115.09
113.83
83.60
83.23
77.32
77.00
76.68
66.37
55.25
26.57
25.36
18.66
220 210 200 190 180 170 160 150 140 130 120 110 100 90 80 70 60 50 40 30 20 10 0
Chemical Shift (ppm)

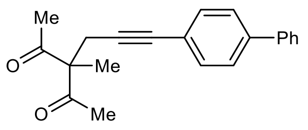
Me
O
Me
O
Me
Ph

1i

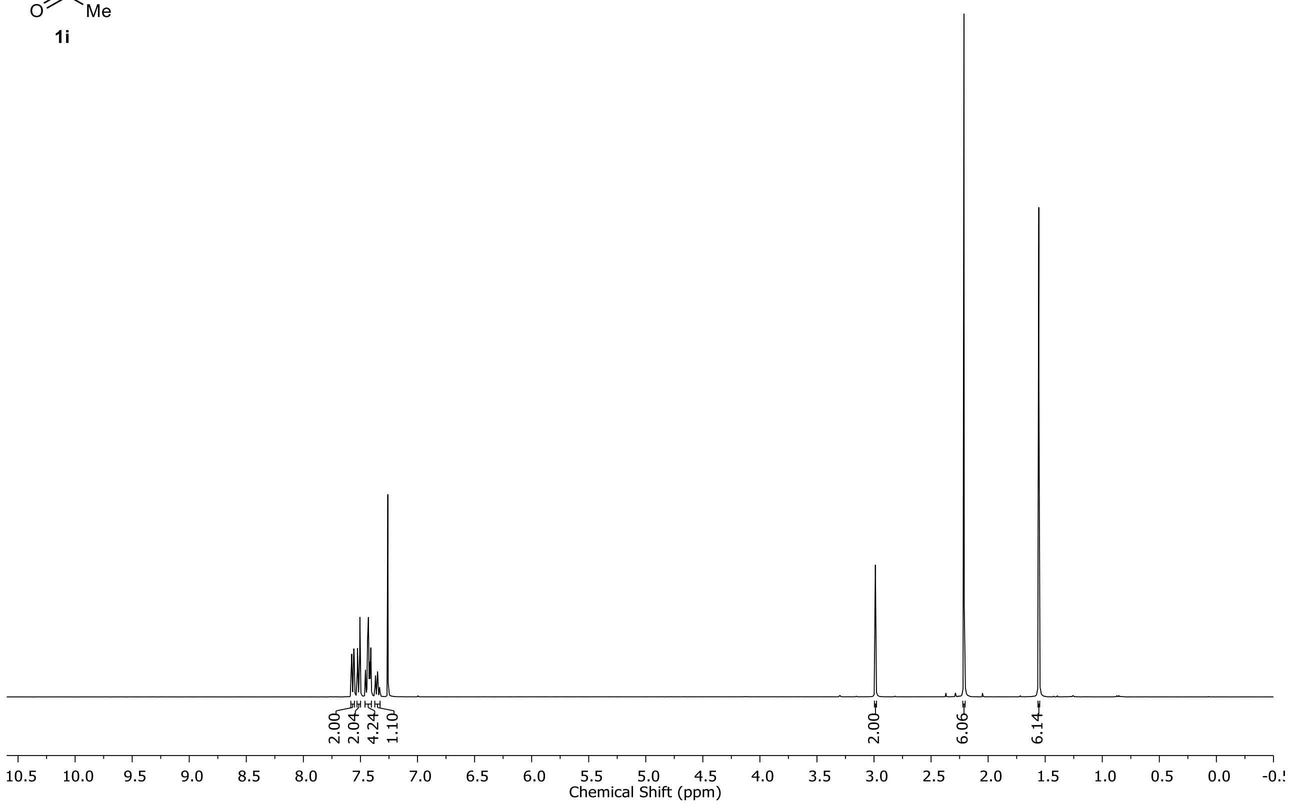
2.00
2.04
4.24
1.10
2.00
6.06
6.14
10.5 10.0 9.5 9.0 8.5 8.0 7.5 7.0 6.5 6.0 5.5 5.0 4.5 4.0 3.5 3.0 2.5 2.0 1.5 1.0 0.5 0.0
Chemical Shift (ppm)

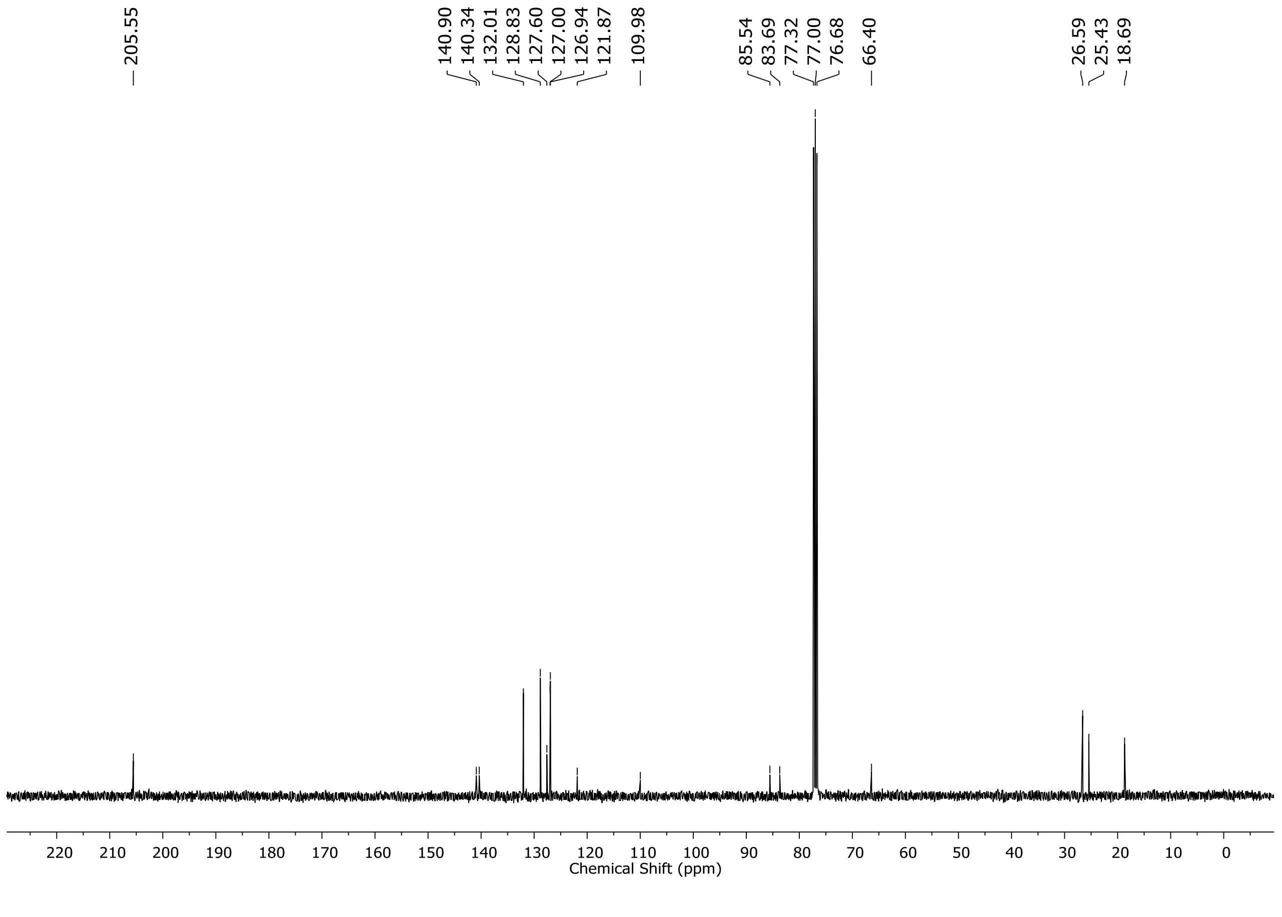
205.55
140.90
140.34
132.01
128.83
127.60
127.00
126.94
121.87
109.98
85.54
83.69
77.32
77.00
76.68
66.40
26.59
25.43
18.69
220 210 200 190 180 170 160 150 140 130 120 110 100 90 80 70 60 50 40 30 20 10 0
Chemical Shift (ppm)

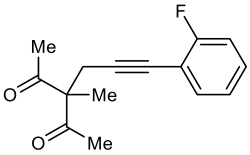
F
Me
O
Me
O
Me

1j

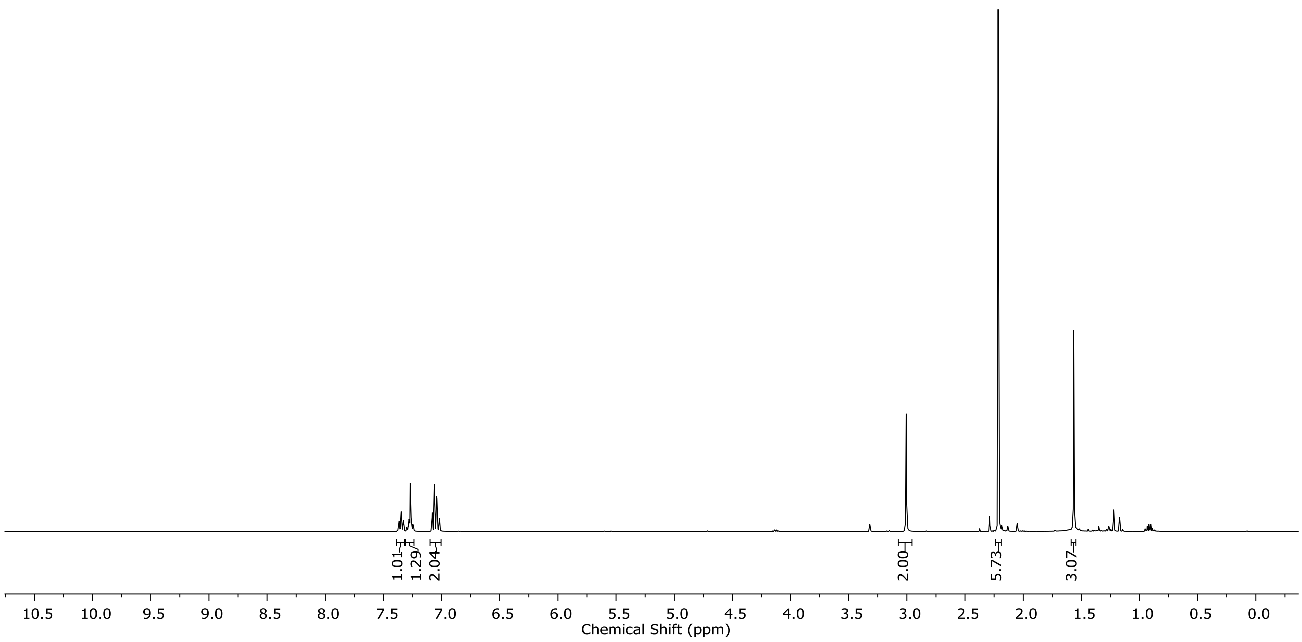
1.01
1.29
2.04
2.00
5.73
3.07
10.5 10.0 9.5 9.0 8.5 8.0 7.5 7.0 6.5 6.0 5.5 5.0 4.5 4.0 3.5 3.0 2.5 2.0 1.5 1.0 0.5 0.0
Chemical Shift (ppm)

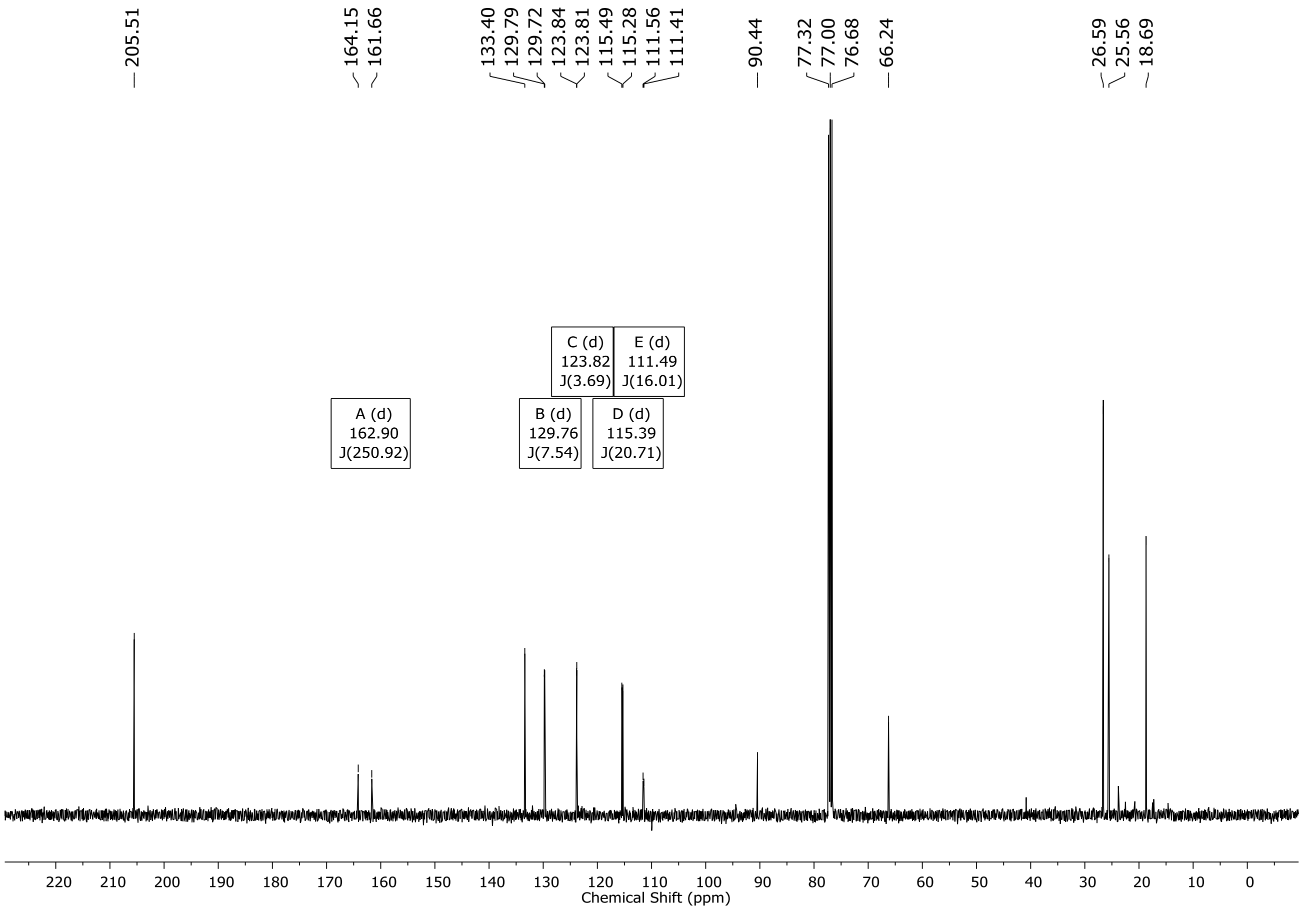
205.51
164.15
161.66
133.40
129.79
129.72
123.84
123.81
115.49
115.28
111.56
111.41
90.44
77.32
77.00
76.68
66.24
26.59
25.56
18.69
C (d) 123.82 J(3.69)
E (d) 111.49 J(16.01)
A (d) 162.90 J(250.92)
B (d) 129.76 J(7.54)
D (d) 115.39 J(20.71)
220 210 200 190 180 170 160 150 140 130 120 110 100 90 80 70 60 50 40 30 20 10 0
Chemical Shift (ppm)

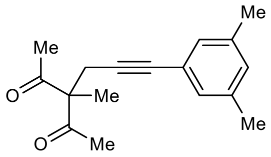
Me
Me
O
Me
O
Me
Me

1k

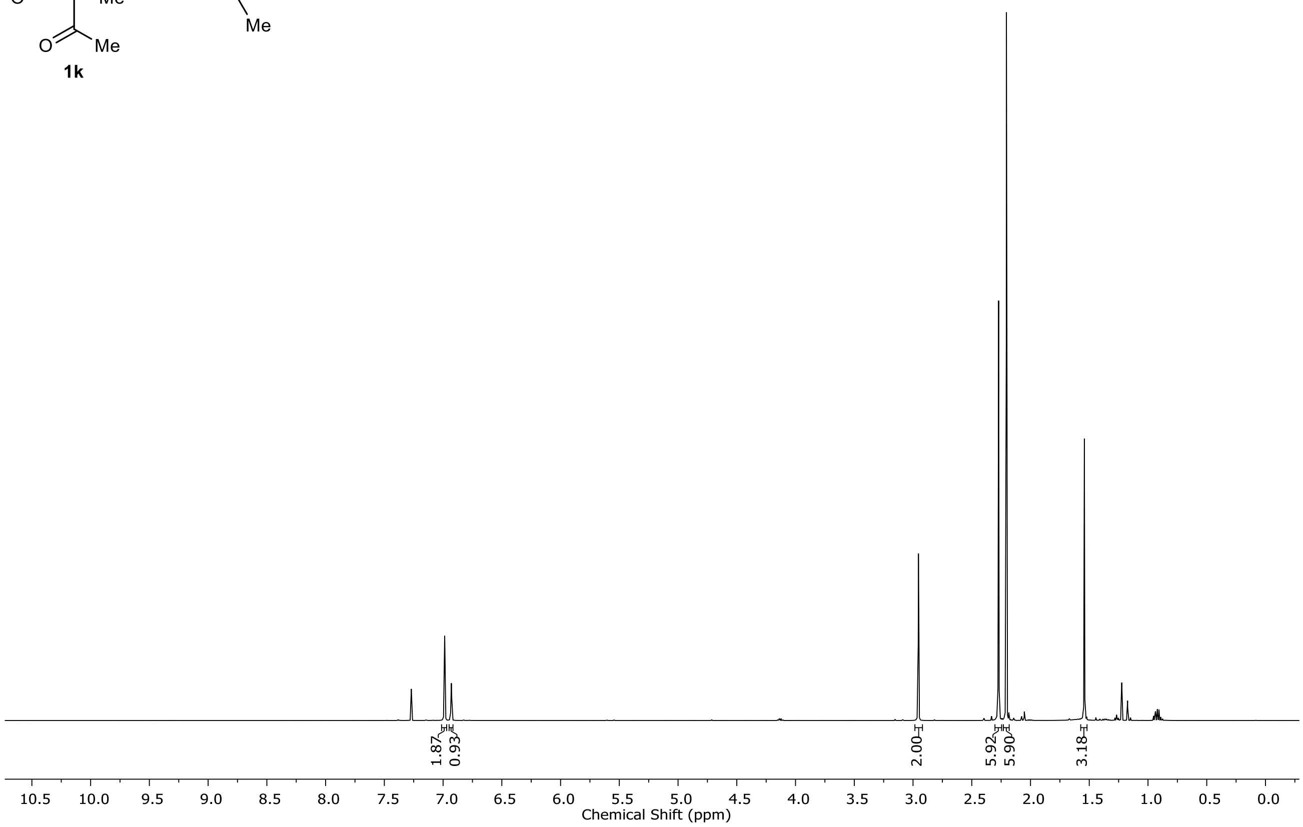
1.87
0.93
2.00
5.92
5.90
3.18
Chemical Shift (ppm)

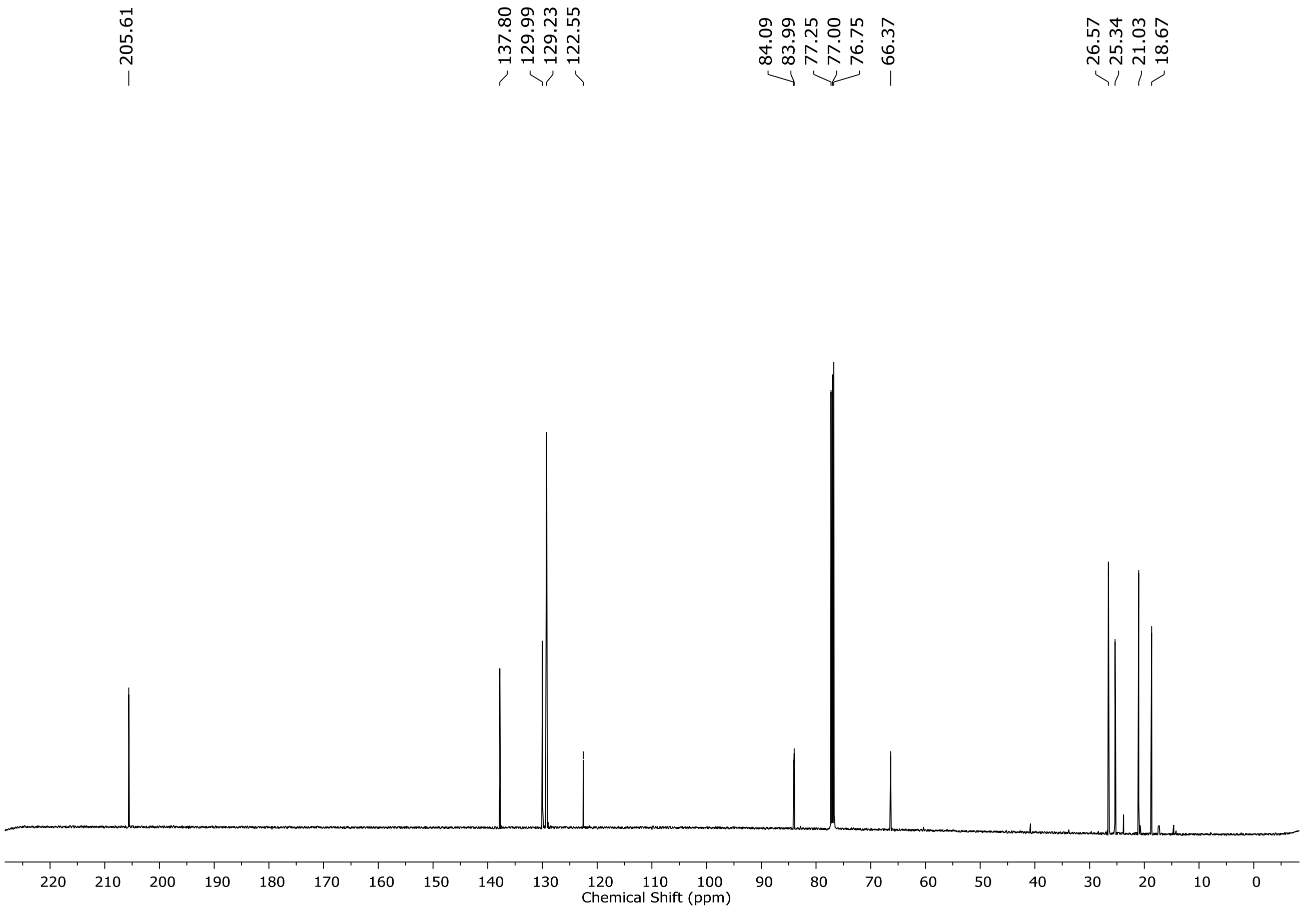
205.61
137.80
129.99
129.23
122.55
84.09
83.99
77.25
77.00
76.75
66.37
26.57
25.34
21.03
18.67
Chemical Shift (ppm)

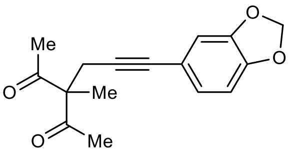
Me
O
Me
O
Me
O
O

1l

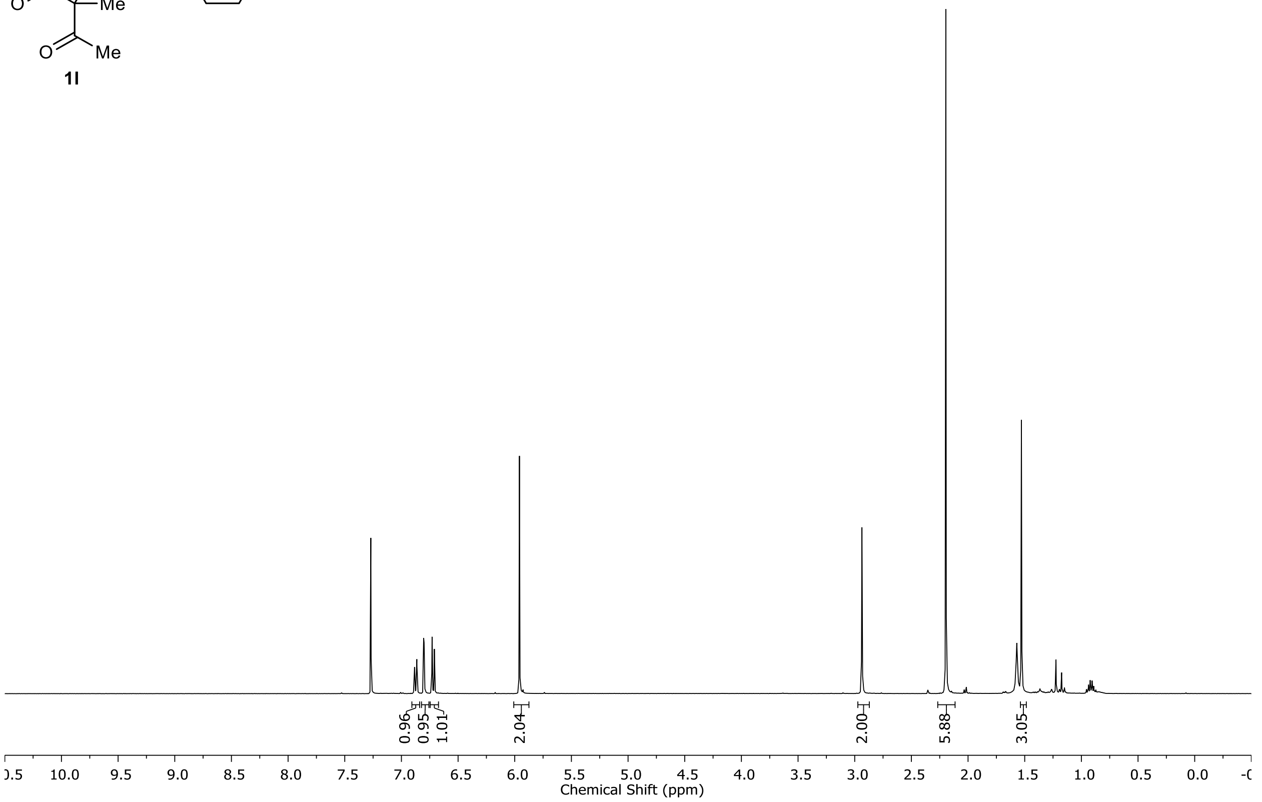
0.96
0.95
1.01
2.04
2.00
5.88
3.05
10.0 9.5 9.0 8.5 8.0 7.5 7.0 6.5 6.0 5.5 5.0 4.5 4.0 3.5 3.0 2.5 2.0 1.5 1.0 0.5 0.0
Chemical Shift (ppm)

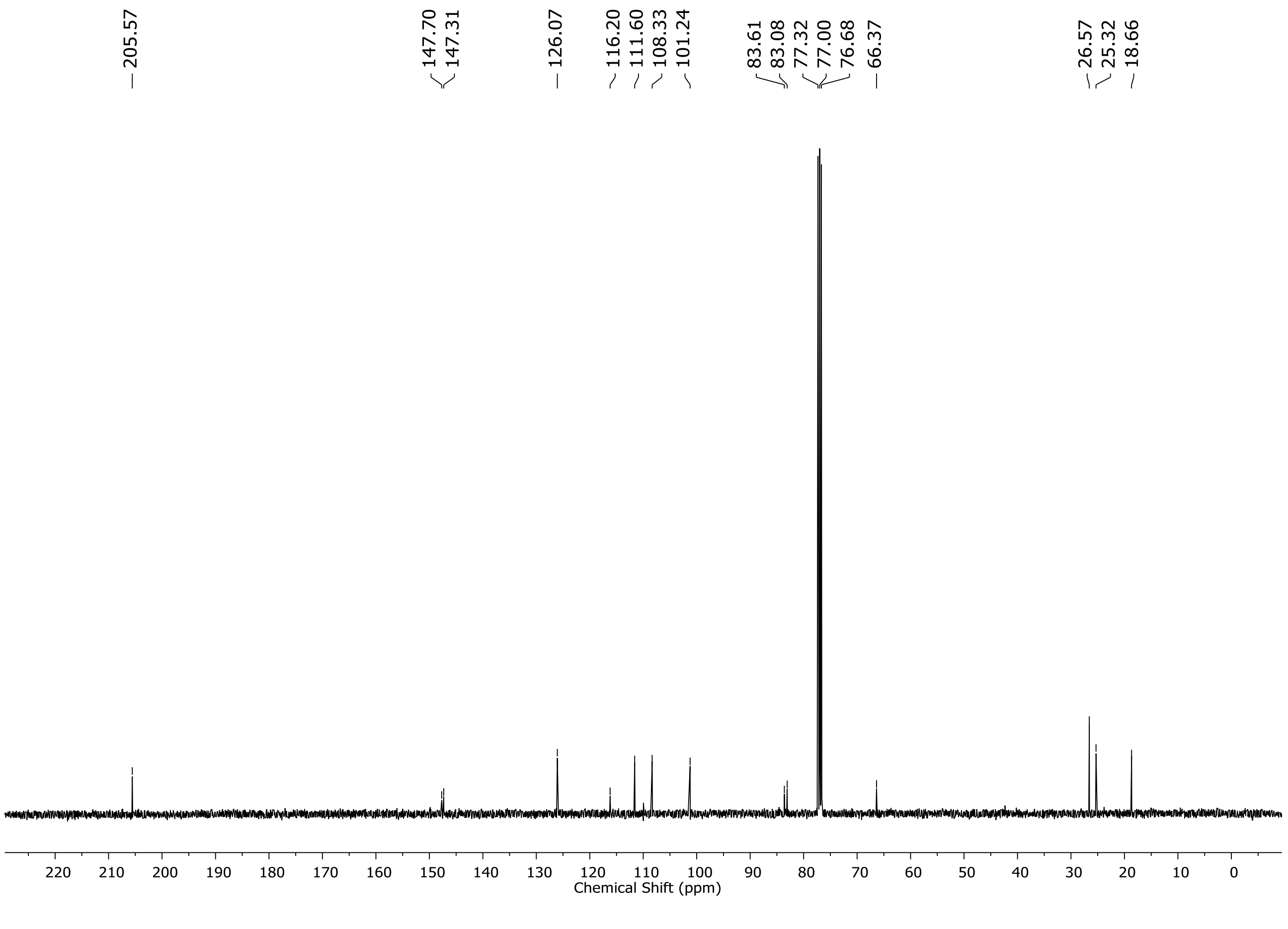
205.57
147.70
147.31
126.07
116.20
111.60
108.33
101.24
83.61
83.08
77.32
77.00
76.68
66.37
26.57
25.32
18.66
220 210 200 190 180 170 160 150 140 130 120 110 100 90 80 70 60 50 40 30 20 10 0
Chemical Shift (ppm)

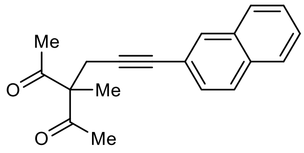
Me
O
Me
O
Me

1m

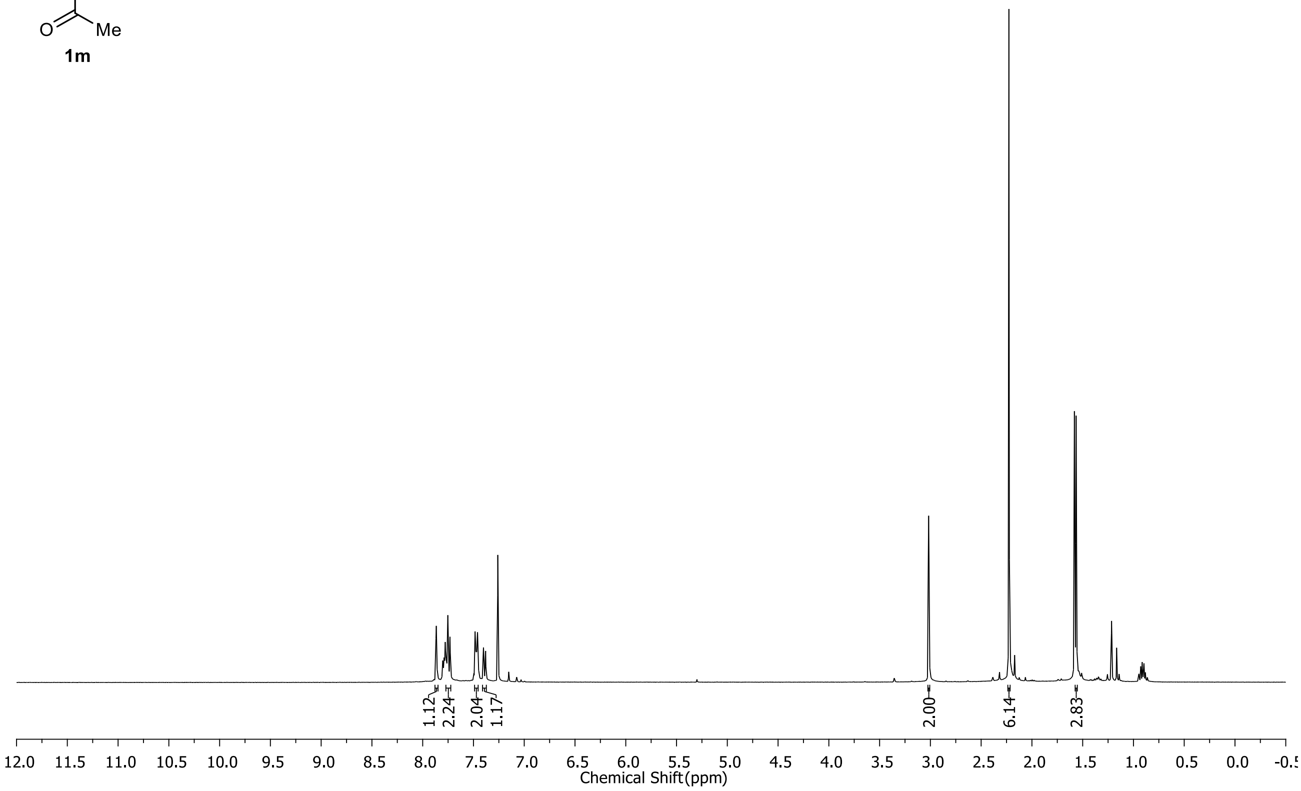
1.12
2.24
2.04
1.17
2.00
6.14
2.83
12.0 11.5 11.0 10.5 10.0 9.5 9.0 8.5 8.0 7.5 7.0 6.5 6.0 5.5 5.0 4.5 4.0 3.5 3.0 2.5 2.0 1.5 1.0 0.5 0.0
Chemical Shift(ppm)

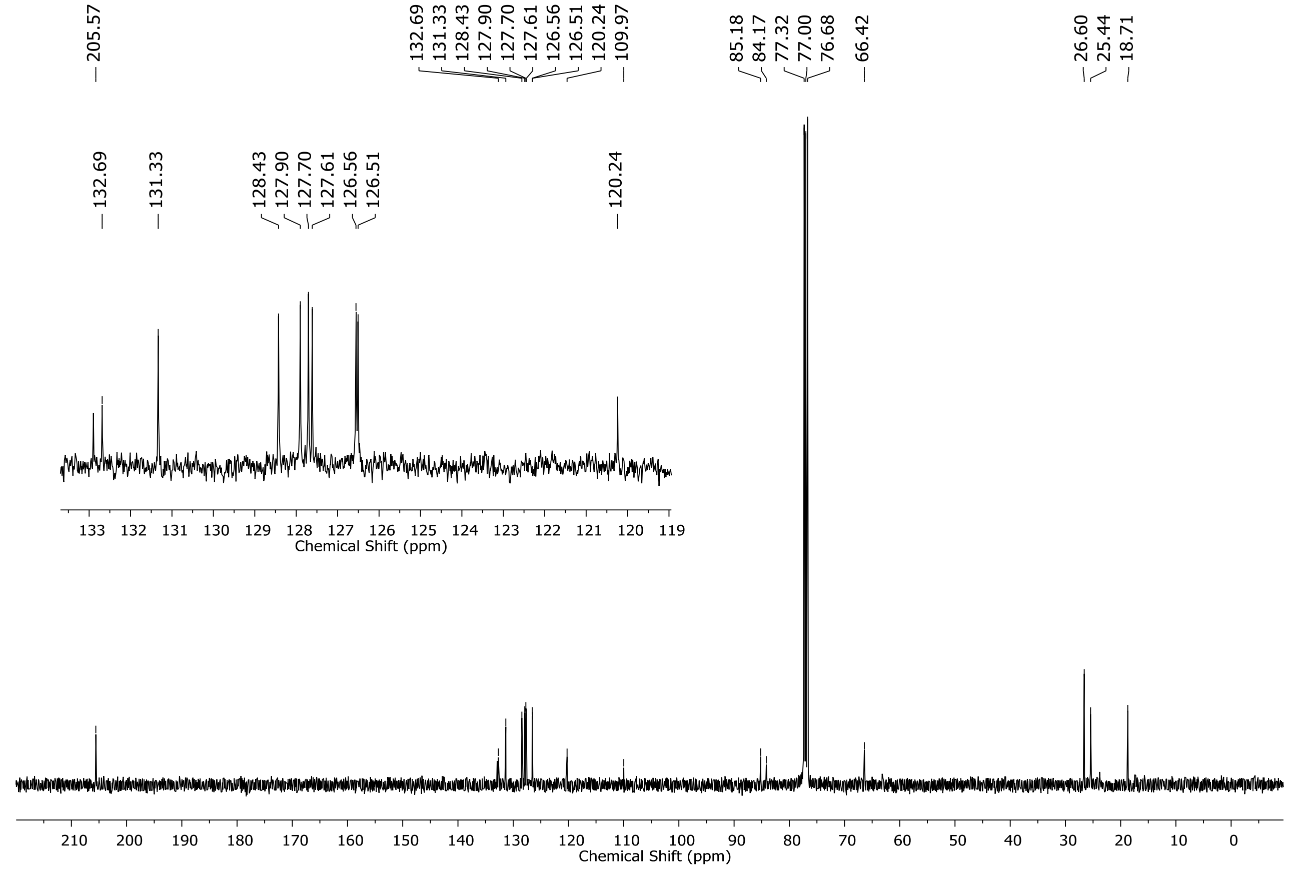
205.57
132.69
131.33
128.43
127.90
127.70
127.61
126.56
126.51
120.24
109.97
85.18
84.17
77.32
77.00
76.68
66.42
26.60
25.44
18.71
133 132 131 130 129 128 127 126 125 124 123 122 121 120 119
Chemical Shift (ppm)
210 200 190 180 170 160 150 140 130 120 110 100 90 80 70 60 50 40 30 20 10 0
Chemical Shift (ppm)

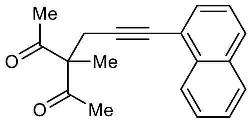
Me
O
Me
O
Me

1n

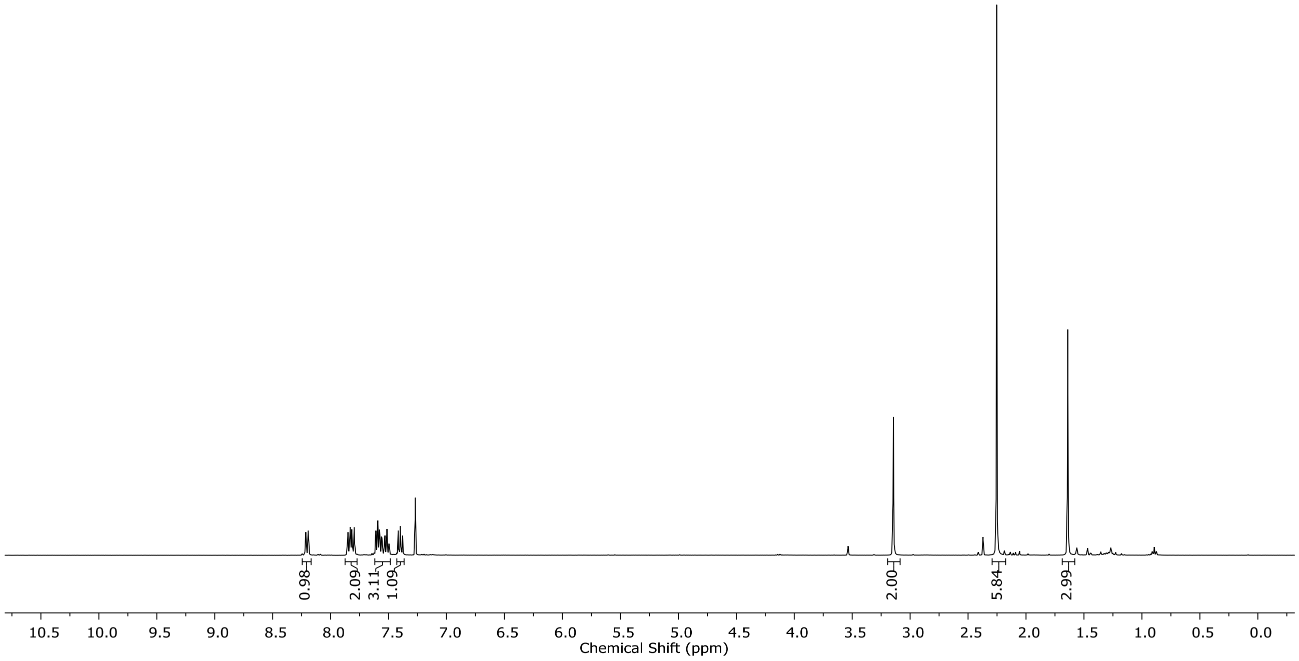
0.98
2.09
3.11
1.09
2.00
5.84
2.99
10.5 10.0 9.5 9.0 8.5 8.0 7.5 7.0 6.5 6.0 5.5 5.0 4.5 4.0 3.5 3.0 2.5 2.0 1.5 1.0 0.5 0.0
Chemical Shift (ppm)

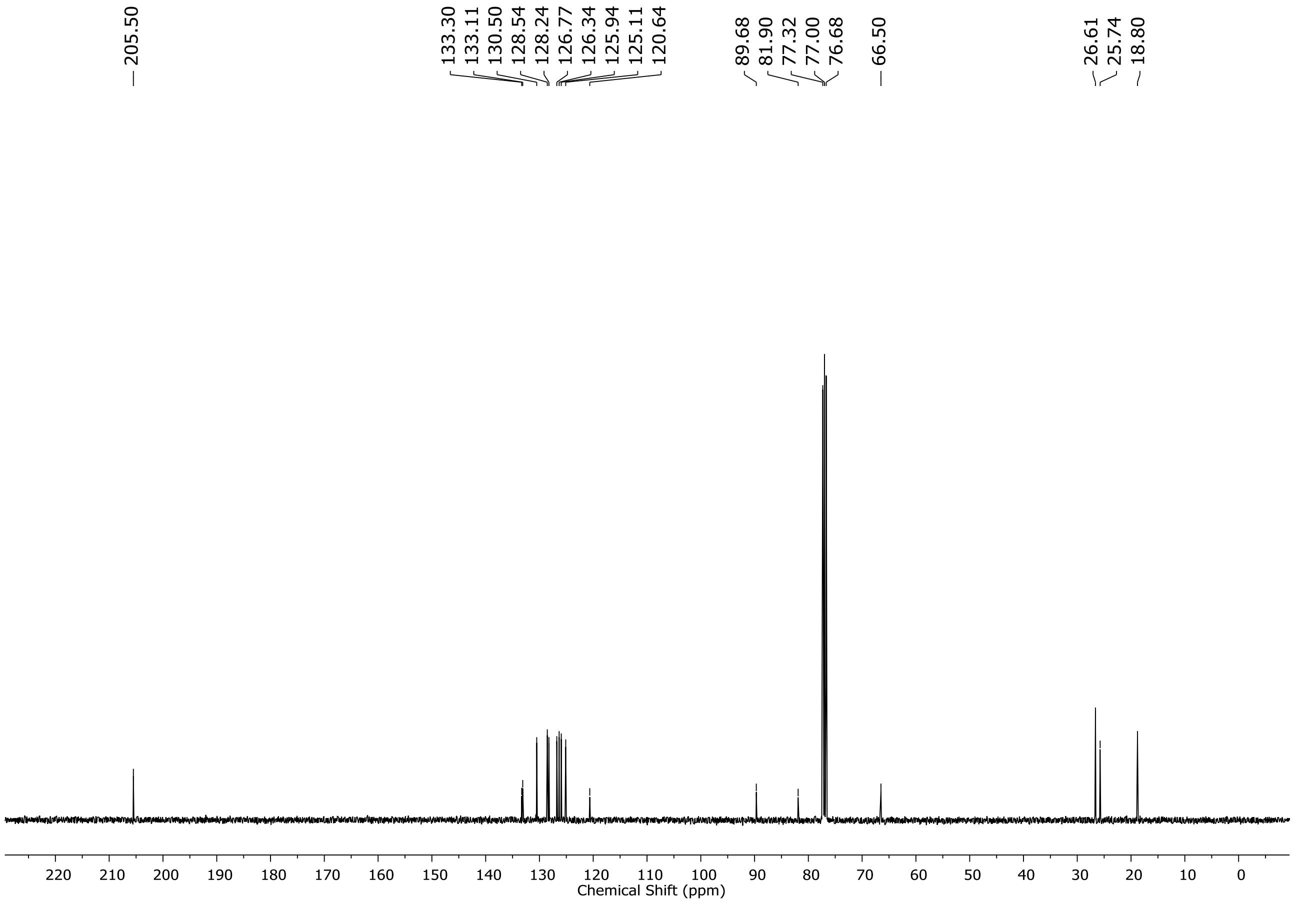
205.50
133.30
133.11
130.50
128.54
128.24
126.77
126.34
125.94
125.11
120.64
89.68
81.90
77.32
77.00
76.68
66.50
26.61
25.74
18.80
220 210 200 190 180 170 160 150 140 130 120 110 100 90 80 70 60 50 40 30 20 10 0
Chemical Shift (ppm)

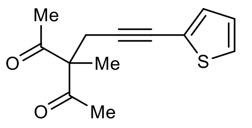
Me
O
Me
S
O
Me

1o

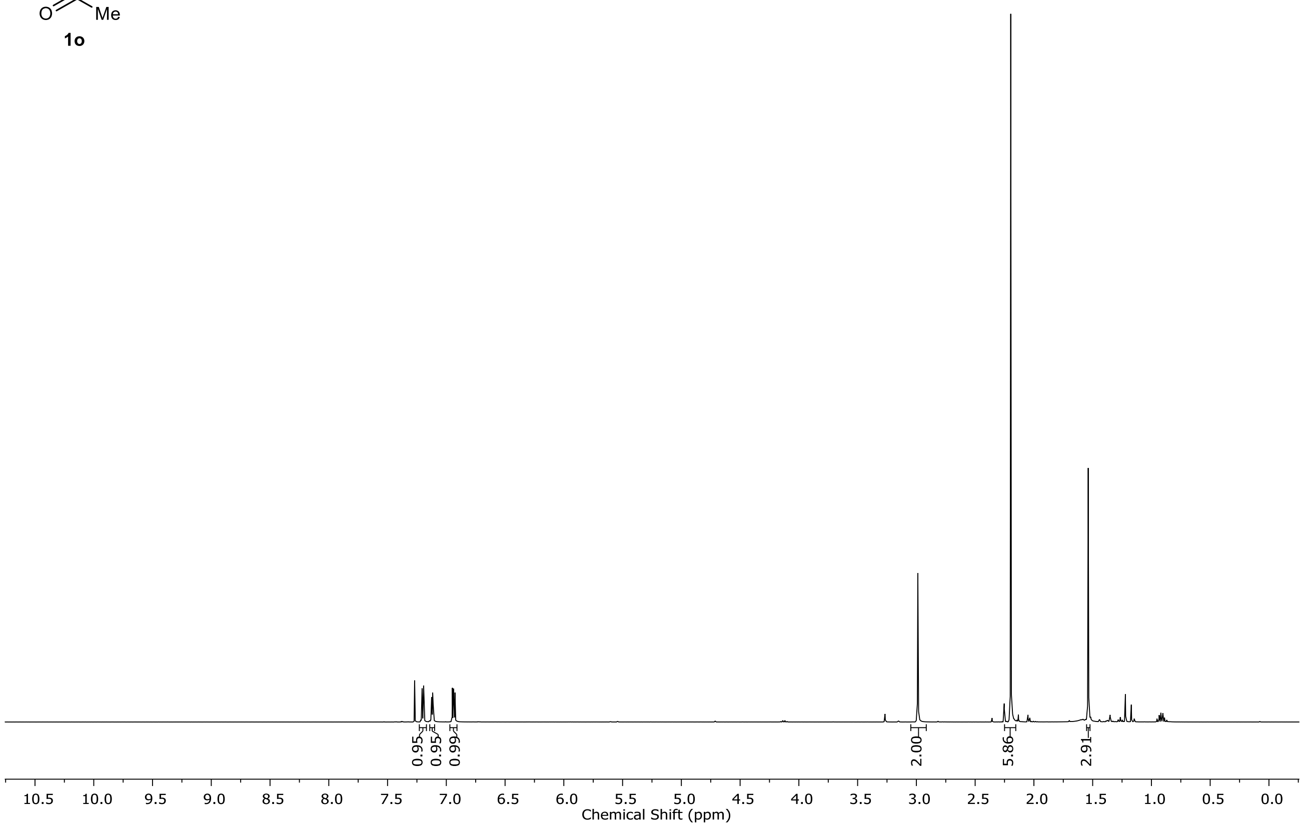
0.95
0.95
0.99
2.00
5.86
2.91
10.5 10.0 9.5 9.0 8.5 8.0 7.5 7.0 6.5 6.0 5.5 5.0 4.5 4.0 3.5 3.0 2.5 2.0 1.5 1.0 0.5 0.0
Chemical Shift (ppm)

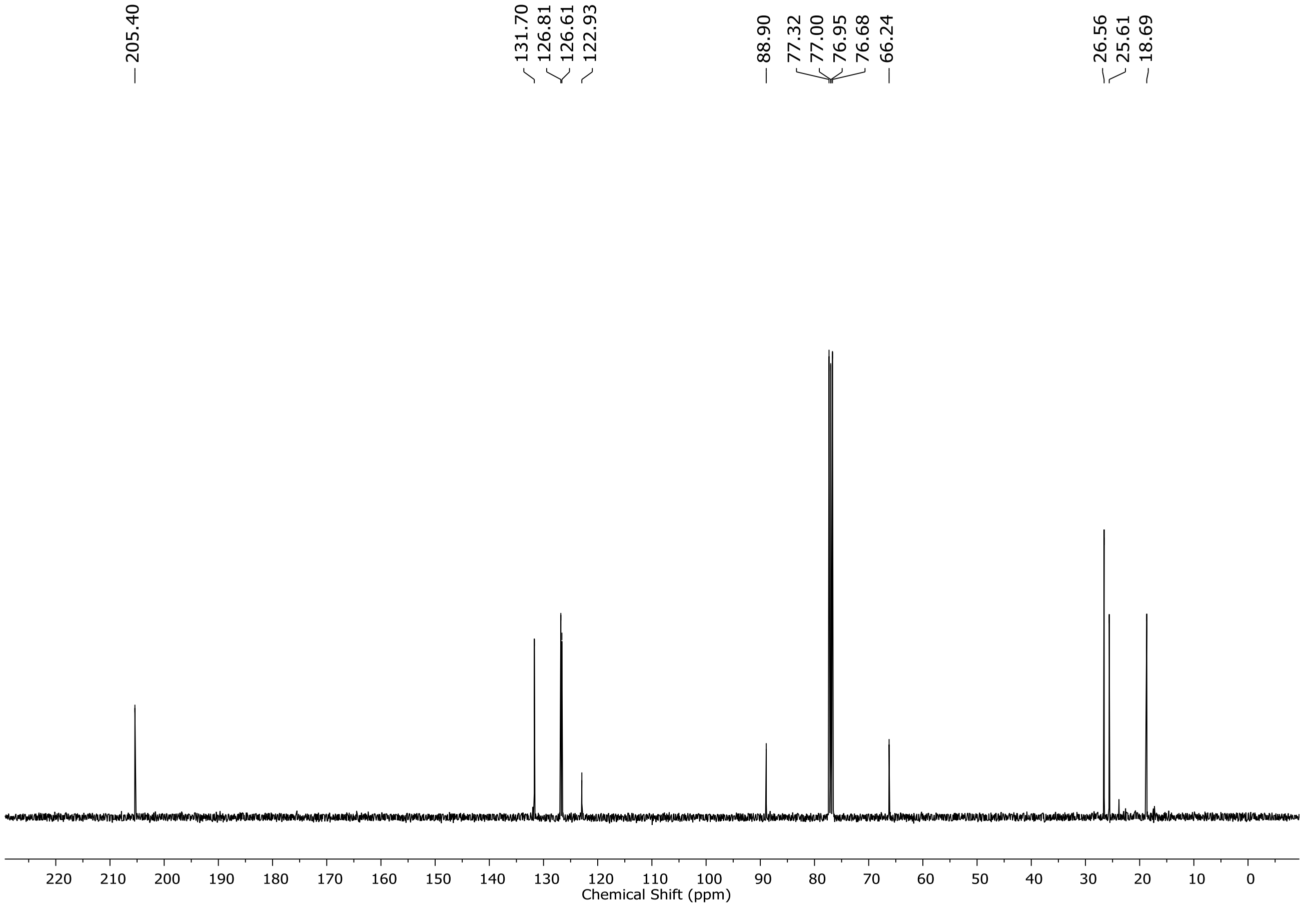
205.40
131.70
126.81
126.61
122.93
88.90
77.32
77.00
76.95
76.68
66.24
26.56
25.61
18.69
220 210 200 190 180 170 160 150 140 130 120 110 100 90 80 70 60 50 40 30 20 10 0
Chemical Shift (ppm)

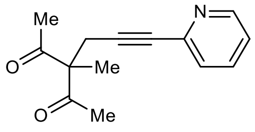
Me
O
Me
N
O
Me

1p

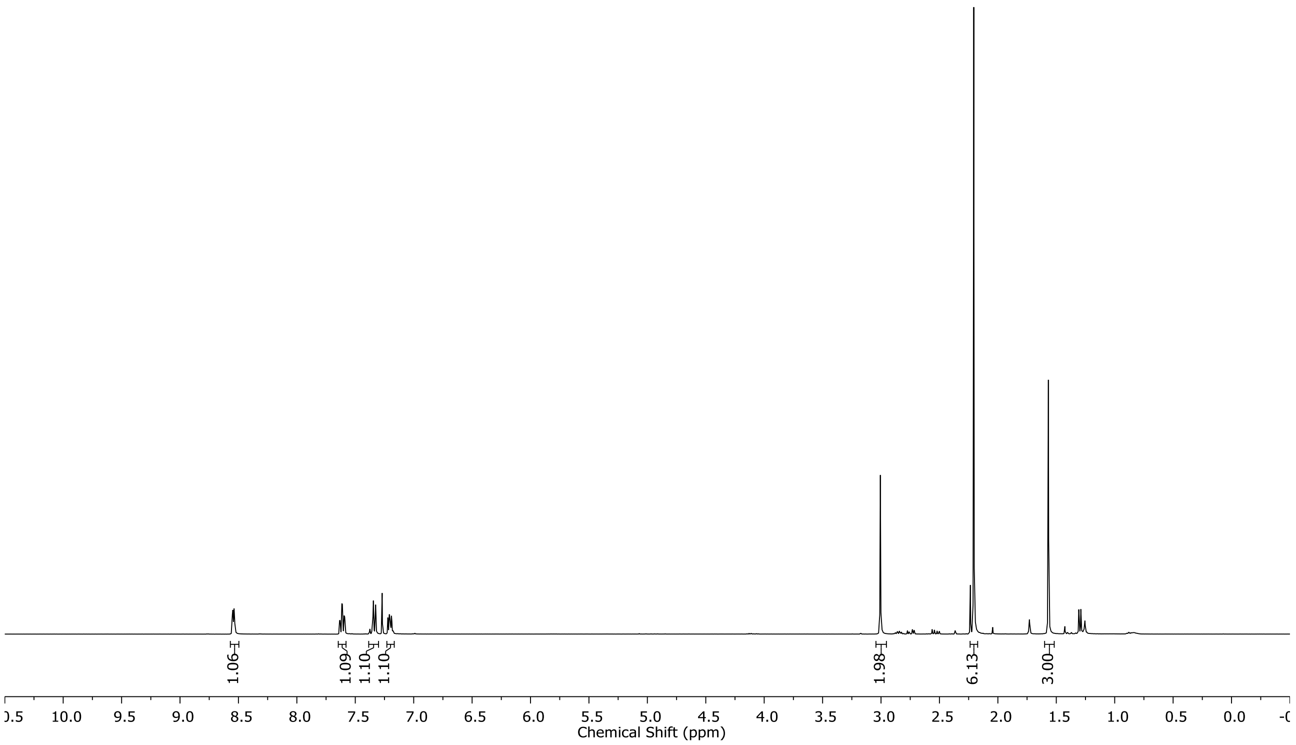
1.06
1.09
1.10
1.10
1.98
6.13
3.00
Chemical Shift (ppm)

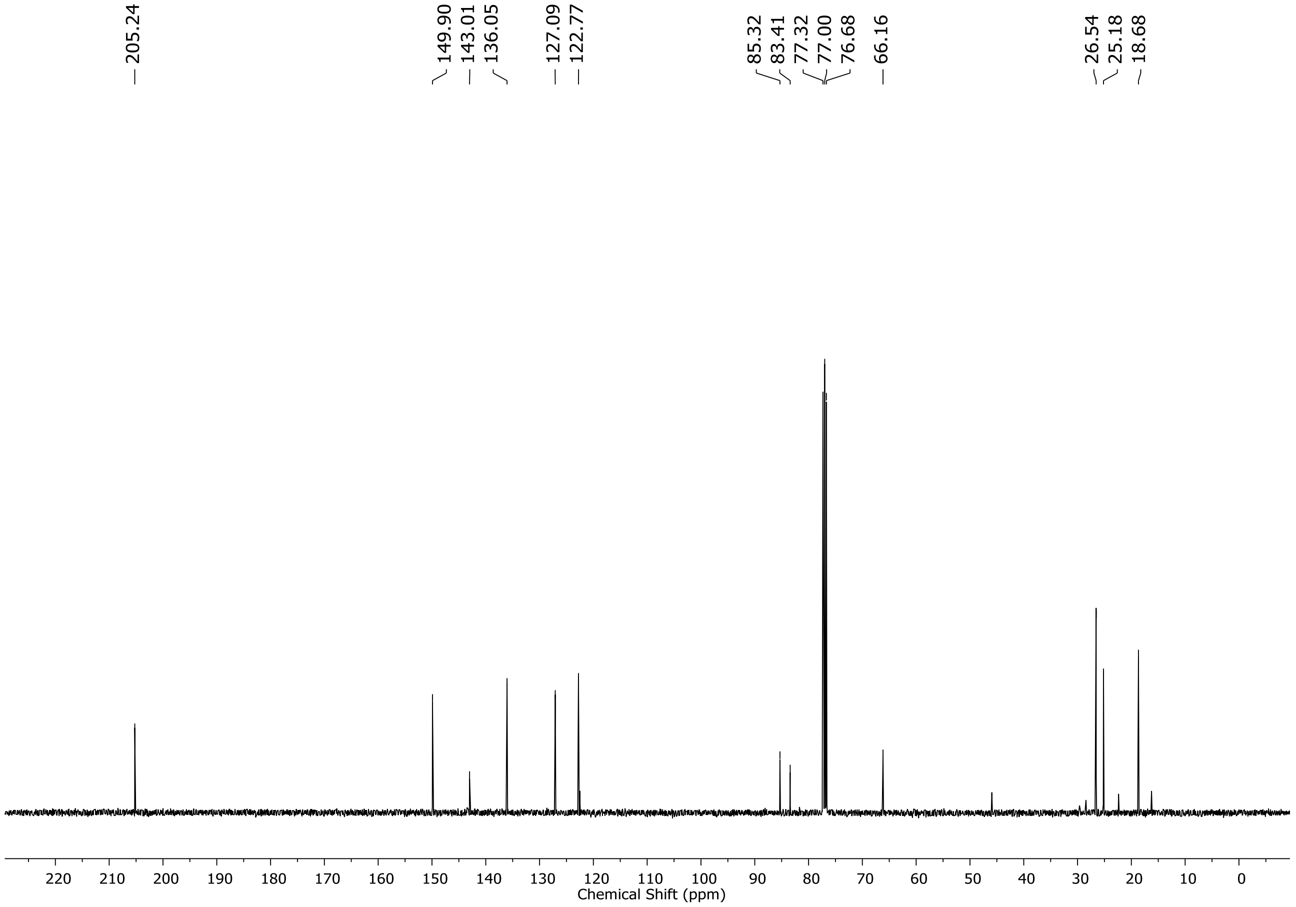
205.24
149.90
143.01
136.05
127.09
122.77
85.32
83.41
77.32
77.00
76.68
66.16
26.54
25.18
18.68
Chemical Shift (ppm)

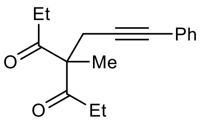
Et
Ph
O
Me
O
Et

1q

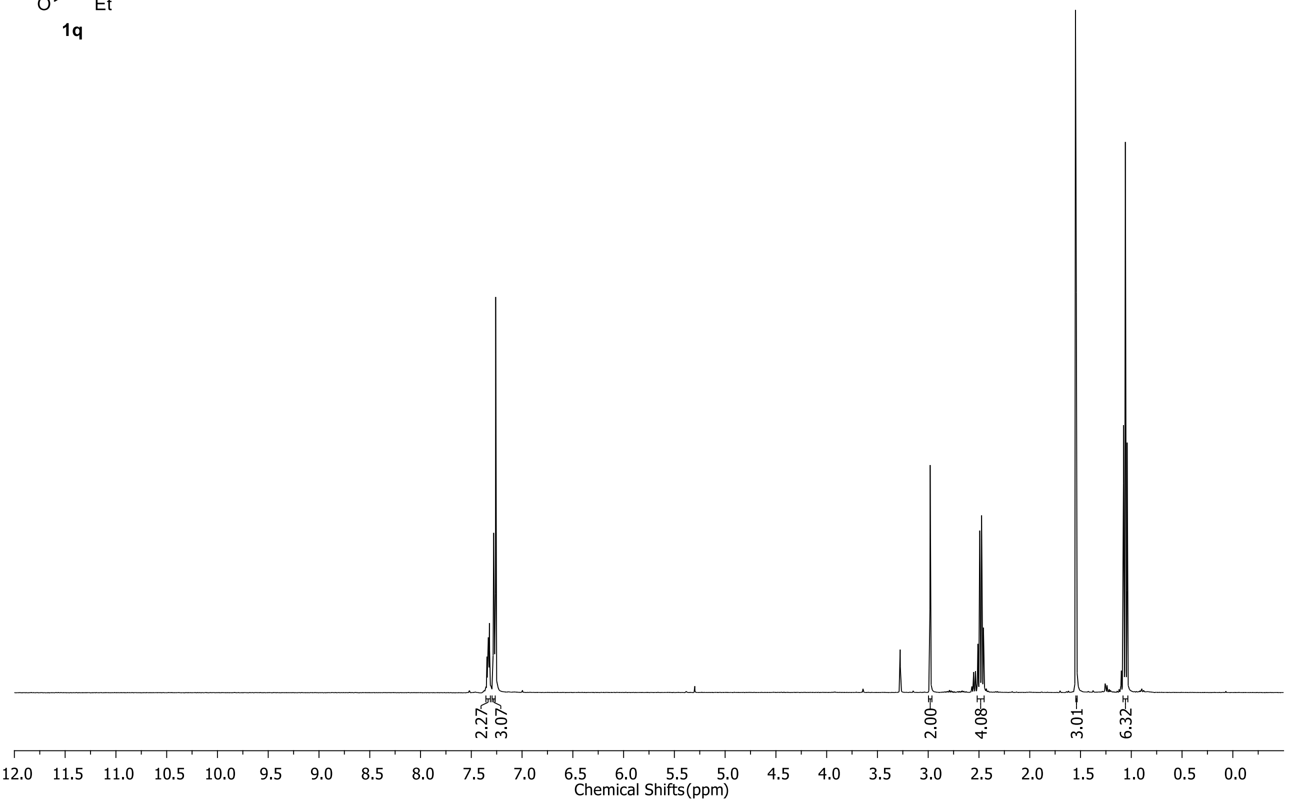
2.27
3.07
2.00
4.08
3.01
6.32
12.0 11.5 11.0 10.5 10.0 9.5 9.0 8.5 8.0 7.5 7.0 6.5 6.0 5.5 5.0 4.5 4.0 3.5 3.0 2.5 2.0 1.5 1.0 0.5 0.0
Chemical Shifts(ppm)

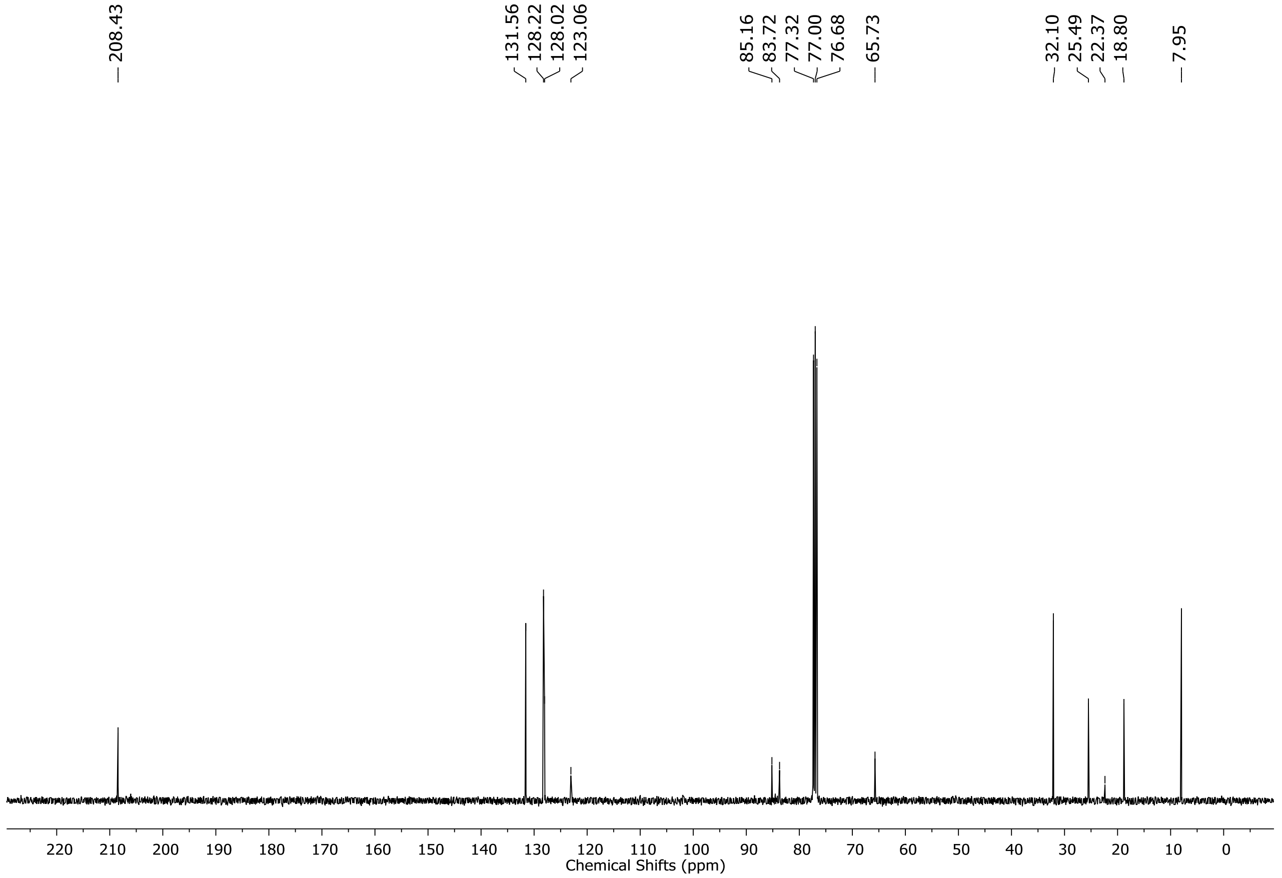
208.43
131.56
128.22
128.02
123.06
85.16
83.72
77.32
77.00
76.68
65.73
32.10
25.49
22.37
18.80
7.95
220 210 200 190 180 170 160 150 140 130 120 110 100 90 80 70 60 50 40 30 20 10 0
Chemical Shifts (ppm)

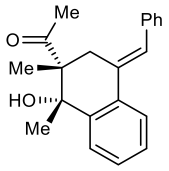
Me
Ph
O
Me
HO
Me

2a

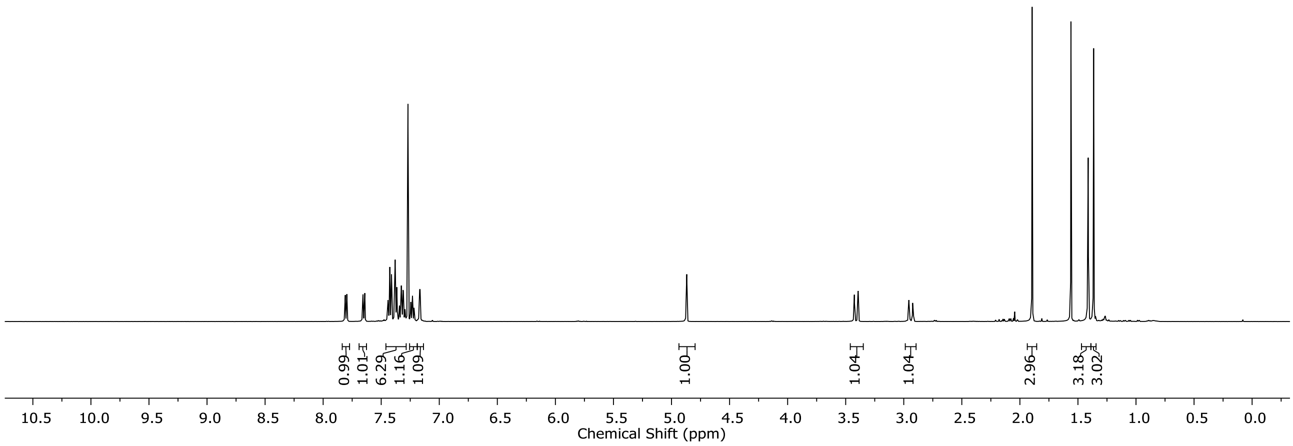
0.99
1.01
6.29
1.16
1.09
1.00
1.04
1.04
2.96
3.18
3.02
10.5 10.0 9.5 9.0 8.5 8.0 7.5 7.0 6.5 6.0 5.5 5.0 4.5 4.0 3.5 3.0 2.5 2.0 1.5 1.0 0.5 0.0
Chemical Shift (ppm)

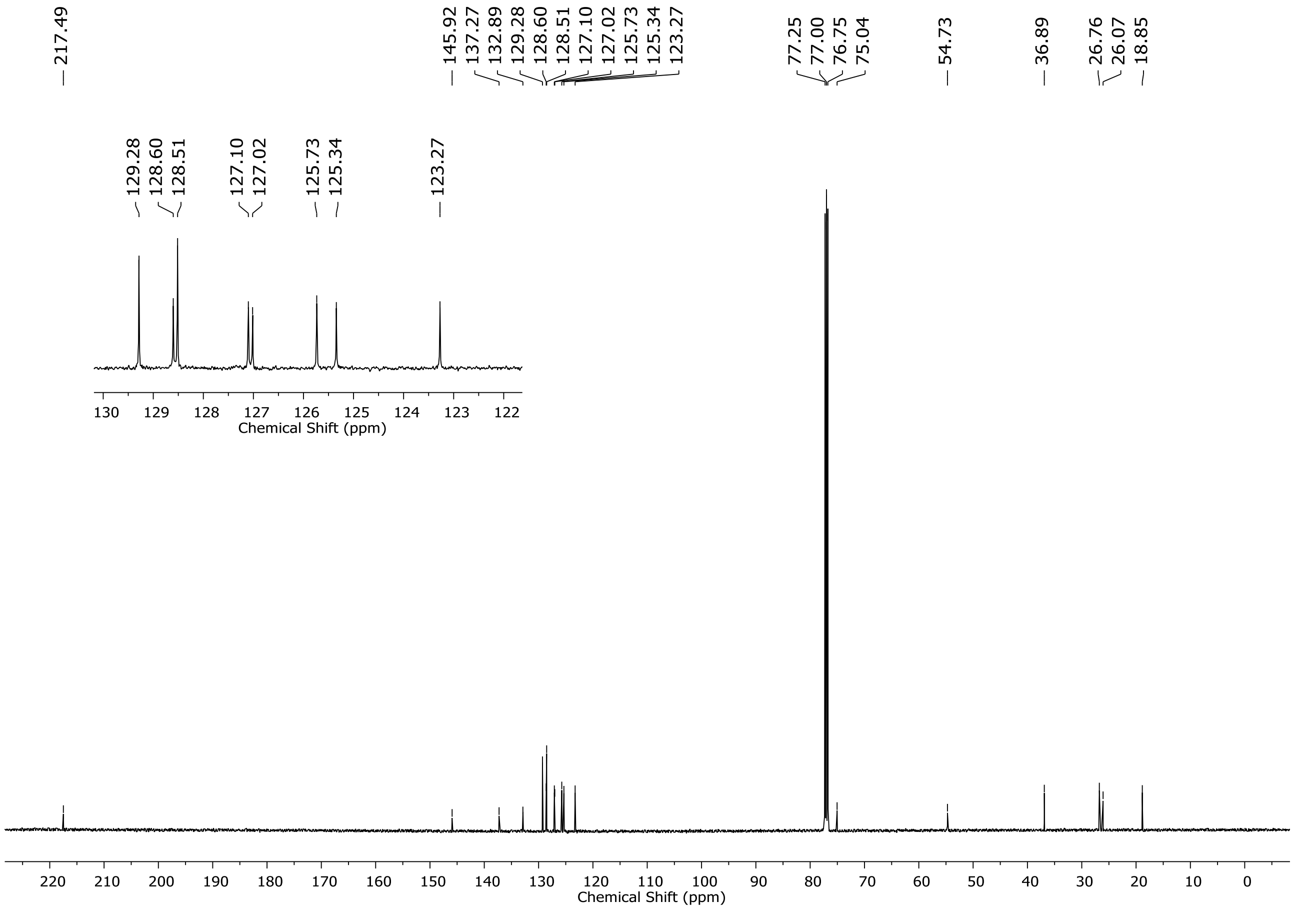
217.49
145.92
137.27
132.89
129.28
128.60
128.51
127.10
127.02
125.73
125.34
123.27
77.25
77.00
76.75
75.04
54.73
36.89
26.76
26.07
18.85
129.28
128.60
128.51
127.10
127.02
125.73
125.34
123.27
130 129 128 127 126 125 124 123 122
Chemical Shift (ppm)
220 210 200 190 180 170 160 150 140 130 120 110 100 90 80 70 60 50 40 30 20 10 0
Chemical Shift (ppm)

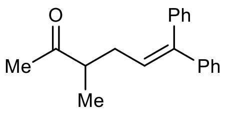
O
Ph
Me
Ph
Me

3a

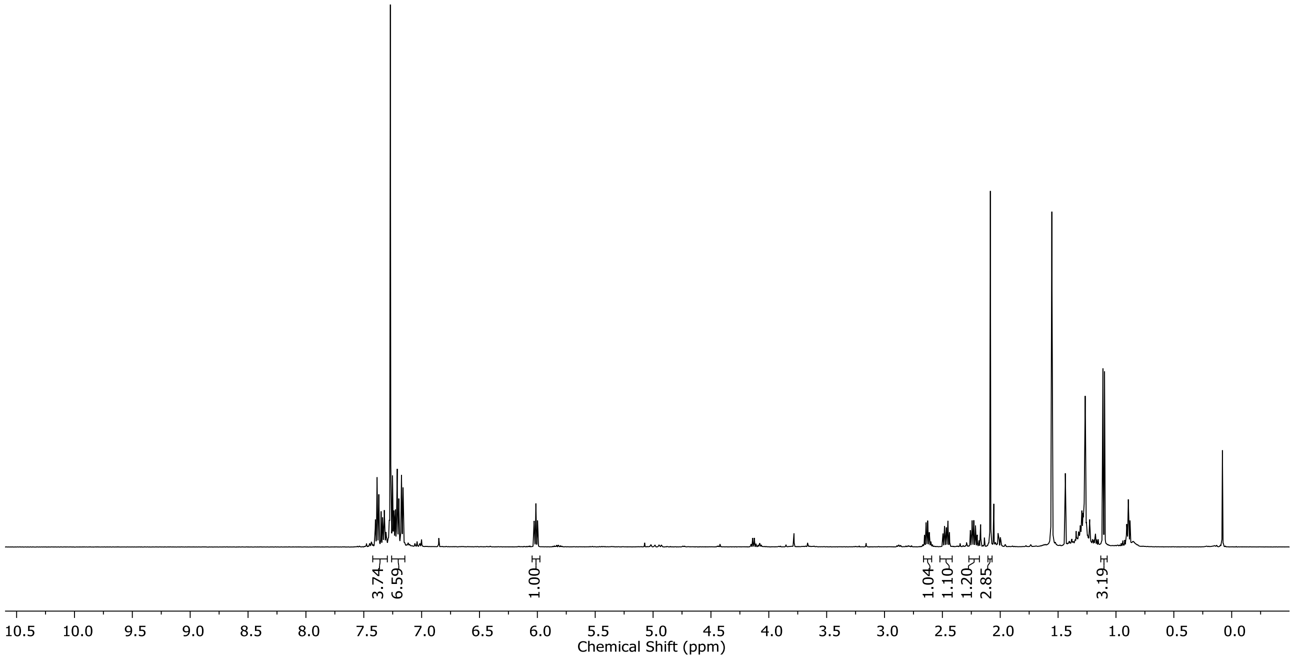
3.74
6.59
1.00
1.04
1.10
1.20
2.85
3.19
10.5
10.0
9.5
9.0
8.5
8.0
7.5
7.0
6.5
6.0
5.5
5.0
4.5
4.0
3.5
3.0
2.5
2.0
1.5
1.0
0.5
0.0
Chemical Shift (ppm)

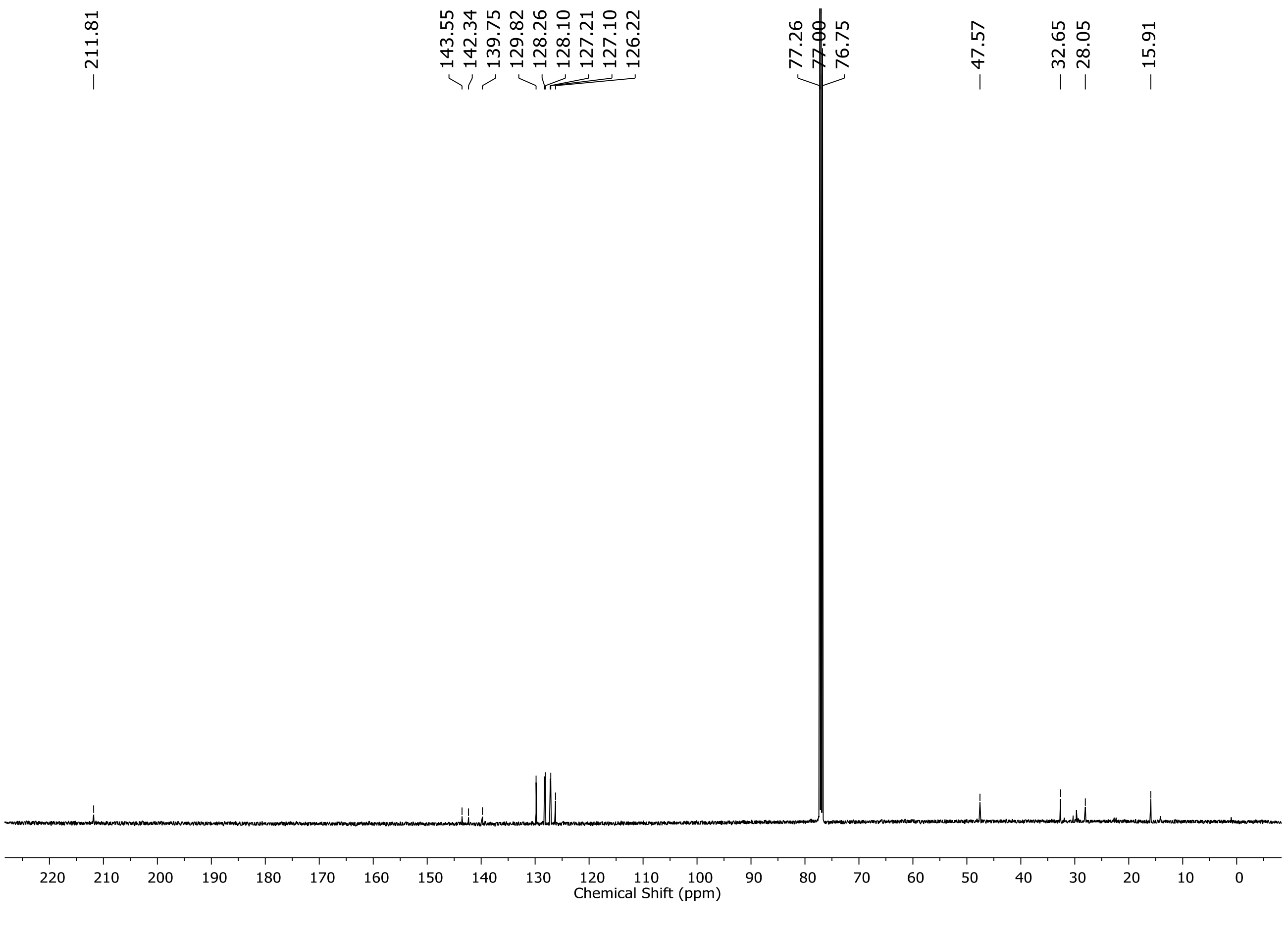
211.81
143.55
142.34
139.75
129.82
128.26
128.10
127.21
127.10
126.22
77.26
77.00
76.75
47.57
32.65
28.05
15.91
220
210
200
190
180
170
160
150
140
130
120
110
100
90
80
70
60
50
40
30
20
10
0
Chemical Shift (ppm)

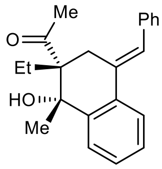
Me
Ph
O
Et
HO
Me

2b

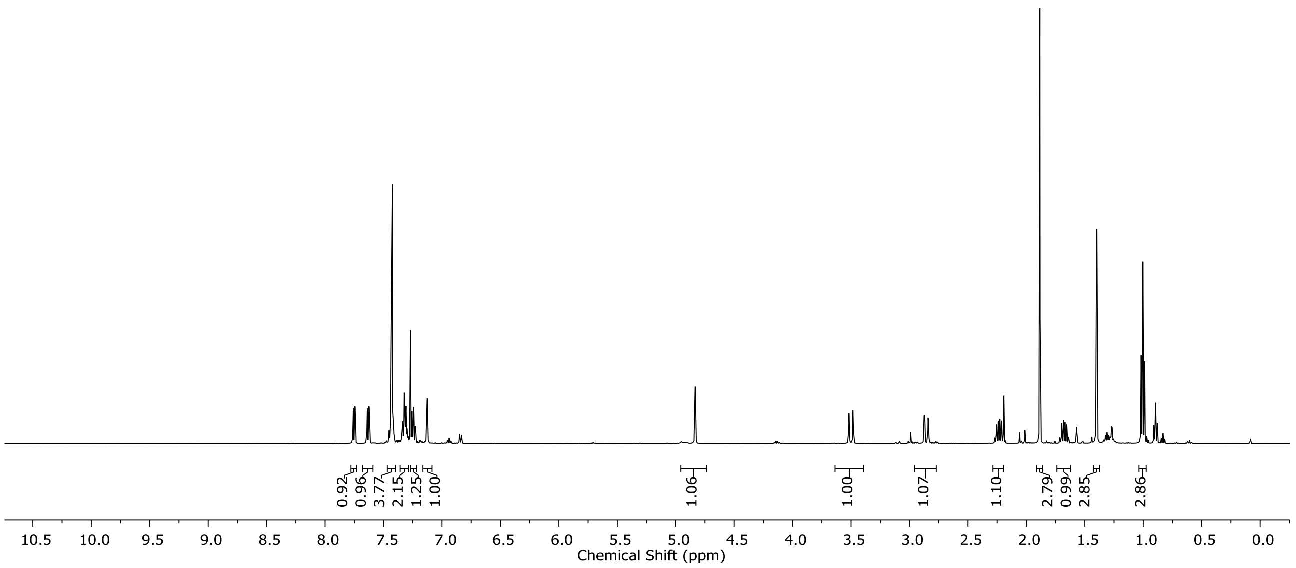
0.92
0.96
3.77
2.15
1.25
1.00
1.06
1.00
1.07
1.10
2.79
0.99
2.85
2.86
10.5 10.0 9.5 9.0 8.5 8.0 7.5 7.0 6.5 6.0 5.5 5.0 4.5 4.0 3.5 3.0 2.5 2.0 1.5 1.0 0.5 0.0
Chemical Shift (ppm)

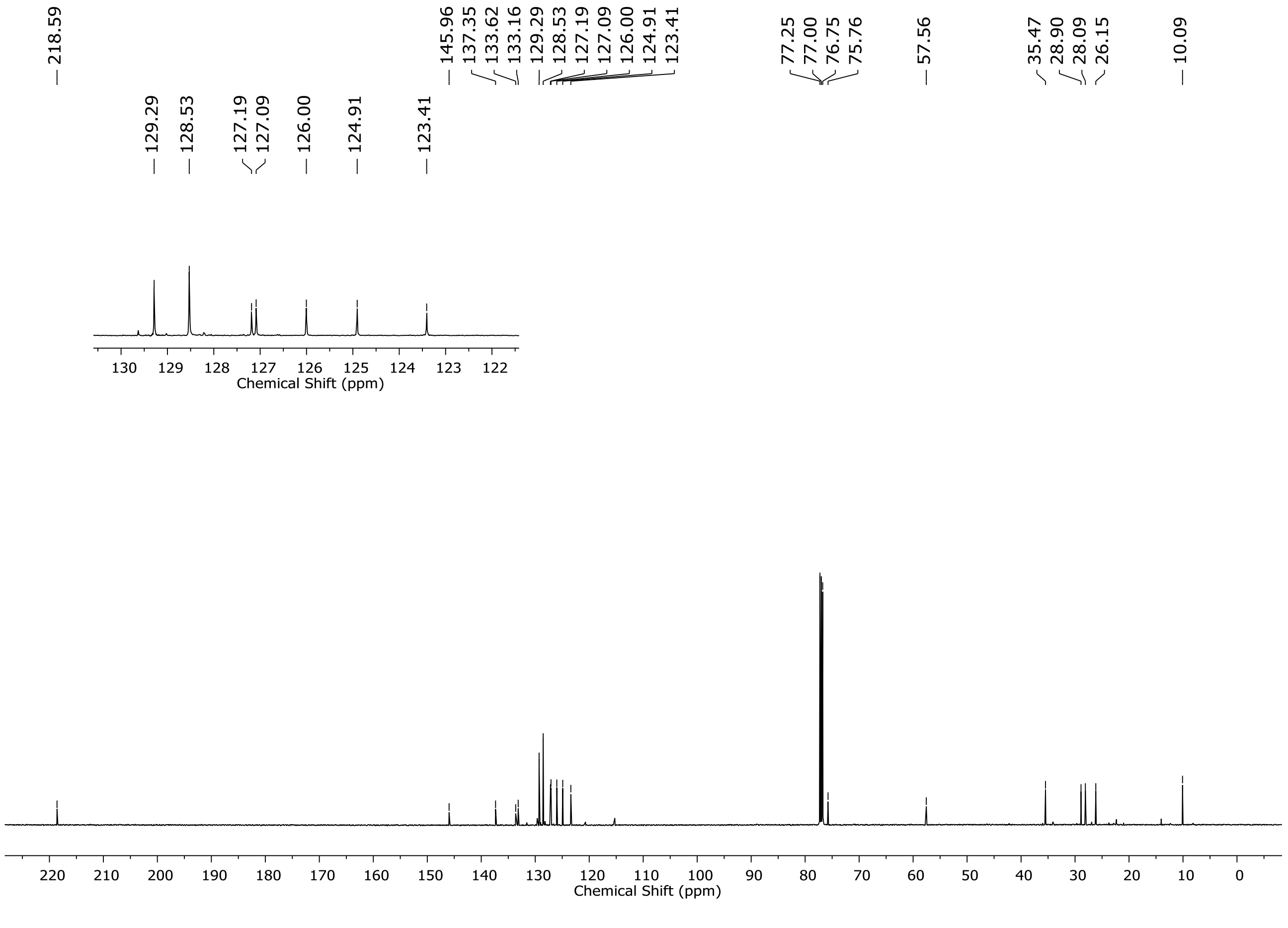
218.59
145.96
137.35
133.62
133.16
129.29
128.53
127.19
127.09
126.00
124.91
123.41
77.25
77.00
76.75
75.76
57.56
35.47
28.90
28.09
26.15
10.09
129.29
128.53
127.19
127.09
126.00
124.91
123.41
130 129 128 127 126 125 124 123 122
Chemical Shift (ppm)
220 210 200 190 180 170 160 150 140 130 120 110 100 90 80 70 60 50 40 30 20 10 0
Chemical Shift (ppm)

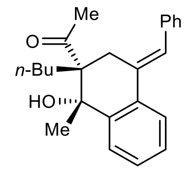
Me
O
n-Bu
HO
Me
Ph

2c

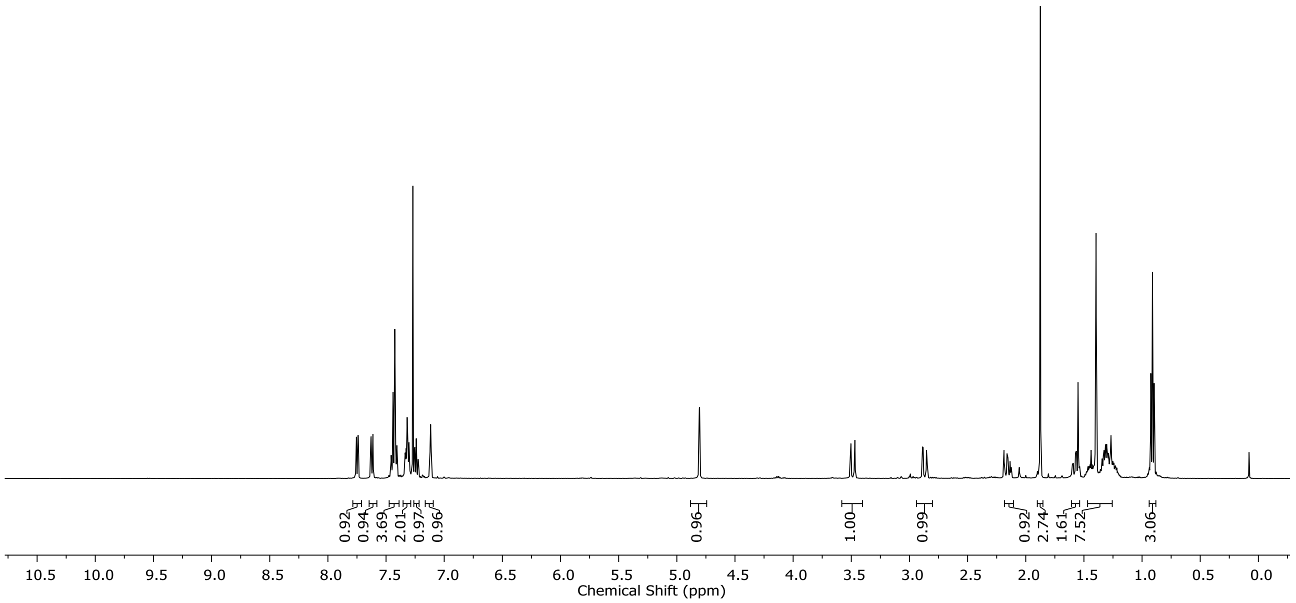
0.92
0.94
3.69
2.01
0.97
0.96
0.96
1.00
0.99
0.92
2.74
1.61
7.52
3.06
10.5 10.0 9.5 9.0 8.5 8.0 7.5 7.0 6.5 6.0 5.5 5.0 4.5 4.0 3.5 3.0 2.5 2.0 1.5 1.0 0.5 0.0
Chemical Shift (ppm)

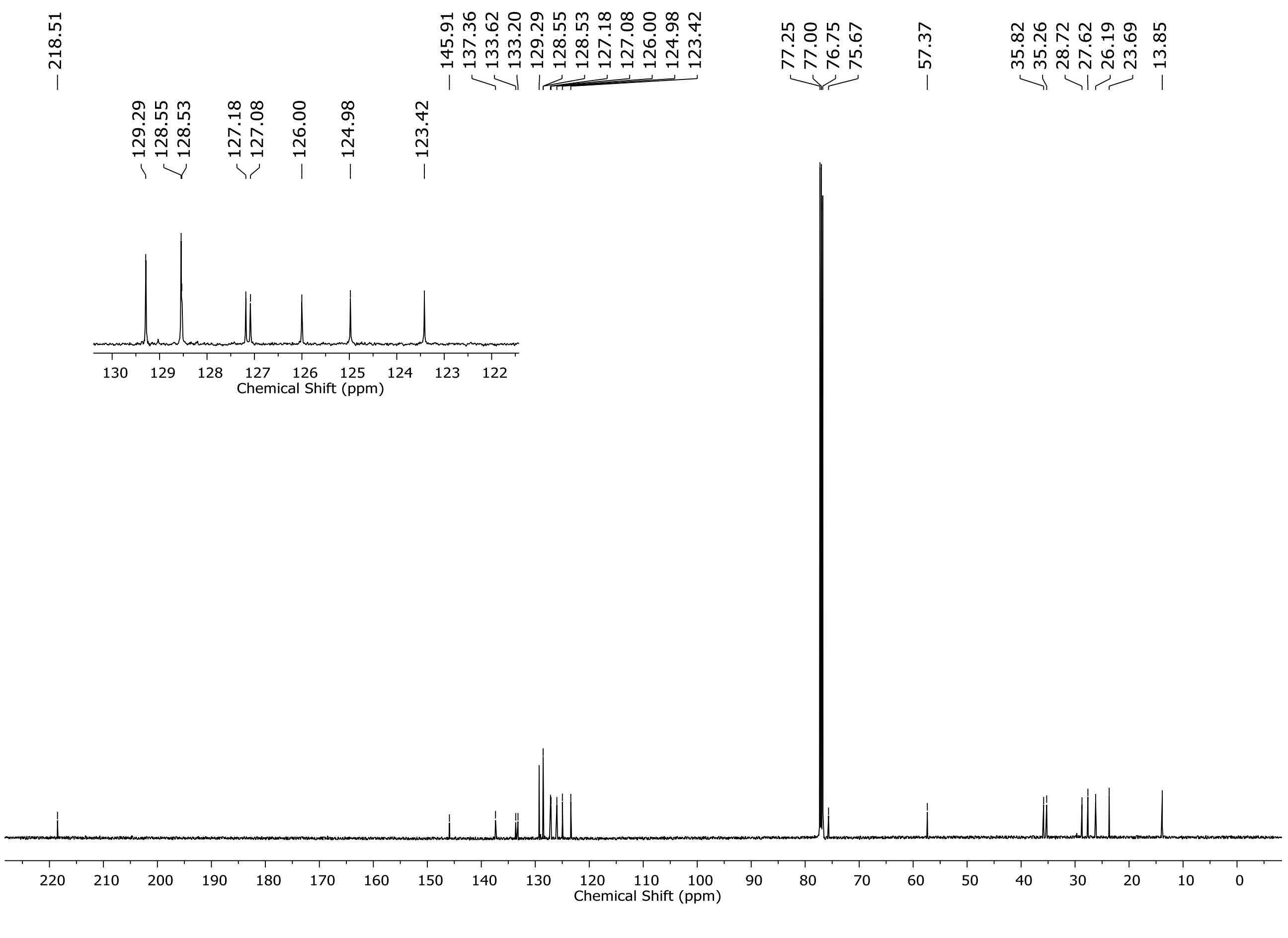
218.51
145.91
137.36
133.62
133.20
129.29
128.55
128.53
127.18
127.08
126.00
124.98
123.42
77.25
77.00
76.75
75.67
57.37
35.82
35.26
28.72
27.62
26.19
23.69
13.85
129.29
128.55
128.53
127.18
127.08
126.00
124.98
123.42
130 129 128 127 126 125 124 123 122
Chemical Shift (ppm)
220 210 200 190 180 170 160 150 140 130 120 110 100 90 80 70 60 50 40 30 20 10 0
Chemical Shift (ppm)

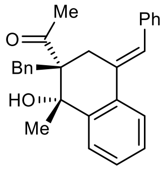
Me
O
Ph
Bn
HO
Me

2d

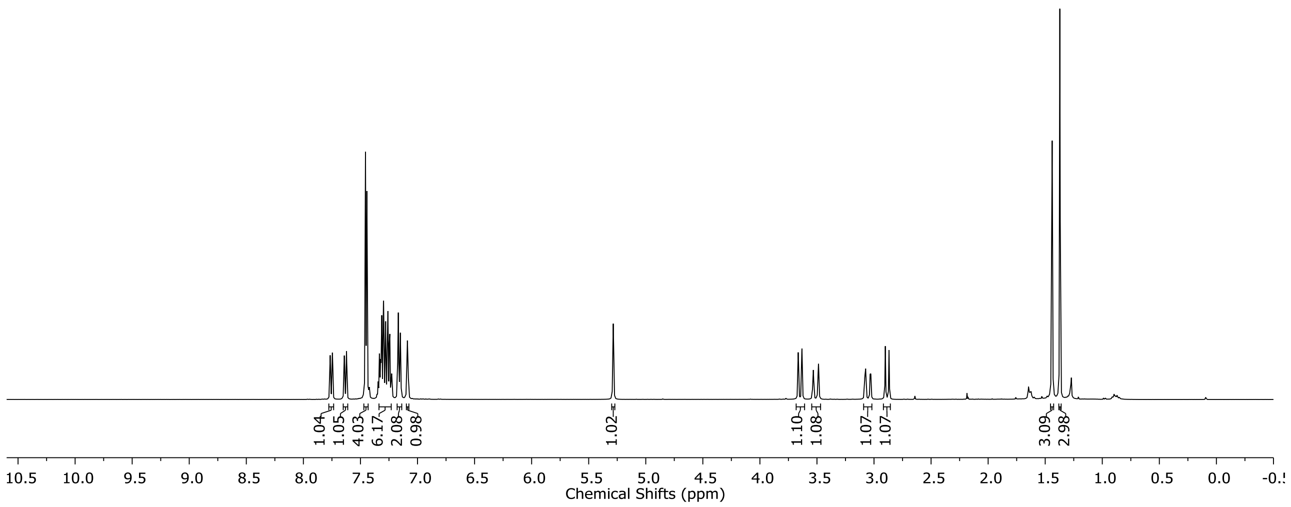
1.04
1.05
4.03
6.17
2.08
0.98
1.02
1.10
1.08
1.07
1.07
3.09
2.98
10.5 10.0 9.5 9.0 8.5 8.0 7.5 7.0 6.5 6.0 5.5 5.0 4.5 4.0 3.5 3.0 2.5 2.0 1.5 1.0 0.5 0.0
Chemical Shifts (ppm)

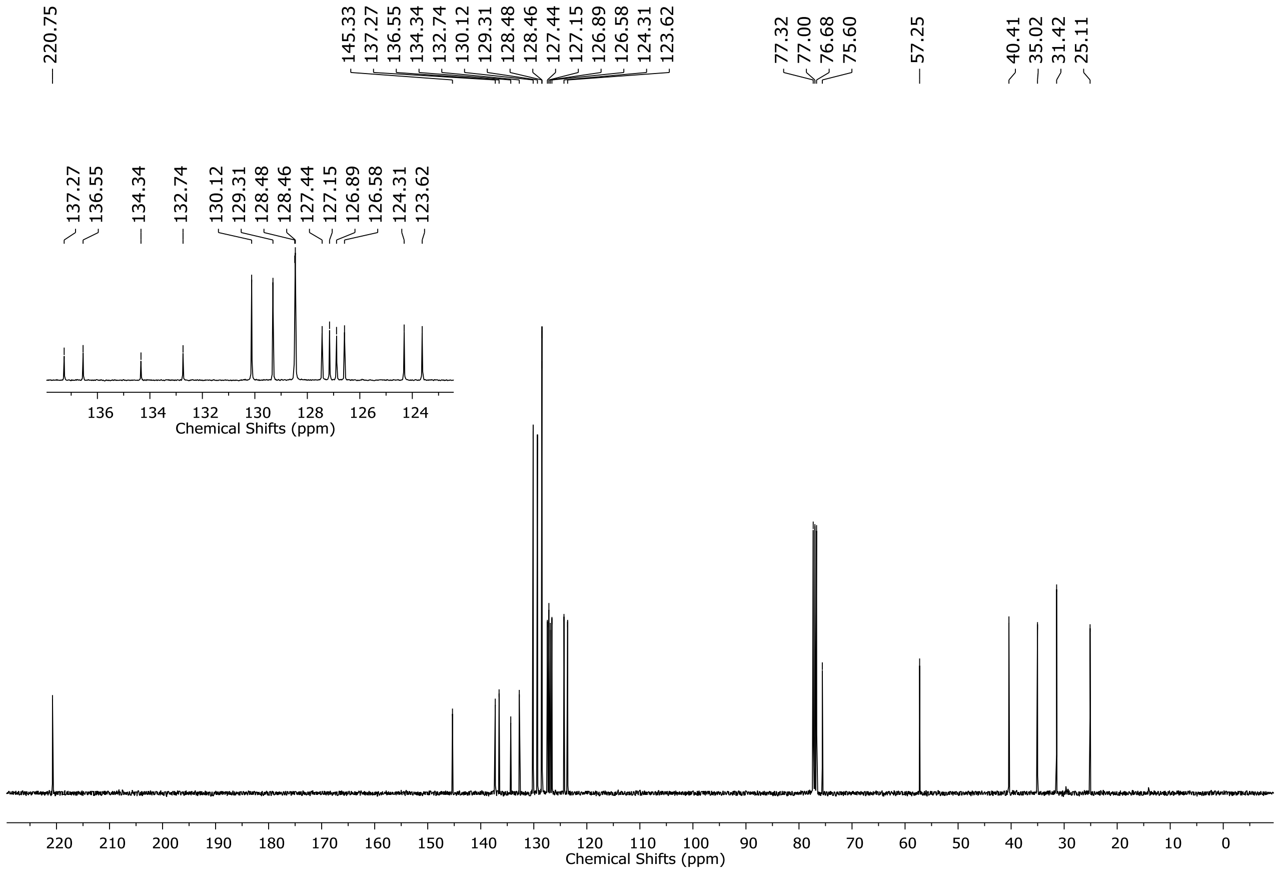
220.75
145.33
137.27
136.55
134.34
132.74
130.12
129.31
128.48
128.46
127.44
127.15
126.89
126.58
124.31
123.62
77.32
77.00
76.68
75.60
57.25
40.41
35.02
31.42
25.11
136 134 132 130 128 126 124
Chemical Shifts (ppm)
220 210 200 190 180 170 160 150 140 130 120 110 100 90 80 70 60 50 40 30 20 10 0
Chemical Shifts (ppm)

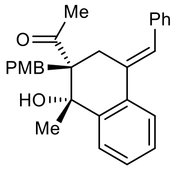
Me
Ph
O
PMB
HO
Me

2e

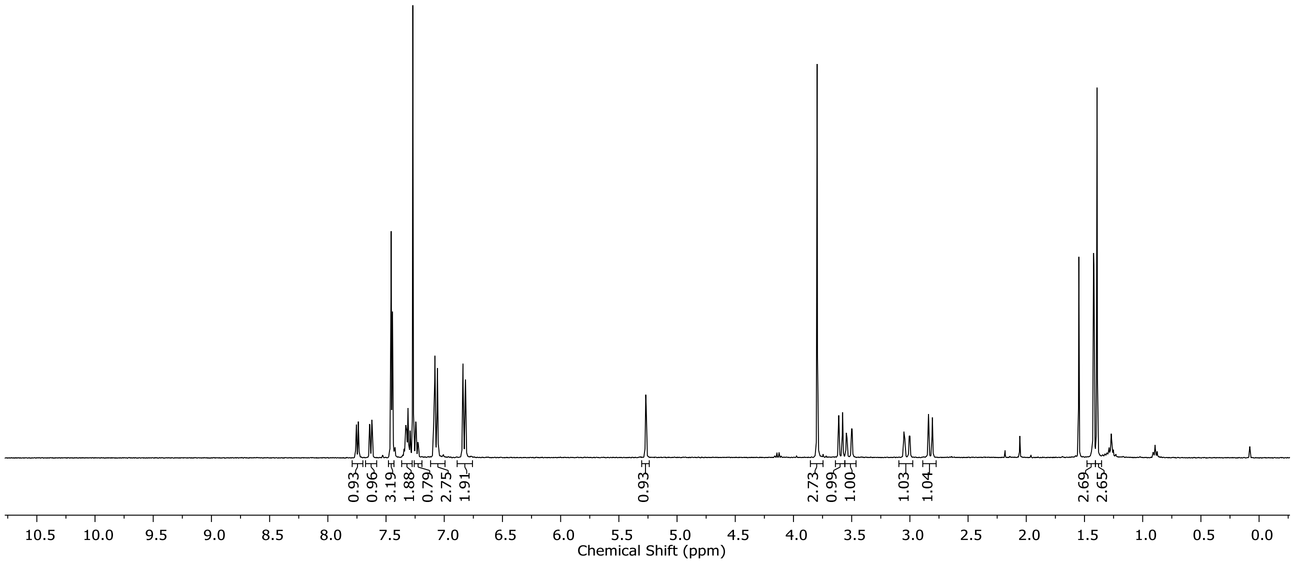
0.93
0.96
3.19
1.88
0.79
2.75
1.91
0.93
2.73
0.99
1.00
1.03
1.04
2.69
2.65
10.5 10.0 9.5 9.0 8.5 8.0 7.5 7.0 6.5 6.0 5.5 5.0 4.5 4.0 3.5 3.0 2.5 2.0 1.5 1.0 0.5 0.0
Chemical Shift (ppm)

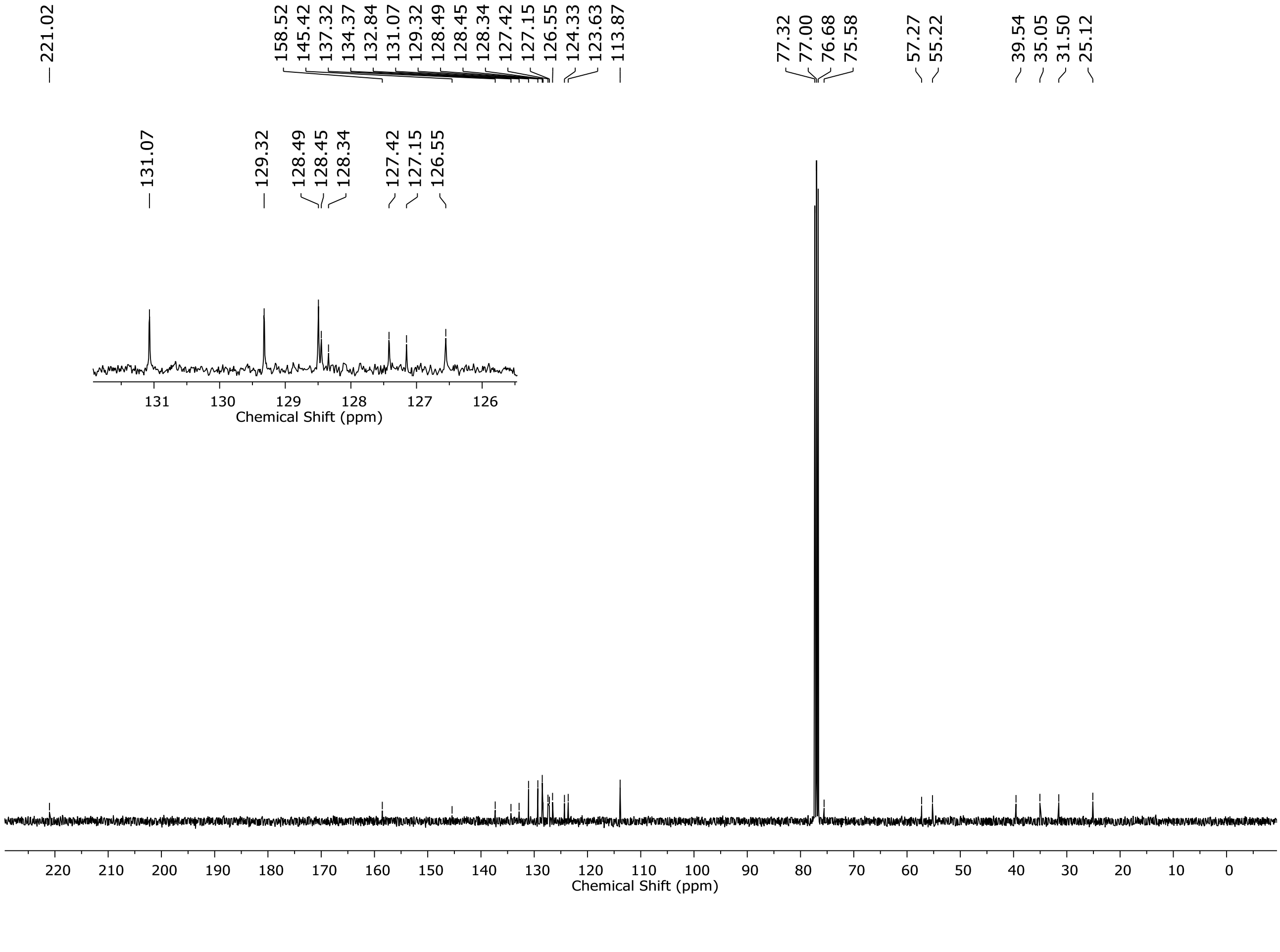
221.02
158.52
145.42
137.32
134.37
132.84
131.07
129.32
128.49
128.45
128.34
127.42
127.15
126.55
124.33
123.63
113.87
77.32
77.00
76.68
75.58
57.27
55.22
39.54
35.05
31.50
25.12
131.07
129.32
128.49
128.45
128.34
127.42
127.15
126.55
131 130 129 128 127 126
Chemical Shift (ppm)
220 210 200 190 180 170 160 150 140 130 120 110 100 90 80 70 60 50 40 30 20 10 0
Chemical Shift (ppm)

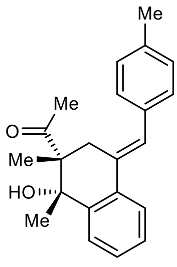
Me
Me
O
Me
HO
Me

2f

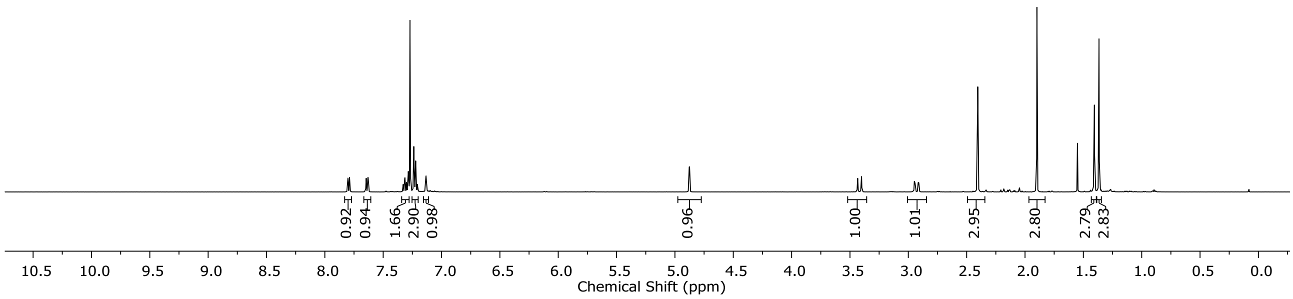
0.92
0.94
1.66
2.90
0.98
0.96
1.00
1.01
2.95
2.80
2.79
2.83
10.5 10.0 9.5 9.0 8.5 8.0 7.5 7.0 6.5 6.0 5.5 5.0 4.5 4.0 3.5 3.0 2.5 2.0 1.5 1.0 0.5 0.0
Chemical Shift (ppm)

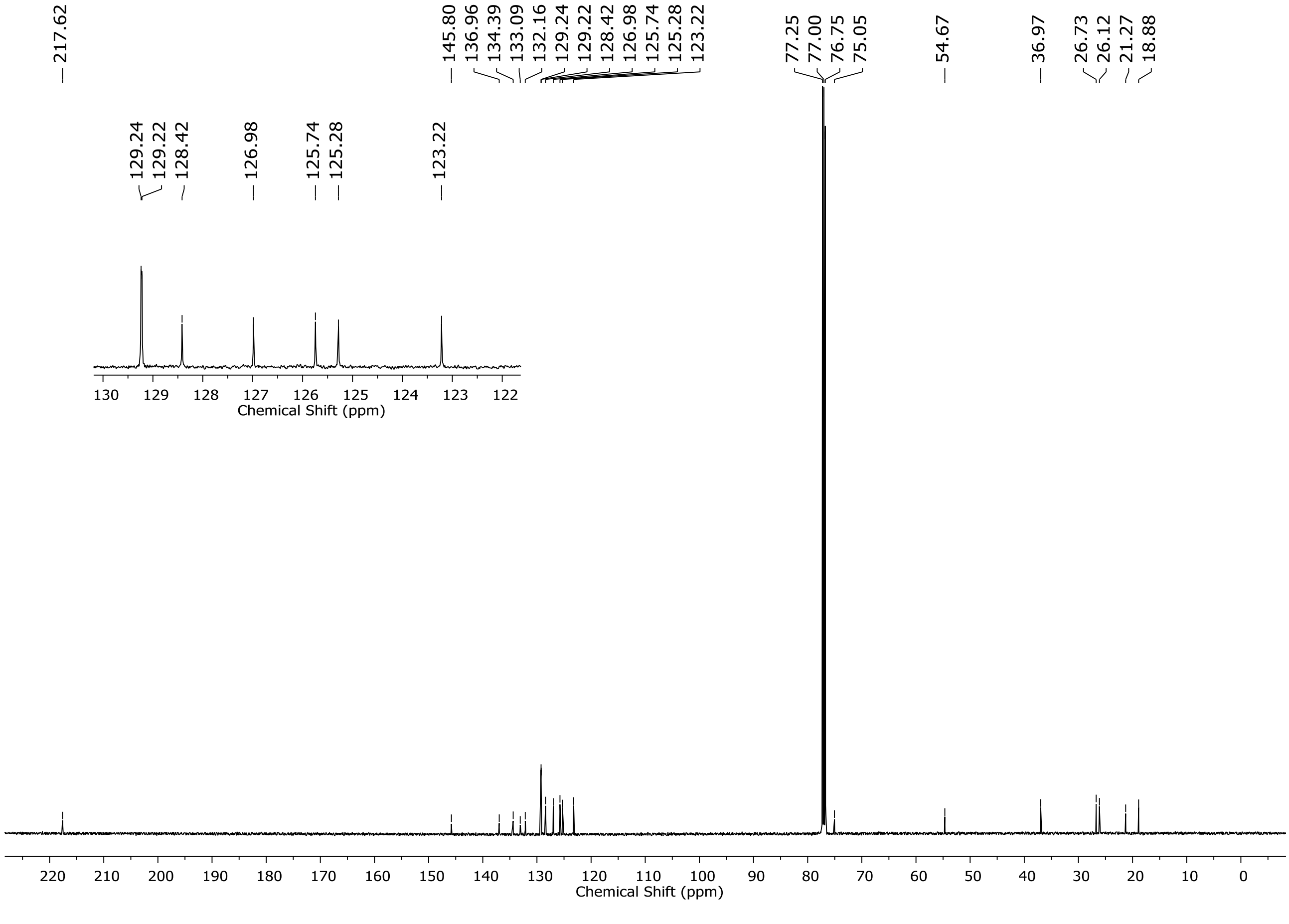
217.62
145.80
136.96
134.39
133.09
132.16
129.24
129.22
128.42
126.98
125.74
125.28
123.22
77.25
77.00
76.75
75.05
54.67
36.97
26.73
26.12
21.27
18.88
129.24
129.22
128.42
126.98
125.74
125.28
123.22
130 129 128 127 126 125 124 123 122
Chemical Shift (ppm)
220 210 200 190 180 170 160 150 140 130 120 110 100 90 80 70 60 50 40 30 20 10 0
Chemical Shift (ppm)

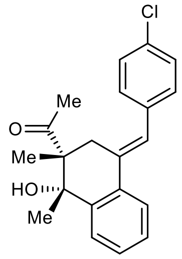
Cl
Me
O
Me
HO
Me

2g

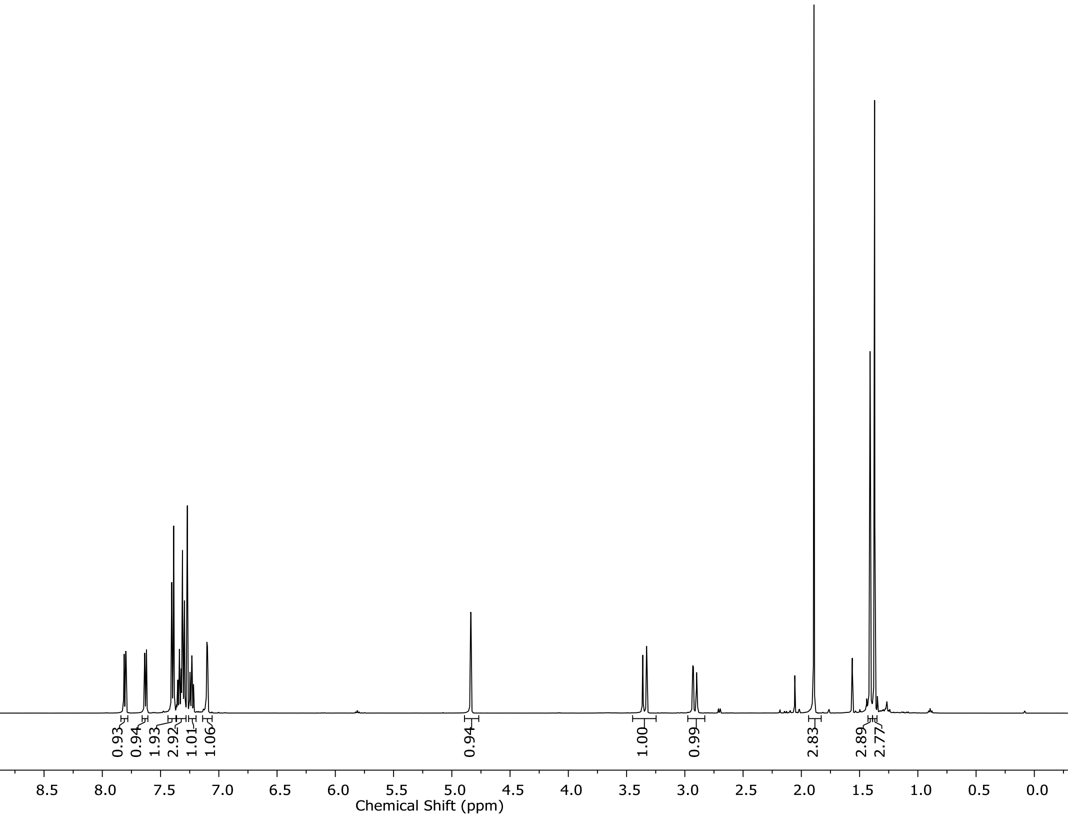
0.93
0.94
1.93
2.92
1.01
1.06
0.94
1.00
0.99
2.83
2.89
2.77
10.5 10.0 9.5 9.0 8.5 8.0 7.5 7.0 6.5 6.0 5.5 5.0 4.5 4.0 3.5 3.0 2.5 2.0 1.5 1.0 0.5 0.0
Chemical Shift (ppm)

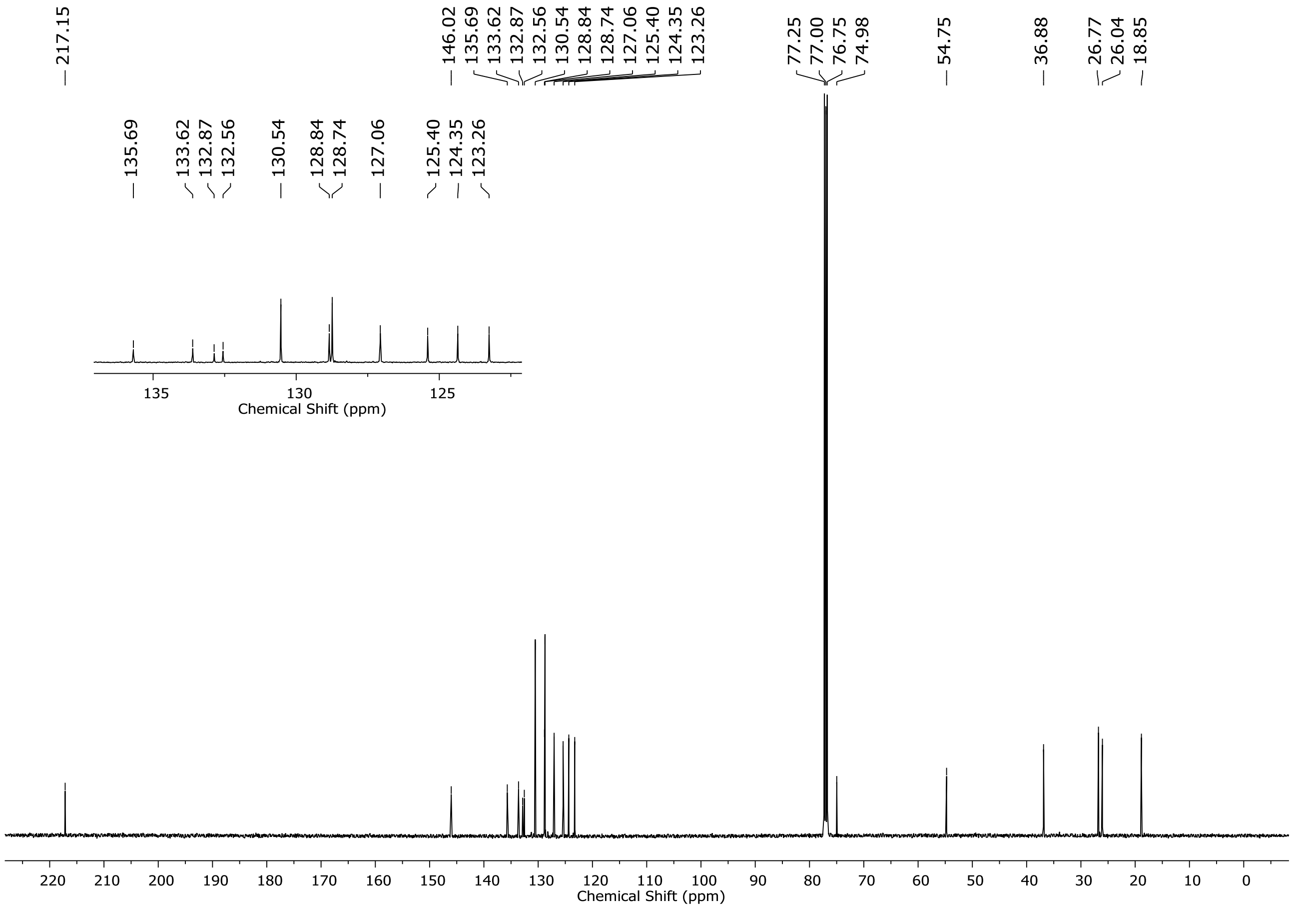
217.15
146.02
135.69
133.62
132.87
132.56
130.54
128.84
128.74
127.06
125.40
124.35
123.26
77.25
77.00
76.75
74.98
54.75
36.88
26.77
26.04
18.85
135.69
133.62
132.87
132.56
130.54
128.84
128.74
127.06
125.40
124.35
123.26
135 130 125
Chemical Shift (ppm)
220 210 200 190 180 170 160 150 140 130 120 110 100 90 80 70 60 50 40 30 20 10 0
Chemical Shift (ppm)

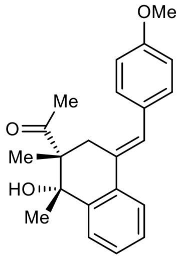
OMe
Me
O
Me
HO
Me

2h

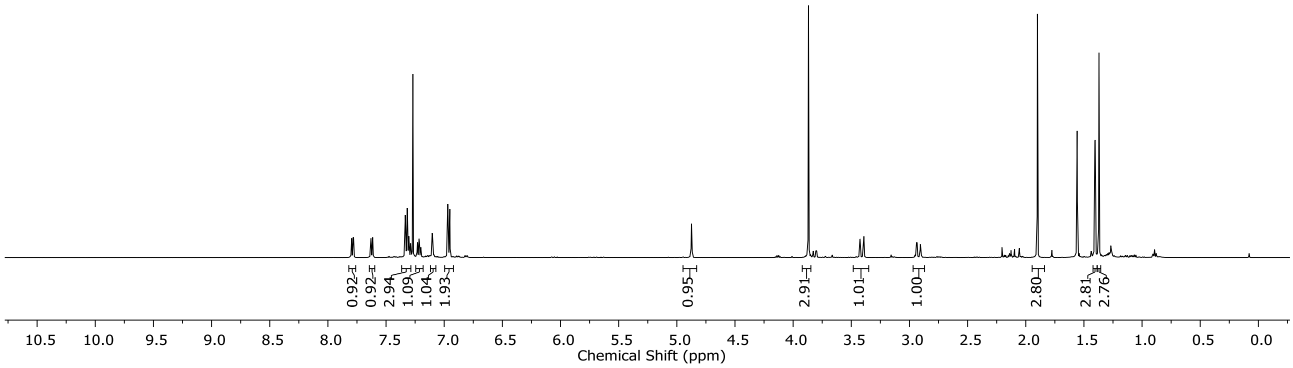
0.92
0.92
2.94
1.09
1.04
1.93
0.95
2.91
1.01
1.00
2.80
2.81
2.76
10.5 10.0 9.5 9.0 8.5 8.0 7.5 7.0 6.5 6.0 5.5 5.0 4.5 4.0 3.5 3.0 2.5 2.0 1.5 1.0 0.5 0.0
Chemical Shift (ppm)

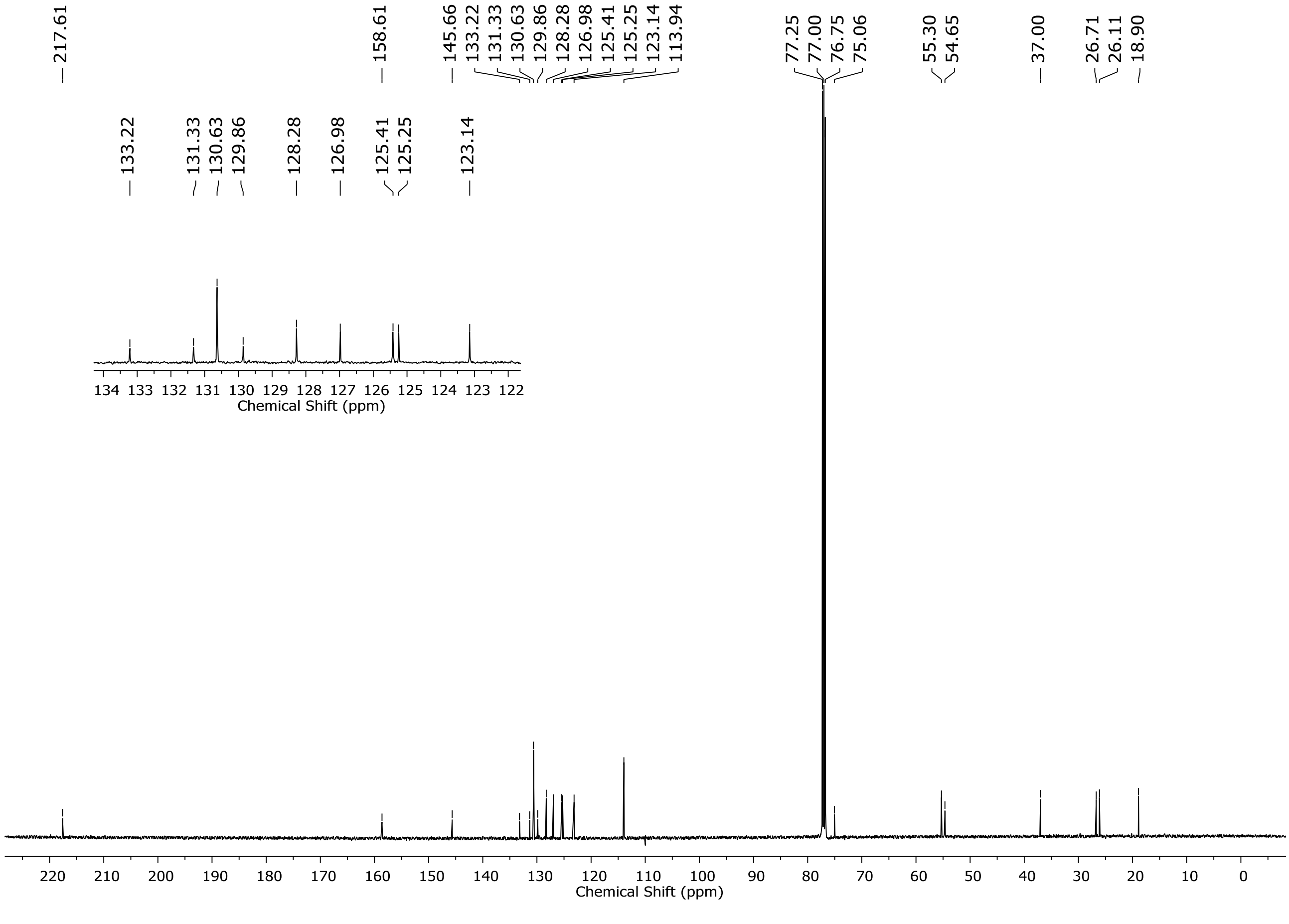
217.61
158.61
145.66
133.22
131.33
130.63
129.86
128.28
126.98
125.41
125.25
123.14
113.94
77.25
77.00
76.75
75.06
55.30
54.65
37.00
26.71
26.11
18.90
133.22
131.33
130.63
129.86
128.28
126.98
125.41
125.25
123.14
134 133 132 131 130 129 128 127 126 125 124 123 122
Chemical Shift (ppm)
220 210 200 190 180 170 160 150 140 130 120 110 100 90 80 70 60 50 40 30 20 10 0
Chemical Shift (ppm)

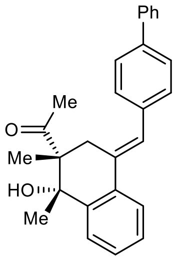
Ph
Me
O
Me
HO
Me

2i

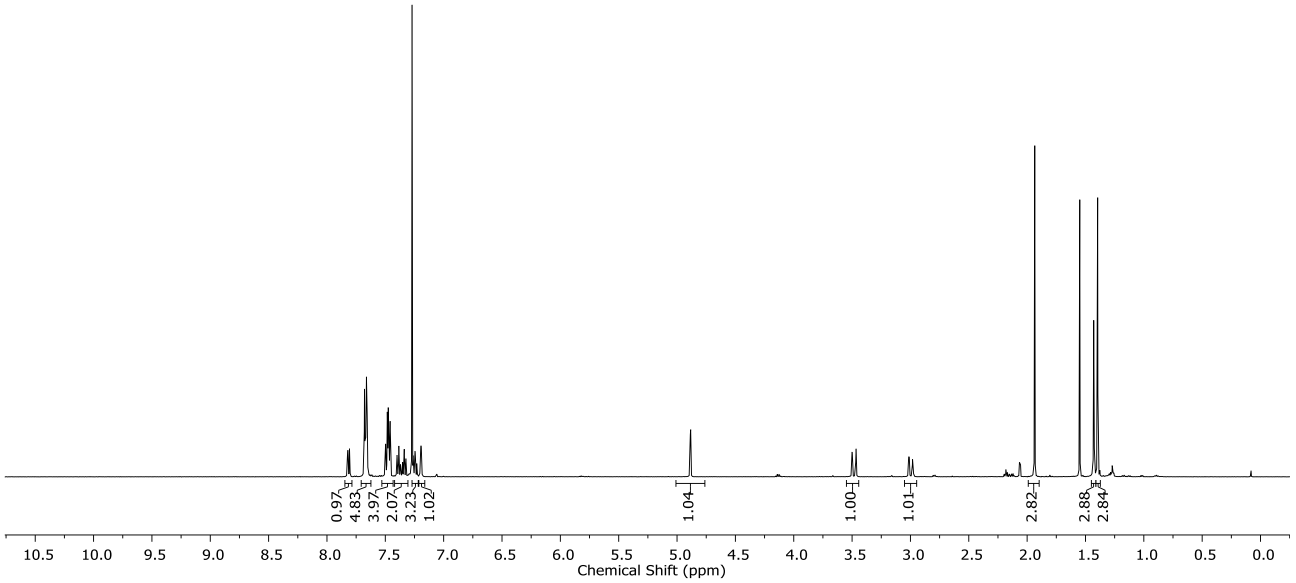
0.97
4.83
3.97
2.07
3.23
1.02
1.04
1.00
1.01
2.82
2.88
2.84
10.5 10.0 9.5 9.0 8.5 8.0 7.5 7.0 6.5 6.0 5.5 5.0 4.5 4.0 3.5 3.0 2.5 2.0 1.5 1.0 0.5 0.0
Chemical Shift (ppm)

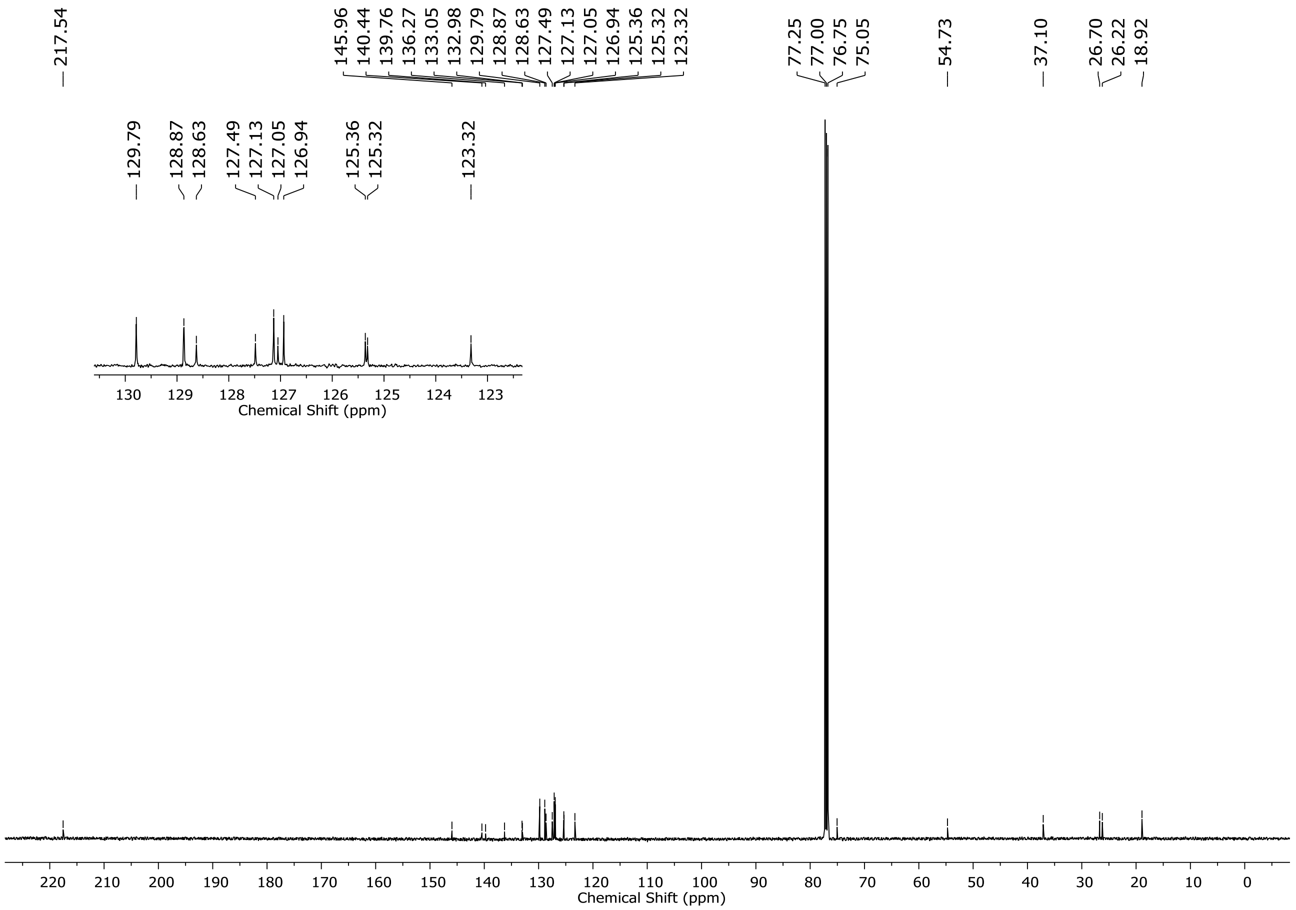
217.54
145.96
140.44
139.76
136.27
133.05
132.98
129.79
128.87
128.63
127.49
127.13
127.05
126.94
125.36
125.32
123.32
77.25
77.00
76.75
75.05
54.73
37.10
26.70
26.22
18.92
129.79
128.87
128.63
127.49
127.13
127.05
126.94
125.36
125.32
123.32
130 129 128 127 126 125 124 123
Chemical Shift (ppm)
220 210 200 190 180 170 160 150 140 130 120 110 100 90 80 70 60 50 40 30 20 10 0
Chemical Shift (ppm)

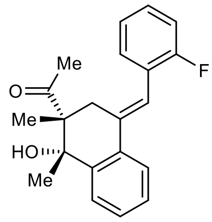
Me
O
Me
HO
Me
F

2j

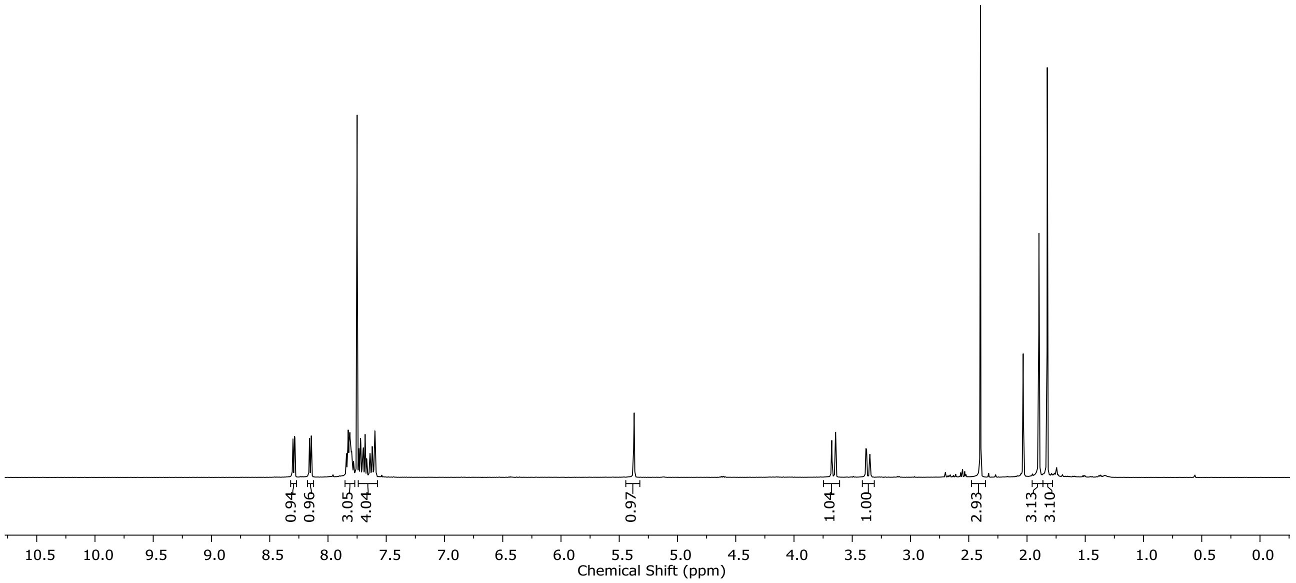
0.94
0.96
3.05
4.04
0.97
1.04
1.00
2.93
3.13
3.10
10.5 10.0 9.5 9.0 8.5 8.0 7.5 7.0 6.5 6.0 5.5 5.0 4.5 4.0 3.5 3.0 2.5 2.0 1.5 1.0 0.5 0.0
Chemical Shift (ppm)

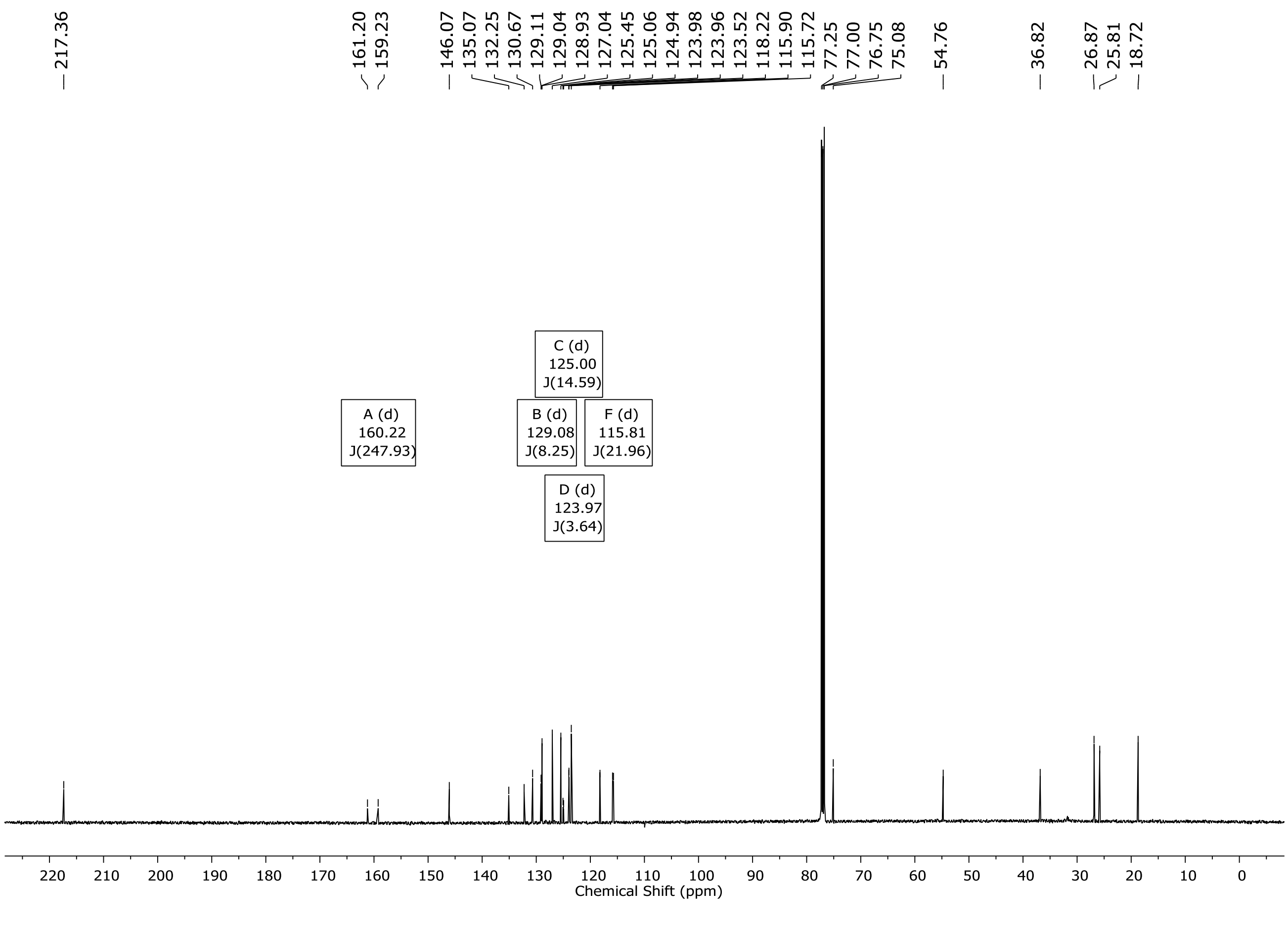
217.36
161.20
159.23
146.07
135.07
132.25
130.67
129.11
129.04
128.93
127.04
125.45
125.06
124.94
123.98
123.96
123.52
118.22
115.90
115.72
77.25
77.00
76.75
75.08
54.76
36.82
26.87
25.81
18.72
C (d)
125.00
J(14.59)
A (d)
160.22
J(247.93)
B (d)
129.08
J(8.25)
F (d)
115.81
J(21.96)
D (d)
123.97
J(3.64)
220 210 200 190 180 170 160 150 140 130 120 110 100 90 80 70 60 50 40 30 20 10 0
Chemical Shift (ppm)

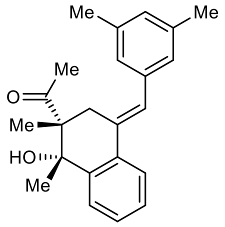
Me
Me
O
Me
Me
HO
Me

2k

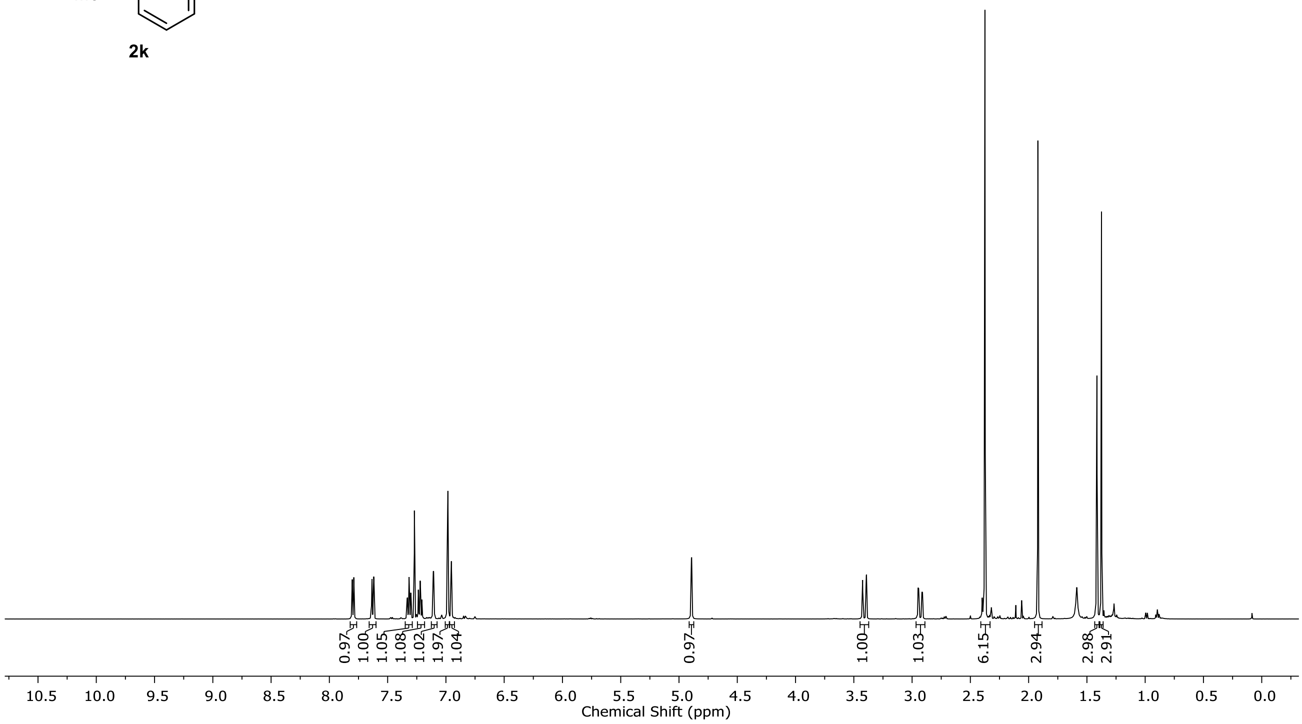
0.97
1.00
1.05
1.08
1.02
1.97
1.04
0.97
1.00
1.03
6.15
2.94
2.98
2.91
10.5 10.0 9.5 9.0 8.5 8.0 7.5 7.0 6.5 6.0 5.5 5.0 4.5 4.0 3.5 3.0 2.5 2.0 1.5 1.0 0.5 0.0
Chemical Shift (ppm)

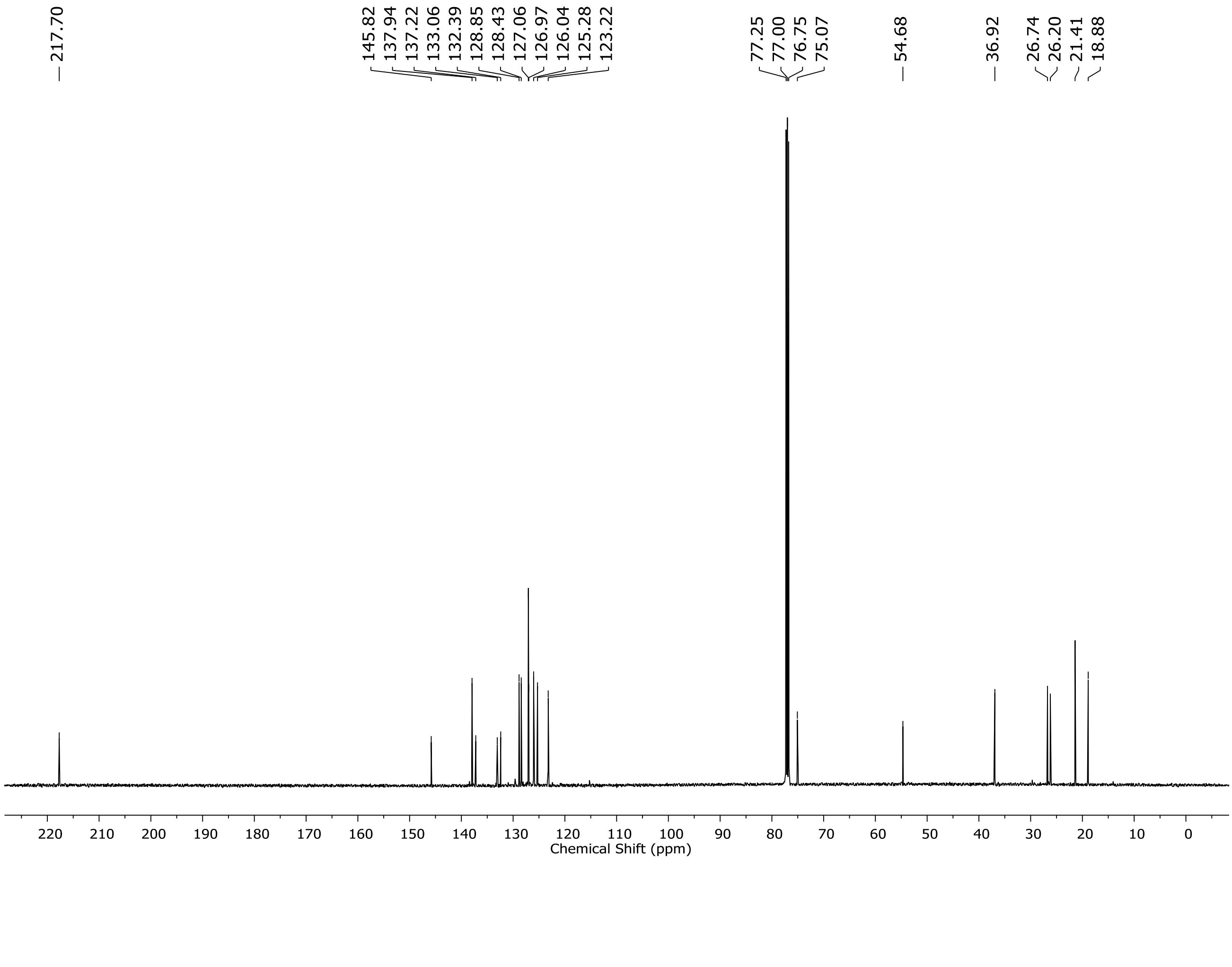
217.70
145.82
137.94
137.22
133.06
132.39
128.85
128.43
127.06
126.97
126.04
125.28
123.22
77.25
77.00
76.75
75.07
54.68
36.92
26.74
26.20
21.41
18.88
220 210 200 190 180 170 160 150 140 130 120 110 100 90 80 70 60 50 40 30 20 10 0
Chemical Shift (ppm)

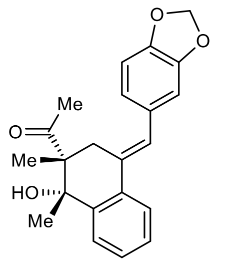
O
O
Me
O
Me
HO
Me

2l

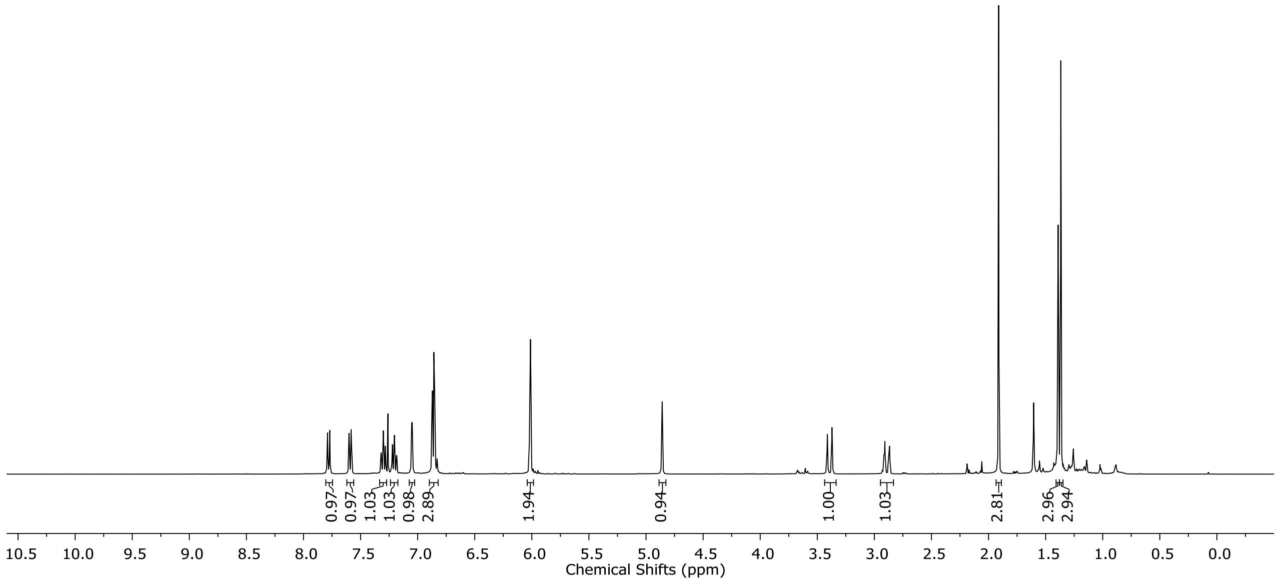
0.97
0.97
1.03
1.03
0.98
2.89
1.94
0.94
1.00
1.03
2.81
2.96
2.94
10.5 10.0 9.5 9.0 8.5 8.0 7.5 7.0 6.5 6.0 5.5 5.0 4.5 4.0 3.5 3.0 2.5 2.0 1.5 1.0 0.5 0.0
Chemical Shifts (ppm)

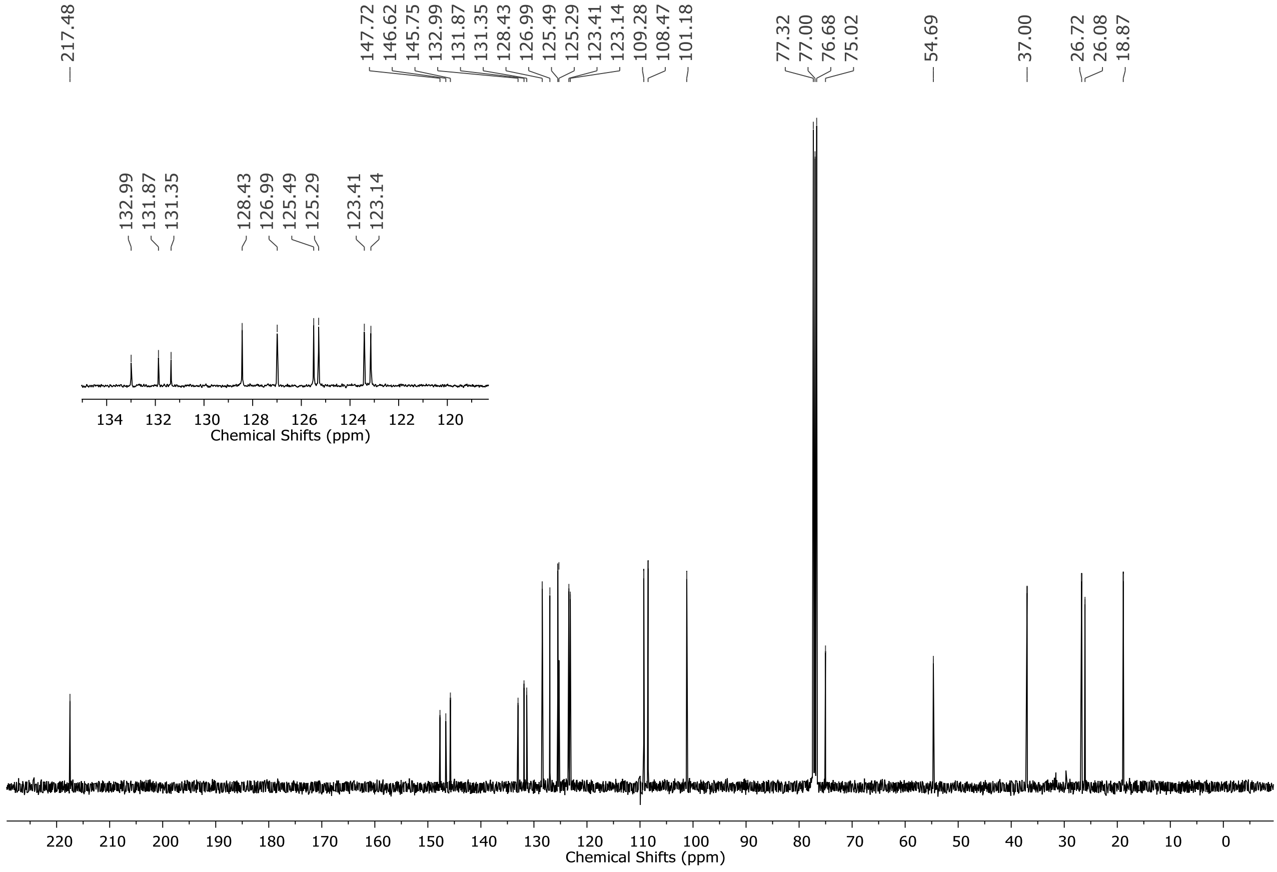
217.48
147.72
146.62
145.75
132.99
131.87
131.35
128.43
126.99
125.49
125.29
123.41
123.14
109.28
108.47
101.18
77.32
77.00
76.68
75.02
54.69
37.00
26.72
26.08
18.87
132.99
131.87
131.35
128.43
126.99
125.49
125.29
123.41
123.14
134 132 130 128 126 124 122 120
Chemical Shifts (ppm)
220 210 200 190 180 170 160 150 140 130 120 110 100 90 80 70 60 50 40 30 20 10 0
Chemical Shifts (ppm)

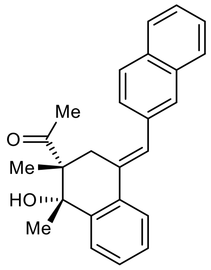
Me
O
Me
HO
Me

2m

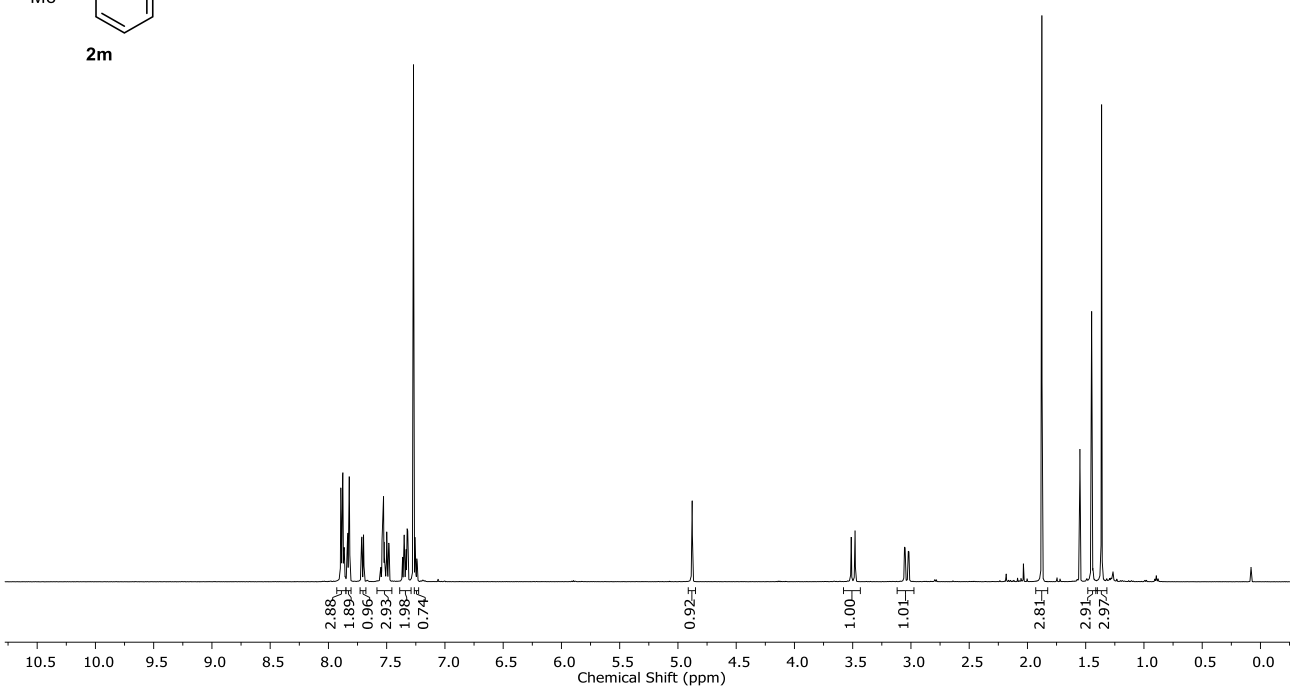
2.88
1.89
0.96
2.93
1.98
0.74
0.92
1.00
1.01
2.81
2.91
2.97
10.5 10.0 9.5 9.0 8.5 8.0 7.5 7.0 6.5 6.0 5.5 5.0 4.5 4.0 3.5 3.0 2.5 2.0 1.5 1.0 0.5 0.0
Chemical Shift (ppm)

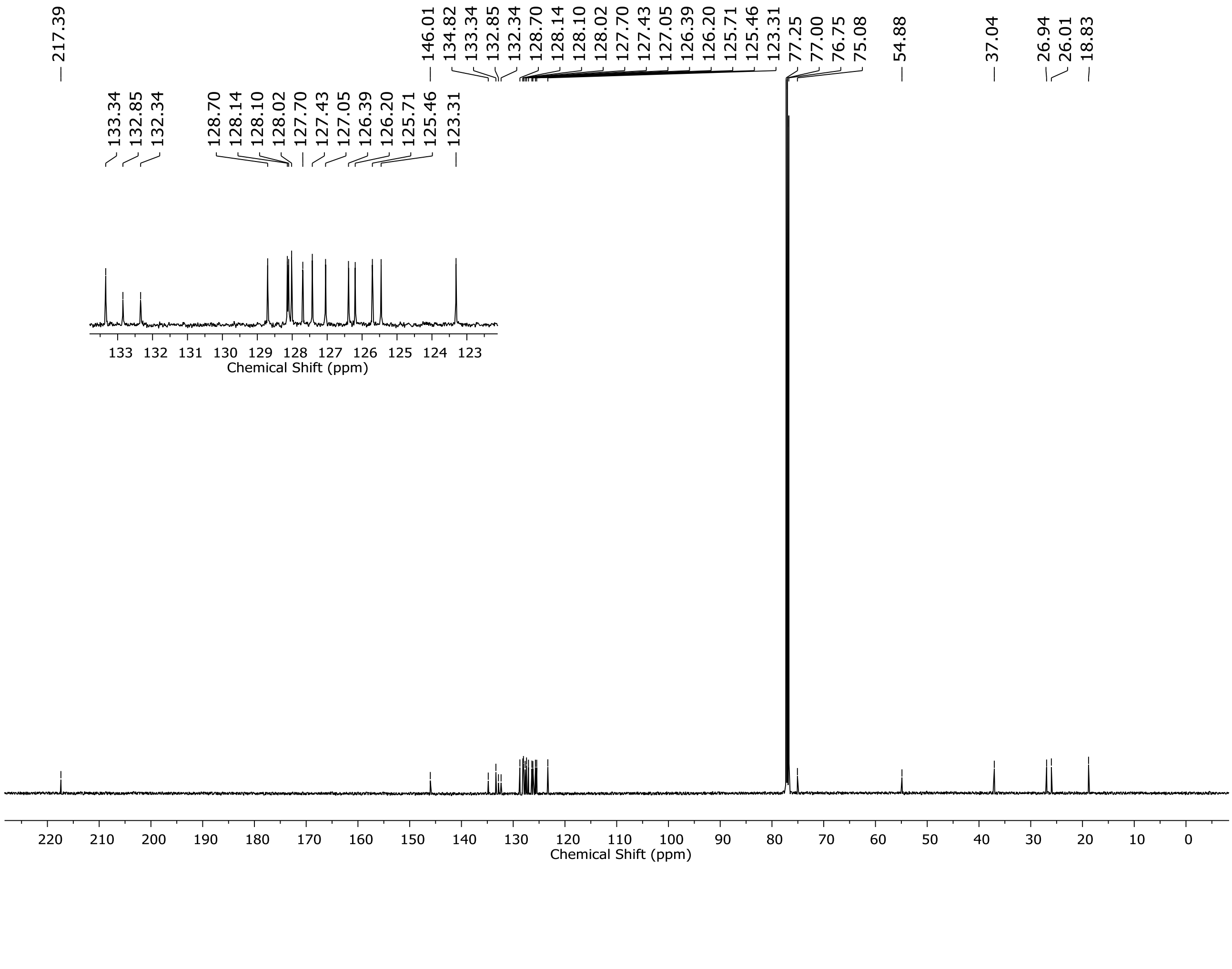
217.39
146.01
134.82
133.34
132.85
132.34
128.70
128.14
128.10
128.02
127.70
127.43
127.05
126.39
126.20
125.71
125.46
123.31
77.25
77.00
76.75
75.08
54.88
37.04
26.94
26.01
18.83
133.34
132.85
132.34
128.70
128.14
128.10
128.02
127.70
127.43
127.05
126.39
126.20
125.71
125.46
123.31
133 132 131 130 129 128 127 126 125 124 123
Chemical Shift (ppm)
220 210 200 190 180 170 160 150 140 130 120 110 100 90 80 70 60 50 40 30 20 10 0
Chemical Shift (ppm)

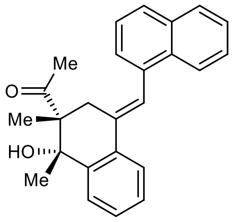
Me
O
Me
HO
Me

2n

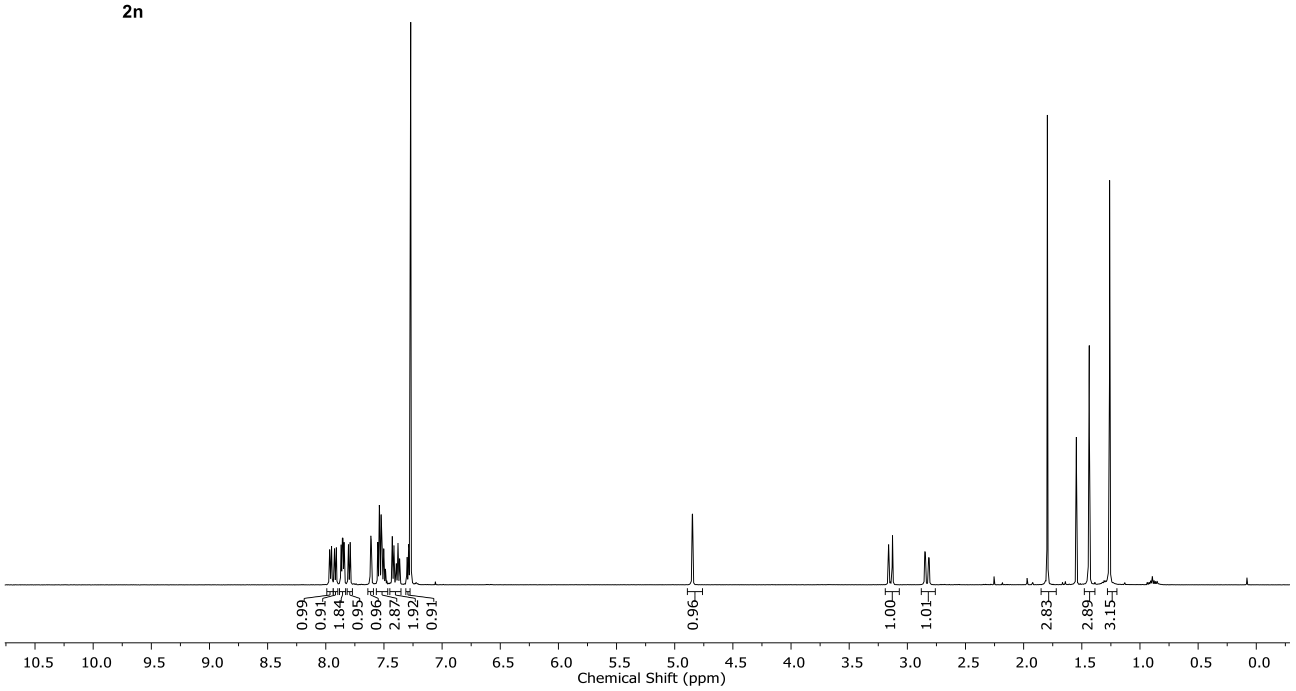
0.99
0.91
1.84
0.95
0.96
2.87
1.92
0.91
0.96
1.00
1.01
2.83
2.89
3.15
10.5 10.0 9.5 9.0 8.5 8.0 7.5 7.0 6.5 6.0 5.5 5.0 4.5 4.0 3.5 3.0 2.5 2.0 1.5 1.0 0.5 0.0
Chemical Shift (ppm)

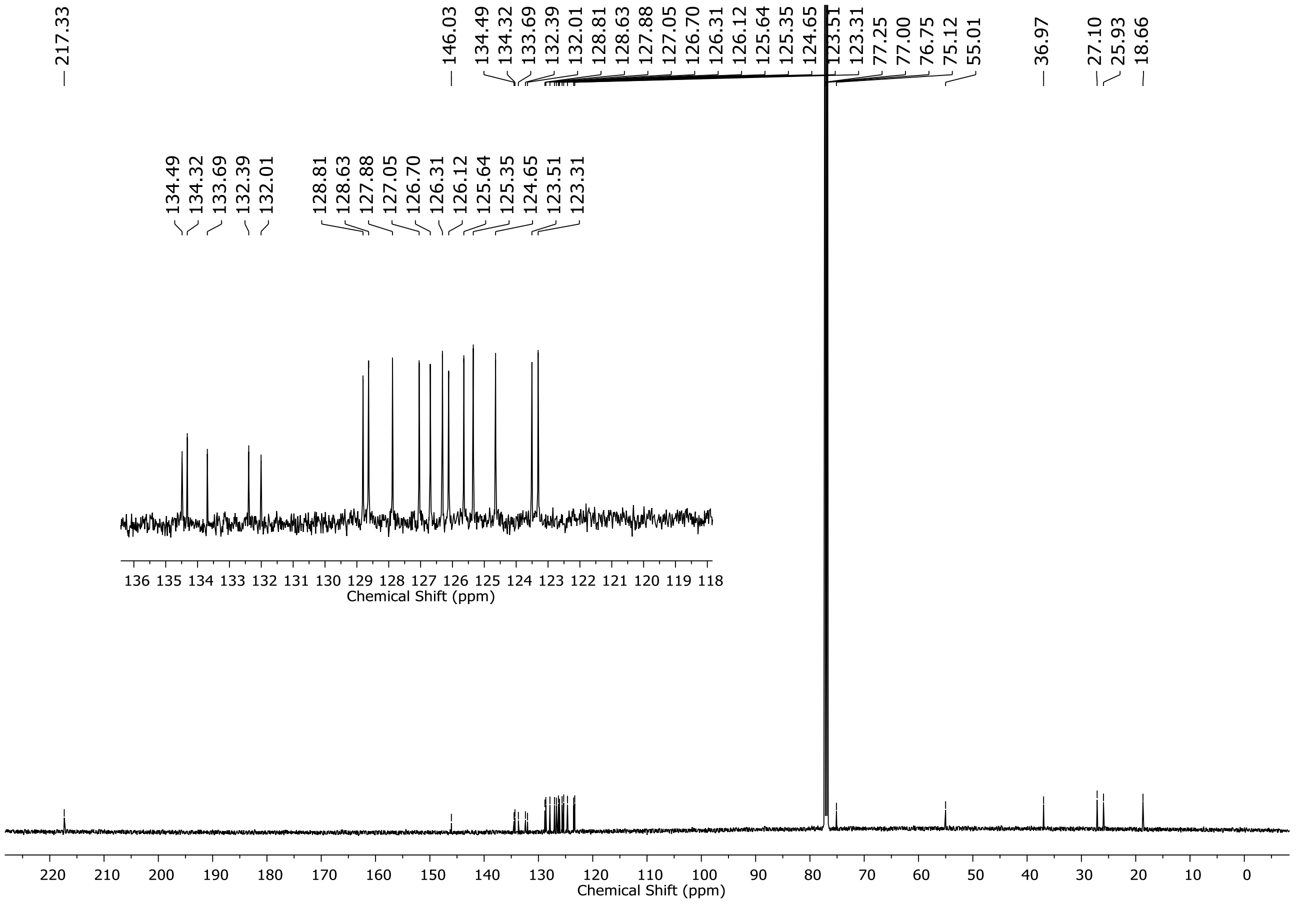
217.33
146.03
134.49
134.32
133.69
132.39
132.01
128.81
128.63
127.88
127.05
126.70
126.31
126.12
125.64
125.35
124.65
123.51
123.31
77.25
77.00
76.75
75.12
55.01
36.97
27.10
25.93
18.66
136 135 134 133 132 131 130 129 128 127 126 125 124 123 122 121 120 119 118
Chemical Shift (ppm)
220 210 200 190 180 170 160 150 140 130 120 110 100 90 80 70 60 50 40 30 20 10 0
Chemical Shift (ppm)

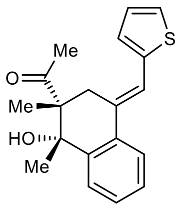
Me
O
S
Me
HO
Me

2o

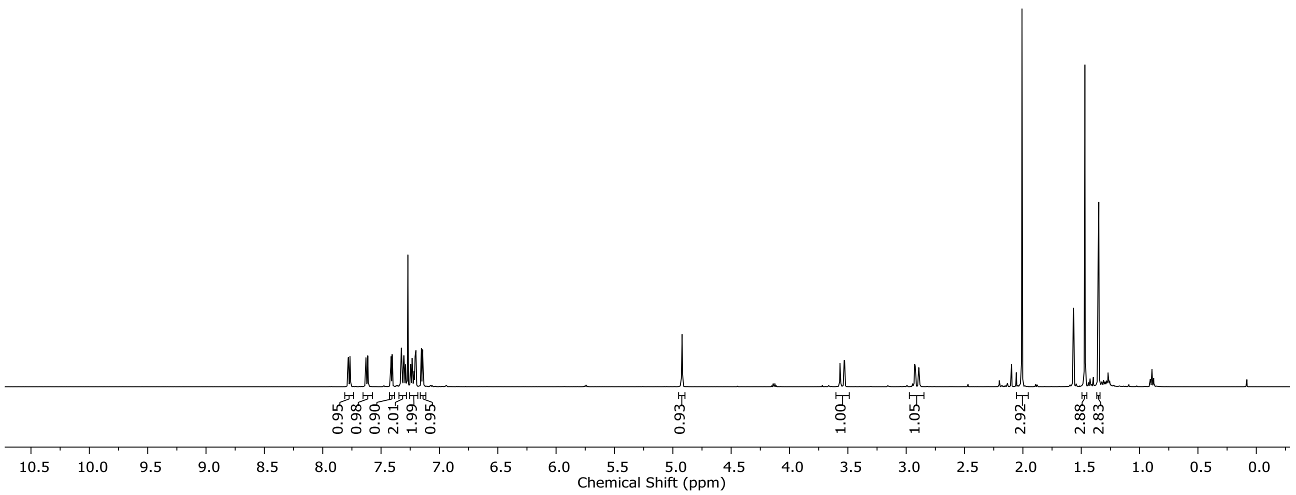
0.95
0.98
0.90
2.01
1.99
0.95
0.93
1.00
1.05
2.92
2.88
2.83
10.5 10.0 9.5 9.0 8.5 8.0 7.5 7.0 6.5 6.0 5.5 5.0 4.5 4.0 3.5 3.0 2.5 2.0 1.5 1.0 0.5 0.0
Chemical Shift (ppm)

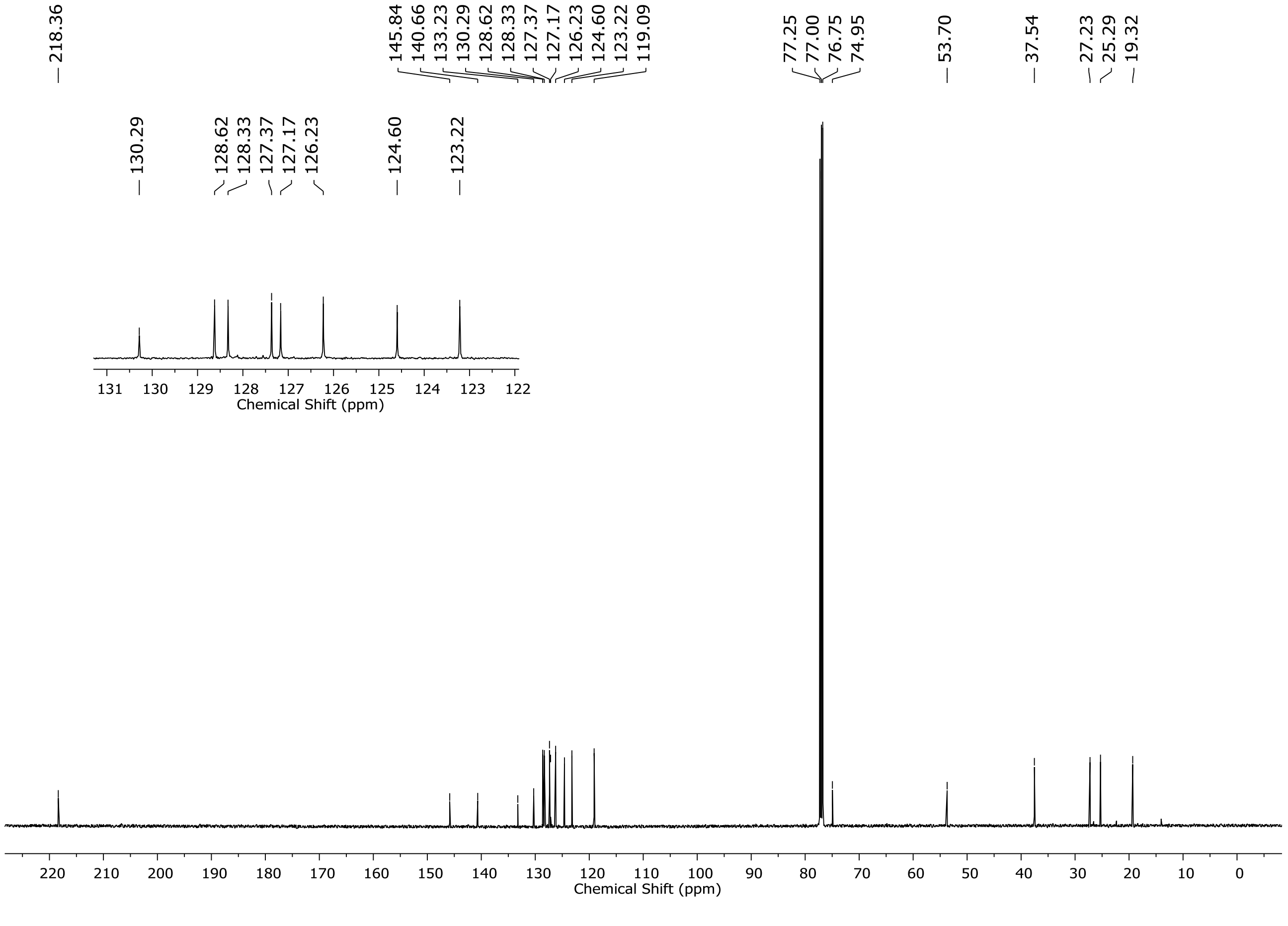
218.36
145.84
140.66
133.23
130.29
128.62
128.33
127.37
127.17
126.23
124.60
123.22
119.09
77.25
77.00
76.75
74.95
53.70
37.54
27.23
25.29
19.32
130.29
128.62
128.33
127.37
127.17
126.23
124.60
123.22
131 130 129 128 127 126 125 124 123 122
Chemical Shift (ppm)
220 210 200 190 180 170 160 150 140 130 120 110 100 90 80 70 60 50 40 30 20 10 0
Chemical Shift (ppm)

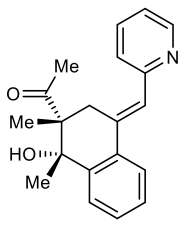
N
Me
O
Me
HO
Me

2p

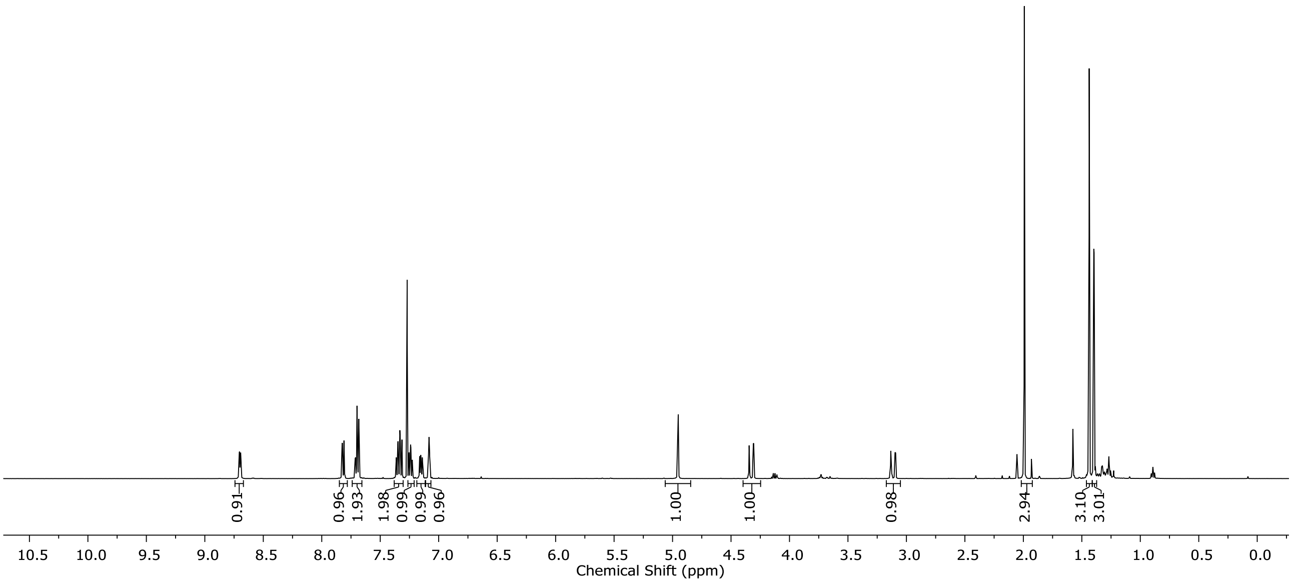
0.91
0.96
1.93
1.98
0.99
0.99
0.99
0.96
1.00
1.00
0.98
2.94
3.10
3.01
10.5 10.0 9.5 9.0 8.5 8.0 7.5 7.0 6.5 6.0 5.5 5.0 4.5 4.0 3.5 3.0 2.5 2.0 1.5 1.0 0.5 0.0
Chemical Shift (ppm)

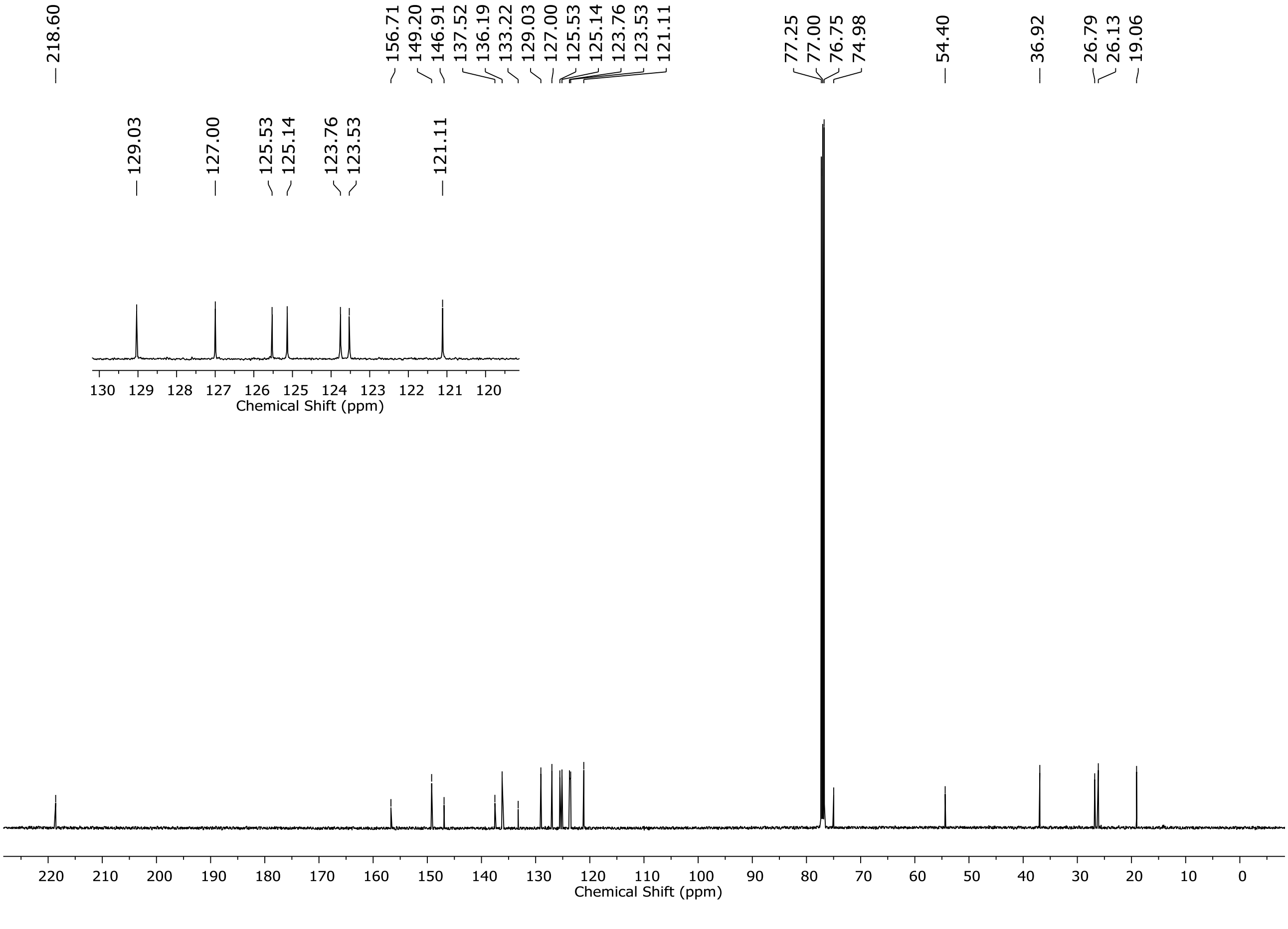
218.60
156.71
149.20
146.91
137.52
136.19
133.22
129.03
127.00
125.53
125.14
123.76
123.53
121.11
77.25
77.00
76.75
74.98
54.40
36.92
26.79
26.13
19.06
129.03
127.00
125.53
125.14
123.76
123.53
121.11
130 129 128 127 126 125 124 123 122 121 120
Chemical Shift (ppm)
220 210 200 190 180 170 160 150 140 130 120 110 100 90 80 70 60 50 40 30 20 10 0
Chemical Shift (ppm)

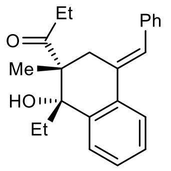
Et
Ph
O
Me
HO
Et

2q

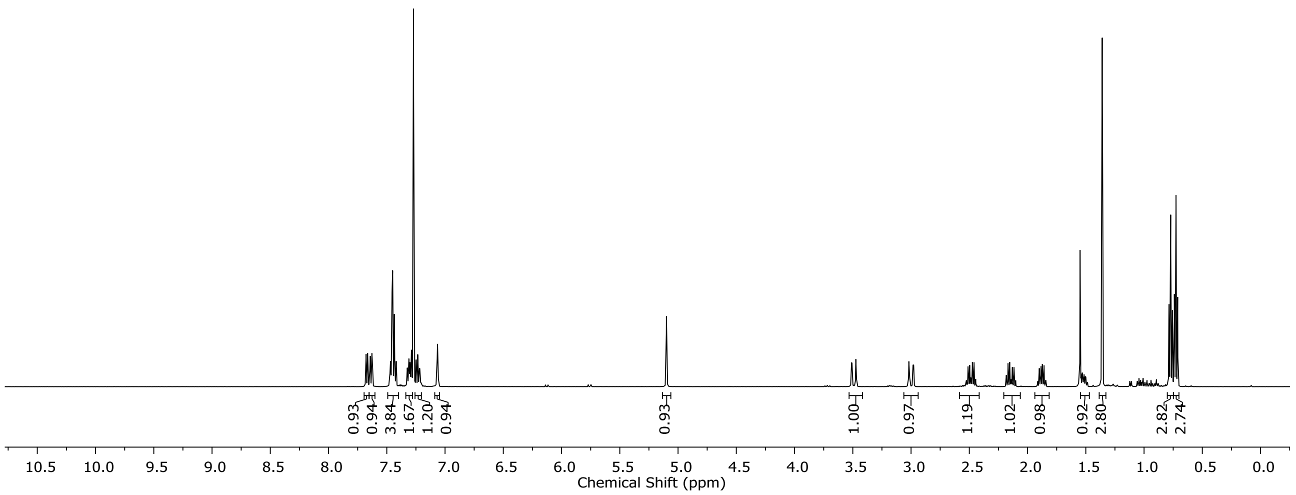
0.93
0.94
3.84
1.67
1.20
0.94
0.93
1.00
0.97
1.19
1.02
0.98
0.92
2.80
2.82
2.74
10.5 10.0 9.5 9.0 8.5 8.0 7.5 7.0 6.5 6.0 5.5 5.0 4.5 4.0 3.5 3.0 2.5 2.0 1.5 1.0 0.5 0.0
Chemical Shift (ppm)

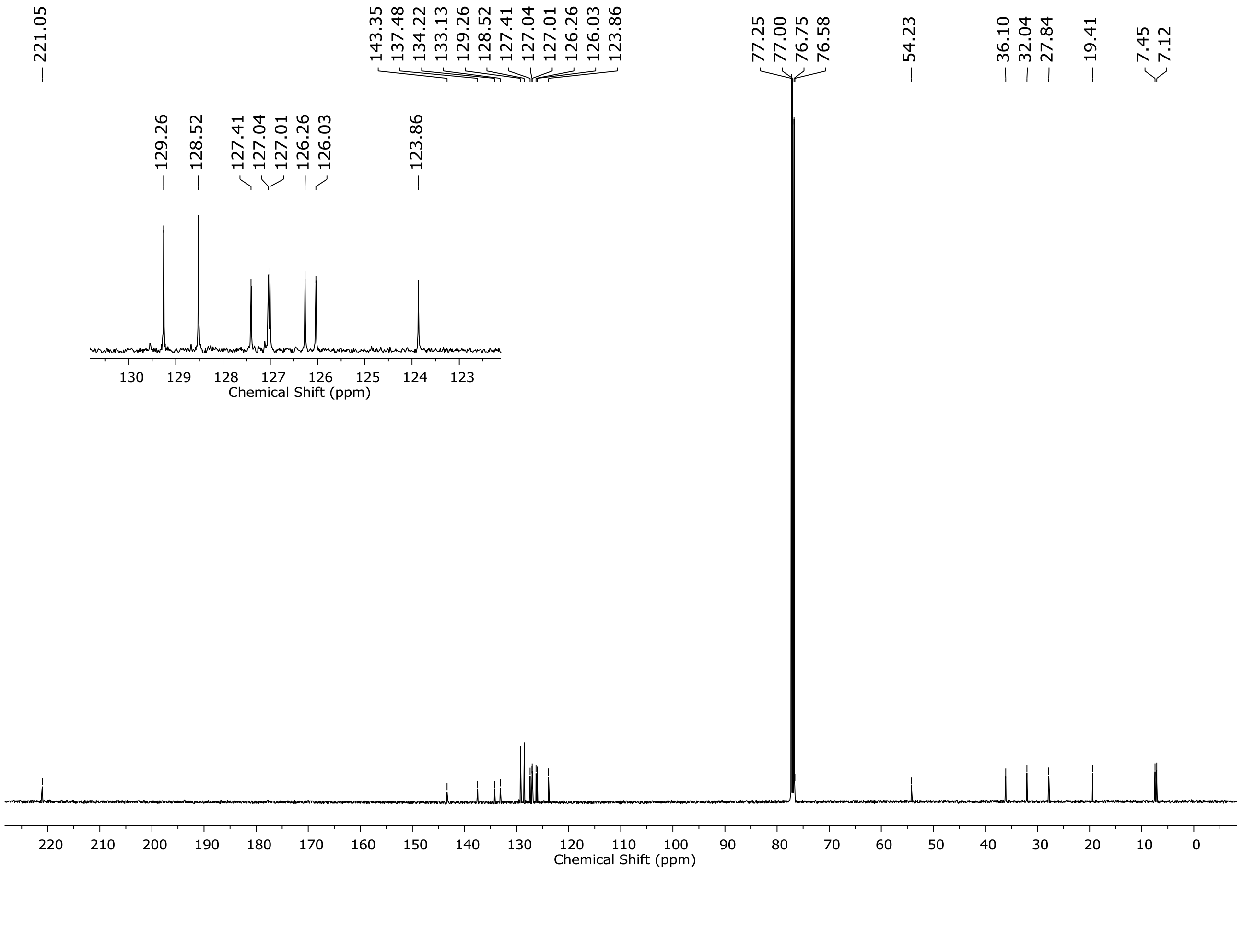
221.05
143.35
137.48
134.22
133.13
129.26
128.52
127.41
127.04
127.01
126.26
126.03
123.86
77.25
77.00
76.75
76.58
54.23
36.10
32.04
27.84
19.41
7.45
7.12
129.26
128.52
127.41
127.04
127.01
126.26
126.03
123.86
130 129 128 127 126 125 124 123
Chemical Shift (ppm)
220 210 200 190 180 170 160 150 140 130 120 110 100 90 80 70 60 50 40 30 20 10 0
Chemical Shift (ppm)

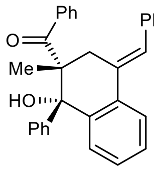
Ph
Ph
O
Me
HO
Ph

2r

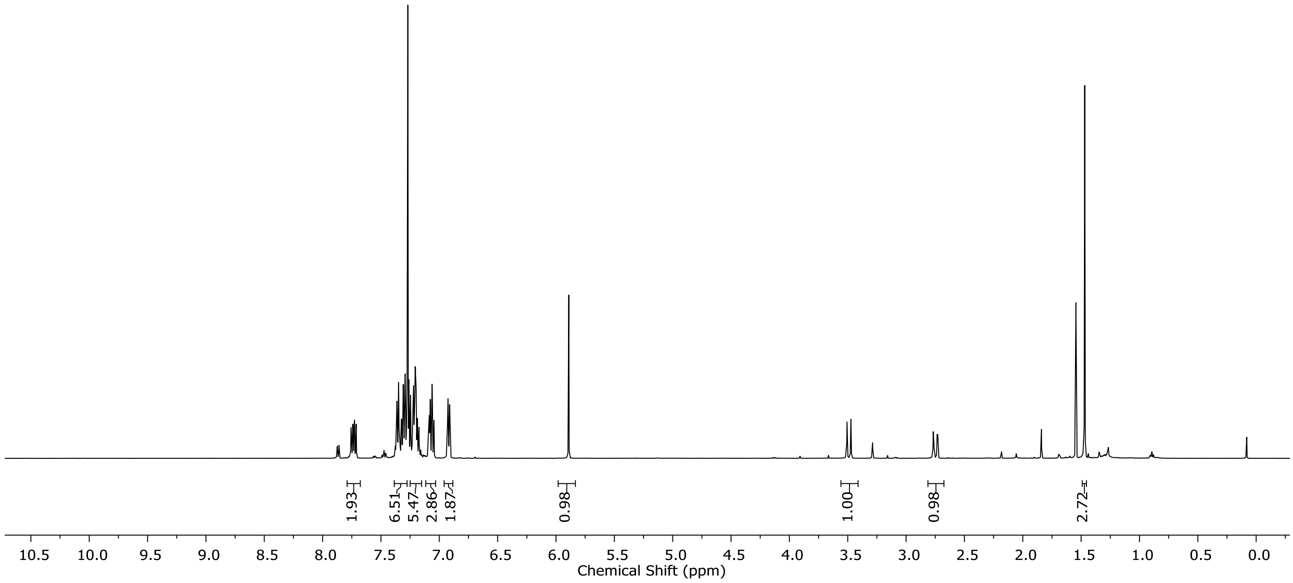
1.93
6.51
5.47
2.86
1.87
0.98
1.00
0.98
2.72
10.5 10.0 9.5 9.0 8.5 8.0 7.5 7.0 6.5 6.0 5.5 5.0 4.5 4.0 3.5 3.0 2.5 2.0 1.5 1.0 0.5 0.0
Chemical Shift (ppm)

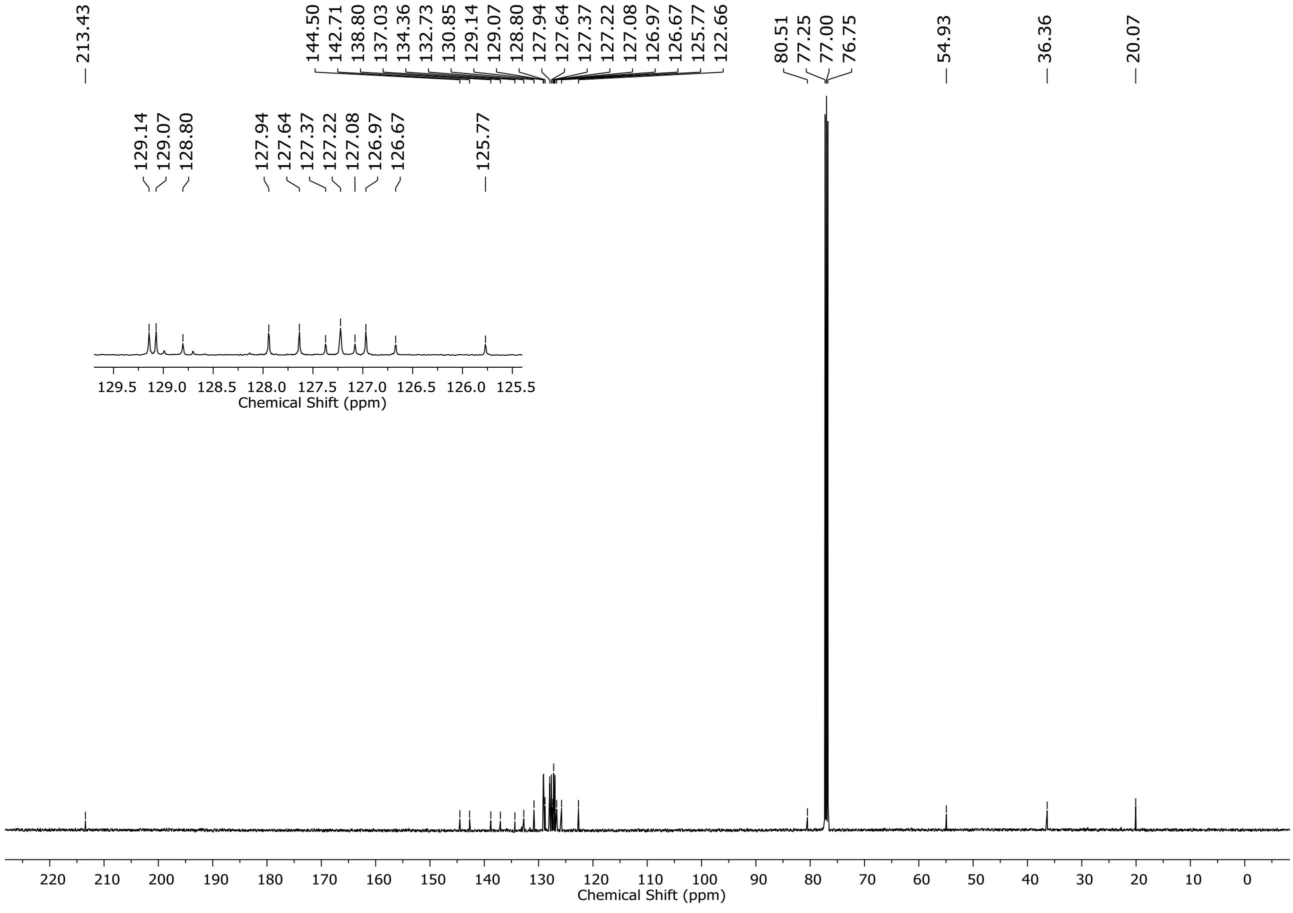
213.43
144.50
142.71
138.80
137.03
134.36
132.73
130.85
129.14
129.07
128.80
127.94
127.64
127.37
127.22
127.08
126.97
126.67
125.77
122.66
80.51
77.25
77.00
76.75
54.93
36.36
20.07
129.14
129.07
128.80
127.94
127.64
127.37
127.22
127.08
126.97
126.67
125.77
129.5 129.0 128.5 128.0 127.5 127.0 126.5 126.0 125.5
Chemical Shift (ppm)
220 210 200 190 180 170 160 150 140 130 120 110 100 90 80 70 60 50 40 30 20 10 0
Chemical Shift (ppm)

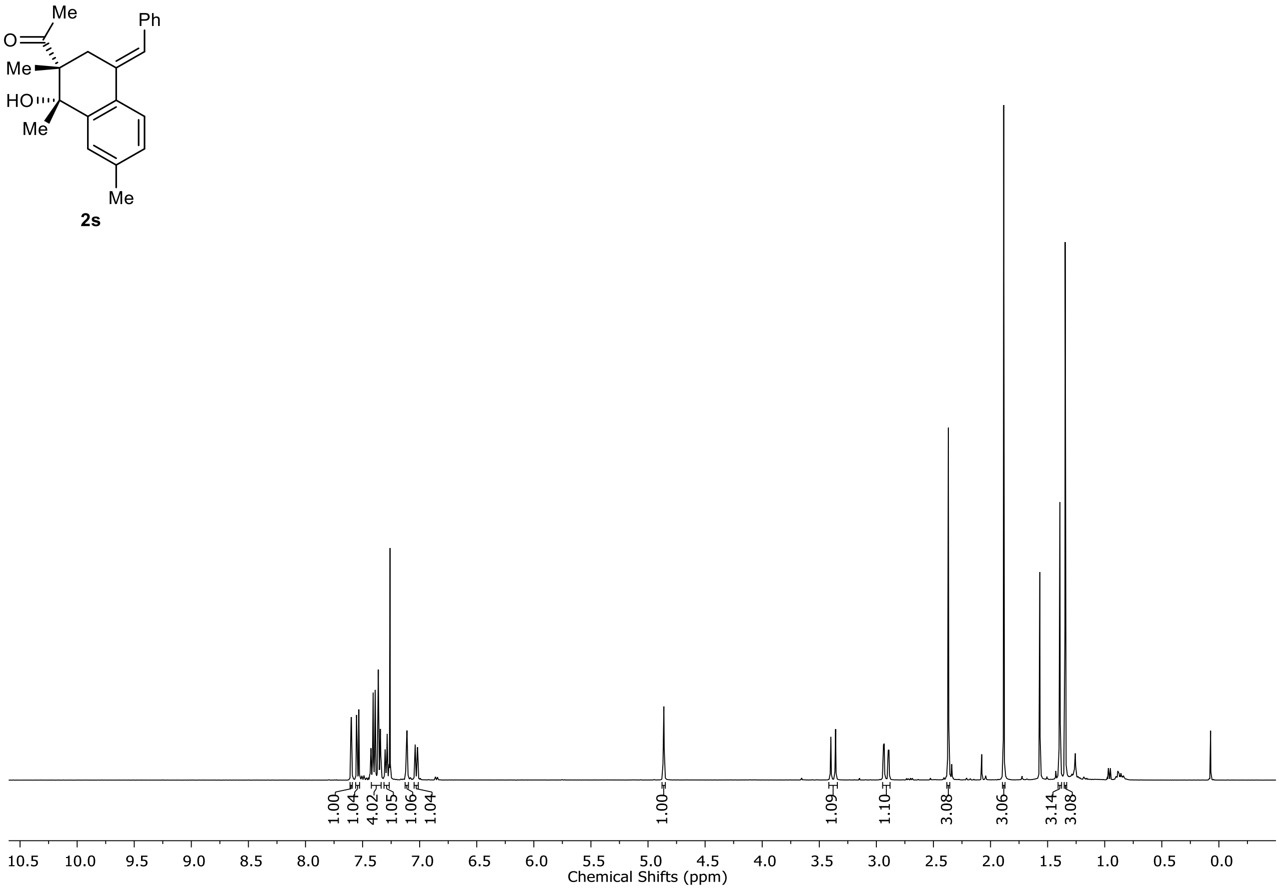
Me
Ph
O
Me
HO
Me
Me

2s

1.00
1.04
4.02
1.05
1.06
1.04
1.00
1.09
1.10
3.08
3.06
3.14
3.08
10.5 10.0 9.5 9.0 8.5 8.0 7.5 7.0 6.5 6.0 5.5 5.0 4.5 4.0 3.5 3.0 2.5 2.0 1.5 1.0 0.5 0.0
Chemical Shifts (ppm)

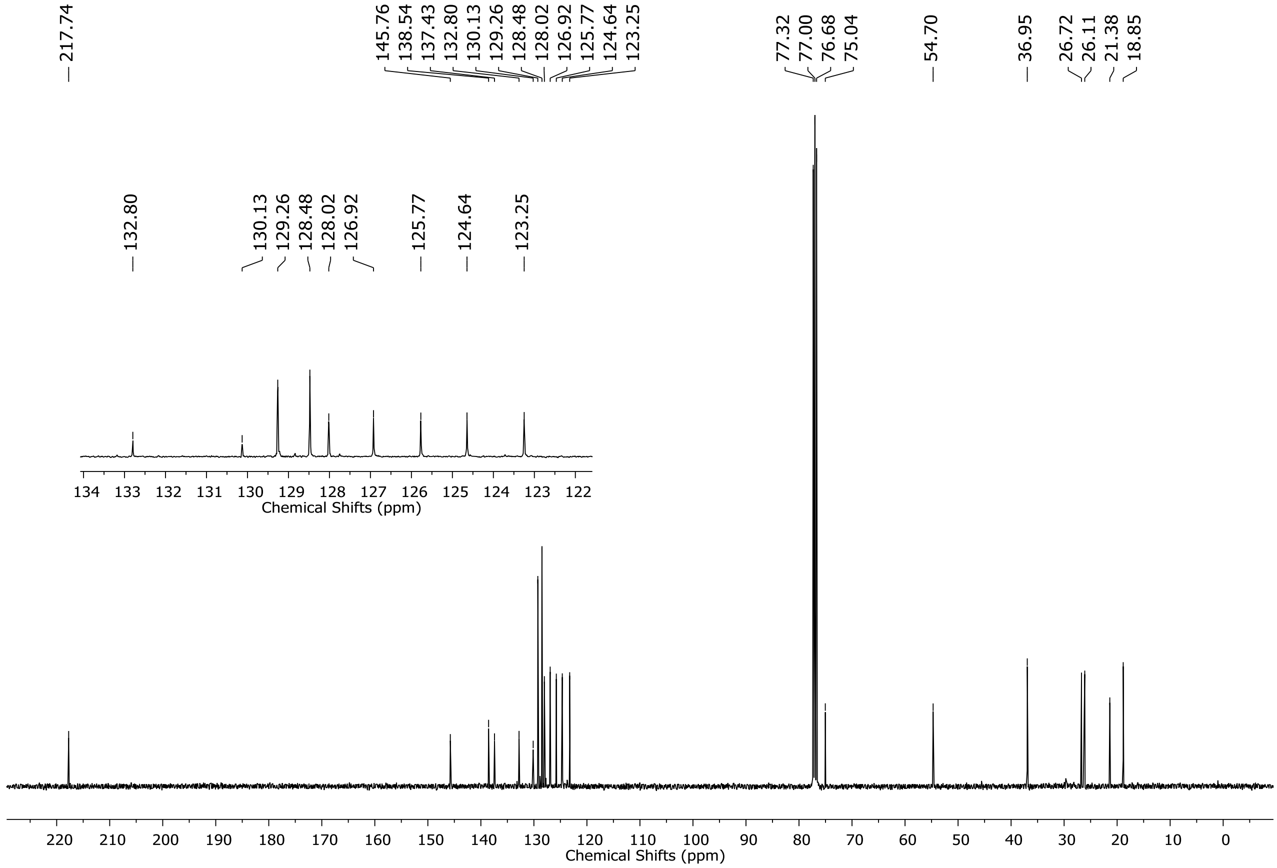
217.74
145.76
138.54
137.43
132.80
130.13
129.26
128.48
128.02
126.92
125.77
124.64
123.25
77.32
77.00
76.68
75.04
54.70
36.95
26.72
26.11
21.38
18.85
132.80
130.13
129.26
128.48
128.02
126.92
125.77
124.64
123.25
134 133 132 131 130 129 128 127 126 125 124 123 122
Chemical Shifts (ppm)
220 210 200 190 180 170 160 150 140 130 120 110 100 90 80 70 60 50 40 30 20 10 0
Chemical Shifts (ppm)

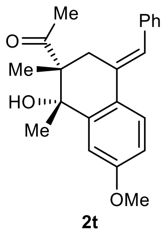
Me
Ph
O
Me
HO
Me
OMe

2t

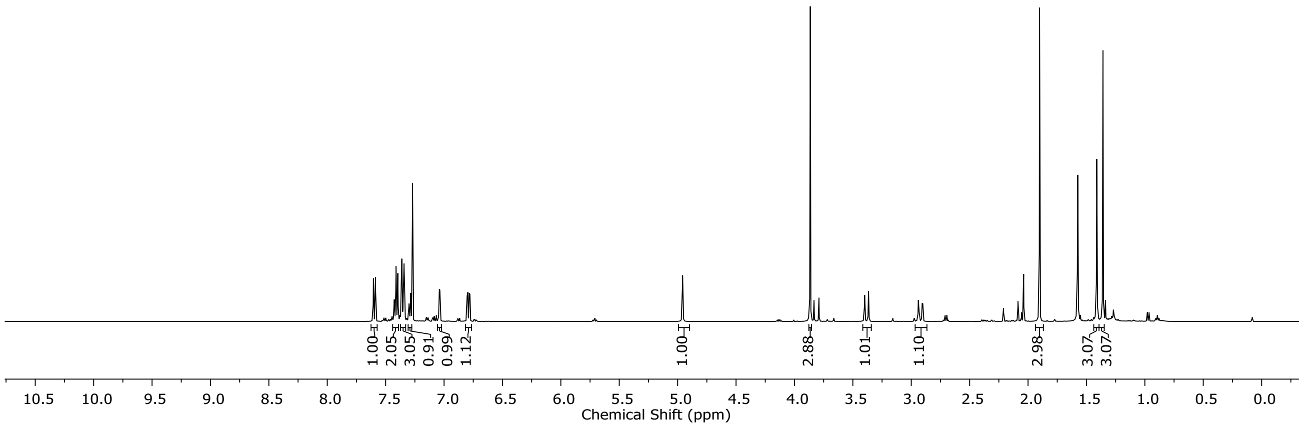
1.00
2.05
3.05
0.91
0.99
1.12
1.00
2.88
1.01
1.10
2.98
3.07
3.07
10.5 10.0 9.5 9.0 8.5 8.0 7.5 7.0 6.5 6.0 5.5 5.0 4.5 4.0 3.5 3.0 2.5 2.0 1.5 1.0 0.5 0.0
Chemical Shift (ppm)

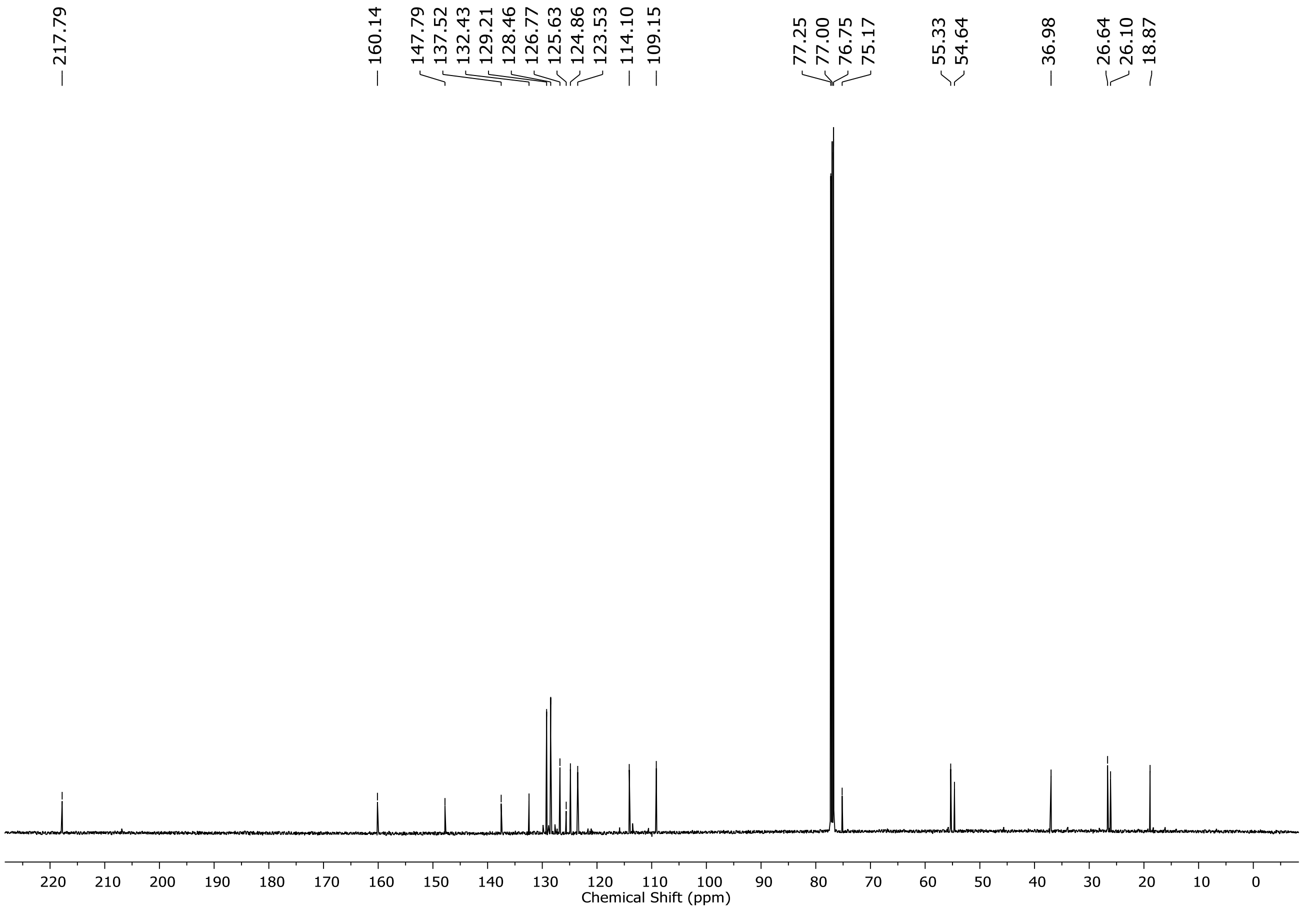
217.79
160.14
147.79
137.52
132.43
129.21
128.46
126.77
125.63
124.86
123.53
114.10
109.15
77.25
77.00
76.75
75.17
55.33
54.64
36.98
26.64
26.10
18.87
220 210 200 190 180 170 160 150 140 130 120 110 100 90 80 70 60 50 40 30 20 10 0
Chemical Shift (ppm)

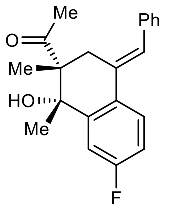
Me
Ph
O
Me
HO
Me
F

2u

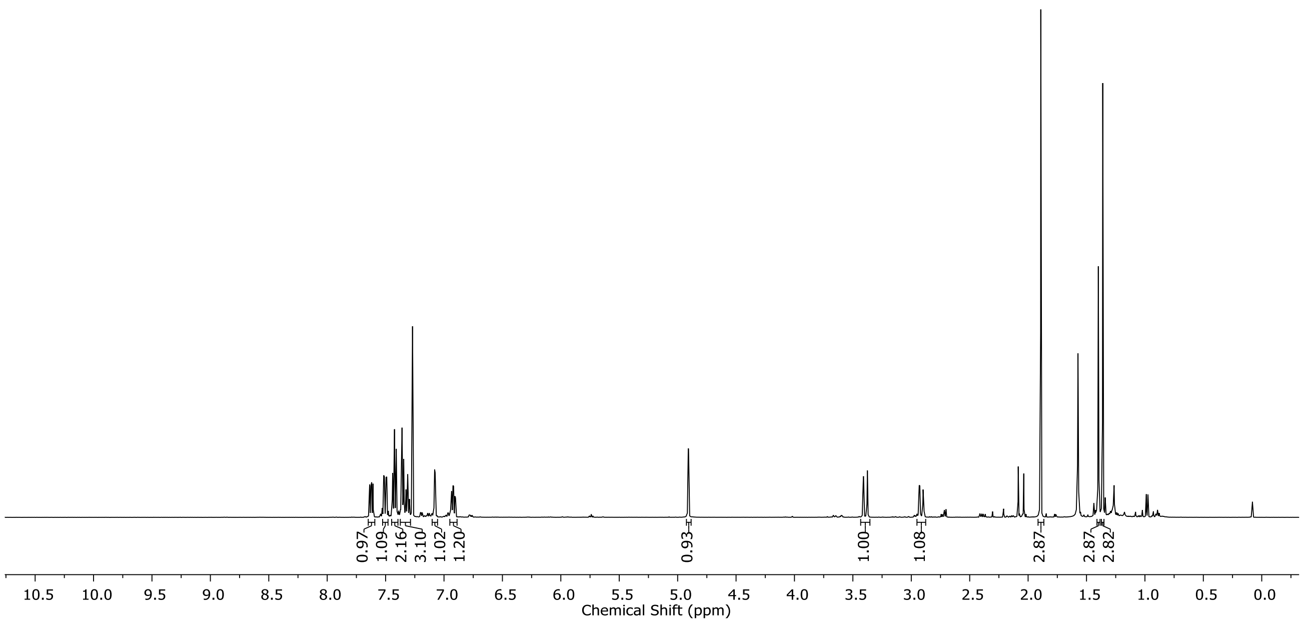
0.97
1.09
2.16
3.10
1.02
1.20
0.93
1.00
1.08
2.87
2.87
2.82
10.5 10.0 9.5 9.0 8.5 8.0 7.5 7.0 6.5 6.0 5.5 5.0 4.5 4.0 3.5 3.0 2.5 2.0 1.5 1.0 0.5 0.0
Chemical Shift (ppm)

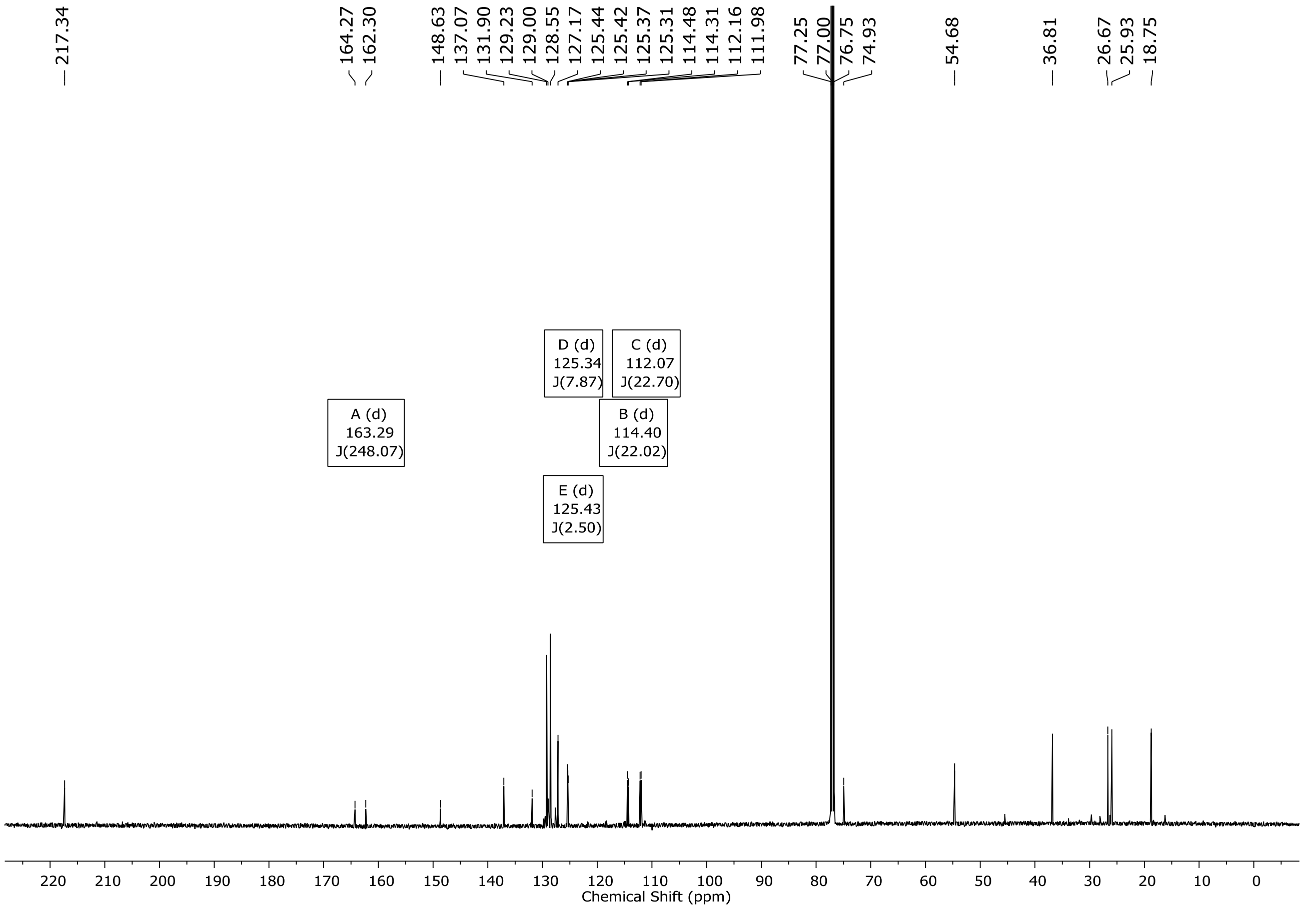
217.34
164.27
162.30
148.63
137.07
131.90
129.23
129.00
128.55
127.17
125.44
125.42
125.37
125.31
114.48
114.31
112.16
111.98
77.25
77.00
76.75
74.93
54.68
36.81
26.67
25.93
18.75
A (d) 163.29 J(248.07)
D (d) 125.34 J(7.87)
C (d) 112.07 J(22.70)
B (d) 114.40 J(22.02)
E (d) 125.43 J(2.50)
220 210 200 190 180 170 160 150 140 130 120 110 100 90 80 70 60 50 40 30 20 10 0
Chemical Shift (ppm)

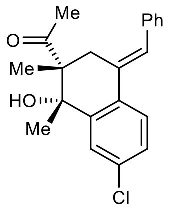
Me
Ph
O
Me
HO
Me
Cl

2v

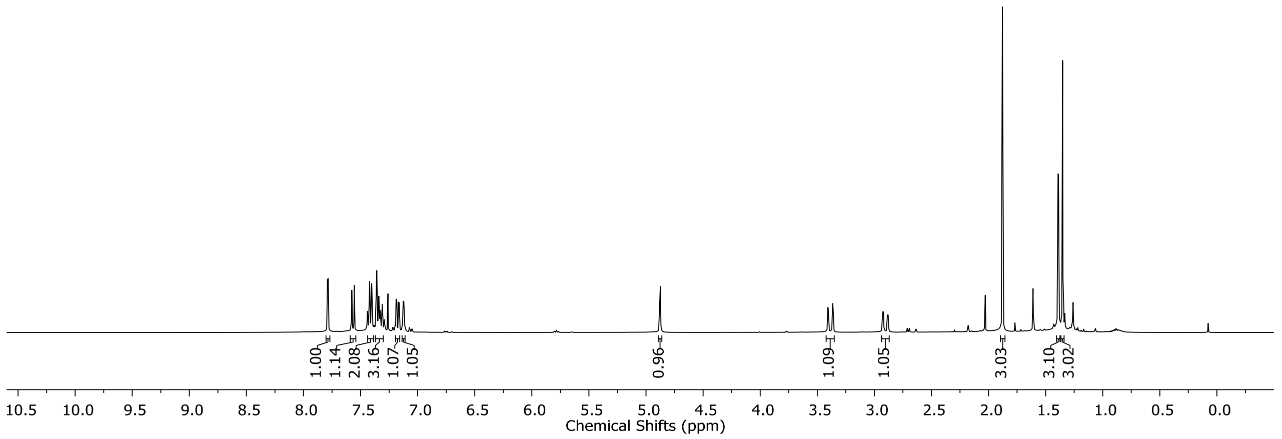
1.00
1.14
2.08
3.16
1.07
1.05
0.96
1.09
1.05
3.03
3.10
3.02
Chemical Shifts (ppm)

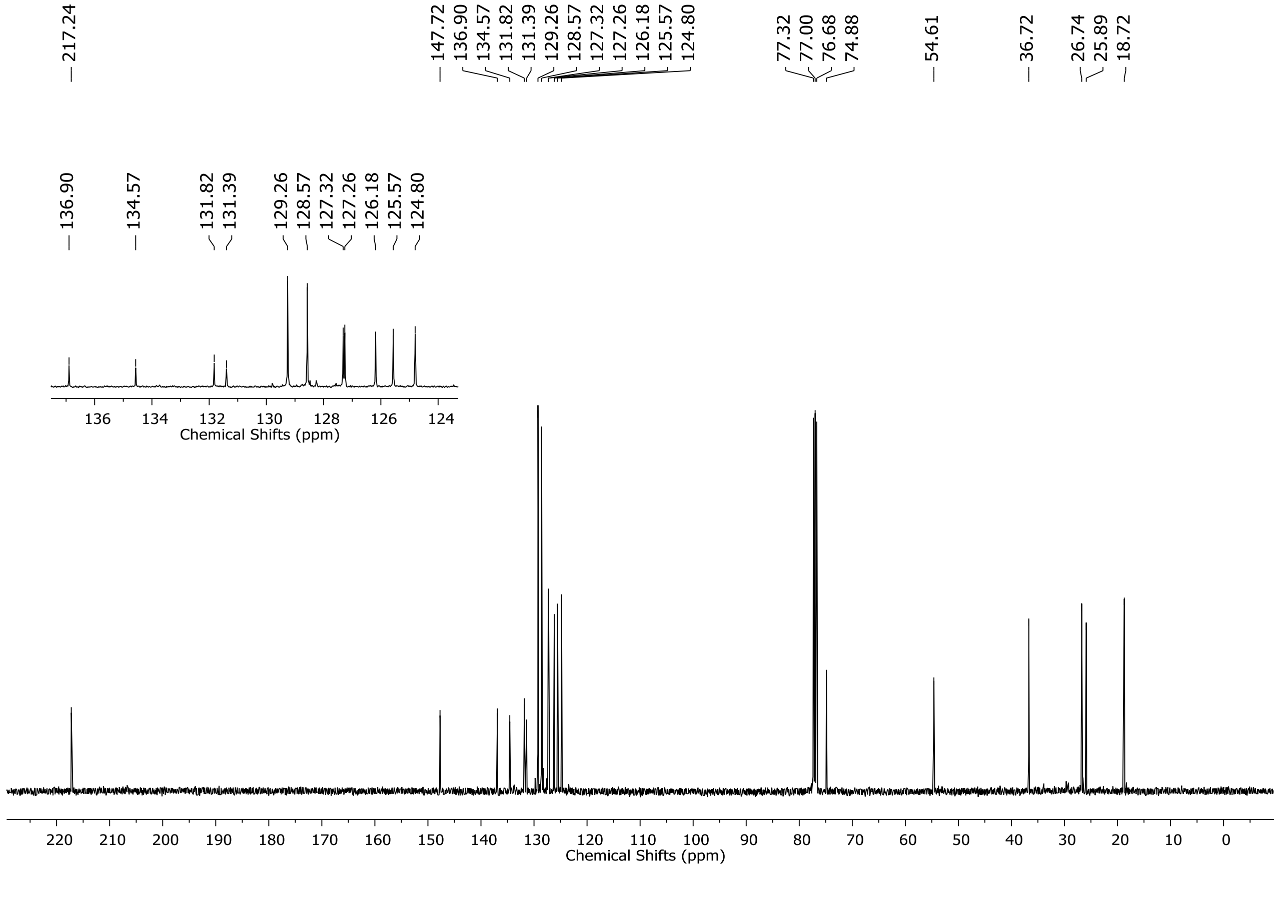
217.24
147.72
136.90
134.57
131.82
131.39
129.26
128.57
127.32
127.26
126.18
125.57
124.80
77.32
77.00
76.68
74.88
54.61
36.72
26.74
25.89
18.72
Chemical Shifts (ppm)

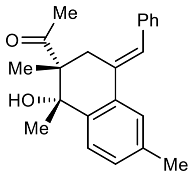
Me
Ph
O
Me
HO
Me
Me

2x

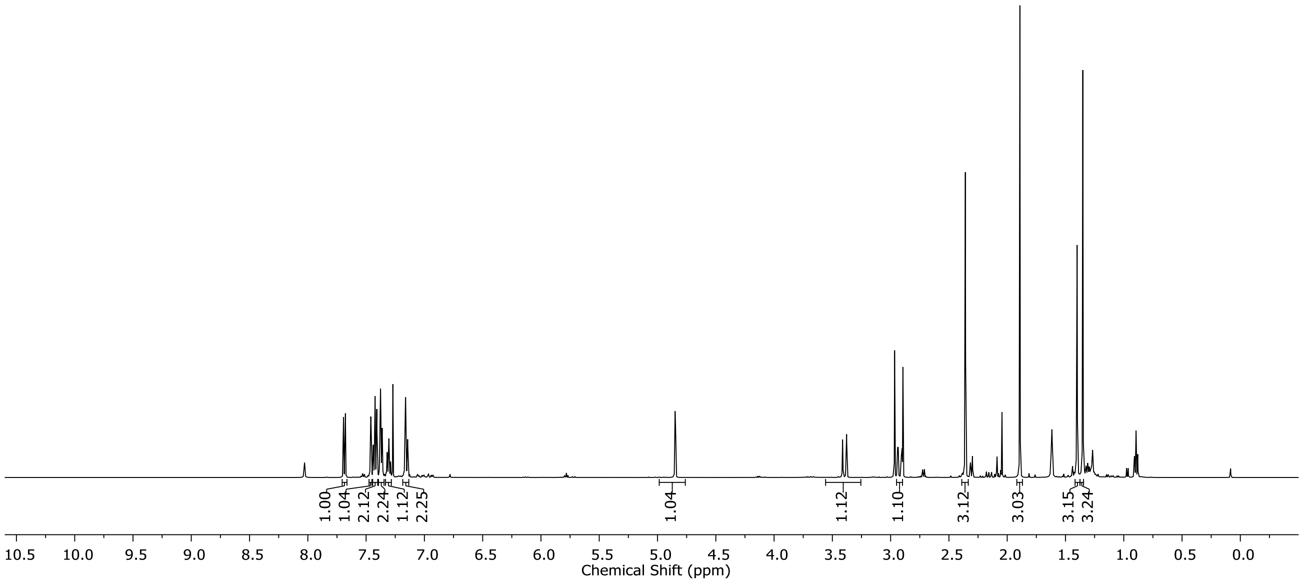
1.00
1.04
2.12
2.24
1.12
2.25
1.04
1.12
1.10
3.12
3.03
3.15
3.24
10.5 10.0 9.5 9.0 8.5 8.0 7.5 7.0 6.5 6.0 5.5 5.0 4.5 4.0 3.5 3.0 2.5 2.0 1.5 1.0 0.5 0.0
Chemical Shift (ppm)

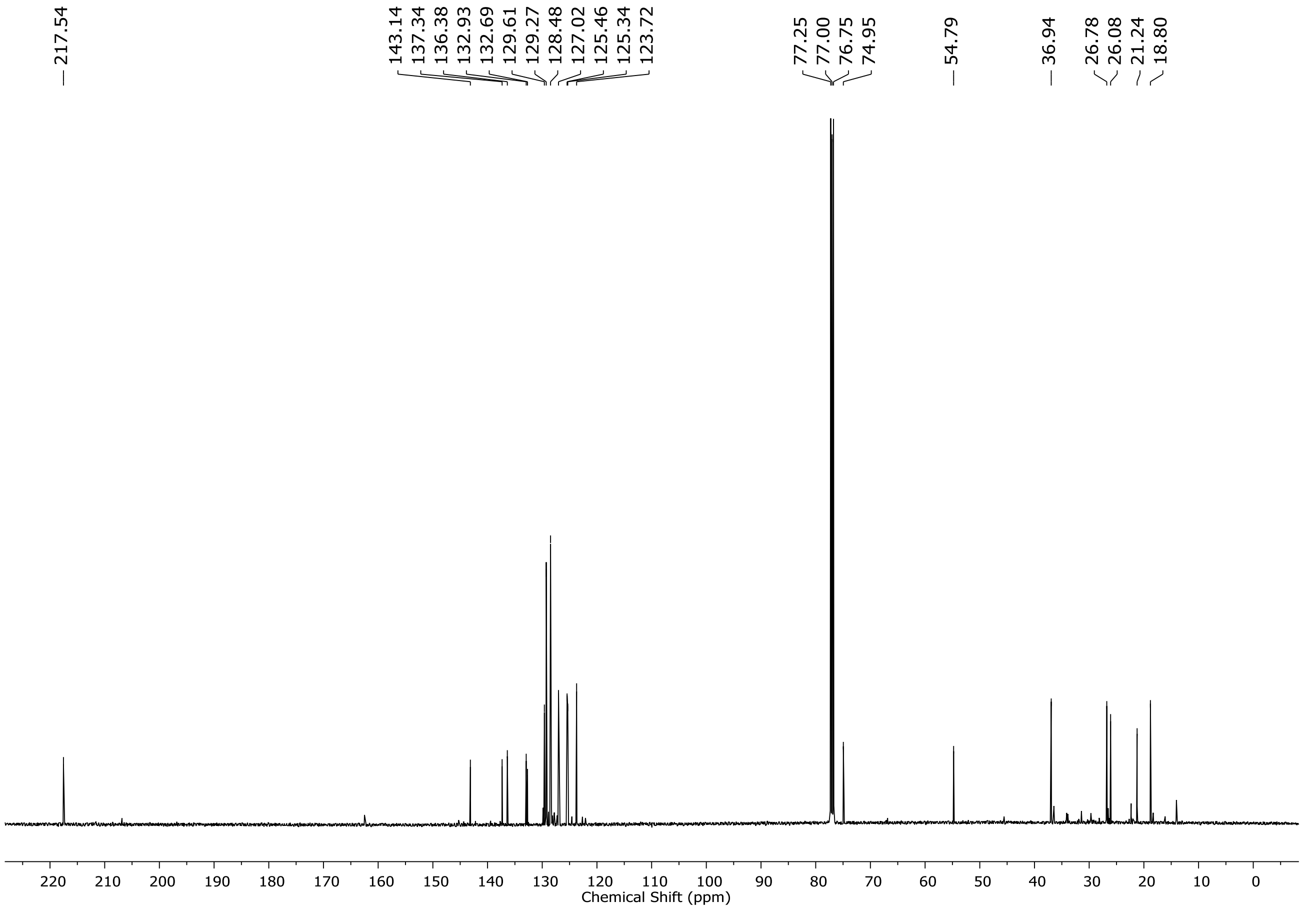
217.54
143.14
137.34
136.38
132.93
132.69
129.61
129.27
128.48
127.02
125.46
125.34
123.72
77.25
77.00
76.75
74.95
54.79
36.94
26.78
26.08
21.24
18.80
220 210 200 190 180 170 160 150 140 130 120 110 100 90 80 70 60 50 40 30 20 10 0
Chemical Shift (ppm)

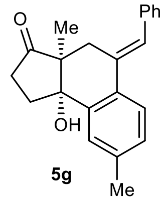
Ph
O
Me
OH
Me

5g

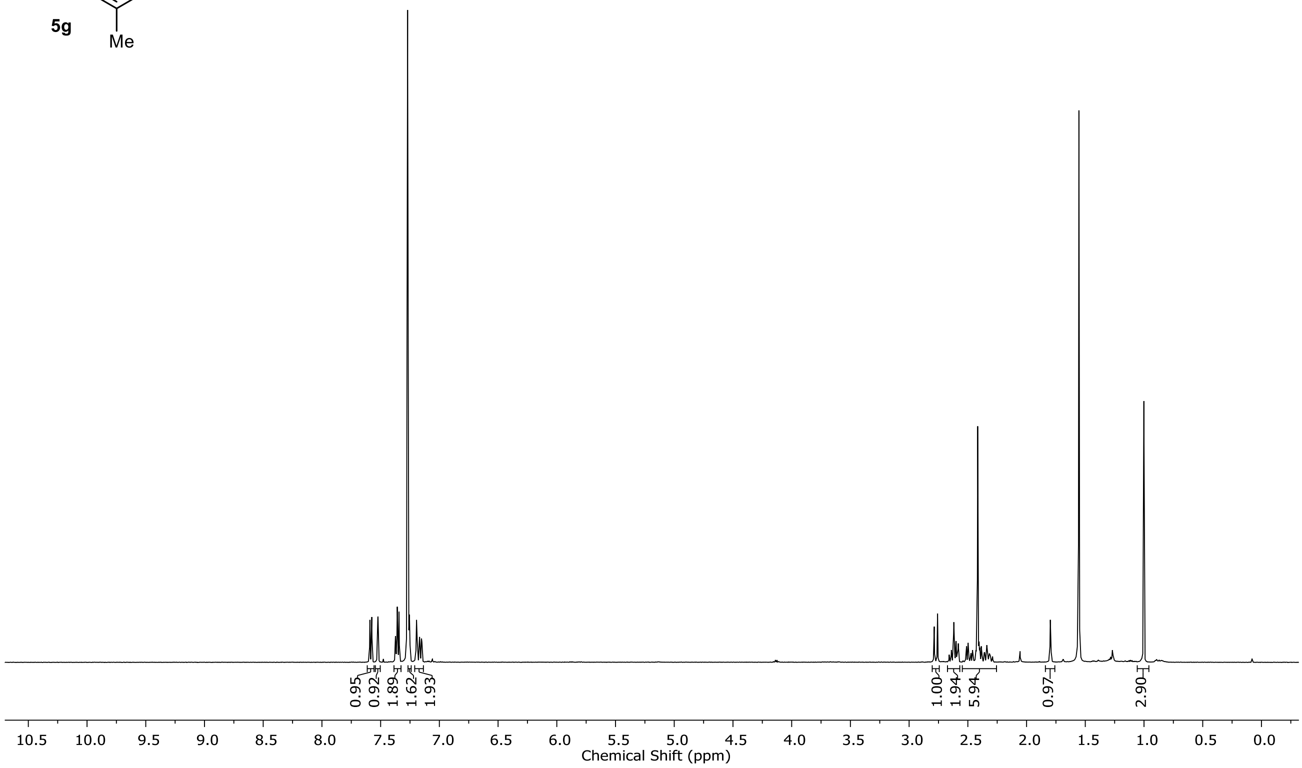
0.95
0.92
1.89
1.62
1.93
1.00
1.94
5.94
0.97
2.90
10.5 10.0 9.5 9.0 8.5 8.0 7.5 7.0 6.5 6.0 5.5 5.0 4.5 4.0 3.5 3.0 2.5 2.0 1.5 1.0 0.5 0.0
Chemical Shift (ppm)

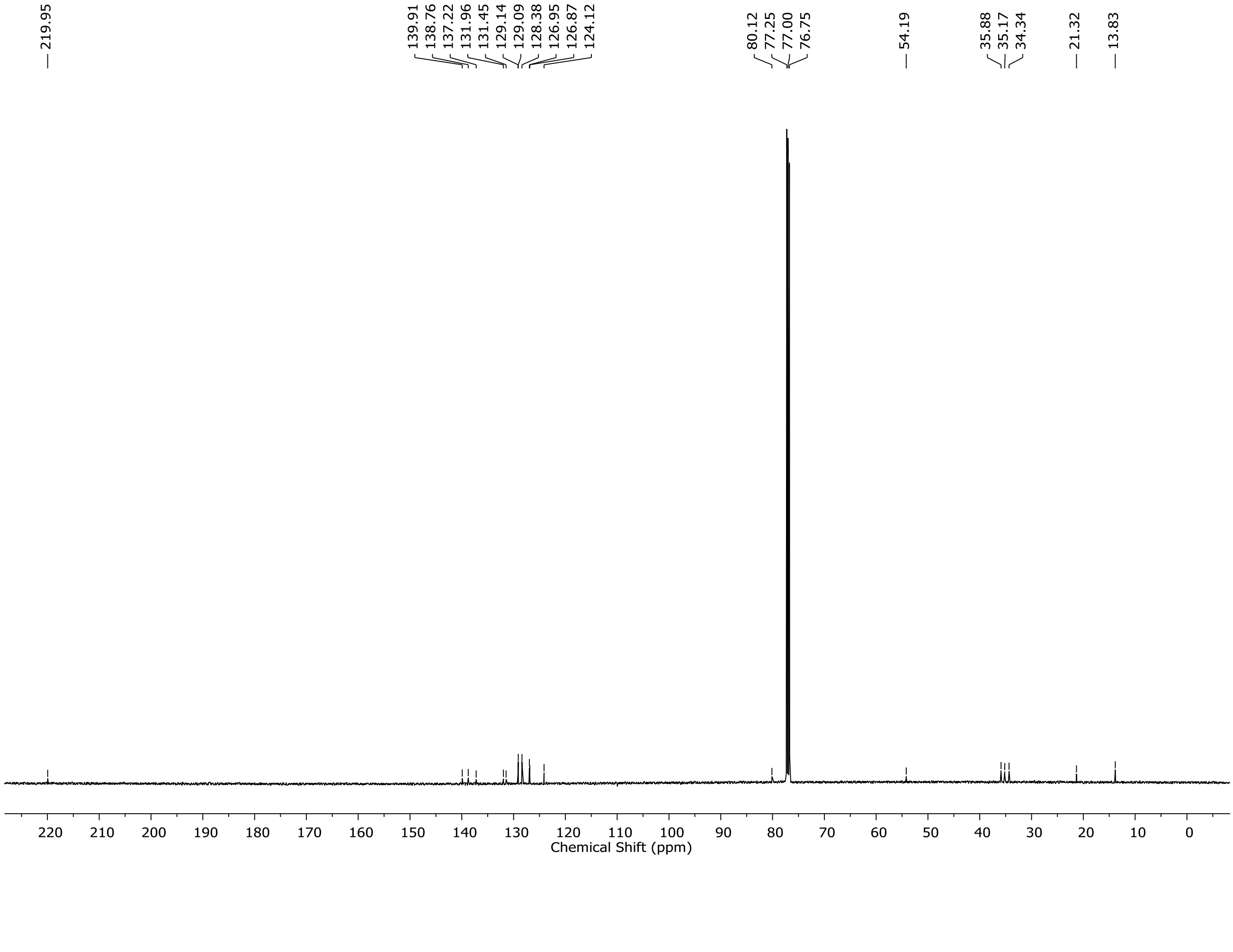
219.95
139.91
138.76
137.22
131.96
131.45
129.14
129.09
128.38
126.95
126.87
124.12
80.12
77.25
77.00
76.75
54.19
35.88
35.17
34.34
21.32
13.83
220 210 200 190 180 170 160 150 140 130 120 110 100 90 80 70 60 50 40 30 20 10 0
Chemical Shift (ppm)

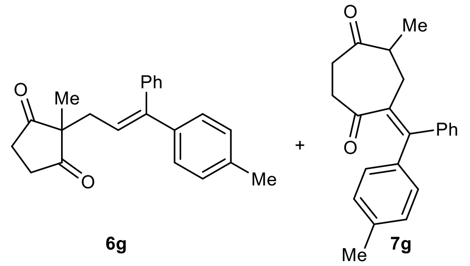

3:1 inseparable mixture

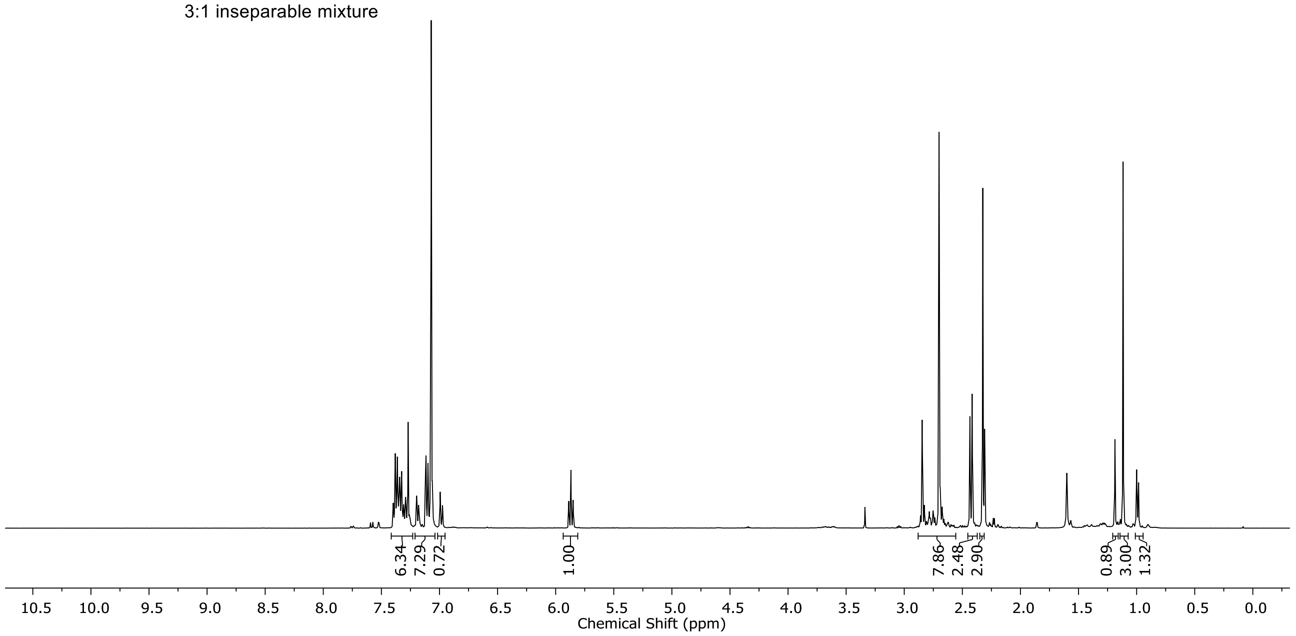

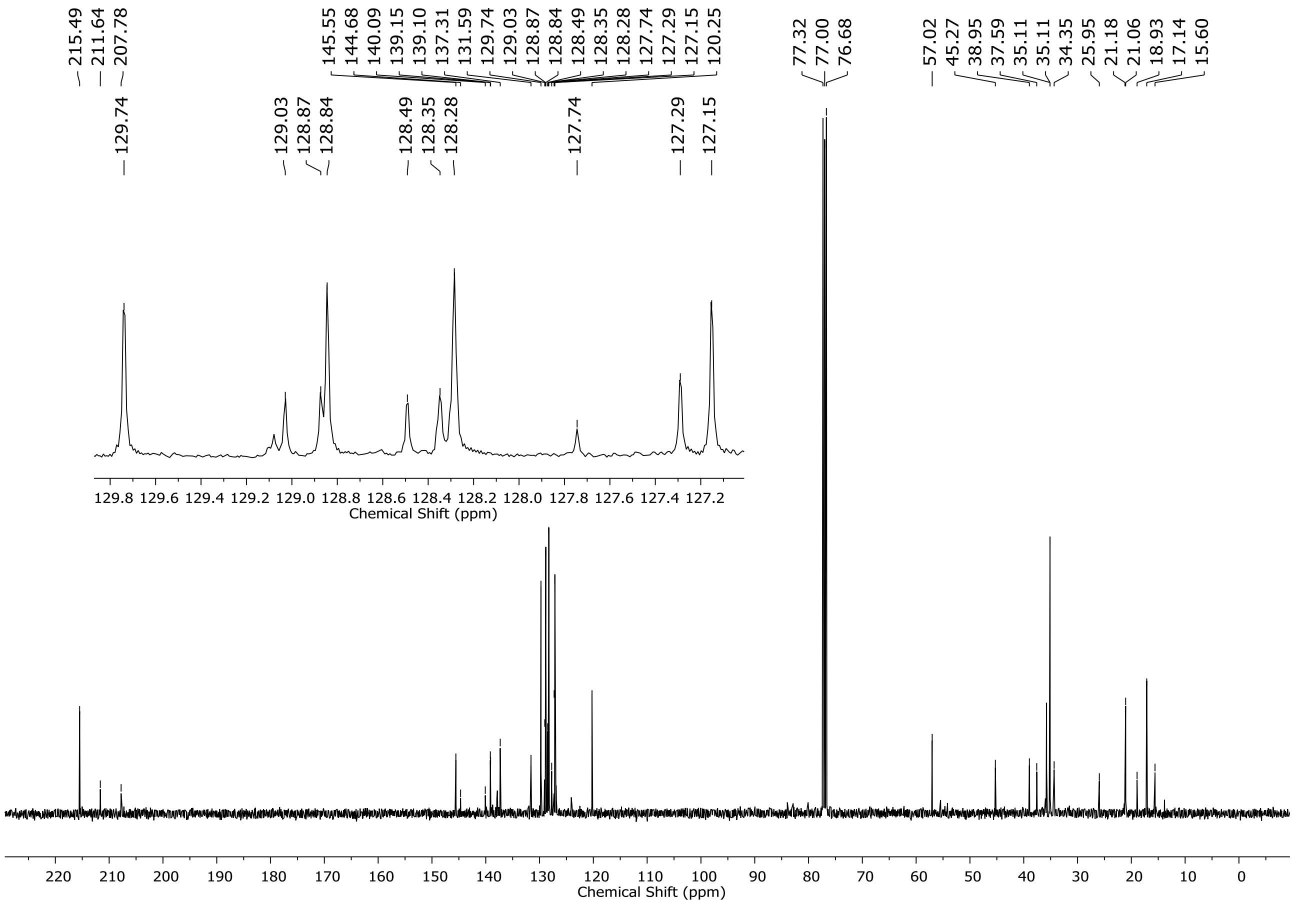

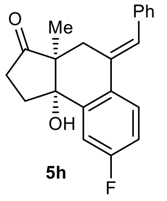
O
Me
Ph
OH
F

5h

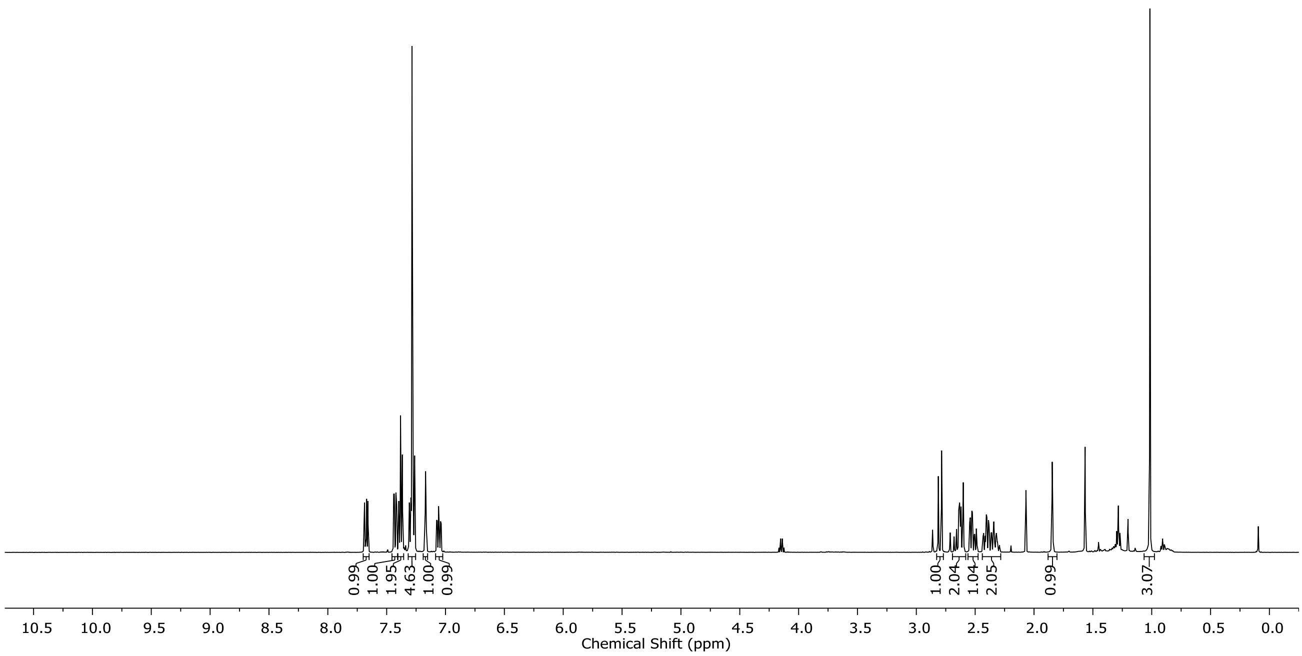
0.99
1.00
1.95
4.63
1.00
0.99
1.00
2.04
1.04
2.05
0.99
3.07
Chemical Shift (ppm)

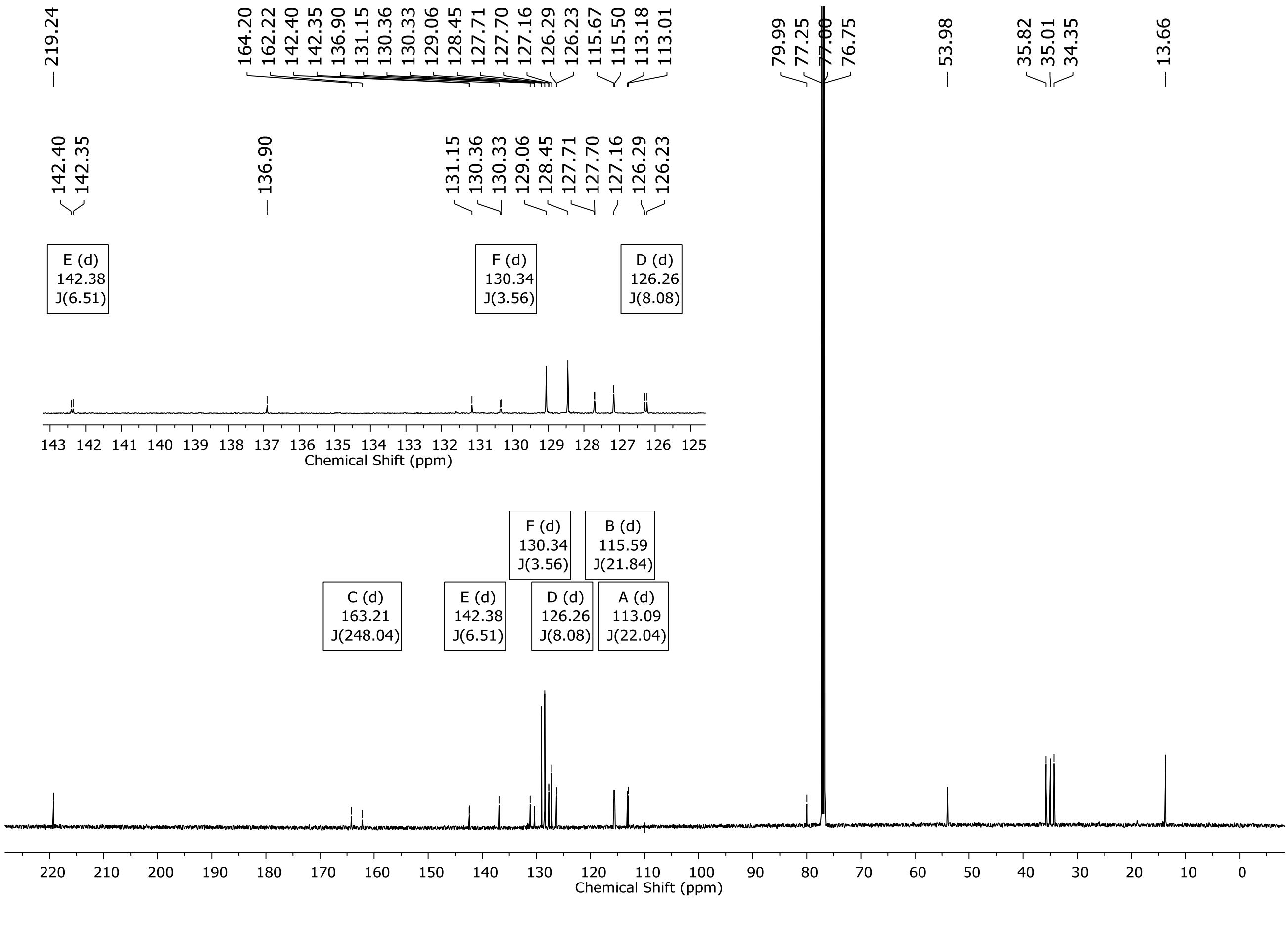
219.24
164.20
162.22
142.40
142.35
136.90
131.15
130.36
130.33
129.06
128.45
127.71
127.70
127.16
126.29
126.23
115.67
115.50
113.18
113.01
79.99
77.25
77.00
76.75
53.98
35.82
35.01
34.35
13.66
E (d) 142.38 J(6.51)
F (d) 130.34 J(3.56)
D (d) 126.26 J(8.08)
B (d) 115.59 J(21.84)
C (d) 163.21 J(248.04)
A (d) 113.09 J(22.04)
Chemical Shift (ppm)

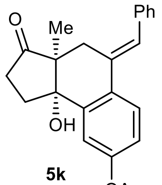
O
Me
Ph
OH
OAc

5k

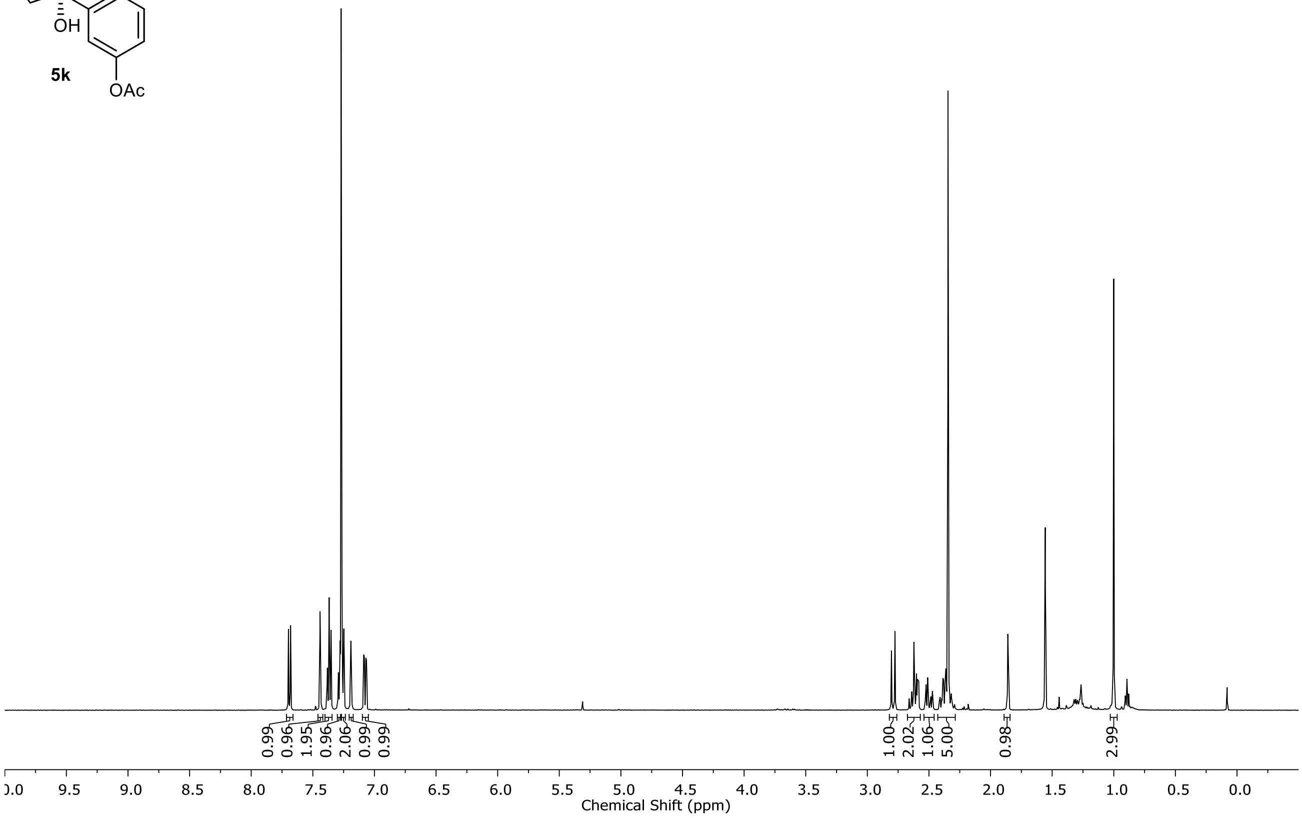
0.99
0.96
1.95
0.96
2.06
0.99
0.99
1.00
2.02
1.06
5.00
0.98
2.99
Chemical Shift (ppm)

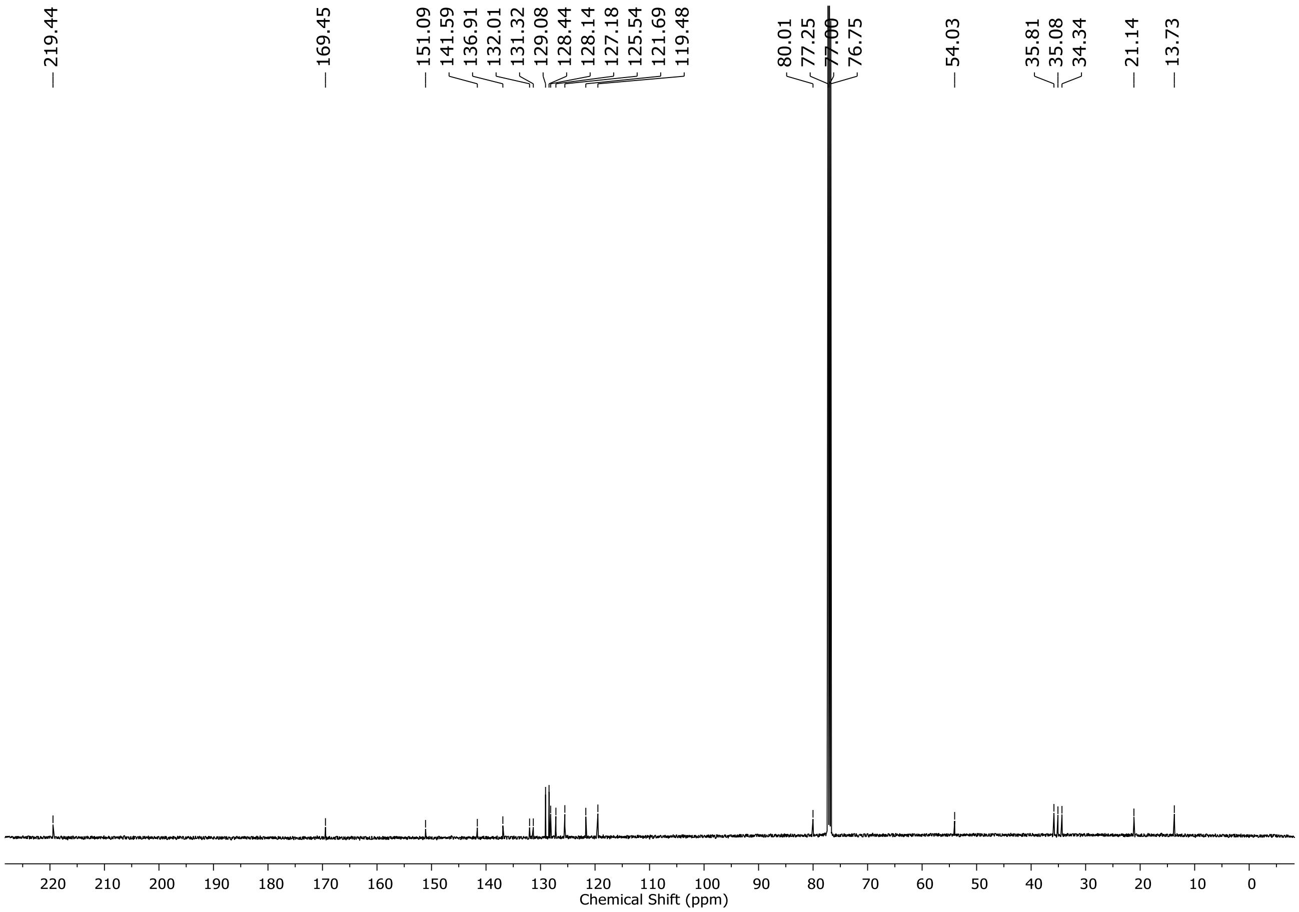
219.44
169.45
151.09
141.59
136.91
132.01
131.32
129.08
128.44
128.14
127.18
125.54
121.69
119.48
80.01
77.25
77.00
76.75
54.03
35.81
35.08
34.34
21.14
13.73
Chemical Shift (ppm)

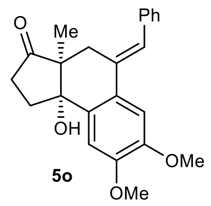
O
Me
Ph
OH
OMe
OMe

5o

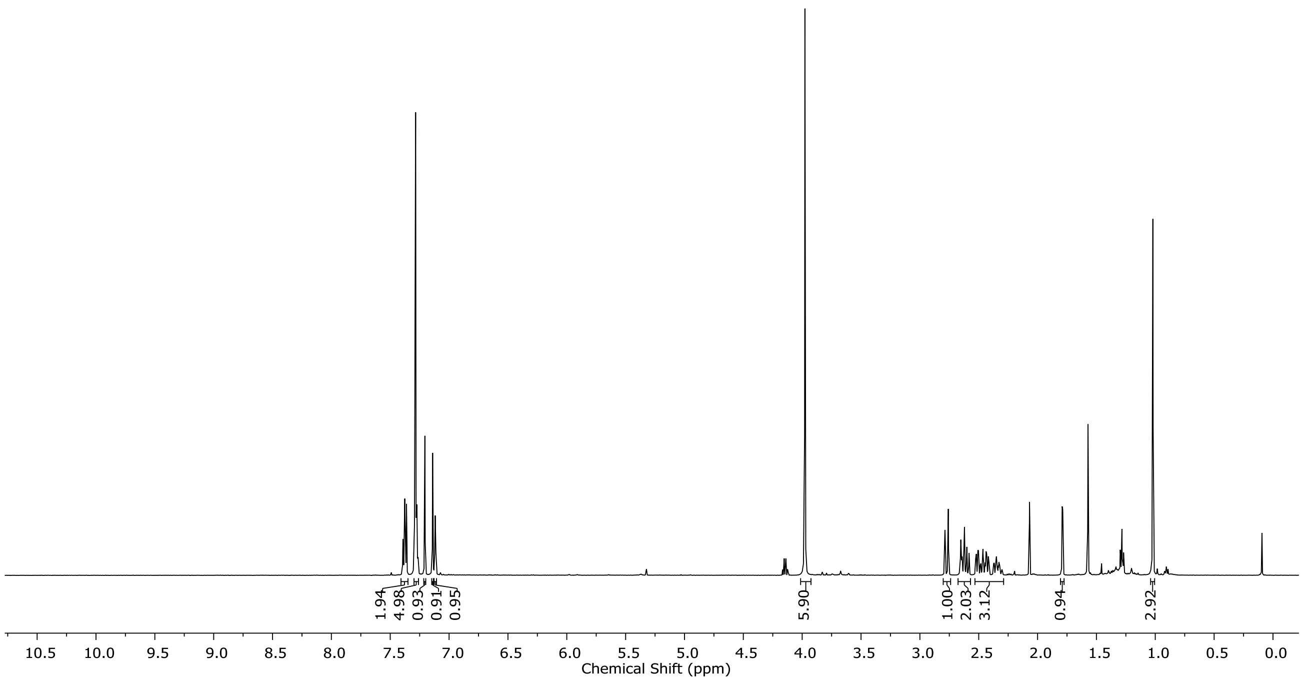
1.94
4.98
0.93
0.91
0.95
5.90
1.00
2.03
3.12
0.94
2.92
10.5
10.0
9.5
9.0
8.5
8.0
7.5
7.0
6.5
6.0
5.5
5.0
4.5
4.0
3.5
3.0
2.5
2.0
1.5
1.0
0.5
0.0
Chemical Shift (ppm)

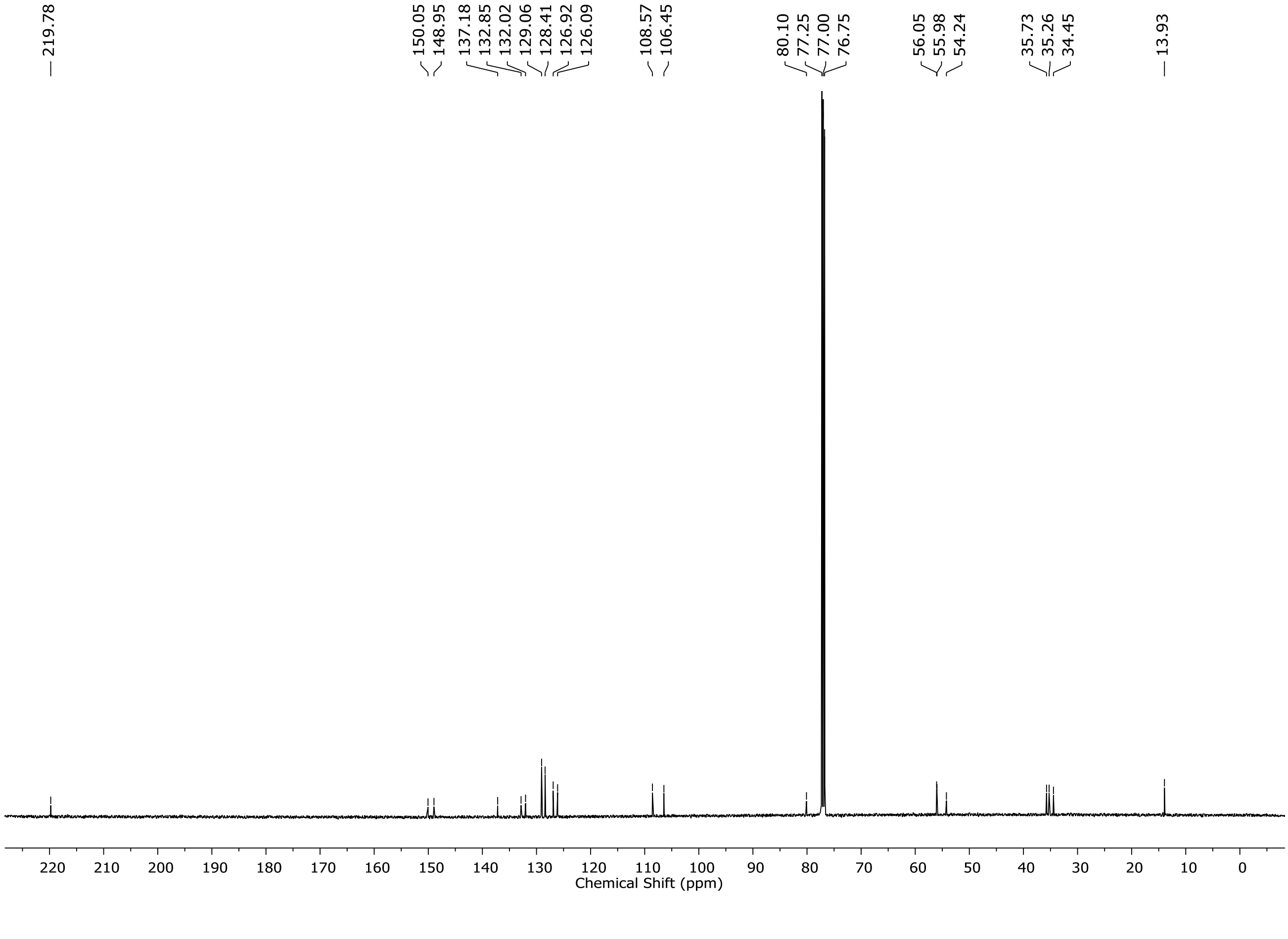
219.78
150.05
148.95
137.18
132.85
132.02
129.06
128.41
126.92
126.09
108.57
106.45
80.10
77.25
77.00
76.75
56.05
55.98
54.24
35.73
35.26
34.45
13.93
220
210
200
190
180
170
160
150
140
130
120
110
100
90
80
70
60
50
40
30
20
10
0
Chemical Shift (ppm)

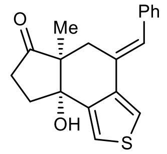
O
Me
Ph
OH
S

5ra

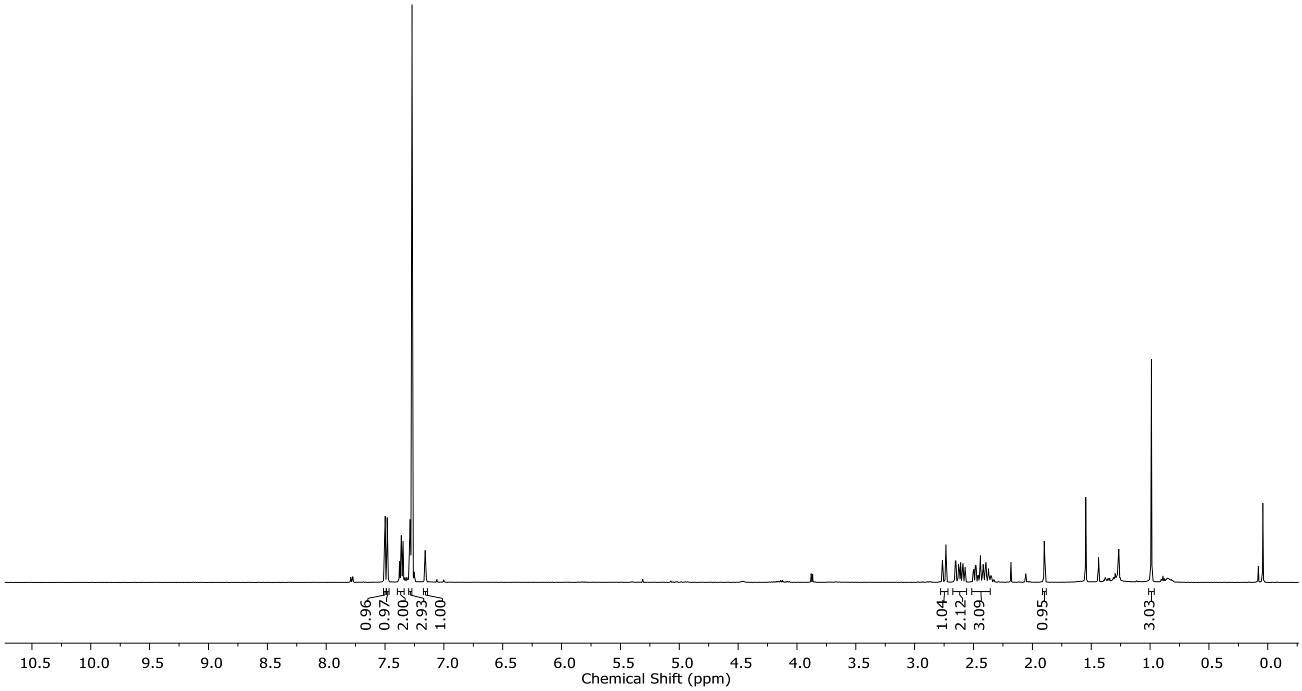
0.96
0.97
2.00
2.93
1.00
1.04
2.12
3.09
0.95
3.03
10.5 10.0 9.5 9.0 8.5 8.0 7.5 7.0 6.5 6.0 5.5 5.0 4.5 4.0 3.5 3.0 2.5 2.0 1.5 1.0 0.5 0.0
Chemical Shift (ppm)

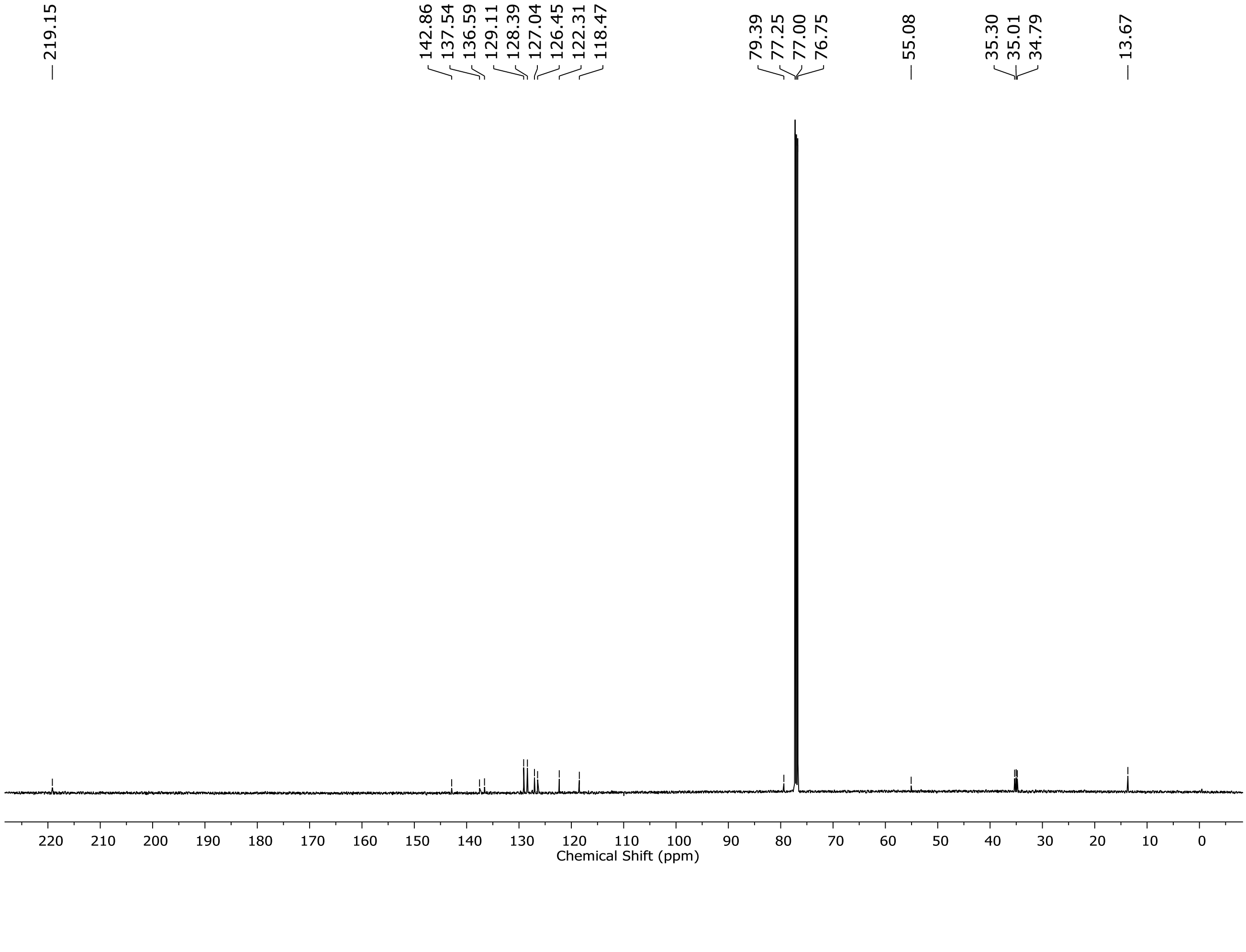
219.15
142.86
137.54
136.59
129.11
128.39
127.04
126.45
122.31
118.47
79.39
77.25
77.00
76.75
55.08
35.30
35.01
34.79
13.67
220 210 200 190 180 170 160 150 140 130 120 110 100 90 80 70 60 50 40 30 20 10 0
Chemical Shift (ppm)

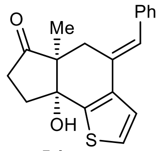
O
Me
Ph
OH
S

5rb

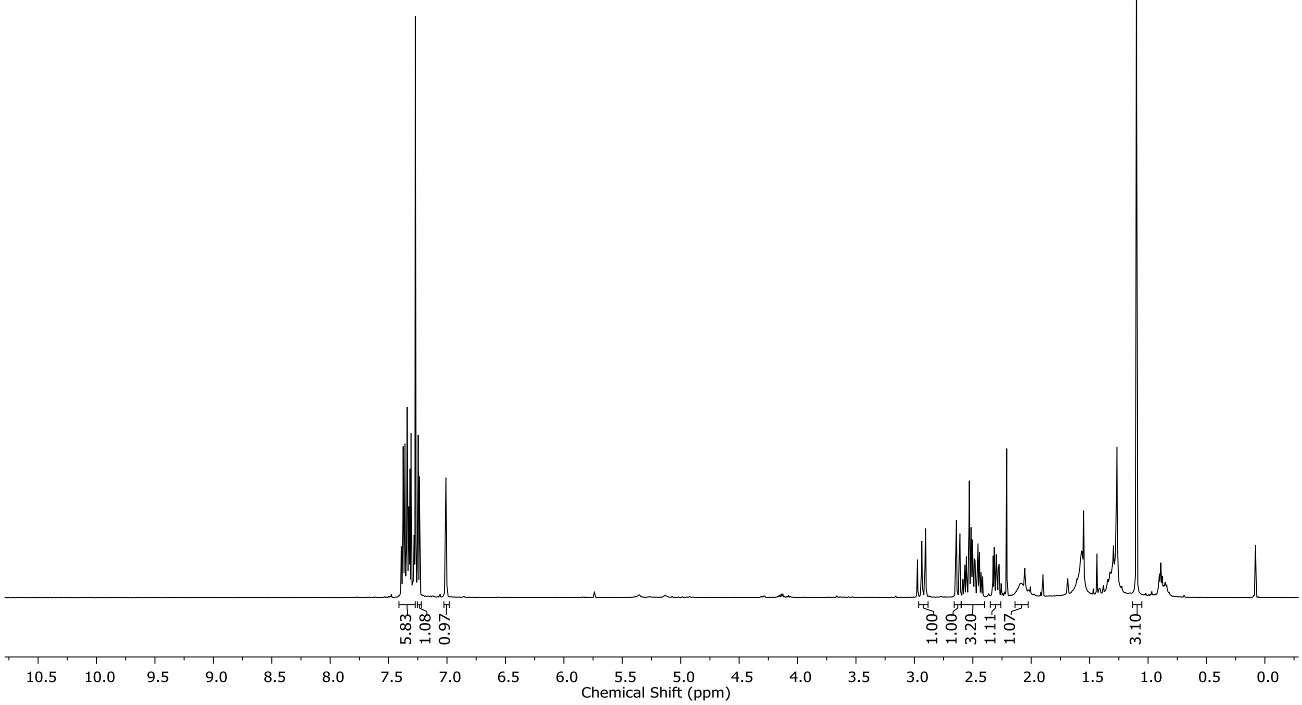
5.83
1.08
0.97
1.00
1.00
3.20
1.11
1.07
3.10
10.5 10.0 9.5 9.0 8.5 8.0 7.5 7.0 6.5 6.0 5.5 5.0 4.5 4.0 3.5 3.0 2.5 2.0 1.5 1.0 0.5 0.0
Chemical Shift (ppm)

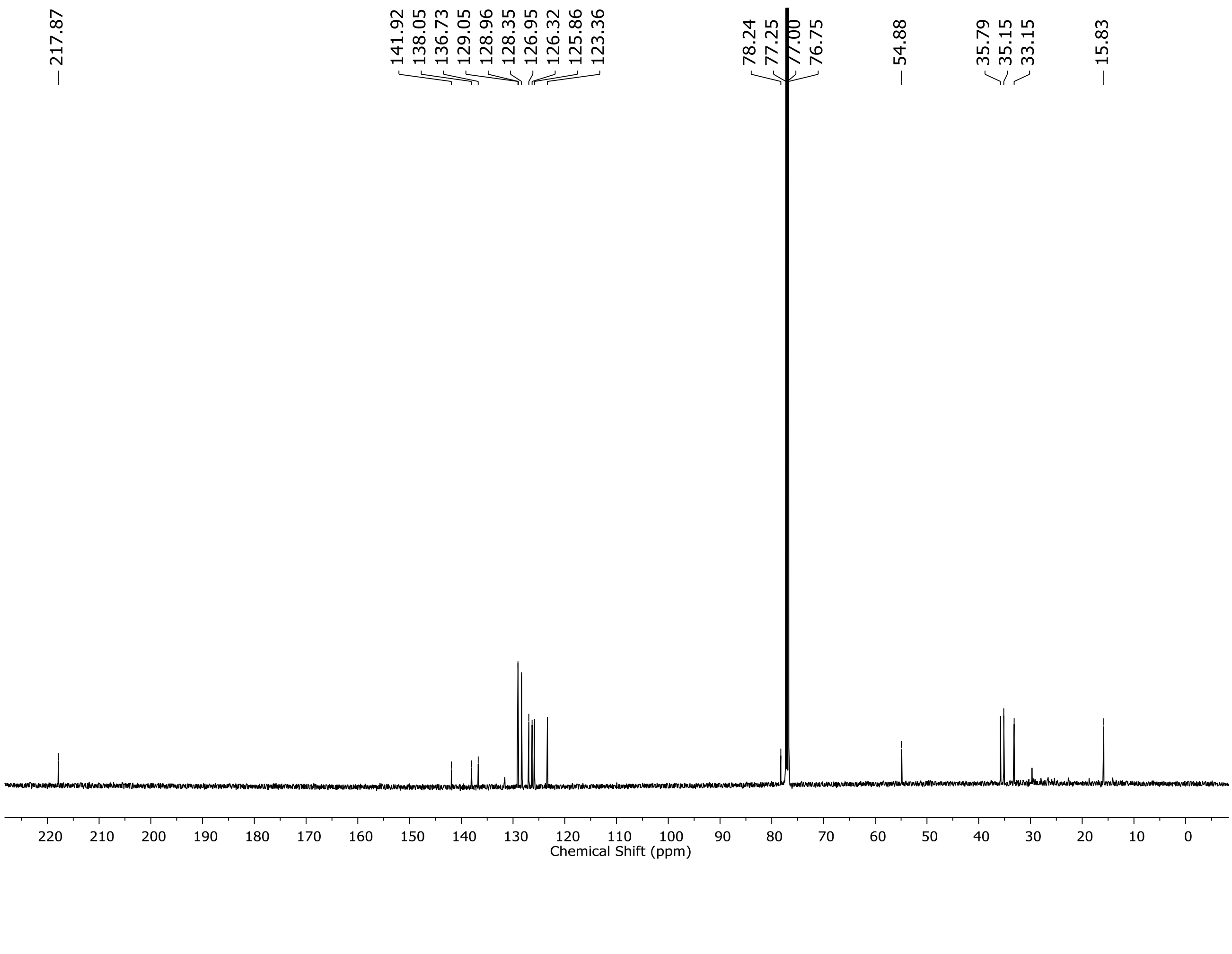
217.87
141.92
138.05
136.73
129.05
128.96
128.35
126.95
126.32
125.86
123.36
78.24
77.25
77.00
76.75
54.88
35.79
35.15
33.15
15.83
220 210 200 190 180 170 160 150 140 130 120 110 100 90 80 70 60 50 40 30 20 10 0
Chemical Shift (ppm)

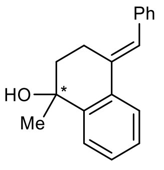
Ph
HO
*
Me

7

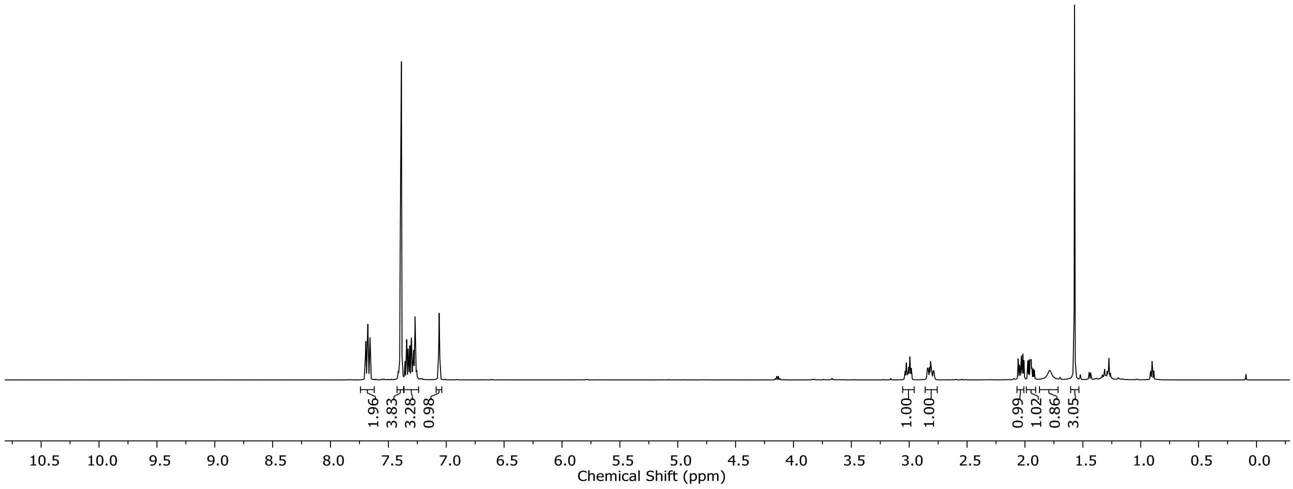
1.96
3.83
3.28
0.98
1.00
1.00
0.99
1.02
0.86
3.05
10.5 10.0 9.5 9.0 8.5 8.0 7.5 7.0 6.5 6.0 5.5 5.0 4.5 4.0 3.5 3.0 2.5 2.0 1.5 1.0 0.5 0.0
Chemical Shift (ppm)

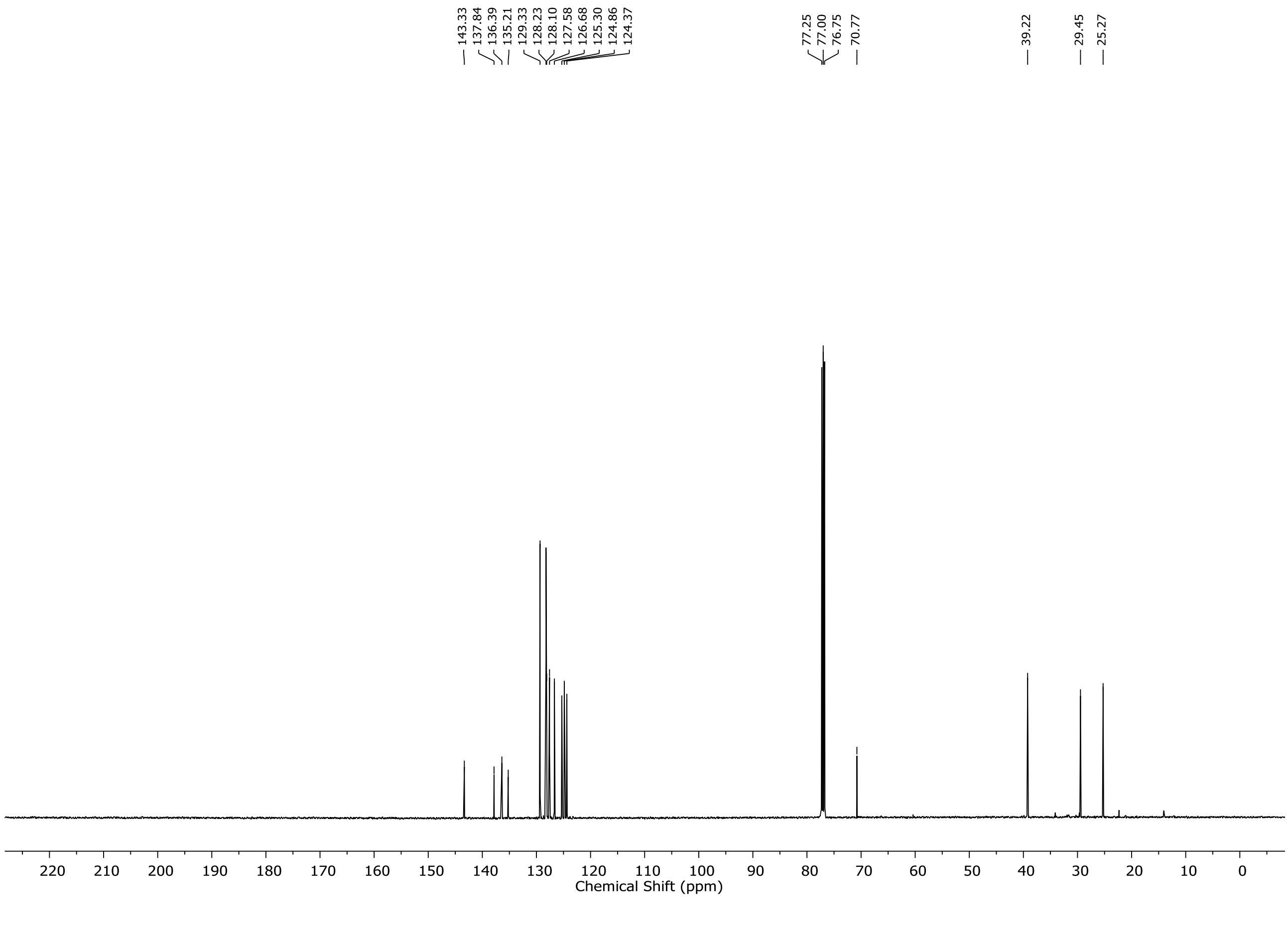
143.33
137.84
136.39
135.21
129.33
128.23
128.10
127.58
126.68
125.30
124.86
124.37
77.25
77.00
76.75
70.77
39.22
29.45
25.27
220 210 200 190 180 170 160 150 140 130 120 110 100 90 80 70 60 50 40 30 20 10 0
Chemical Shift (ppm)

HPLC Traces

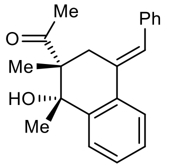

2a

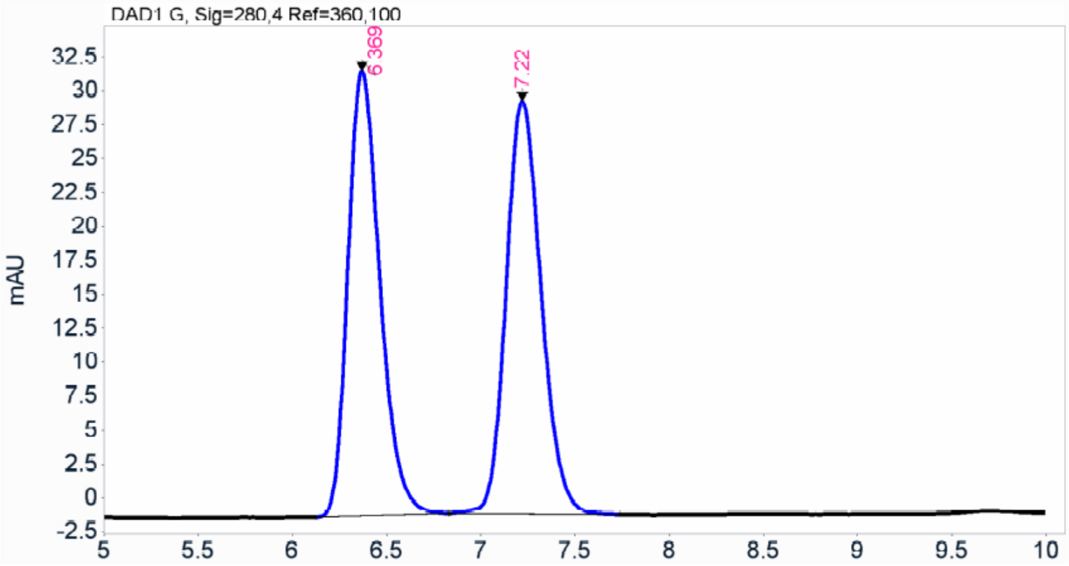

Signal:	DAD1 G, Sig=280,4 Ref=360,100				
RT [min]	Type	Width [min]	Area	Height	Area%
6.369	BB	0.1813	385.180	32.7262	49.52
7.220	BB	0.1987	392.669	30.3892	50.48

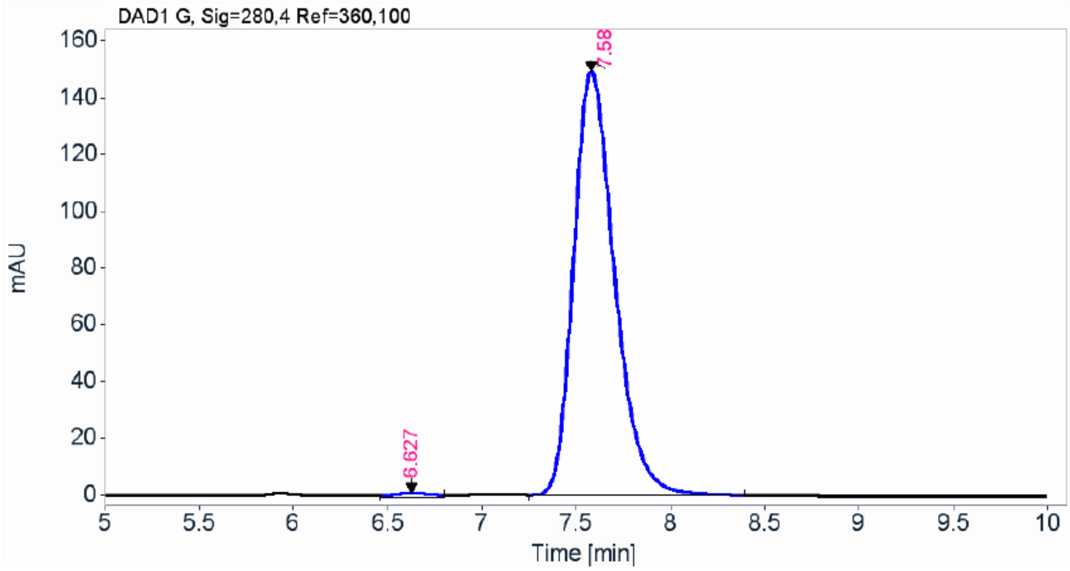

Signal:	DAD1 G, Sig=280,4 Ref=360,100				
RT [min]	Type	Width [min]	Area	Height	Area%
6.627	MM	0.2520	20.633	1.3645	0.91
7.580	BB	0.2317	2248.980	149.3599	99.09

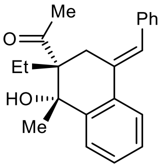

2b

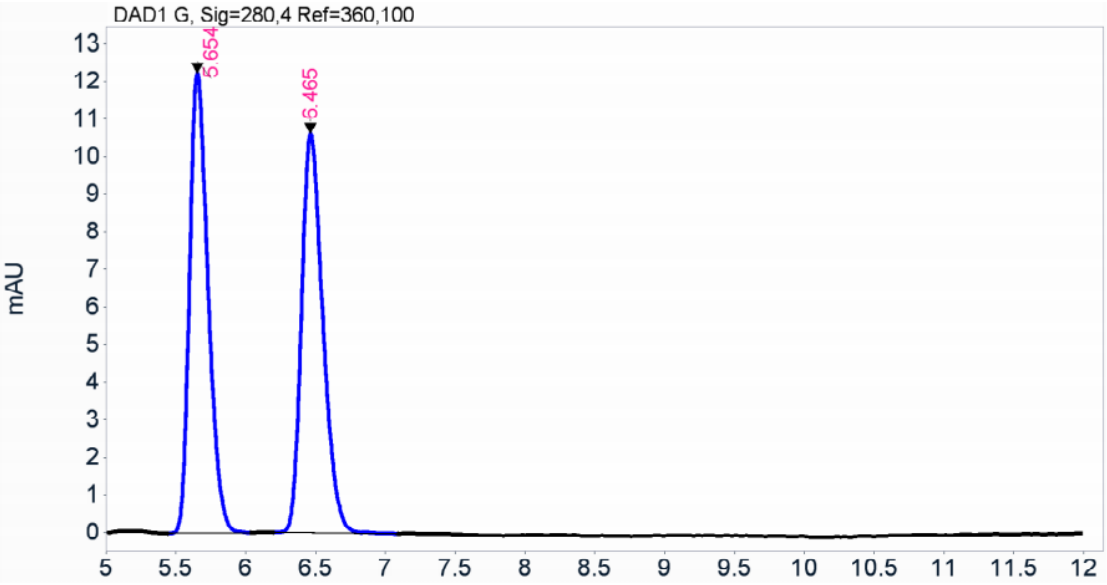

Signal: DAD1 G, Sig=280,4 Ref=360,100

RT [min]	Type	Width [min]	Area	Height	Area%
5.654	BB	0.1395	112.808	12.2448	49.98
6.465	BB	0.1620	112.883	10.6227	50.02

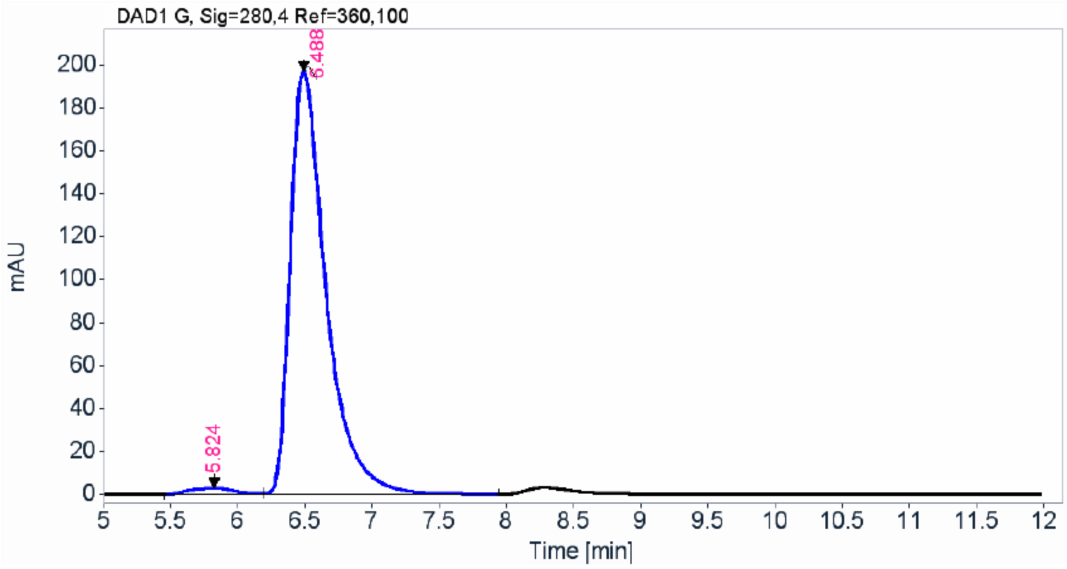

Signal: DAD1 G, Sig=280,4 Ref=360,100

RT [min]	Type	Width [min]	Area	Height	Area%
5.824	BV	0.3101	62.928	2.9120	1.70
6.488	VB	0.2772	3643.215	197.4686	98.30

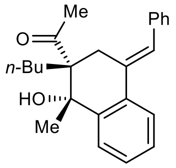

2c

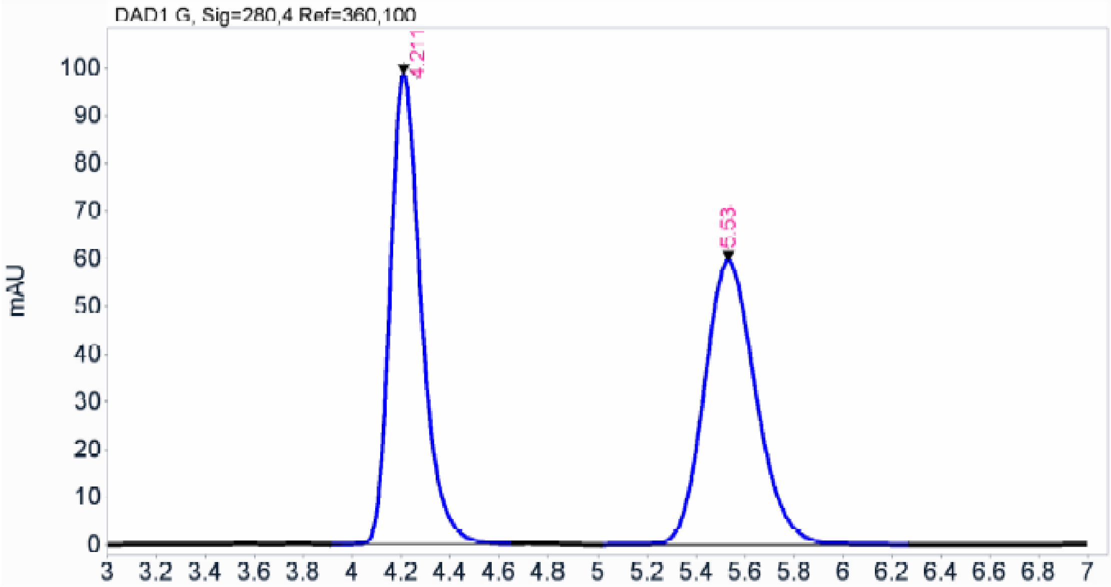

Signal:	DAD1 G, Sig=280,4 Ref=360,100				
RT [min]	Type	Width [min]	Area	Height	Area%
4.211	BB	0.1346	866.564	98.5235	49.89
5.530	BB	0.2268	870.260	59.4397	50.11

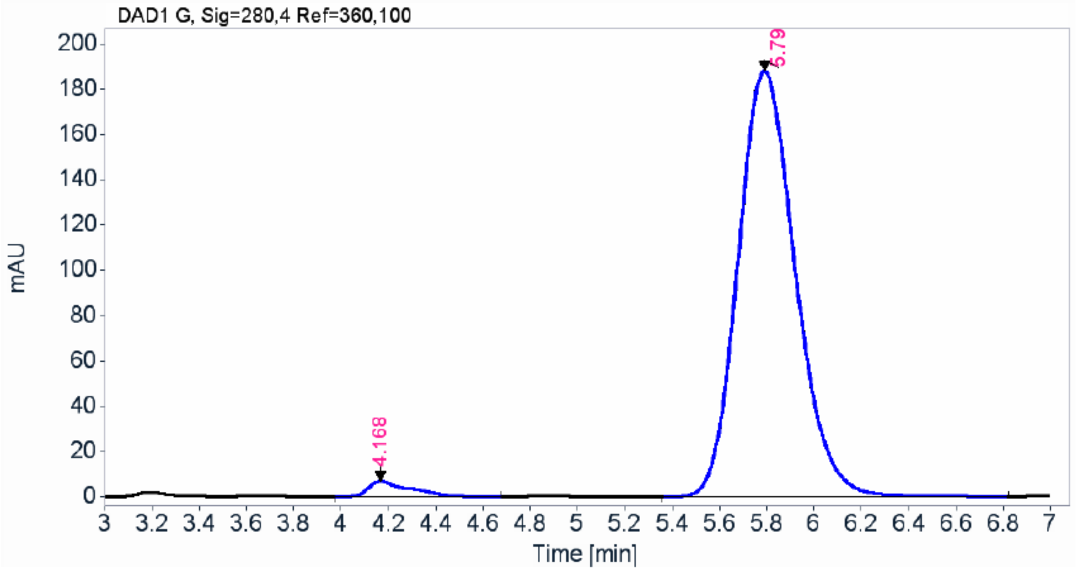

Signal:	DAD1 G, Sig=280,4 Ref=360,100				
RT [min]	Type	Width [min]	Area	Height	Area%
4.168	BB	0.1702	82.161	6.7397	2.54
5.790	BB	0.2599	3158.171	188.1224	97.46

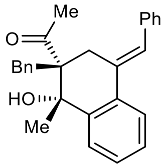

2d

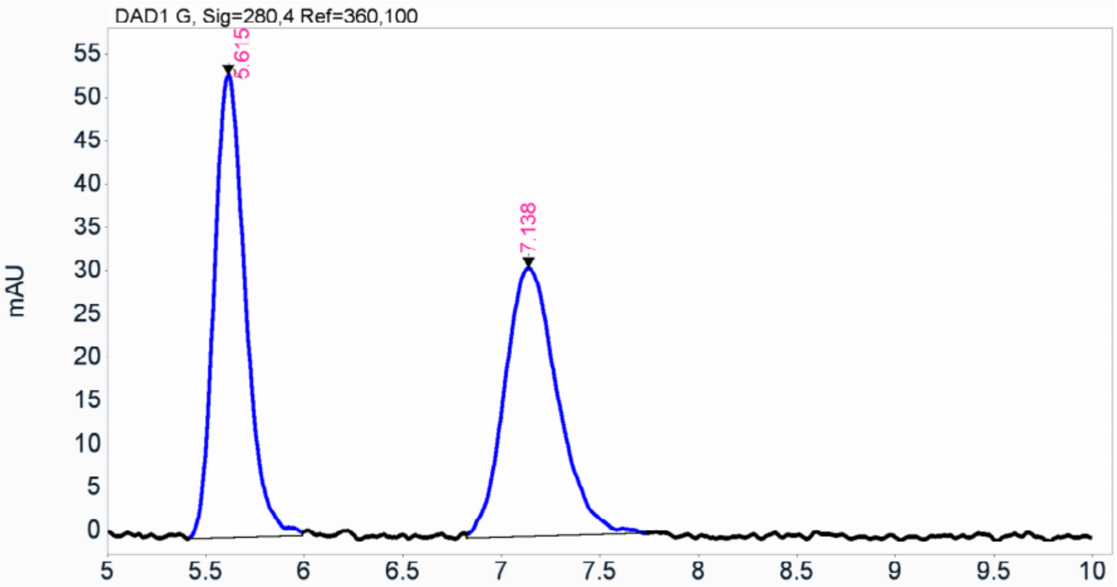

Signal:	DAD1 G, Sig=280,4 Ref=360,100				
RT [min]	Type	Width [min]	Area	Height	Area%
5.615	BV	0.1679	584.283	53.2843	50.33
7.138	VB	0.2893	576.689	30.9583	49.67

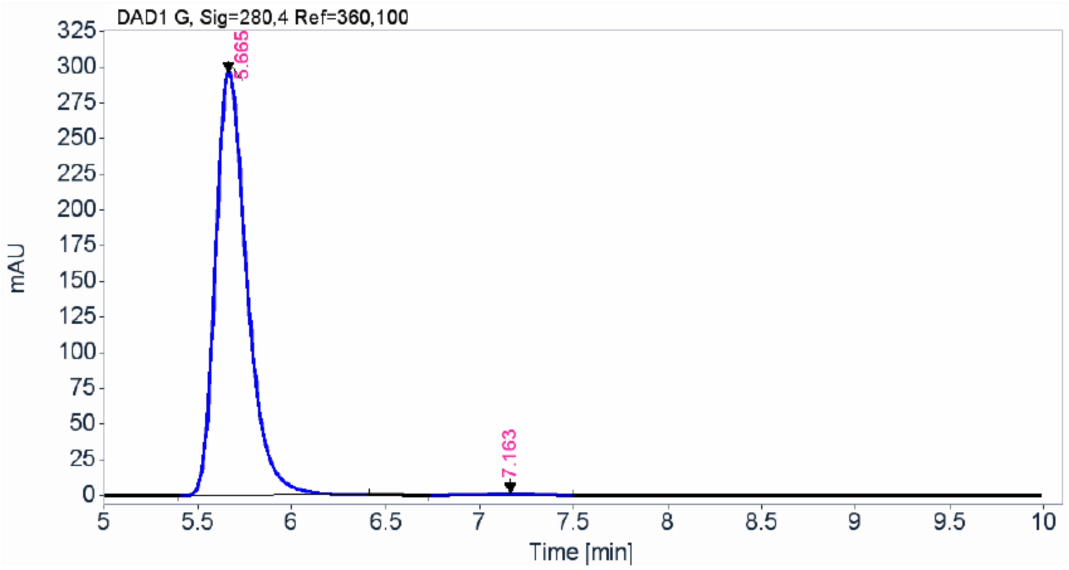

Signal:	DAD1 G, Sig=280,4 Ref=360,100				
RT [min]	Type	Width [min]	Area	Height	Area%
5.665	BB	0.1780	3460.021	296.8958	99.21
7.163	BB	0.2614	27.504	1.4761	0.79

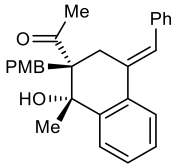

2e

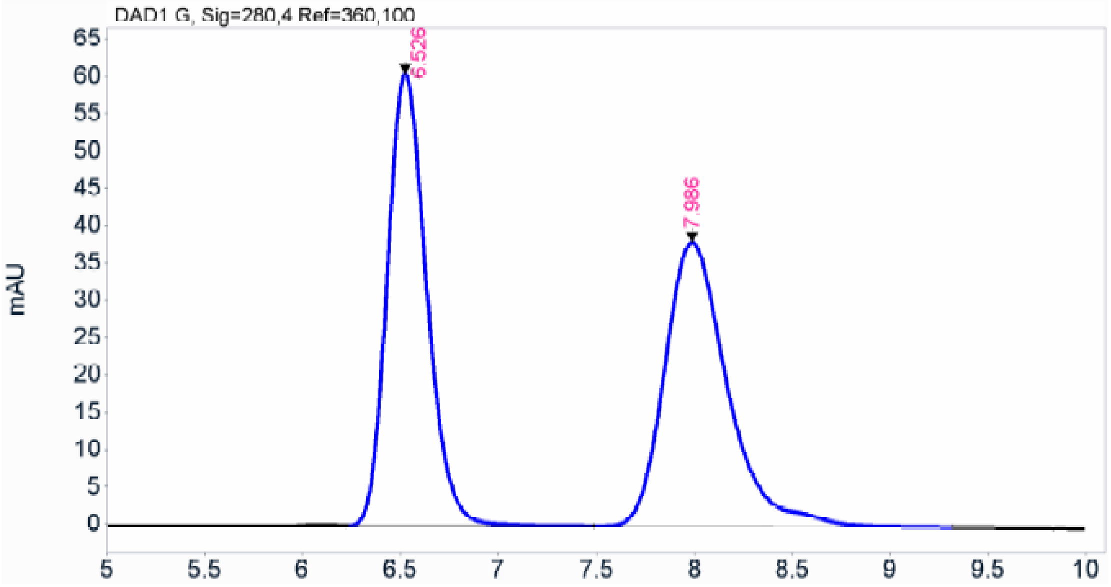

Signal:	DAD1 G, Sig=280,4 Ref=360,100				
RT [min]	Type	Width [min]	Area	Height	Area%
6.526	BB	0.2176	849.526	60.5746	49.90
7.986	BB	0.3413	853.081	38.0871	50.10

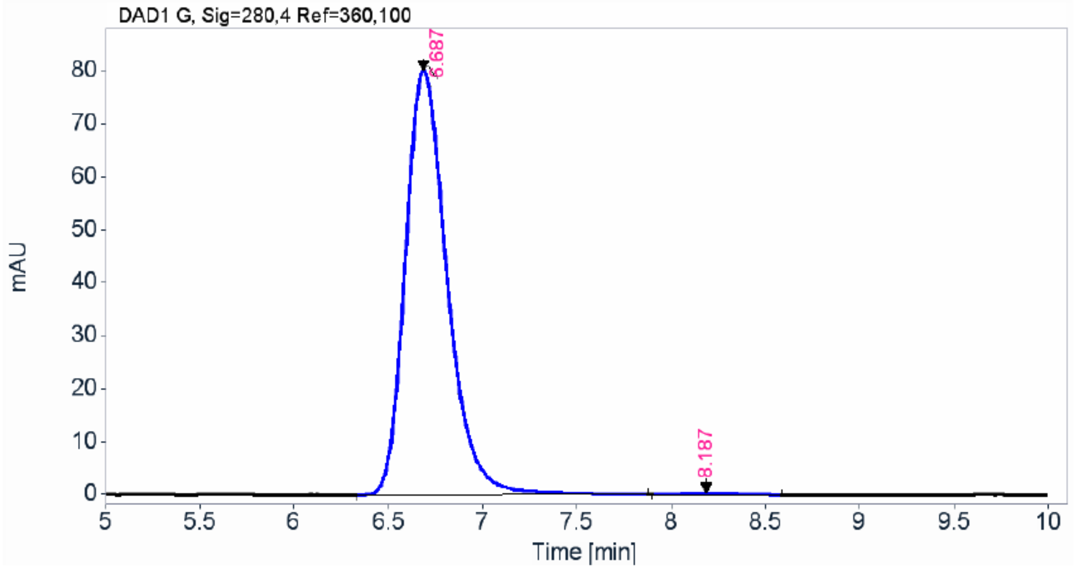

Signal:	DAD1 G, Sig=280,4 Ref=360,100				
RT [min]	Type	Width [min]	Area	Height	Area%
6.687	BB	0.2360	1236.546	80.1485	99.03
8.187	MM	0.4074	12.110	0.4955	0.97

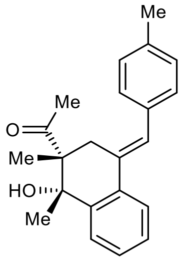

2f

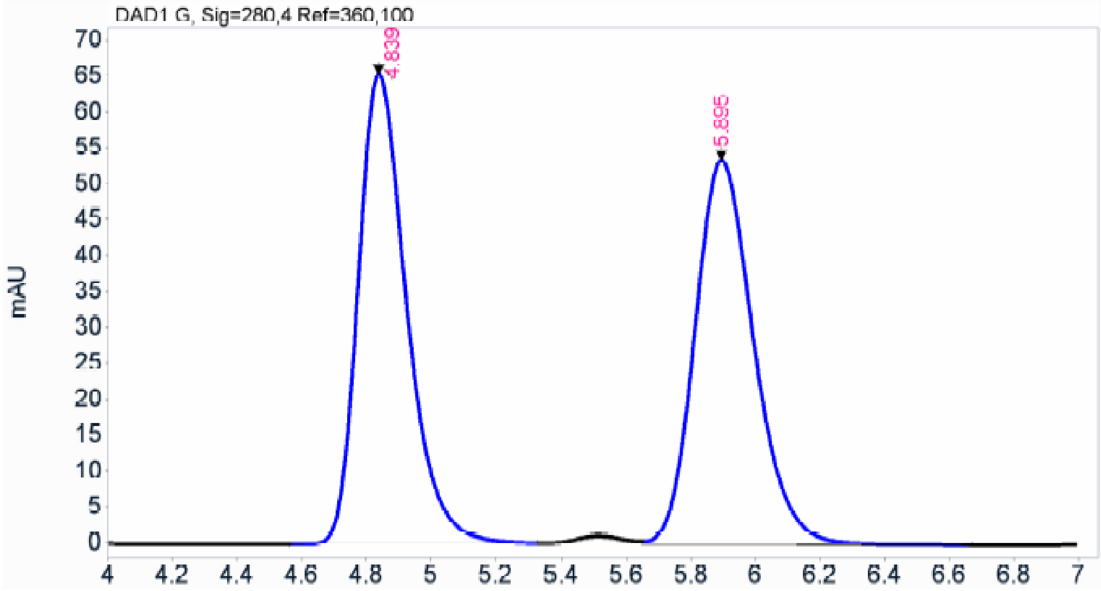

Signal:	DAD1 G, Sig=280,4 Ref=360,100				
RT [min]	Type	Width [min]	Area	Height	Area%
4.839	BB	0.1567	677.047	65.4499	50.08
5.895	VB	0.1953	674.862	53.4393	49.92

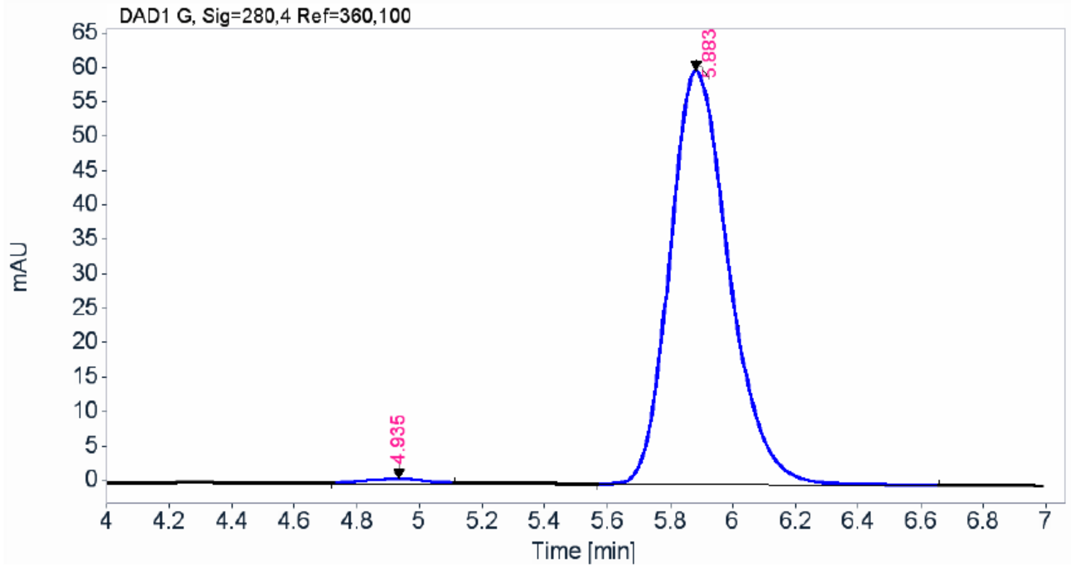

Signal:	DAD1 G, Sig=280,4 Ref=360,100				
RT [min]	Type	Width [min]	Area	Height	Area%
4.935	MM	0.2378	11.071	0.7758	1.39
5.883	BB	0.2008	786.953	60.0695	98.61

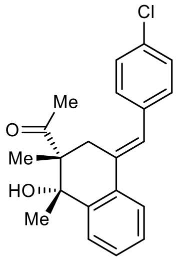

2g

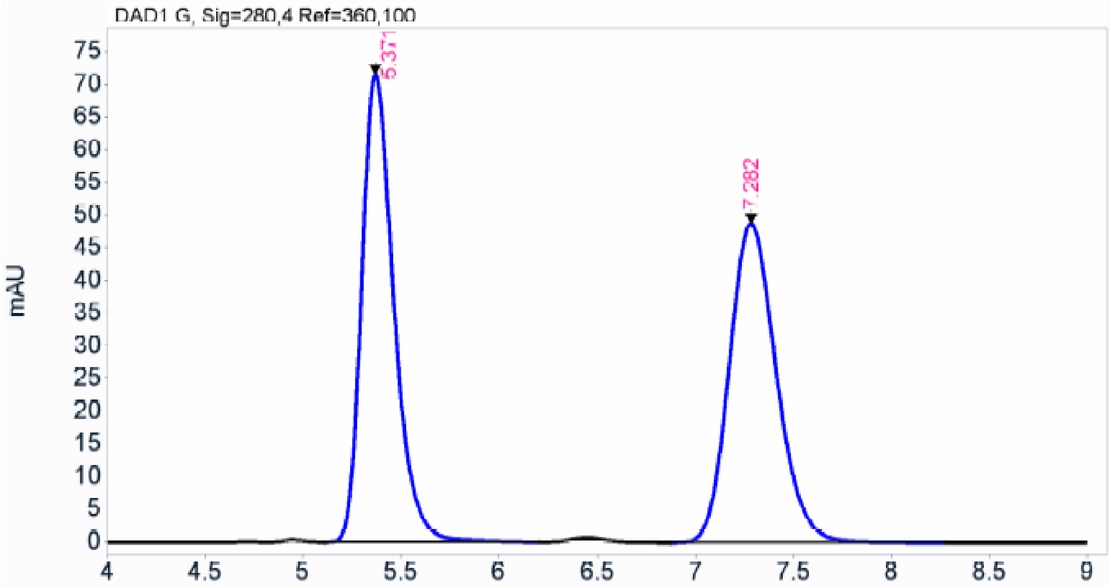

Signal:	DAD1 G, Sig=280,4 Ref=360,100				
RT [min]	Type	Width [min]	Area	Height	Area%
5.371	BB	0.1750	816.194	71.5697	50.00
7.282	BB	0.2589	816.158	48.8433	50.00

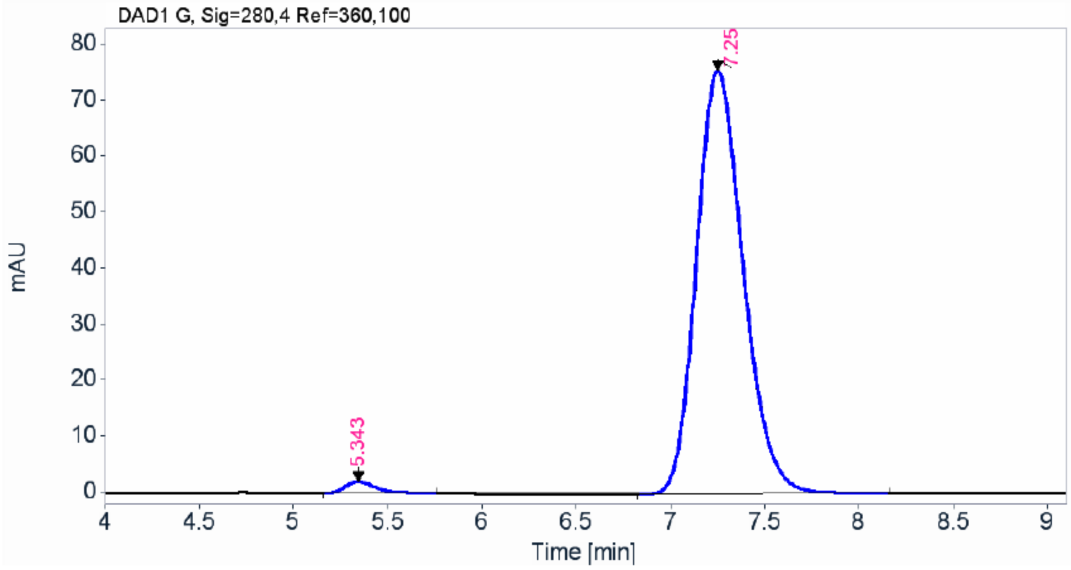

Signal:	DAD1 G, Sig=280,4 Ref=360,100				
RT [min]	Type	Width [min]	Area	Height	Area%
5.343	BB	0.1722	23.151	2.0430	1.75
7.250	BB	0.2668	1301.336	75.6090	98.25

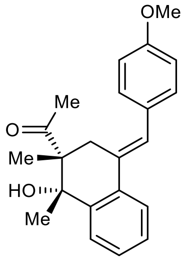

2h

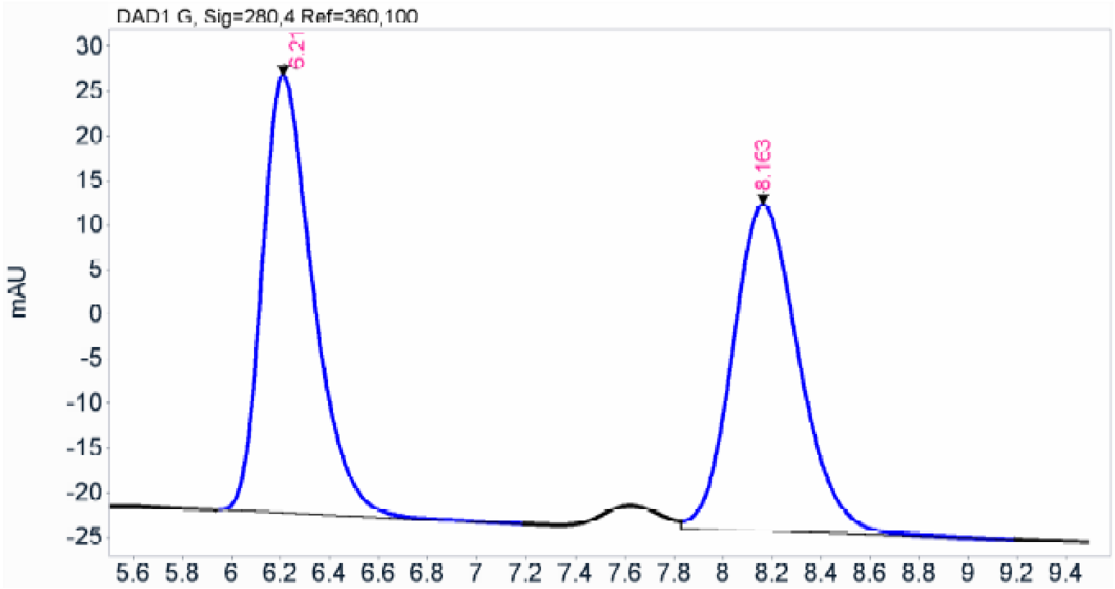

Signal:	DAD1 G, Sig=280,4 Ref=360,100				
**RT [min]**	**Type**	**Width [min]**	**Area**	**Height**	**Area%**
6.210	BB	0.2199	705.808	49.0429	50.54
8.163	VB	0.2919	690.835	36.6498	49.46

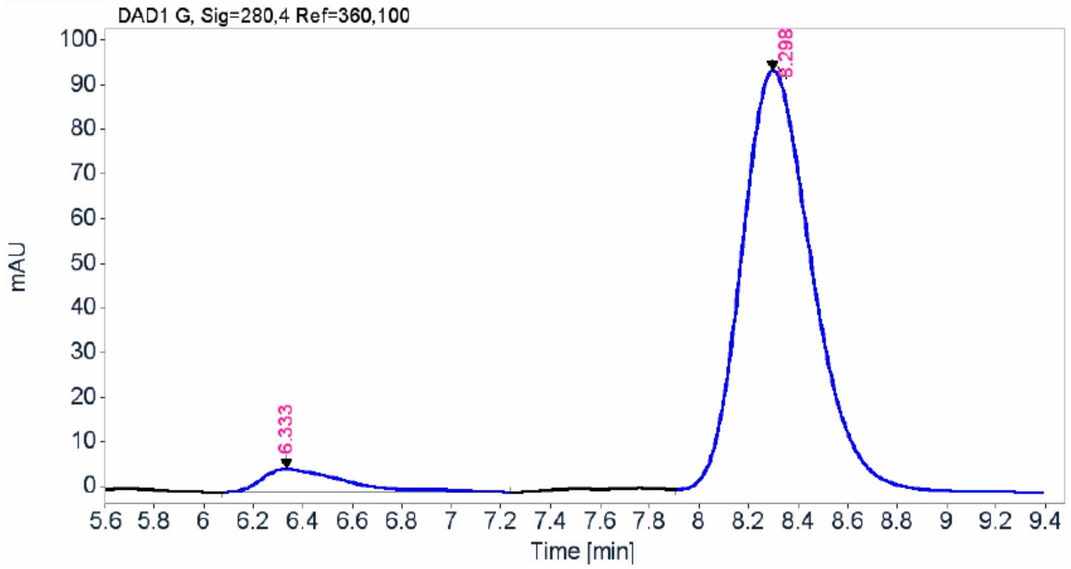

Signal:	DAD1 G, Sig=280,4 Ref=360,100				
**RT [min]**	**Type**	**Width [min]**	**Area**	**Height**	**Area%**
6.333	BB	0.3215	119.517	5.1742	6.07
8.298	BB	0.3056	1848.811	93.9347	93.93

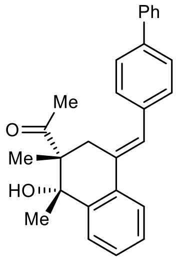

2i

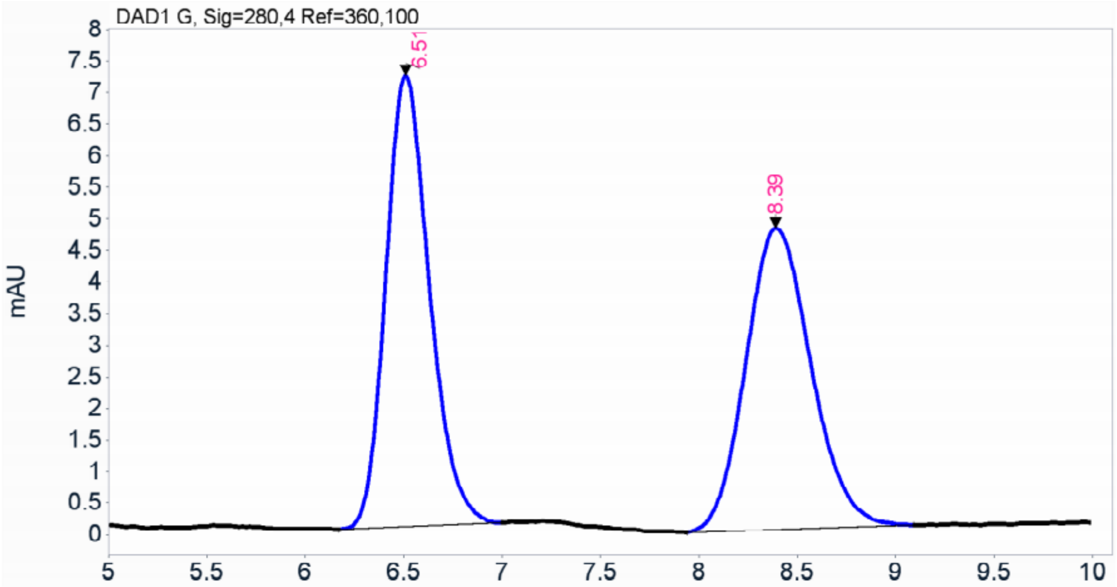

Signal: DAD1 G, Sig=280,4 Ref=360,100

RT [min]	Type	Width [min]	Area	Height	Area%
6.510	BB	0.2379	110.278	7.1515	50.13
8.390	BB	0.3503	109.707	4.7710	49.87

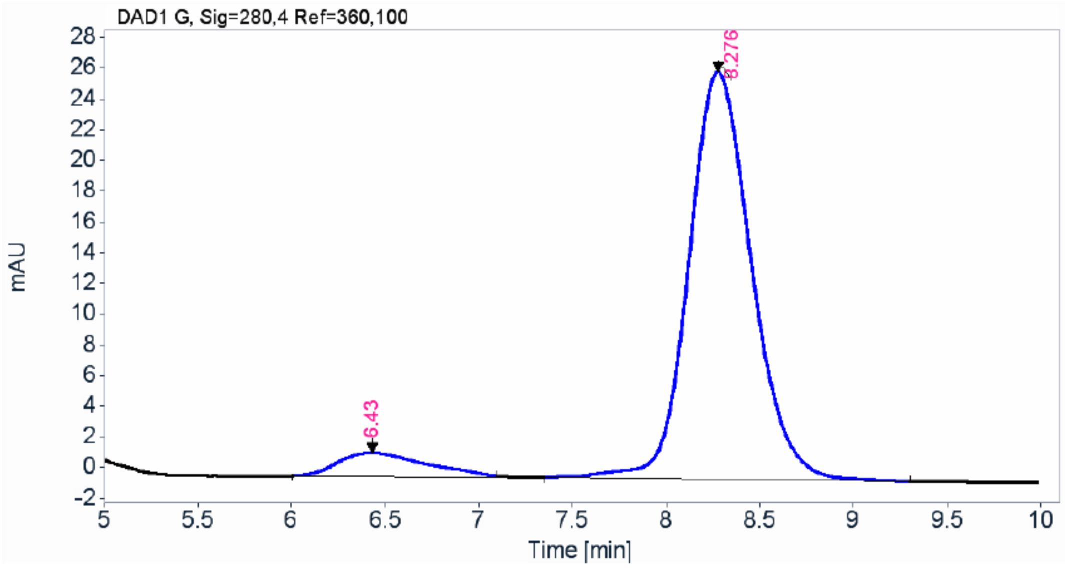

Signal: DAD1 G, Sig=280,4 Ref=360,100

RT [min]	Type	Width [min]	Area	Height	Area%
6.430	MM	0.5427	50.668	1.5562	7.62
8.276	BB	0.3565	614.304	26.4922	92.38

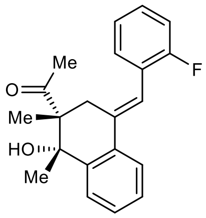

2j

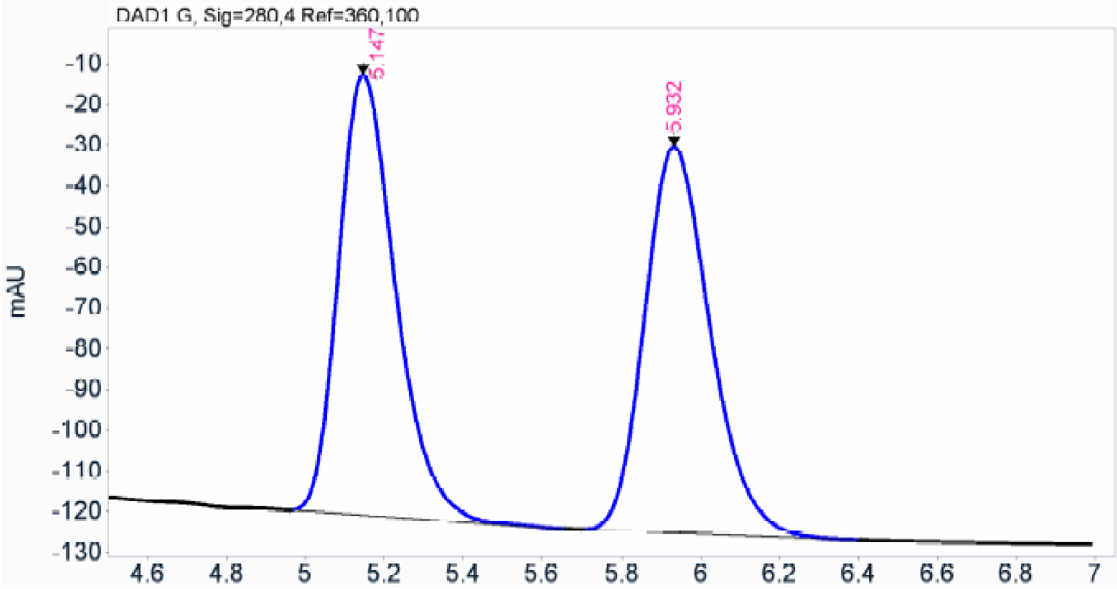

Signal:	DAD1 G, Sig=280,4 Ref=360,100				
RT [min]	Type	Width [min]	Area	Height	Area%
5.147	BB	0.1553	1088.631	108.2370	49.76
5.932	BB	0.1791	1099.151	94.9196	50.24

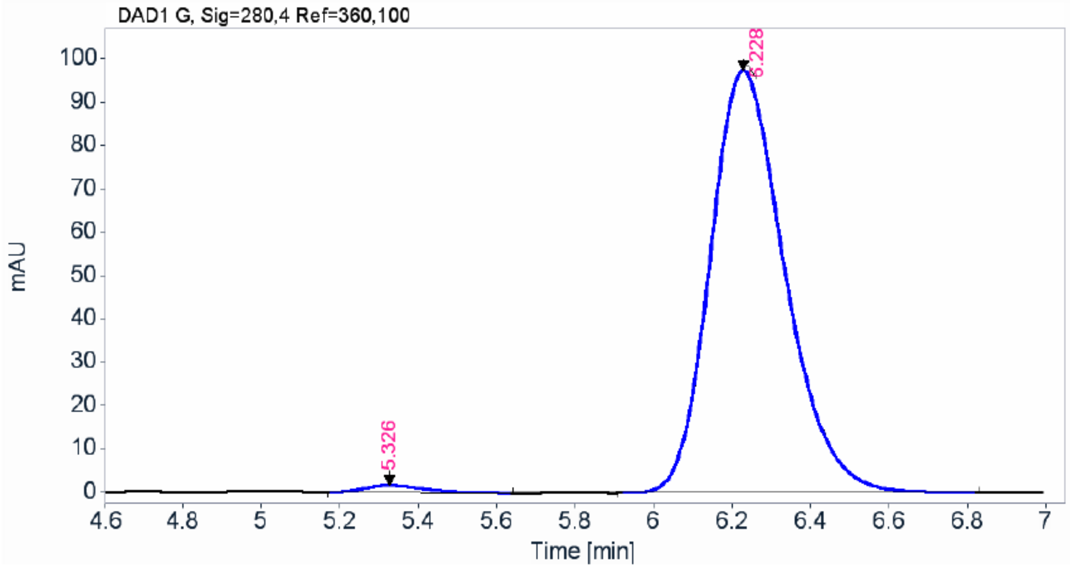

Signal:	DAD1 G, Sig=280,4 Ref=360,100				
RT [min]	Type	Width [min]	Area	Height	Area%
5.326	BB	0.1616	17.168	1.6468	1.32
6.228	BB	0.2040	1286.342	97.4244	98.68

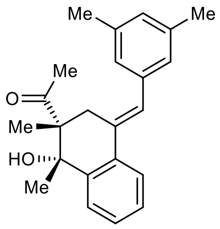

2k

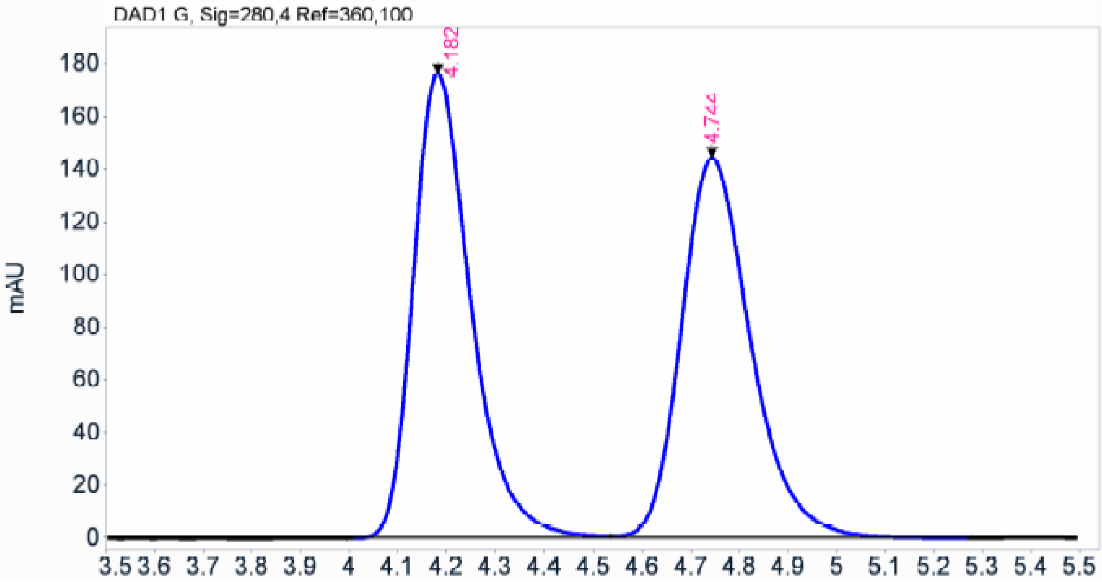

Signal:	DAD1 G, Sig=280,4 Ref=360,100				
RT [min]	Type	Width [min]	Area	Height	Area%
4.182	BV	0.1260	1452.526	176.5803	50.33
4.744	VB	0.1539	1433.747	144.3077	49.67

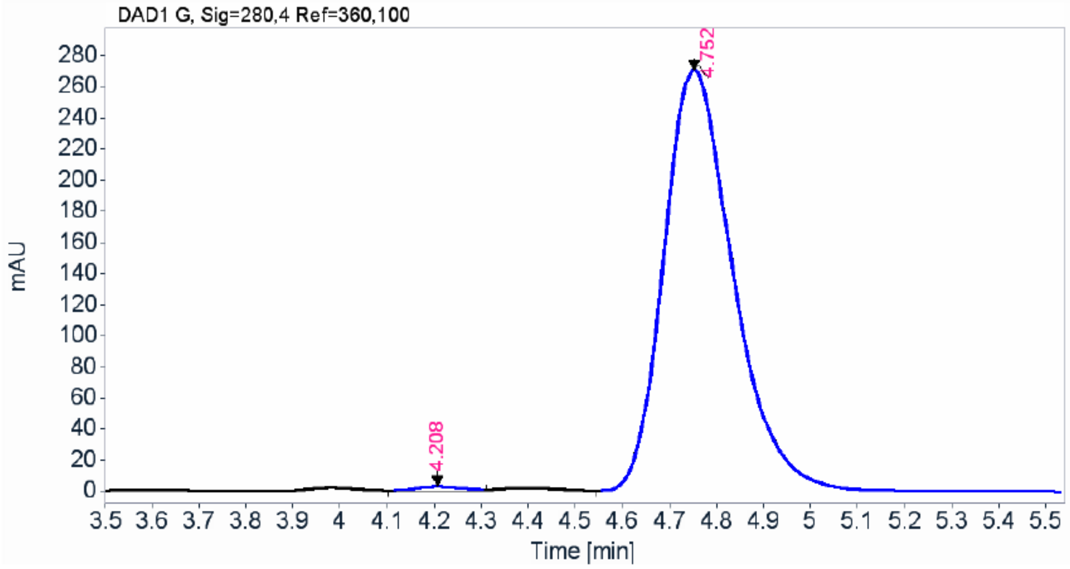

Signal:	DAD1 G, Sig=280,4 Ref=360,100				
RT [min]	Type	Width [min]	Area	Height	Area%
4.208	BV	0.1101	19.138	2.7877	0.68
4.752	VB	0.1577	2779.416	270.8972	99.32

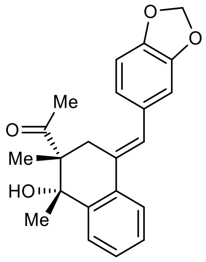

2l

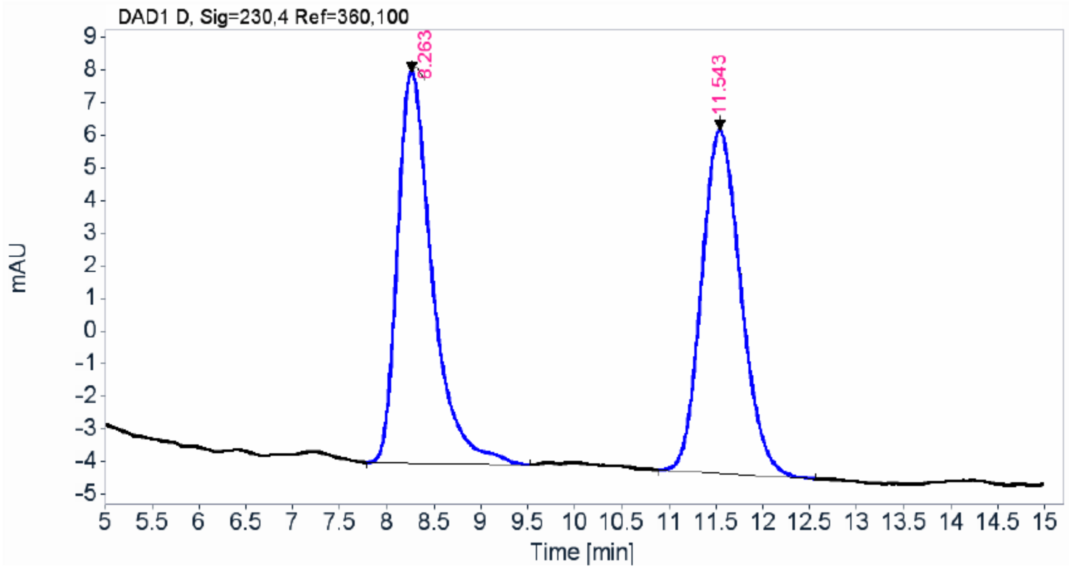

Signal: DAD1 D, Sig=230,4 Ref=360,100

RT [min]	Type	Width [min]	Area	Height	Area%
8.263	BB	0.3877	307.980	11.9899	50.16
11.543	BB	0.4536	306.069	10.5139	49.84

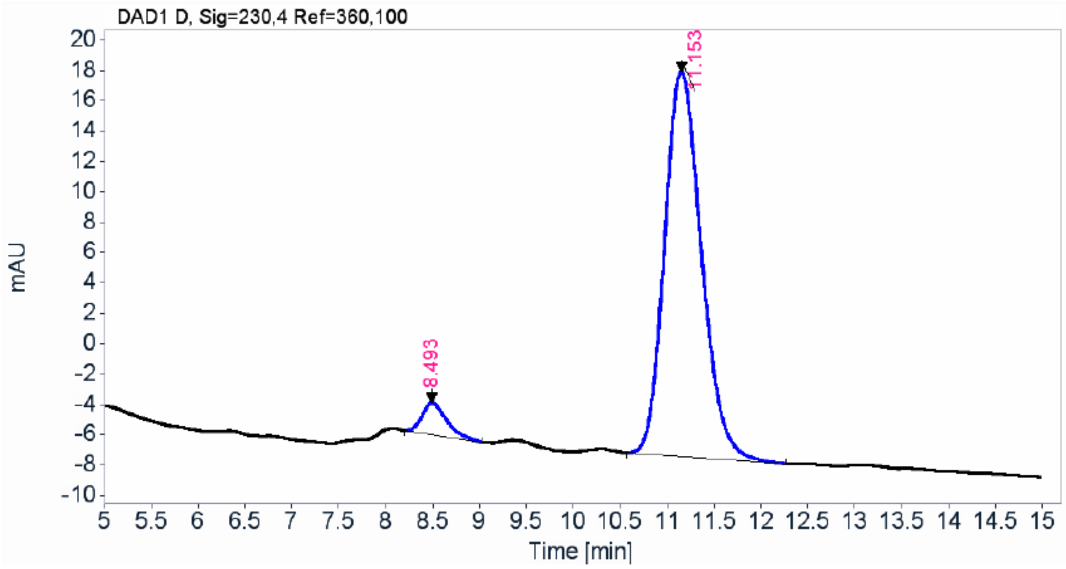

Signal: DAD1 D, Sig=230,4 Ref=360,100

RT [min]	Type	Width [min]	Area	Height	Area%
8.493	BB	0.2625	38.115	2.0728	5.22
11.153	BB	0.4205	691.966	25.3479	94.78

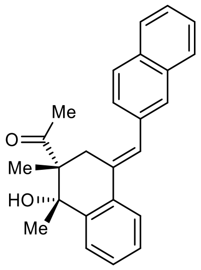

2m

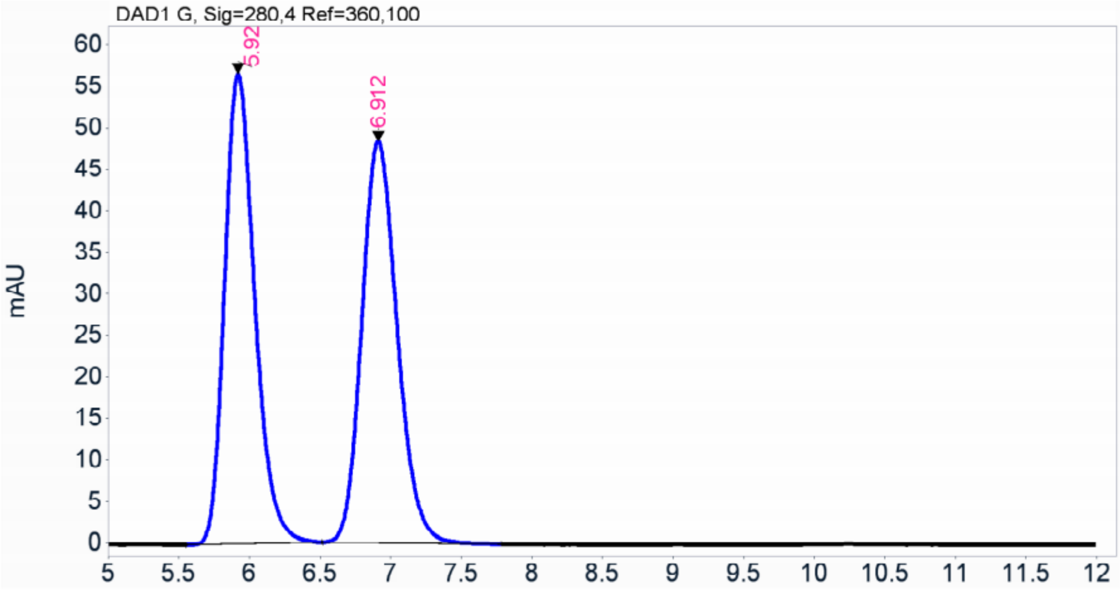

Signal:	DAD1 G, Sig=280,4 Ref=360,100				
RT [min]	Type	Width [min]	Area	Height	Area%
5.920	BB	0.2286	837.386	56.6094	49.92
6.912	BB	0.2690	840.118	48.2851	50.08

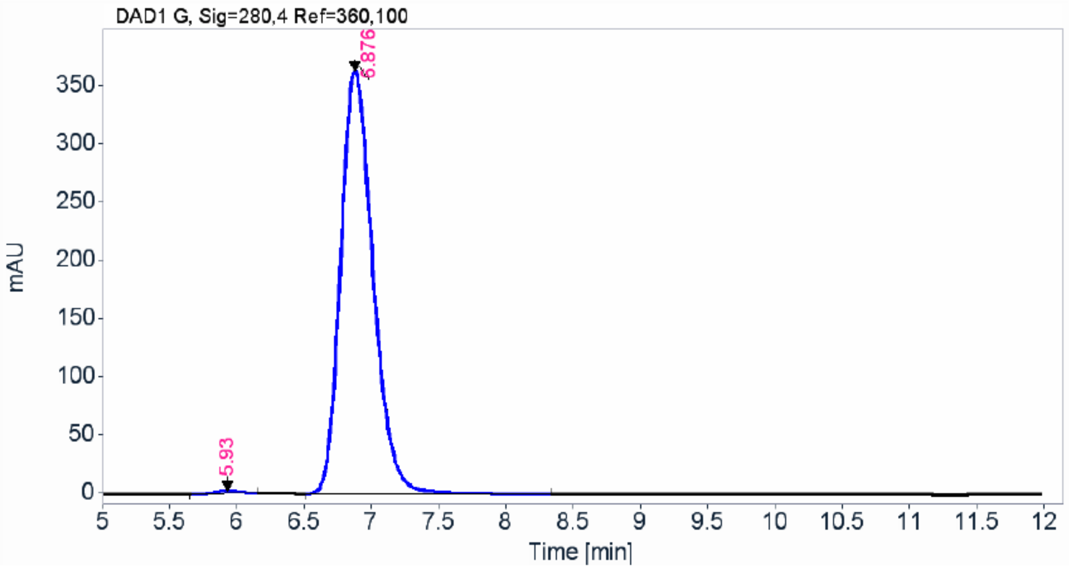

Signal:	DAD1 G, Sig=280,4 Ref=360,100				
RT [min]	Type	Width [min]	Area	Height	Area%
5.930	BB	0.1988	28.923	2.3958	0.47
6.876	BB	0.2593	6074.113	362.8996	99.53

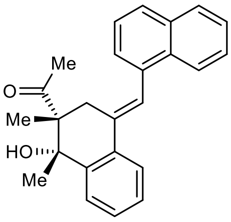

2n

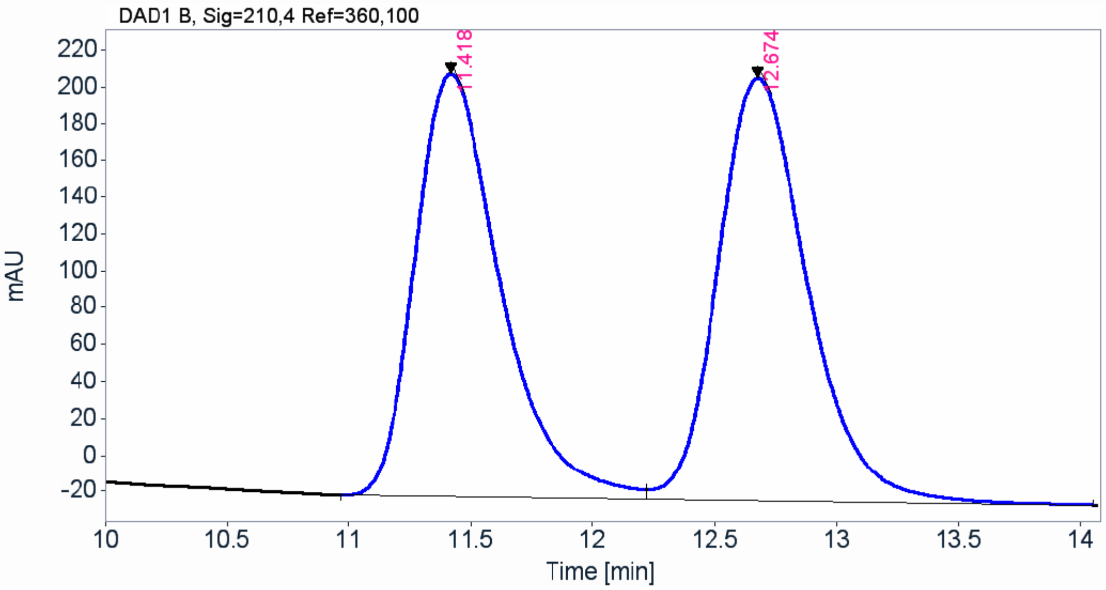

Signal:	DAD1 B, Sig=210,4 Ref=360,100				
RT [min]	Type	Width [min]	Area	Height	Area%
11.418	BV	0.3708	5620.365	230.2351	48.85
12.674	VB	0.3925	5885.084	230.0381	51.15

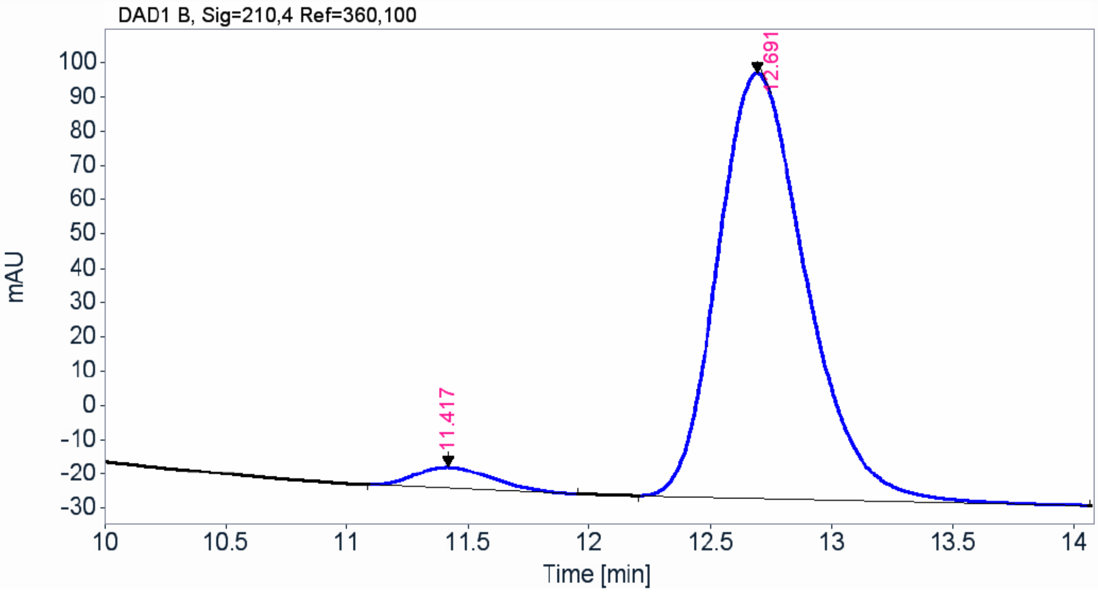

Signal:	DAD1 B, Sig=210,4 Ref=360,100				
RT [min]	Type	Width [min]	Area	Height	Area%
11.417	BB	0.3340	138.194	6.2987	4.21
12.691	BB	0.3914	3142.159	124.1093	95.79

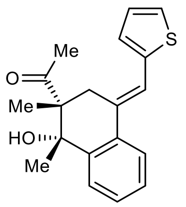

2o

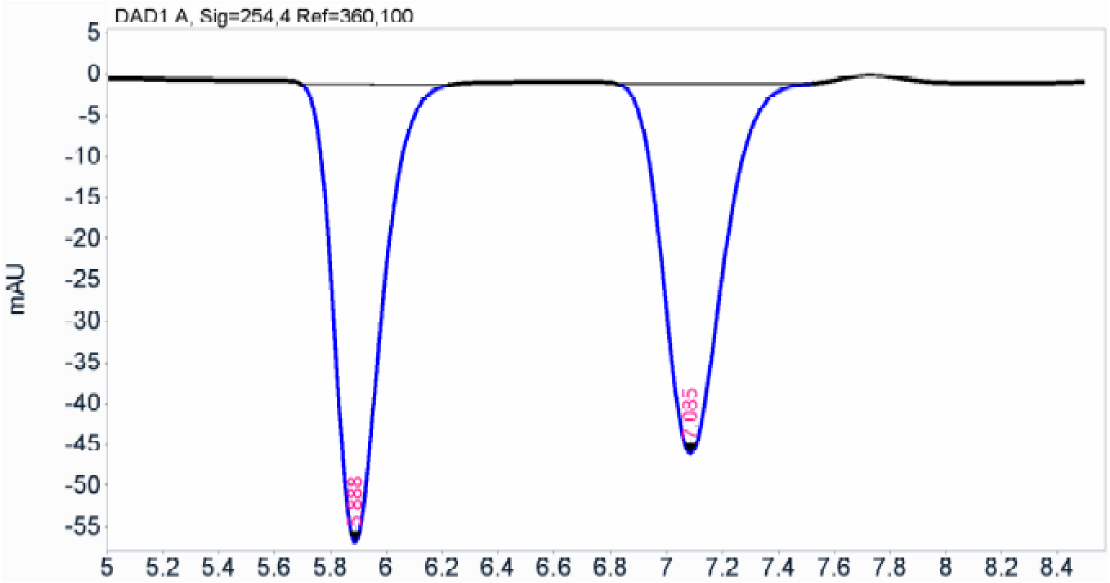

Signal:	DAD1 A, Sig=254,4 Ref=360,100				
RT [min]	Type	Width [min]	Area	Height	Area%
5.888	PP N	0.1877	626.524	55.6463	49.67
7.085	PP N	0.2353	634.799	44.9588	50.33

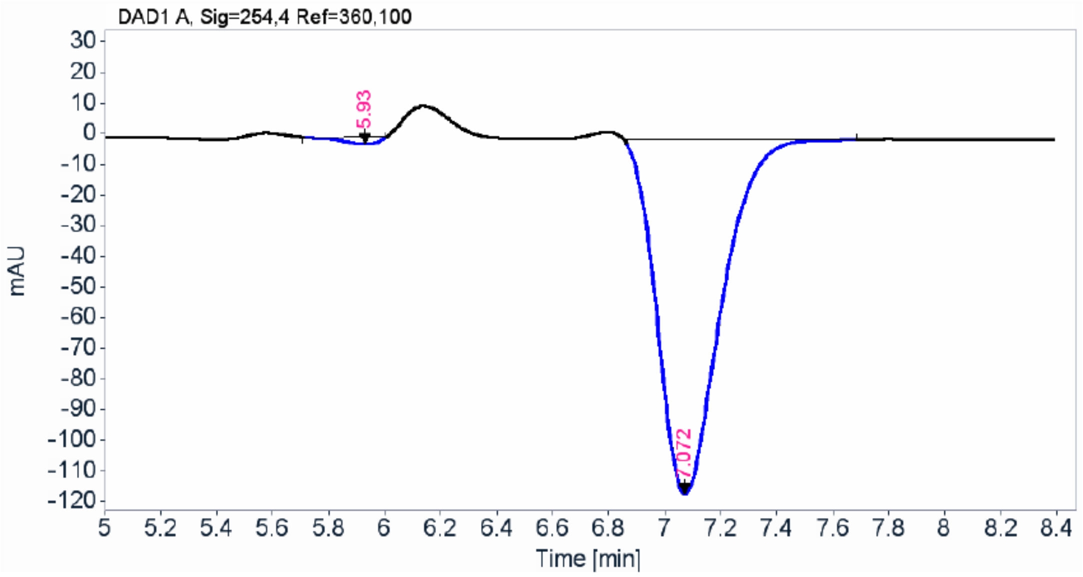

Signal:	DAD1 A, Sig=254,4 Ref=360,100				
RT [min]	Type	Width [min]	Area	Height	Area%
5.930	PM N	0.1470	21.025	2.3841	1.25
7.072	PM N	0.2399	1663.862	115.6015	98.75

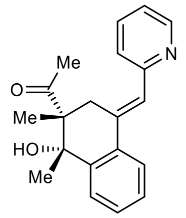

2p

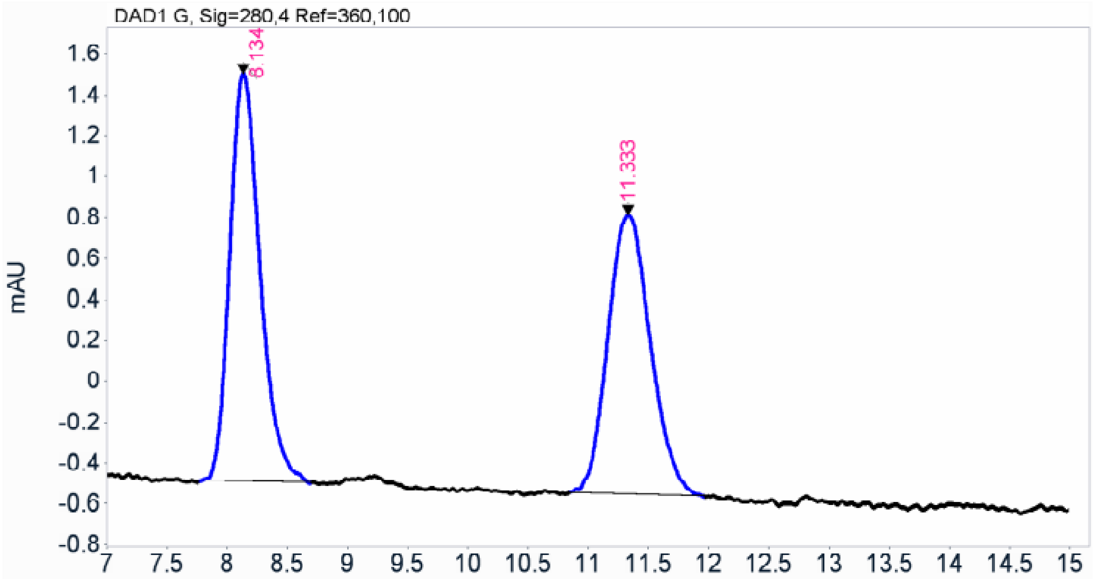

Signal: DAD1 G, Sig=280,4 Ref=360,100

RT [min]	Type	Width [min]	Area	Height	Area%
8.134	MM	0.2909	34.667	1.9863	50.84
11.333	MM	0.4095	33.517	1.3641	49.16

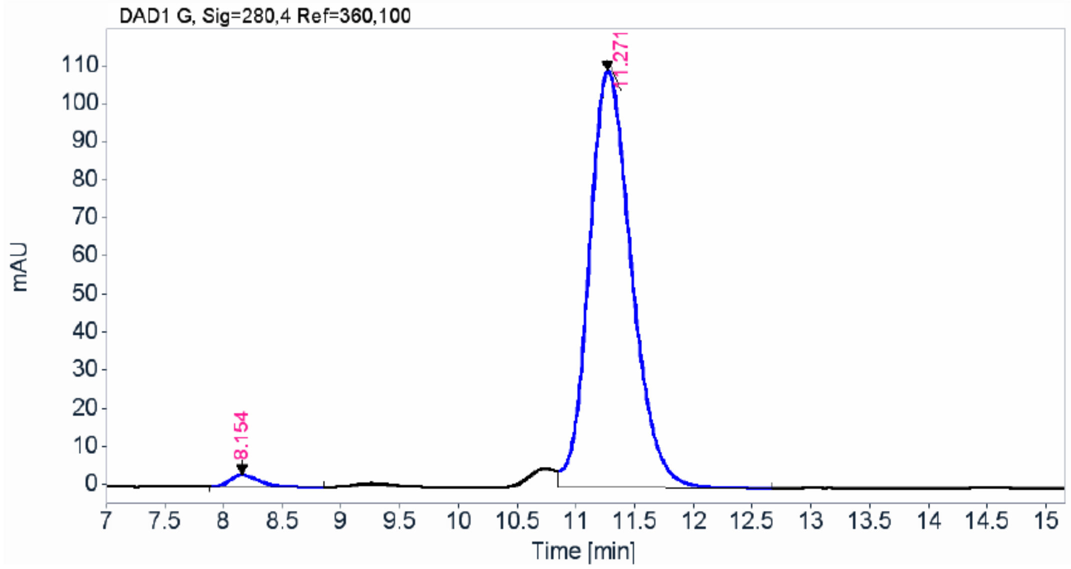

Signal: DAD1 G, Sig=280,4 Ref=360,100

RT [min]	Type	Width [min]	Area	Height	Area%
8.154	BB	0.2554	50.928	3.0721	1.82
11.271	VB	0.3862	2741.028	109.4345	98.18

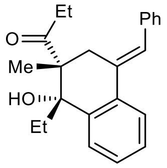

2q

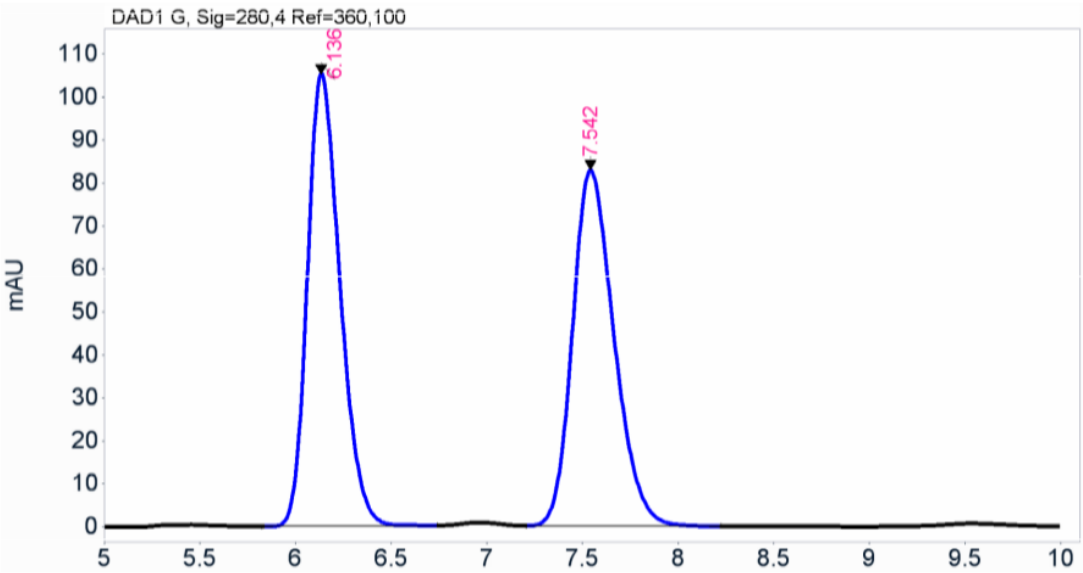

Signal:	DAD1 G, Sig=280,4 Ref=360,100				
RT [min]	Type	Width [min]	Area	Height	Area%
6.136	BB	0.1842	1246.087	105.1695	49.98
7.542	BB	0.2297	1246.845	82.7754	50.02

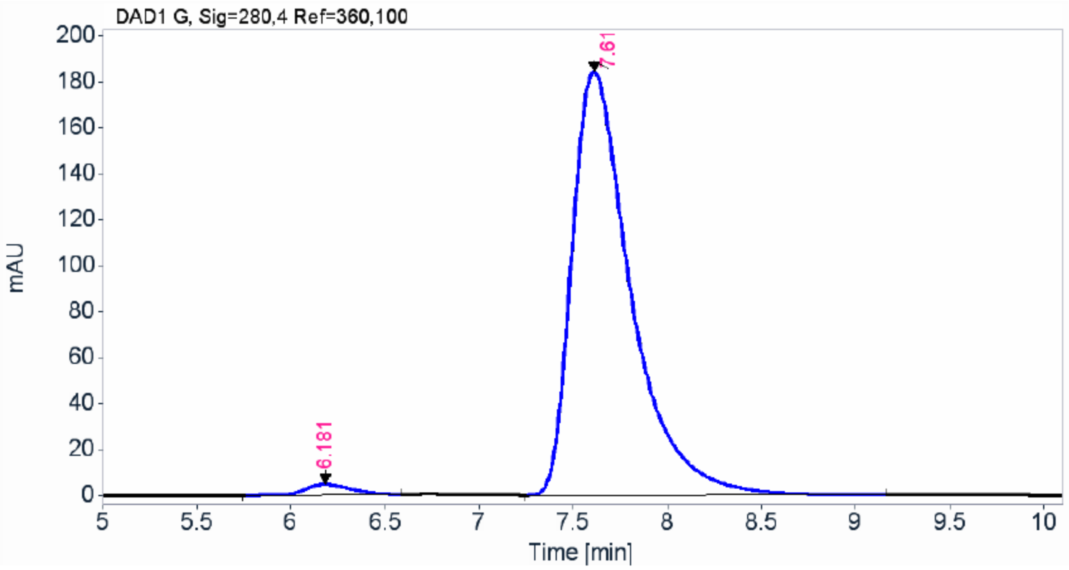

Signal:	DAD1 G, Sig=280,4 Ref=360,100				
RT [min]	Type	Width [min]	Area	Height	Area%
6.181	BB	0.2629	76.907	4.5110	1.88
7.610	BB	0.3286	4018.512	184.1144	98.12

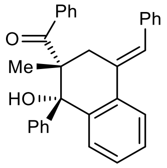

2r

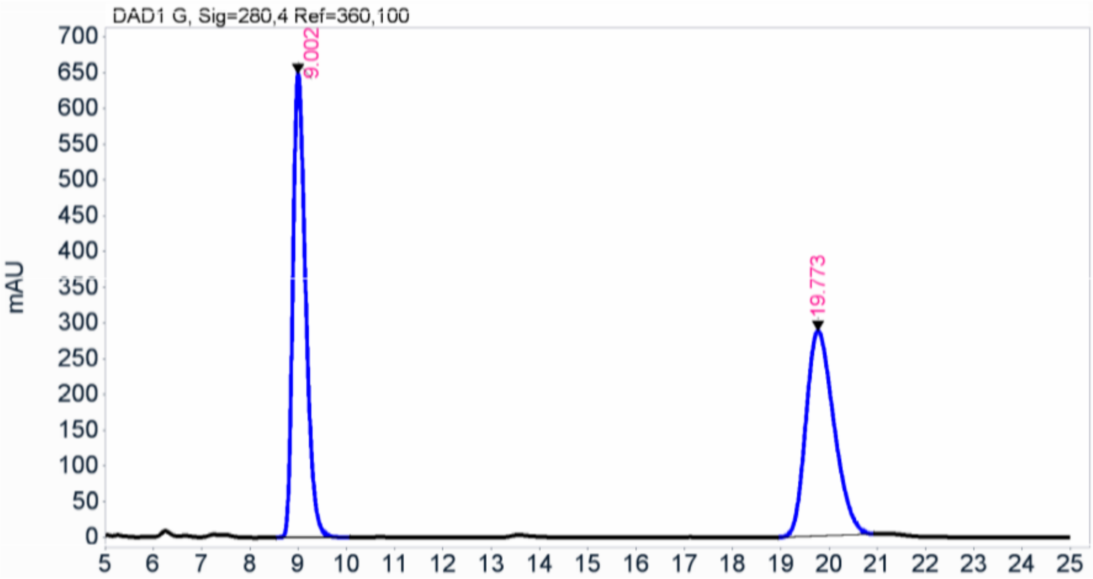

Signal:	DAD1 G, Sig=280,4 Ref=360,100				
RT [min]	Type	Width [min]	Area	Height	Area%
9.002	BB	0.2850	11934.448	647.6667	50.81
19.773	BB	0.6266	11553.501	286.3935	49.19

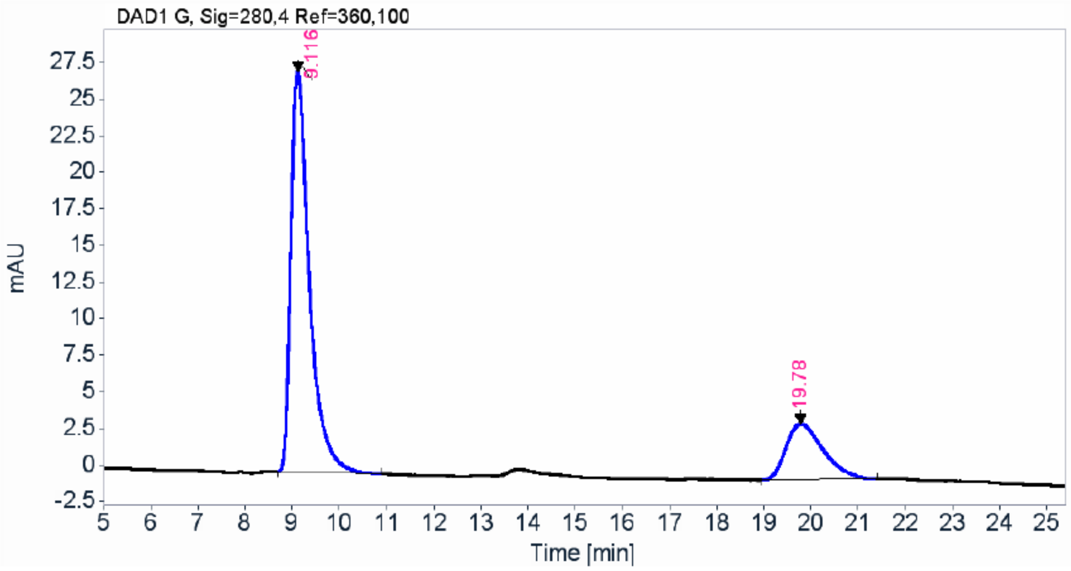

Signal:	DAD1 G, Sig=280,4 Ref=360,100				
RT [min]	Type	Width [min]	Area	Height	Area%
9.116	BB	0.4064	747.049	27.3762	78.07
19.780	BB	0.6852	209.815	3.8058	21.93

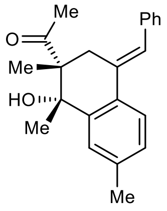

2s

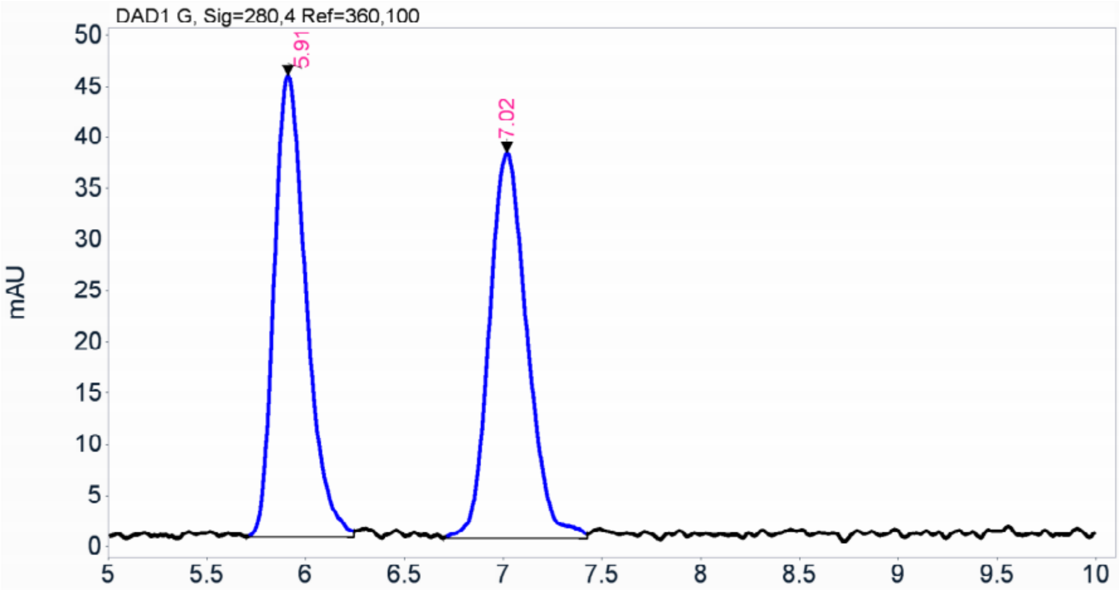

Signal:	DAD1 G, Sig=280,4 Ref=360,100				
RT [min]	Type	Width [min]	Area	Height	Area%
5.910	BV	0.1693	508.823	45.2115	50.33
7.020	BV	0.2032	502.080	37.7338	49.67

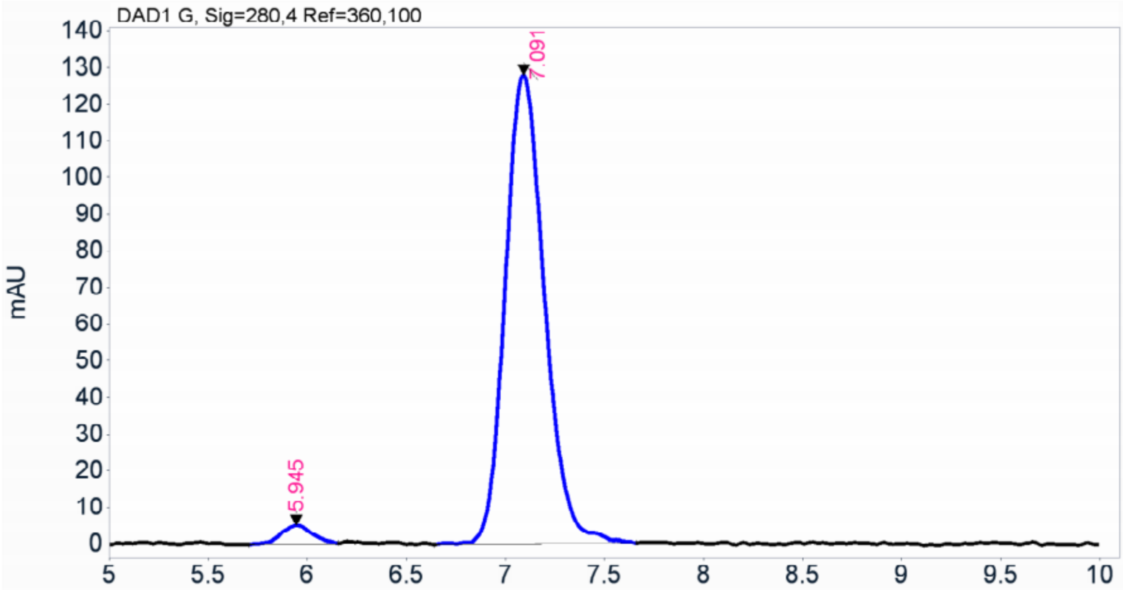

Signal:	DAD1 G, Sig=280,4 Ref=360,100				
RT [min]	Type	Width [min]	Area	Height	Area%
5.945	BV	0.1512	56.946	5.2228	3.12
7.091	VB	0.2110	1767.300	128.0538	96.88

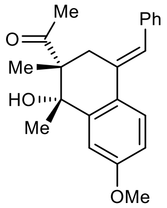

2t

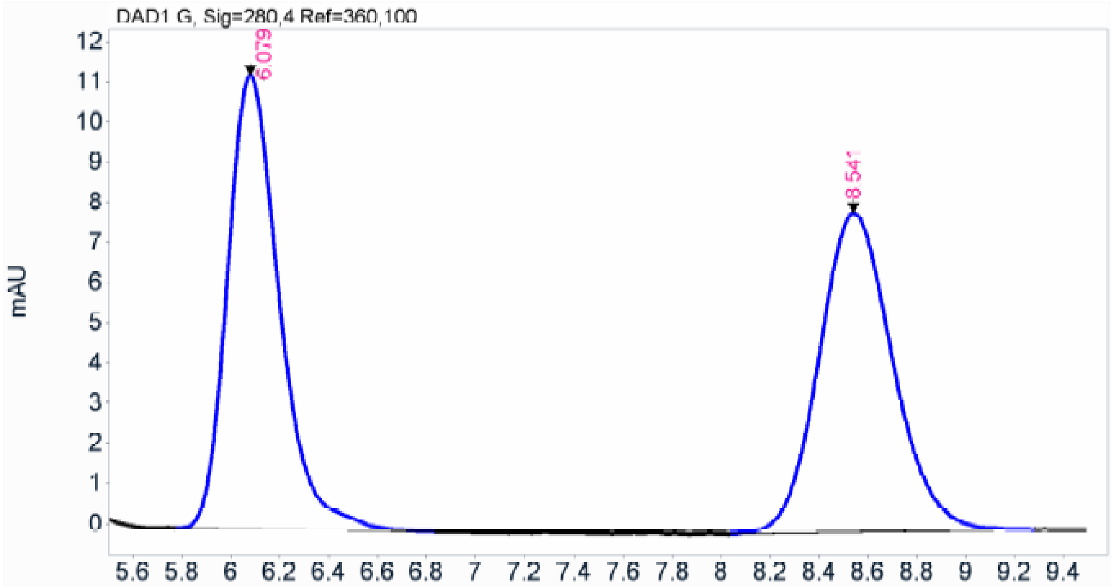

Signal:	DAD1 G, Sig=280,4 Ref=360,100				
RT [min]	Type	Width [min]	Area	Height	Area%
6.079	BB	0.2281	166.735	11.3038	50.01
8.541	BB	0.3266	166.642	7.9484	49.99

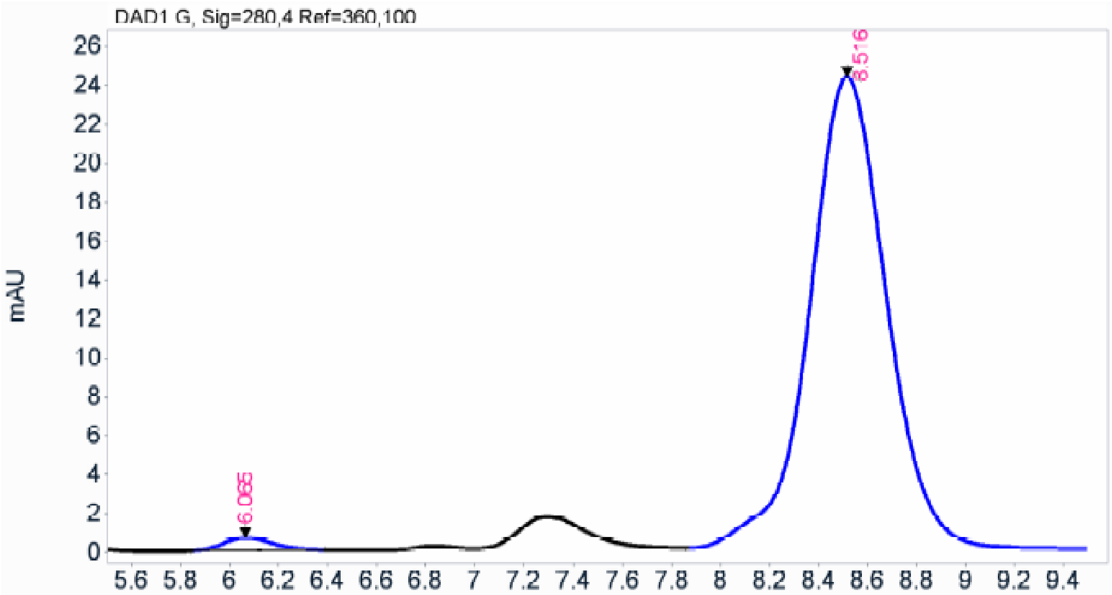

Signal:	DAD1 G, Sig=280,4 Ref=360,100				
RT [min]	Type	Width [min]	Area	Height	Area%
6.065	MM	0.2370	9.691	0.6816	1.80
8.516	BB	0.3348	529.367	24.2407	98.20

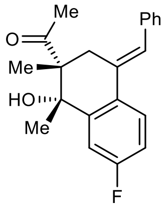

2u

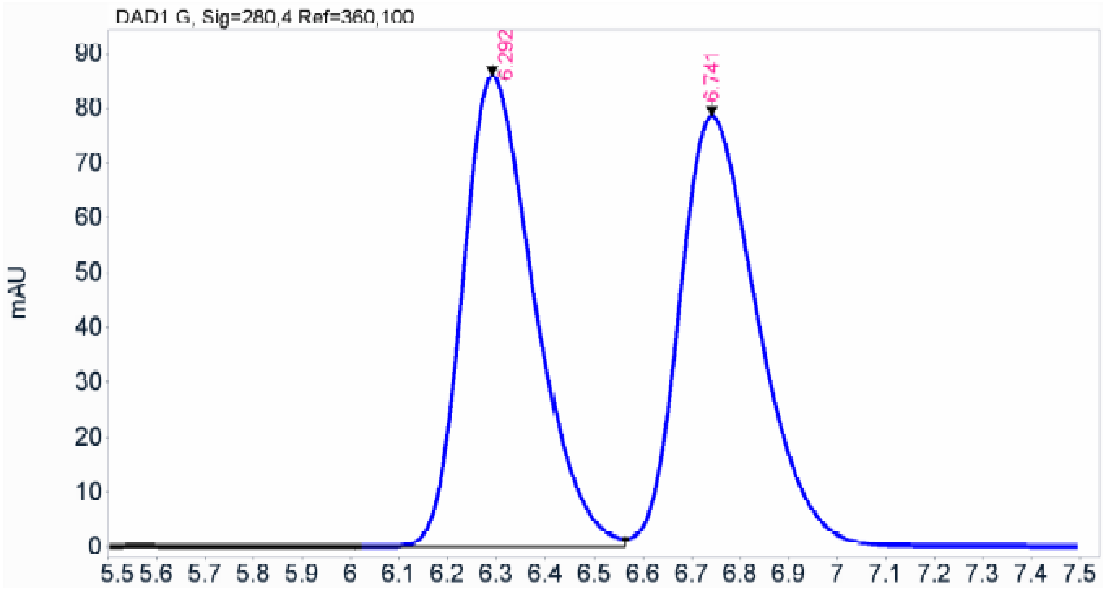

Signal:	DAD1 G, Sig=280,4 Ref=360,100				
RT [min]	Type	Width [min]	Area	Height	Area%
6.292	BV	0.1548	875.644	85.9695	49.83
6.741	VB	0.1727	881.725	78.6782	50.17

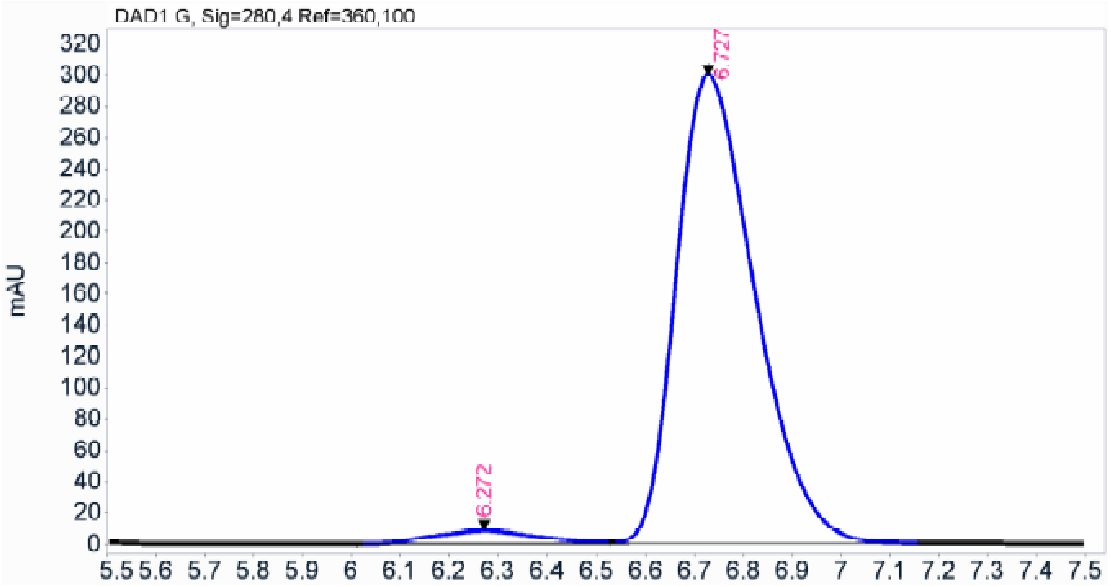

Signal:	DAD1 G, Sig=280,4 Ref=360,100				
RT [min]	Type	Width [min]	Area	Height	Area%
6.272	BV	0.2017	118.889	8.2686	3.43
6.727	VB	0.1722	3345.227	299.8261	96.57

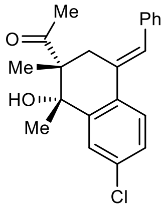

2v

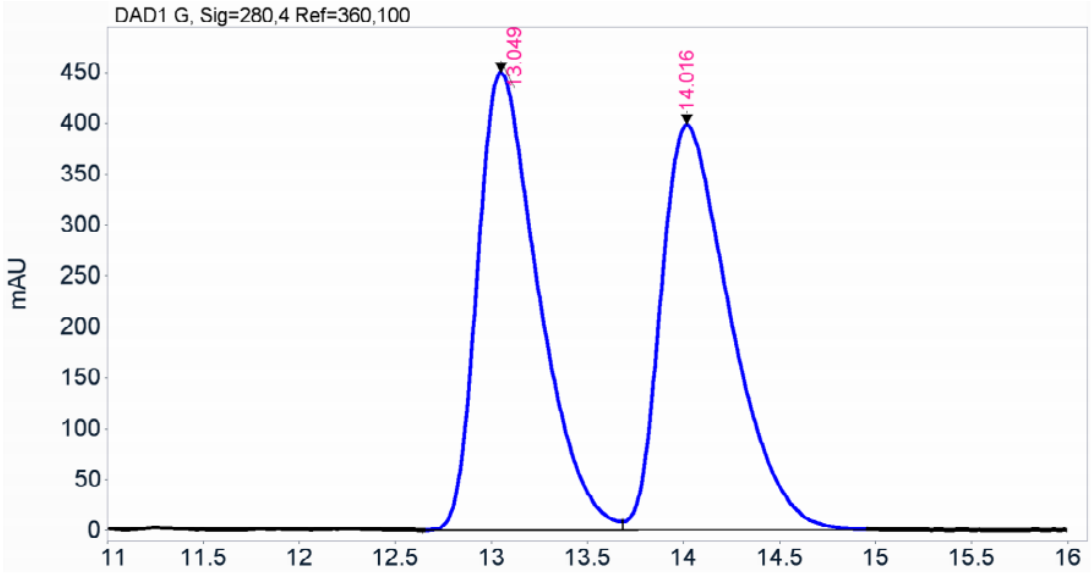

Signal:	DAD1 G, Sig=280,4 Ref=360,100				
RT [min]	Type	Width [min]	Area	Height	Area%
13.049	BV	0.3296	9933.183	449.9369	49.80
14.016	VV	0.3672	10013.787	398.3950	50.20

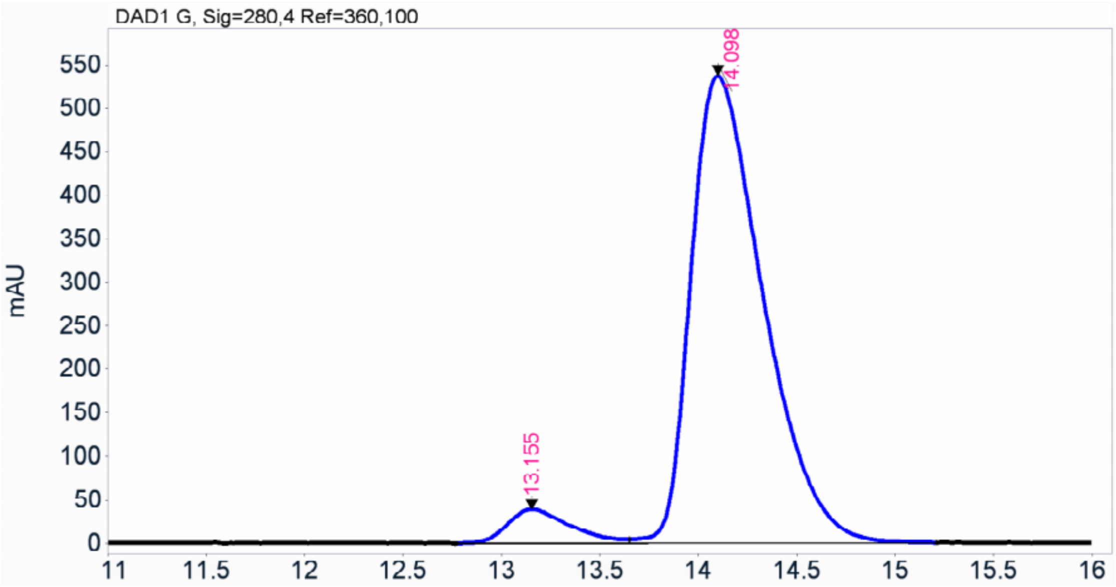

Signal:	DAD1 G, Sig=280,4 Ref=360,100				
RT [min]	Type	Width [min]	Area	Height	Area%
13.155	BV	0.3152	863.853	39.1882	5.93
14.098	VV	0.3834	13712.933	537.9197	94.07

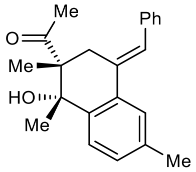

2x

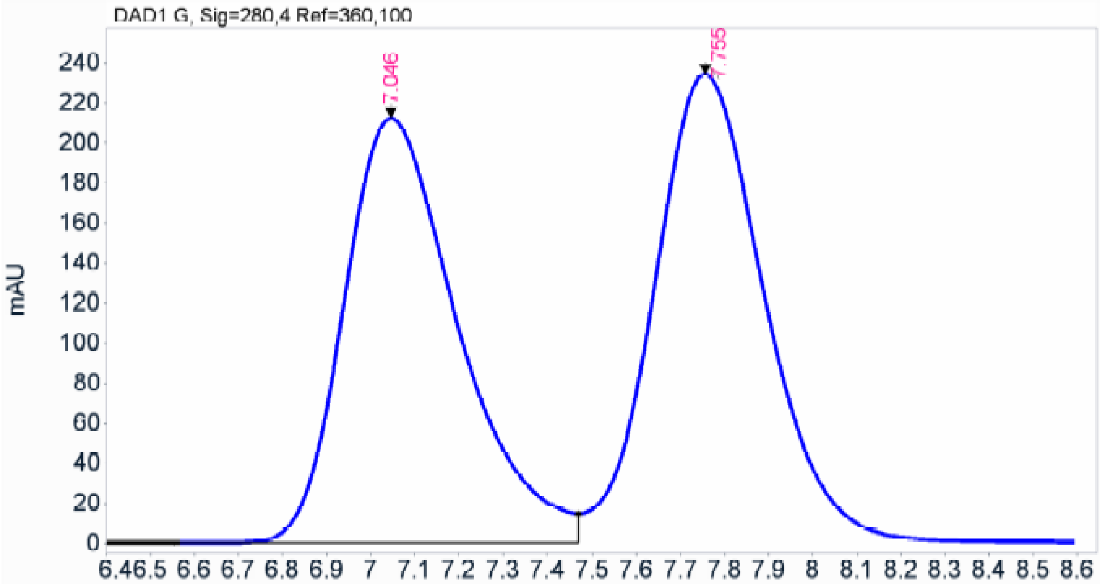

**Signal:**	DAD1 G, Sig=280,4 Ref=360,100				
**RT [min]**	**Type**	**Width [min]**	**Area**	**Height**	**Area%**
7.046	BV	0.2727	3799.888	212.3995	48.41
7.755	VB	0.2659	4048.956	234.0202	51.59

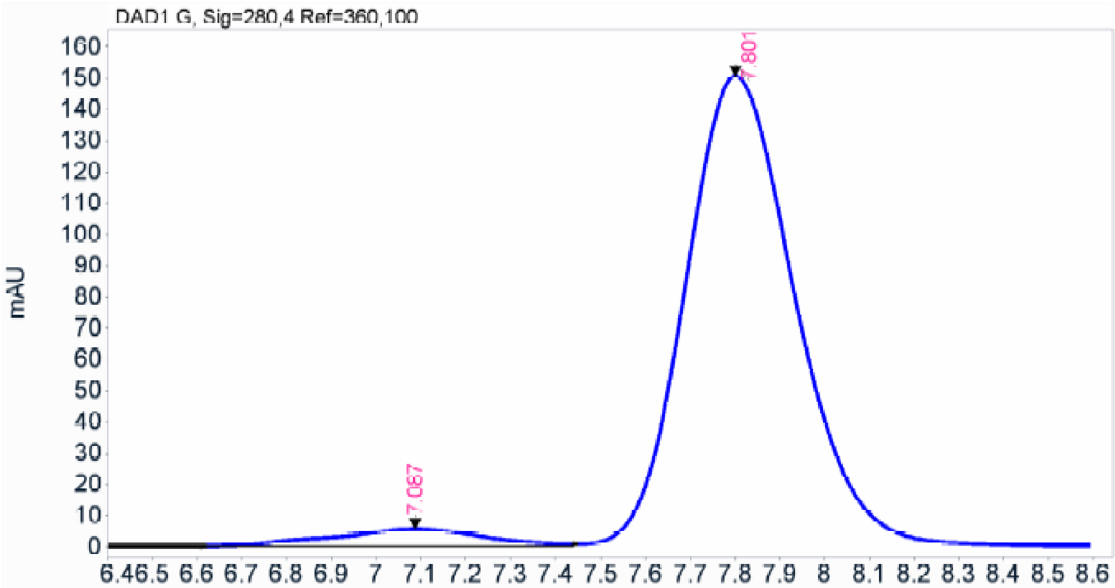

**Signal:**	DAD1 G, Sig=280,4 Ref=360,100				
**RT [min]**	**Type**	**Width [min]**	**Area**	**Height**	**Area%**
7.087	BV	0.3335	127.618	5.4832	4.76
7.801	VB	0.2640	2553.390	150.4275	95.24

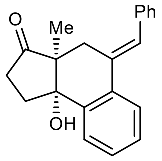

5a

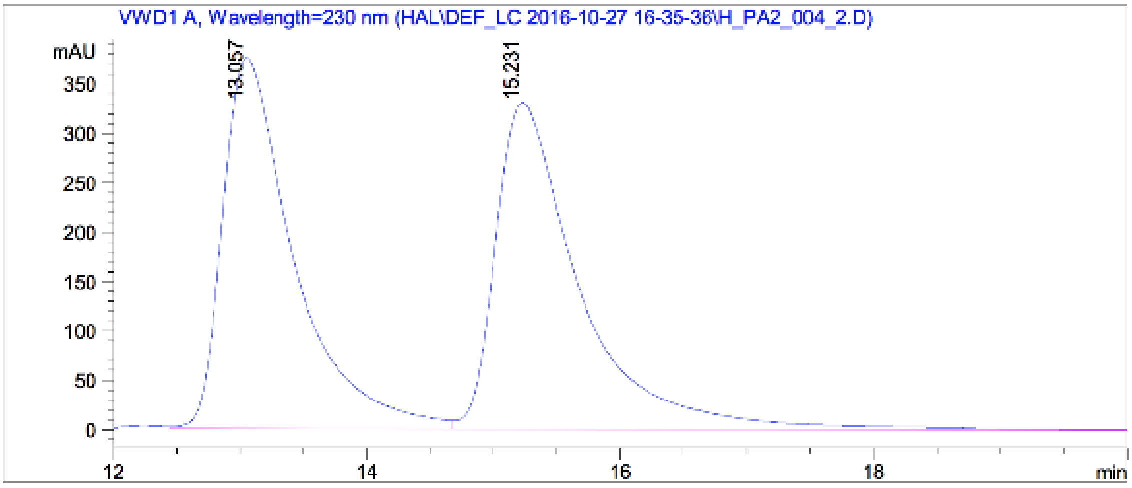

Meas. R	Area %	Width	Symmetr.
13.057	48.647	0.577	0.476
15.231	51.353	0.683	0.424

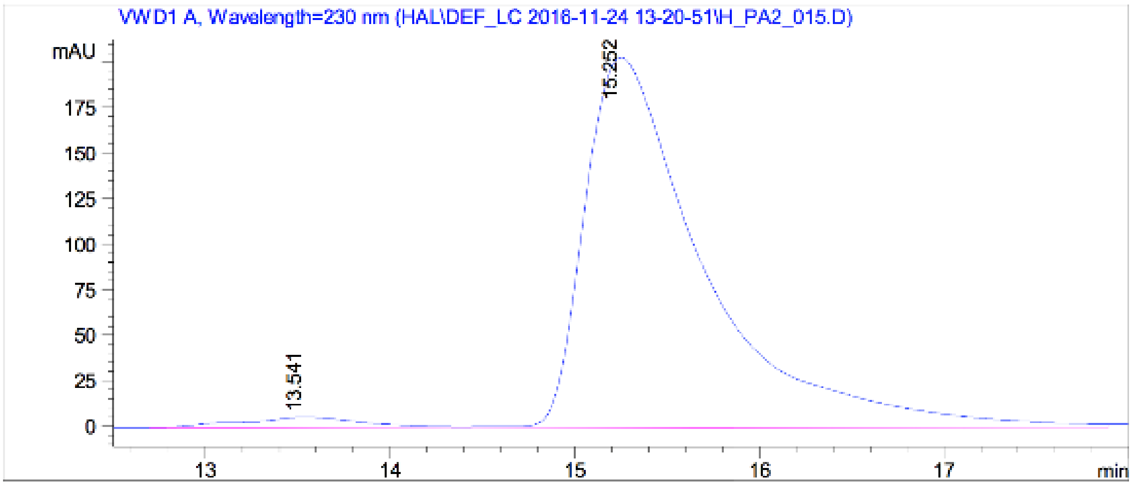

Meas. R	Area %	Width	Symmetr.
13.541	2.652	0.613	1.206
15.252	97.348	0.658	0.395

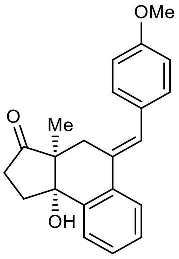

5b

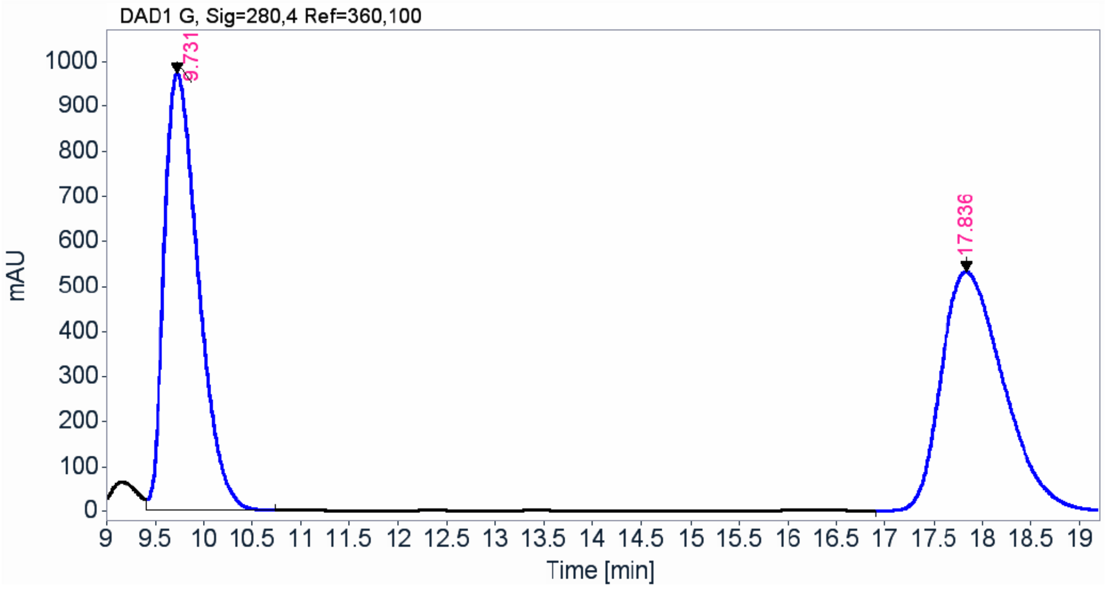

Signal:	DAD1 G, Sig=280,4 Ref=360,100				
RT [min]	Type	Width [min]	Area	Height	Area%
9.731	VB	0.3813	23577.910	970.8066	50.01
17.836	BB	0.6946	23564.064	532.1541	49.99

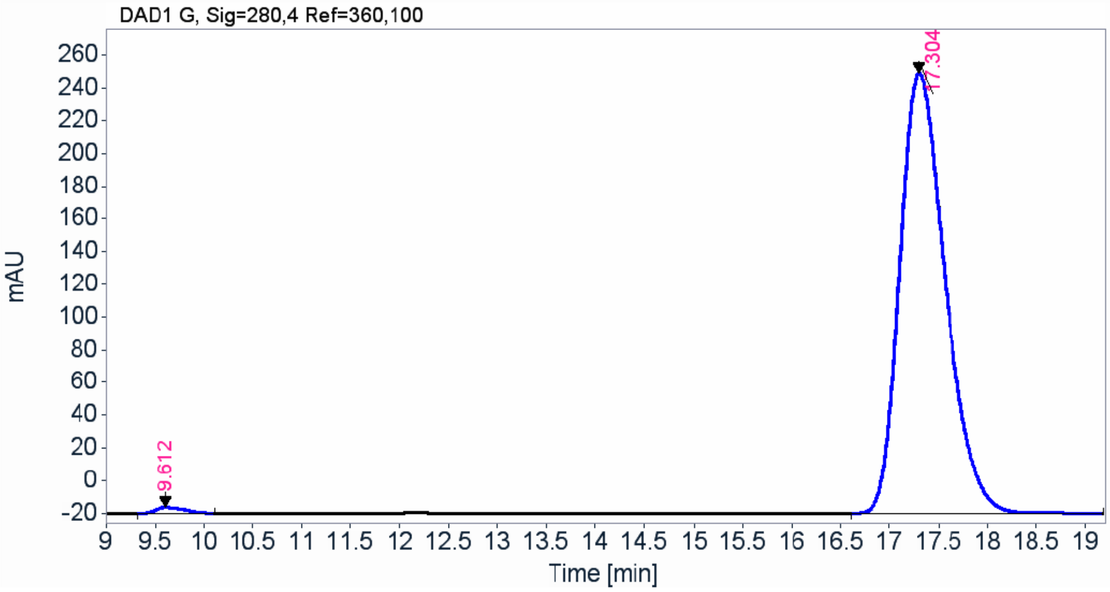

Signal:	DAD1 G, Sig=280,4 Ref=360,100				
RT [min]	Type	Width [min]	Area	Height	Area%
9.612	BB	0.2873	75.552	3.6830	0.87
17.304	BB	0.4934	8615.665	269.1923	99.13

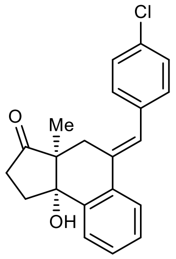

5c

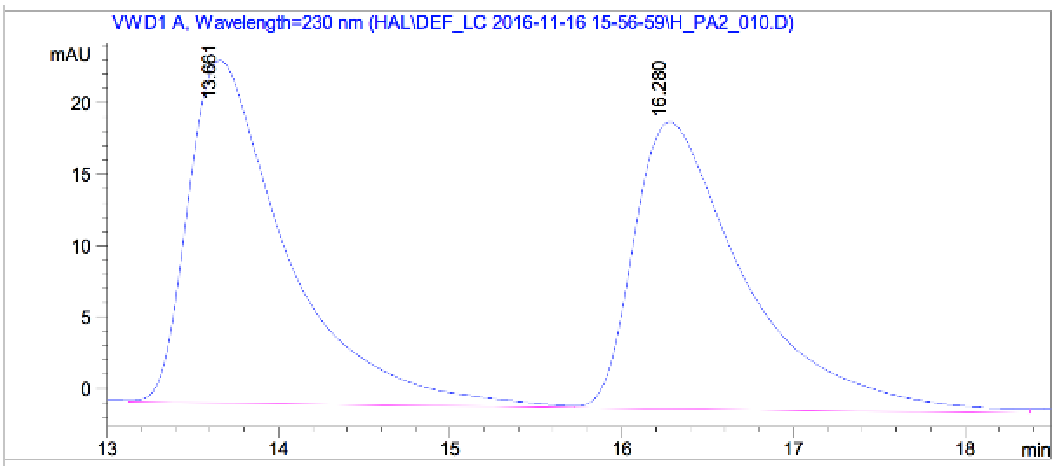

Meas. R	Area %	Width	Symmetr.
13.661	51.360	0.583	0.444
16.280	48.640	0.665	0.436

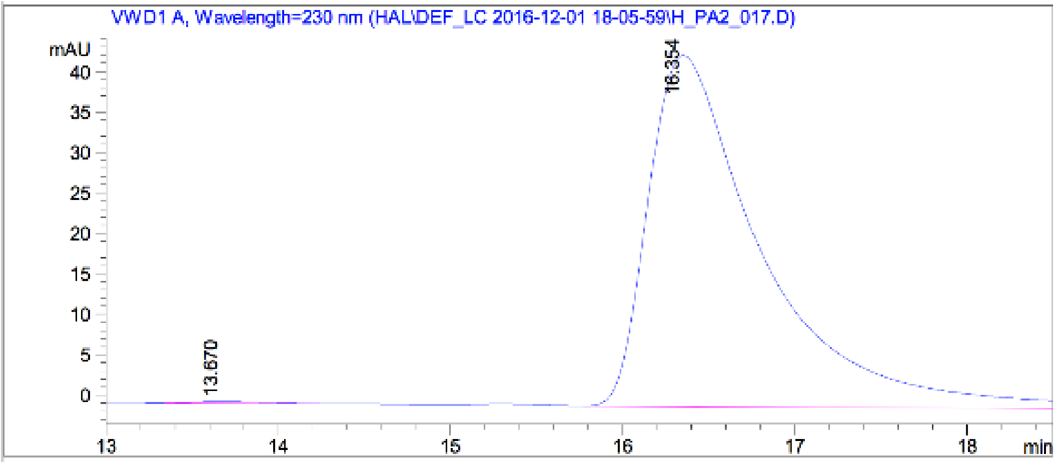

Meas. R	Area %	Width	Symmetr.
13.670	0.339	0.453	0.682
16.354	99.661	0.676	0.397

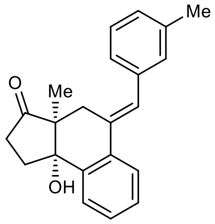

5d

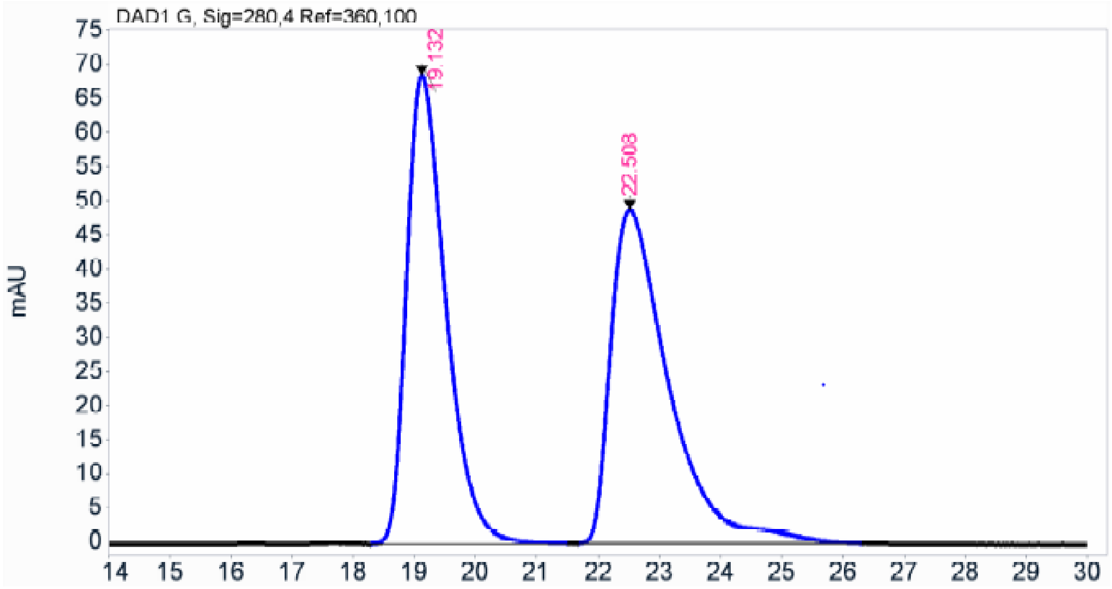

Signal:	DAD1 G, Sig=280,4 Ref=360,100				
RT [min]	Type	Width [min]	Area	Height	Area%
19.132	BB	0.6743	3064.483	68.7132	48.68
22.508	BB	0.9595	3231.097	48.8625	51.32

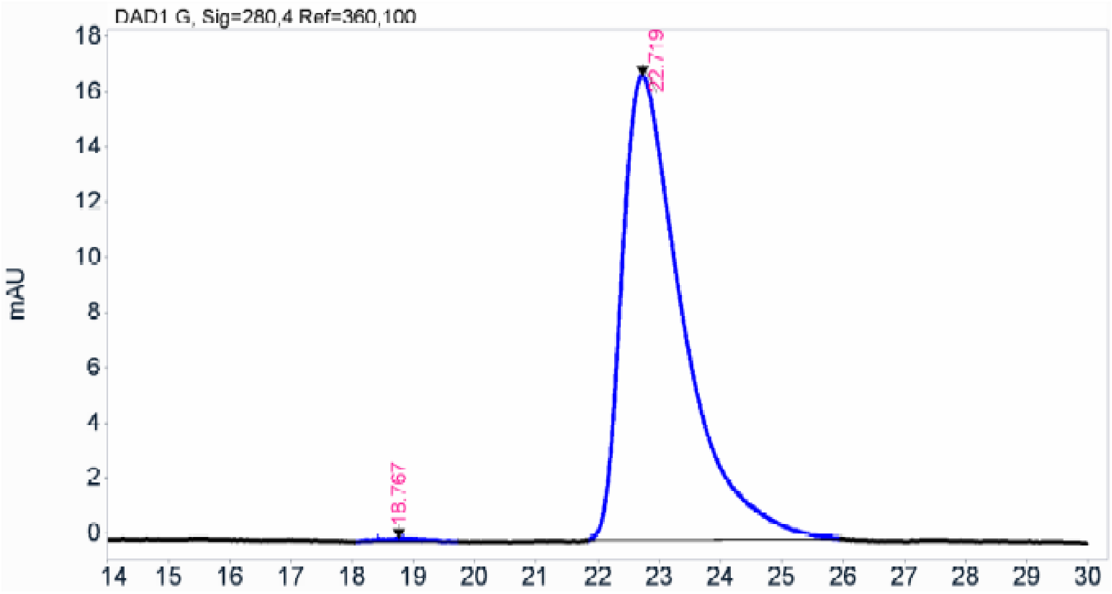

Signal:	DAD1 G, Sig=280,4 Ref=360,100				
RT [min]	Type	Width [min]	Area	Height	Area%
18.767	MM	0.9379	5.455	0.0969	0.47
22.719	BB	0.9691	1164.515	16.8323	99.53

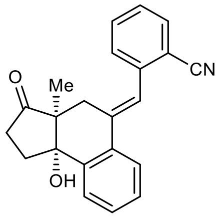

5e

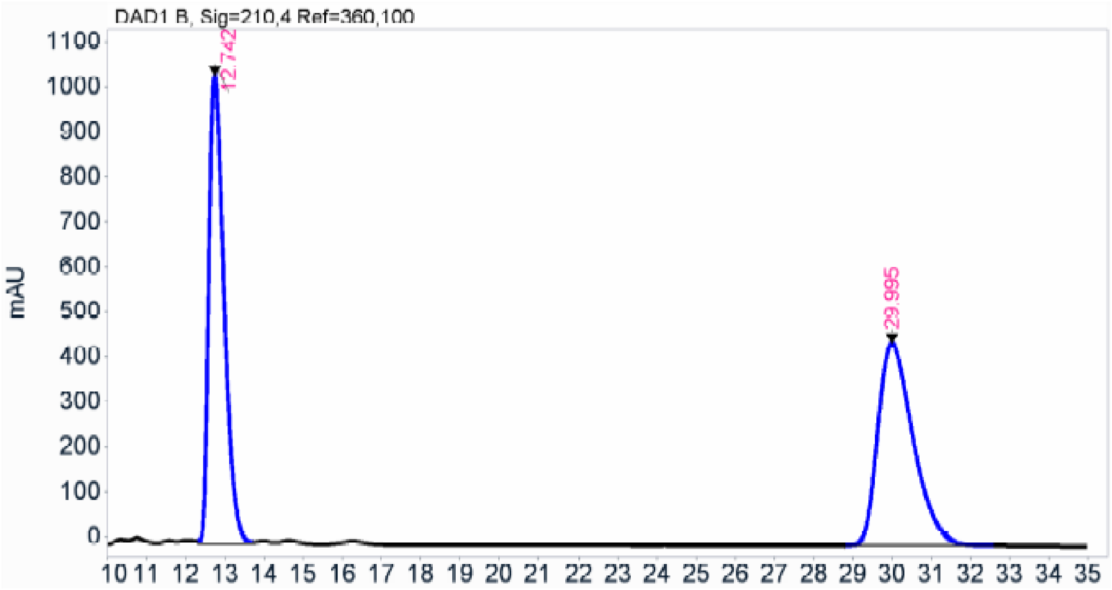

Signal: DAD1 B, Sig=210,4 Ref=360,100

RT [min]	Type	Width [min]	Area	Height	Area%
12.742	VV	0.4196	28137.887	1040.1501	50.00
29.995	BB	0.9488	28134.932	449.1726	50.00

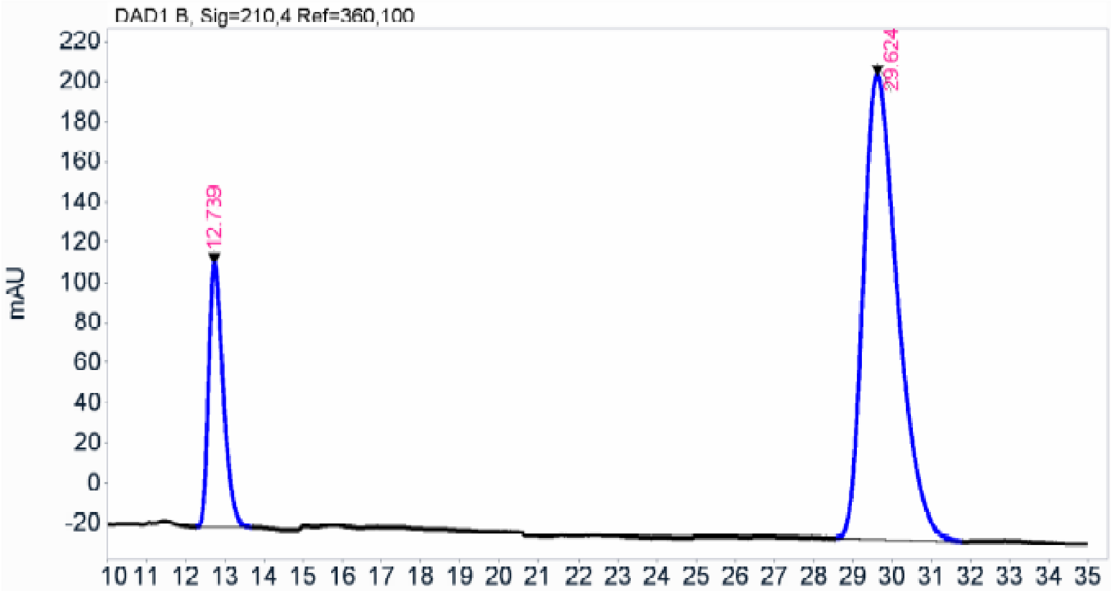

Signal: DAD1 B, Sig=210,4 Ref=360,100

RT [min]	Type	Width [min]	Area	Height	Area%
12.739	BB	0.3933	3382.737	130.9917	19.35
29.624	BB	0.8870	14094.945	231.2712	80.65

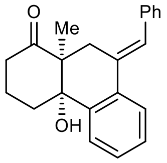

5f

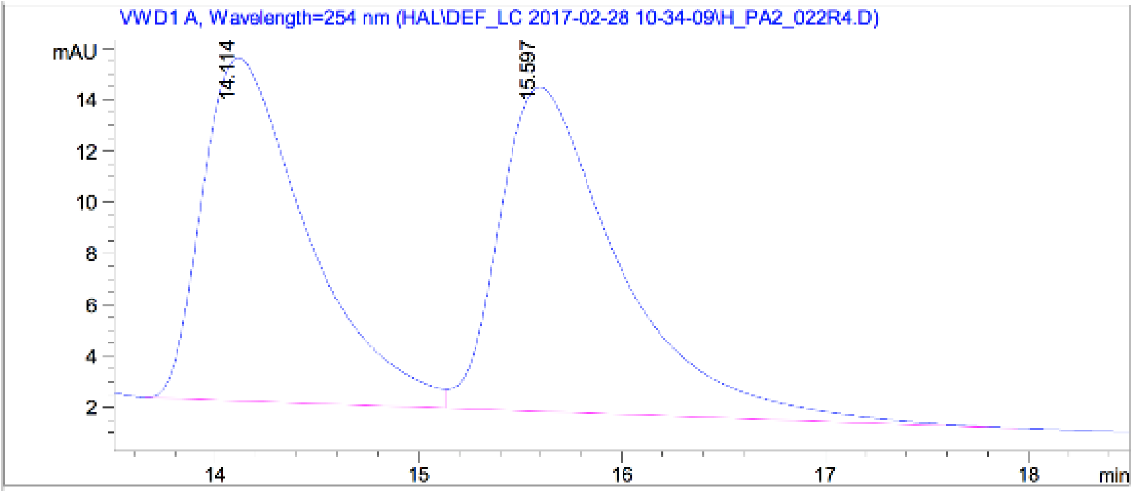

Meas. R	Area %	Width	Symmetr.
14.114	48.063	0.545	0.487
15.597	51.937	0.609	0.476

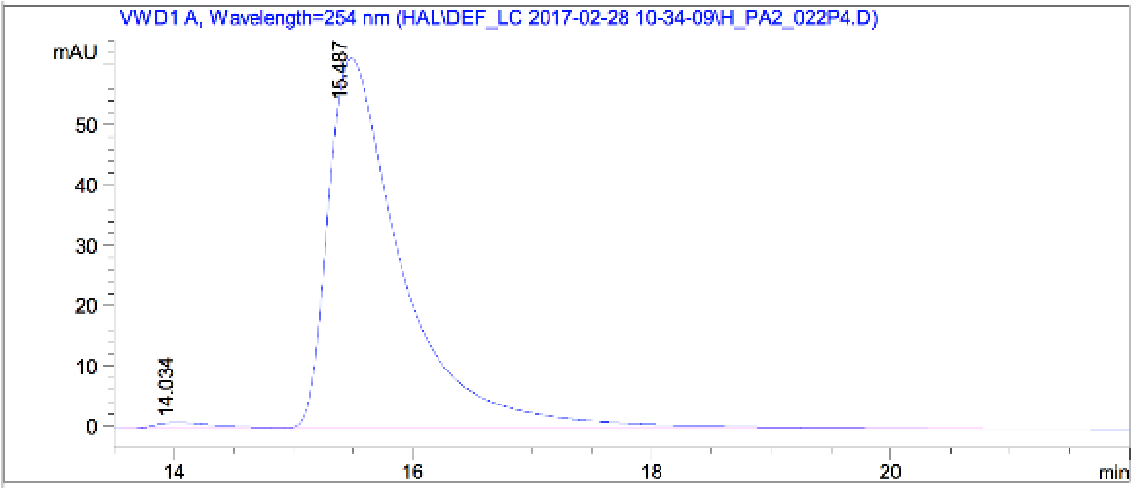

Meas. R	Area %	Width	Symmetr.
14.034	1.424	0.547	0.468
15.487	98.576	0.638	0.406

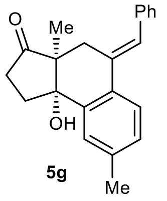

5g

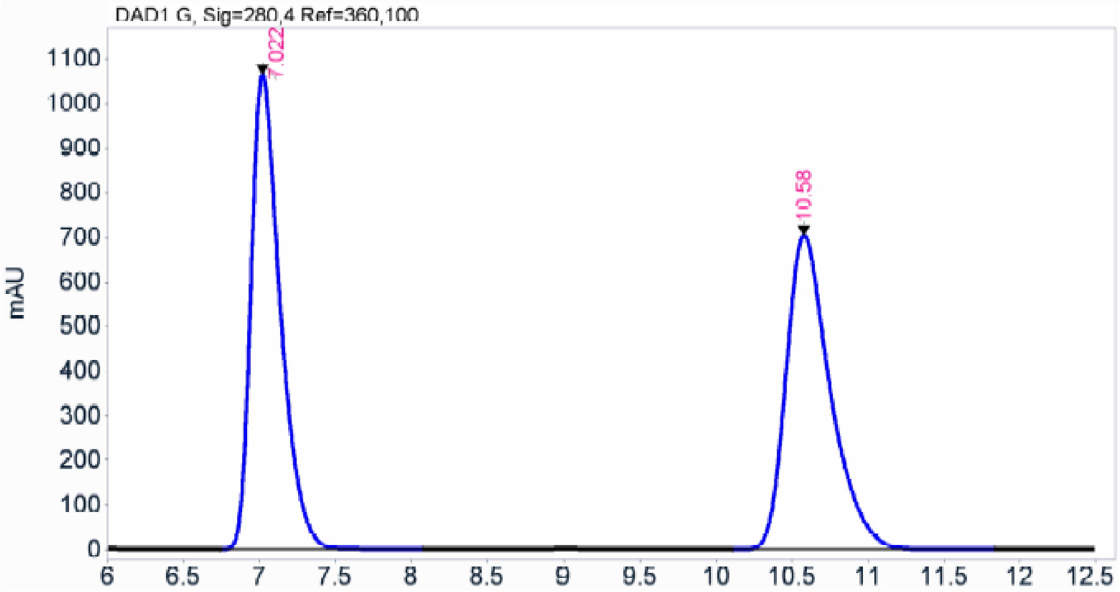

Signal:	DAD1 G, Sig=280,4 Ref=360,100				
RT [min]	Type	Width [min]	Area	Height	Area%
7.022	BB	0.2000	14240.782	1064.7043	50.01
10.580	BB	0.3037	14234.305	704.5869	49.99

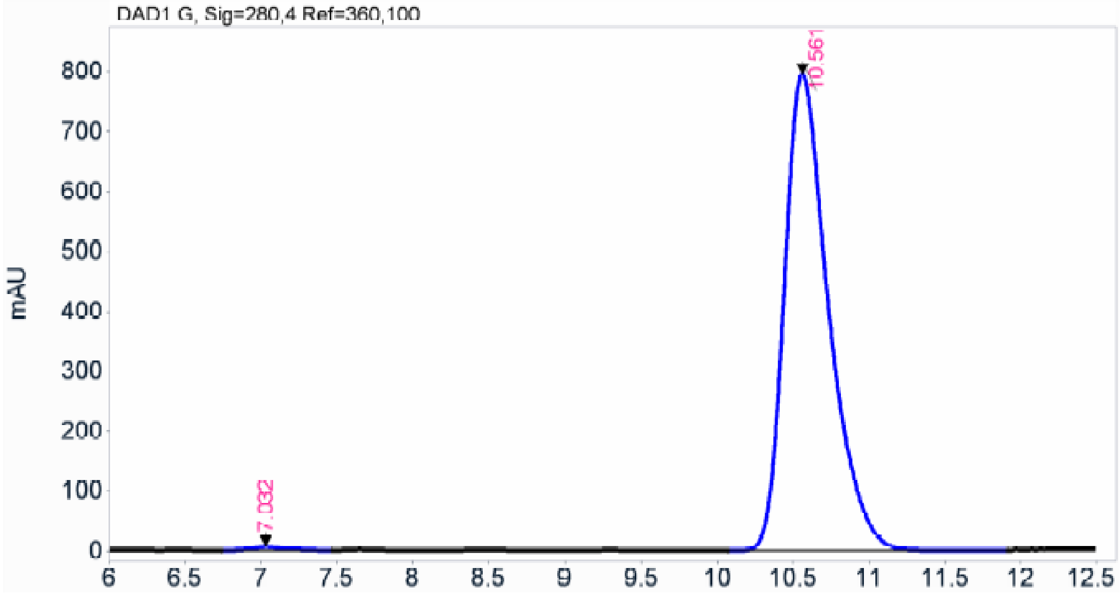

Signal:	DAD1 G, Sig=280,4 Ref=360,100				
RT [min]	Type	Width [min]	Area	Height	Area%
7.032	BB	0.2154	97.077	6.5322	0.60
10.561	BB	0.3039	16085.202	795.5165	99.40

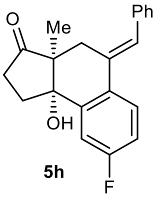

5h

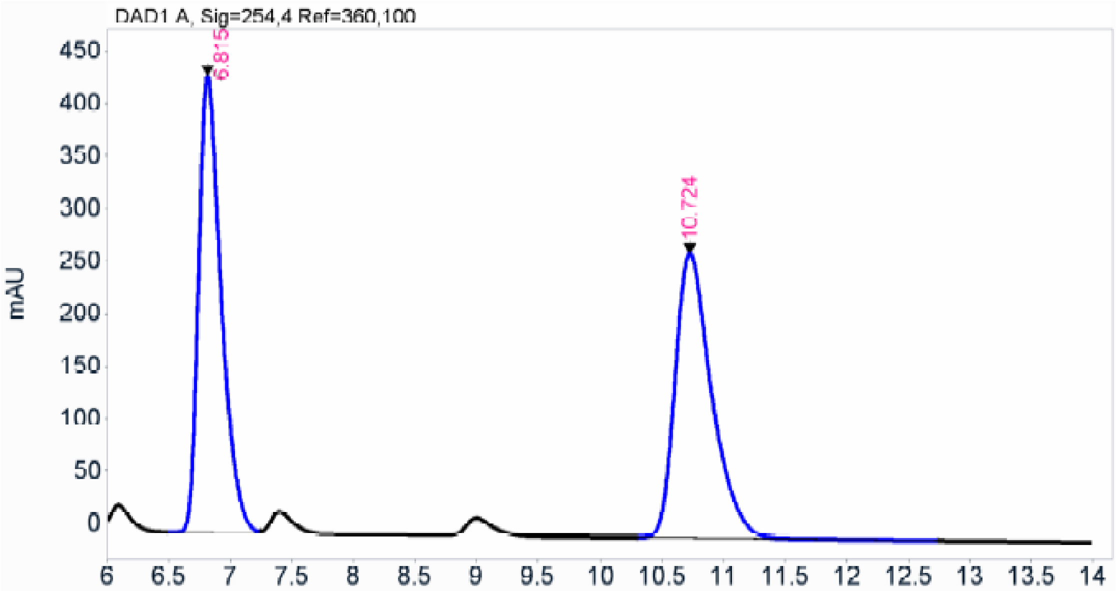

Signal:	DAD1 A, Sig=254,4 Ref=360,100				
RT [min]	Type	Width [min]	Area	Height	Area%
6.815	BV	0.1911	5552.653	434.4767	49.90
10.724	BBA	0.3103	5574.548	270.6377	50.10

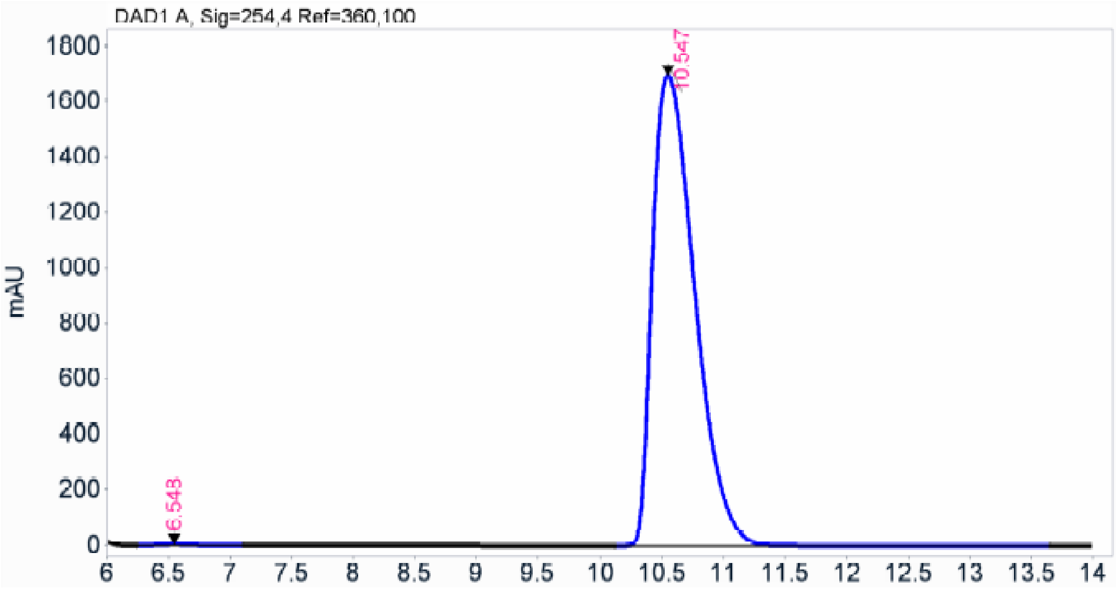

Signal:	DAD1 A, Sig=254,4 Ref=360,100				
RT [min]	Type	Width [min]	Area	Height	Area%
6.548	BB	0.2179	55.128	3.5760	0.14
10.547	BB	0.3760	40437.633	1696.7682	99.86

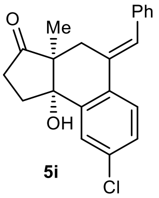

5i

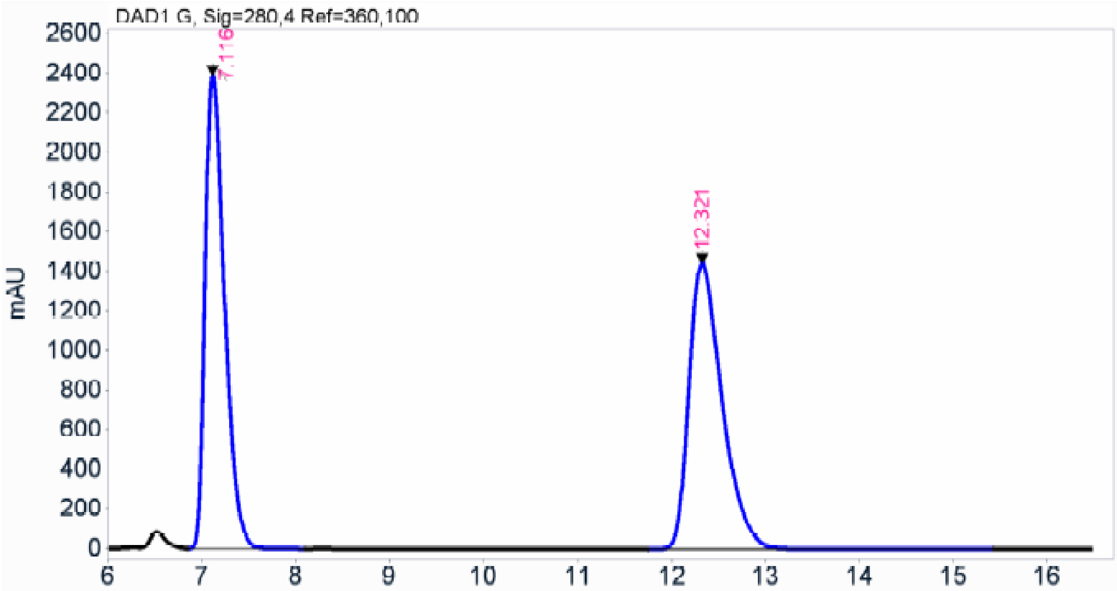

Signal:	DAD1 G, Sig=280,4 Ref=360,100				
RT [min]	Type	Width [min]	Area	Height	Area%
7.116	VB	0.2195	34297.738	2388.9548	49.19
12.321	BB	0.3750	35427.527	1440.2213	50.81

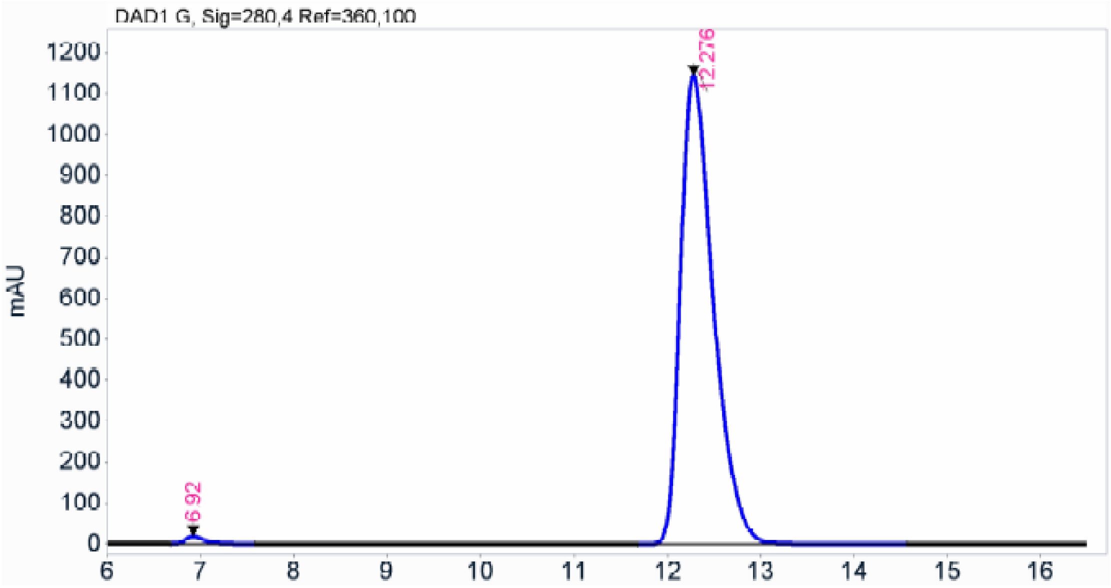

Signal:	DAD1 G, Sig=280,4 Ref=360,100				
RT [min]	Type	Width [min]	Area	Height	Area%
6.920	BB	0.2038	261.525	19.0904	0.92
12.276	BB	0.3744	28118.533	1145.3540	99.08

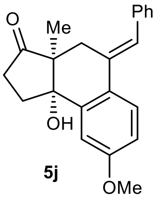

5j

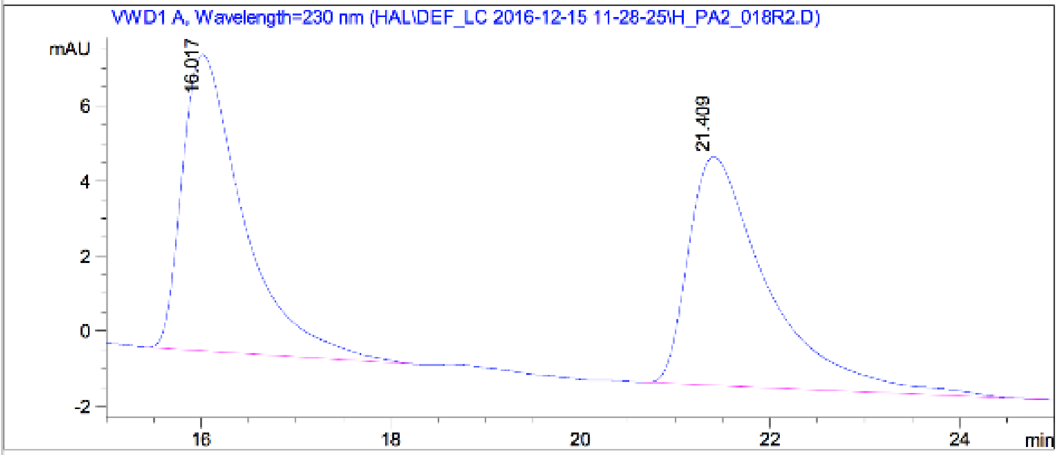

Meas. R	Area %	Width	Symmetr.
16.017	49.886	0.667	0.463
21.409	50.114	0.848	0.429

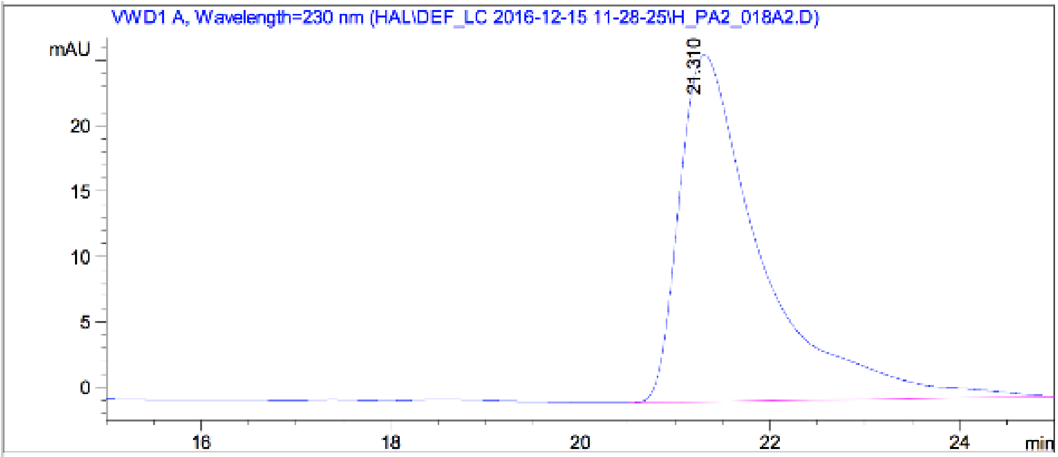

Meas. R	Area %	Width	Symmetr.
7.182	0.868	0.307	0.612
21.310	99.132	0.872	0.399

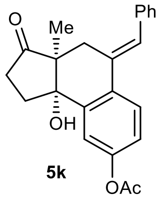

5k

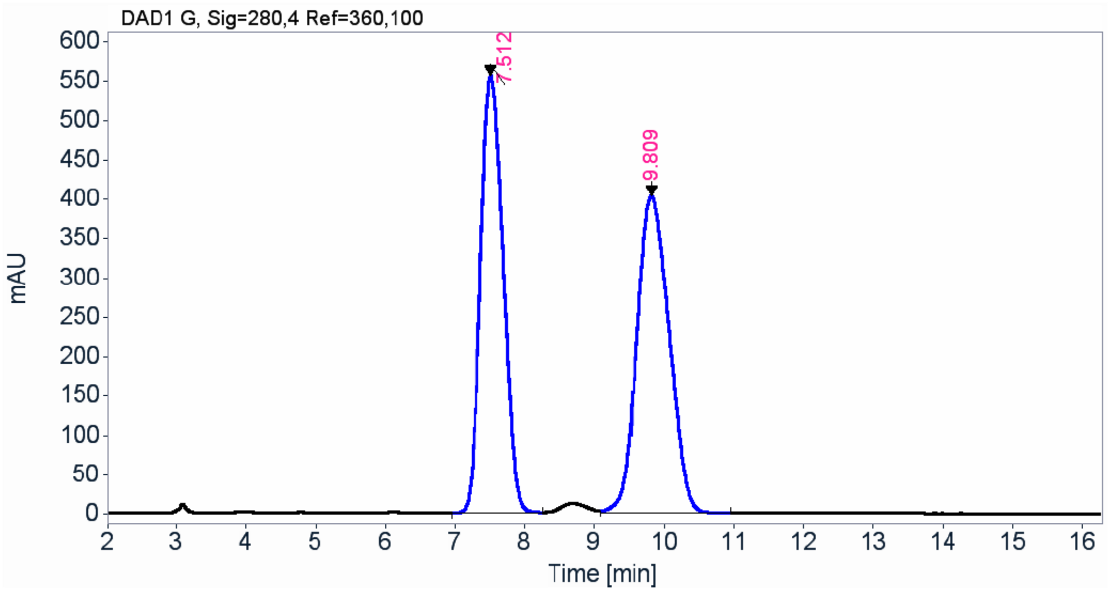

Signal:	DAD1 G, Sig=280,4 Ref=360,100				
RT [min]	Type	Width [min]	Area	Height	Area%
7.512	BB	0.3651	12532.396	555.7339	49.48
9.809	VB	0.5133	12796.297	402.0919	50.52

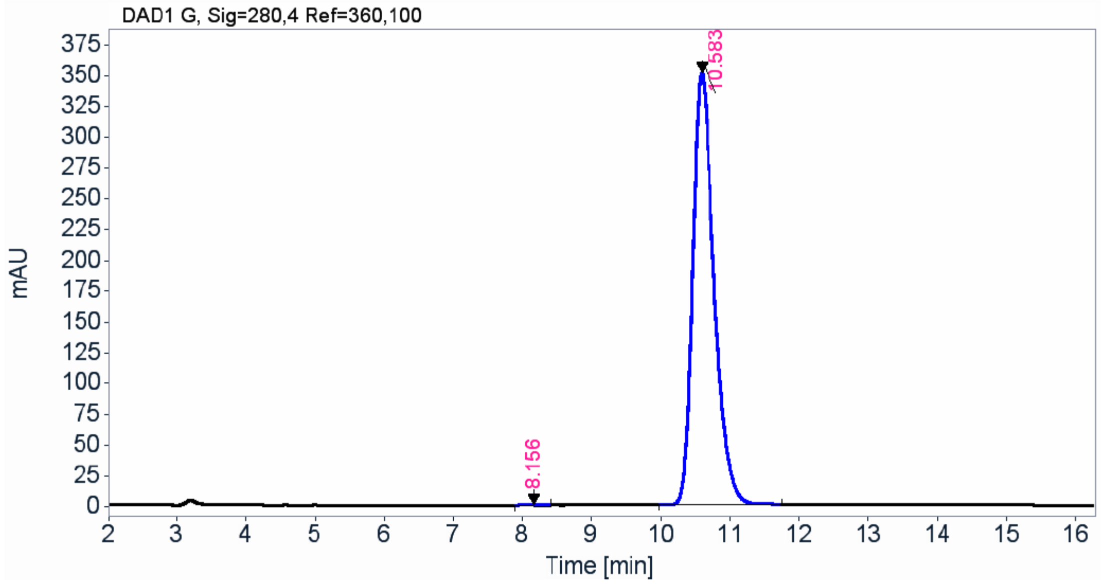

Signal:	DAD1 G, Sig=280,4 Ref=360,100				
RT [min]	Type	Width [min]	Area	Height	Area%
8.156	MM	0.4294	43.995	1.7076	0.57
10.583	BB	0.3290	7608.078	350.8091	99.43

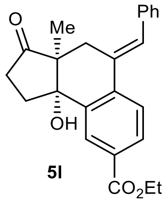

5l

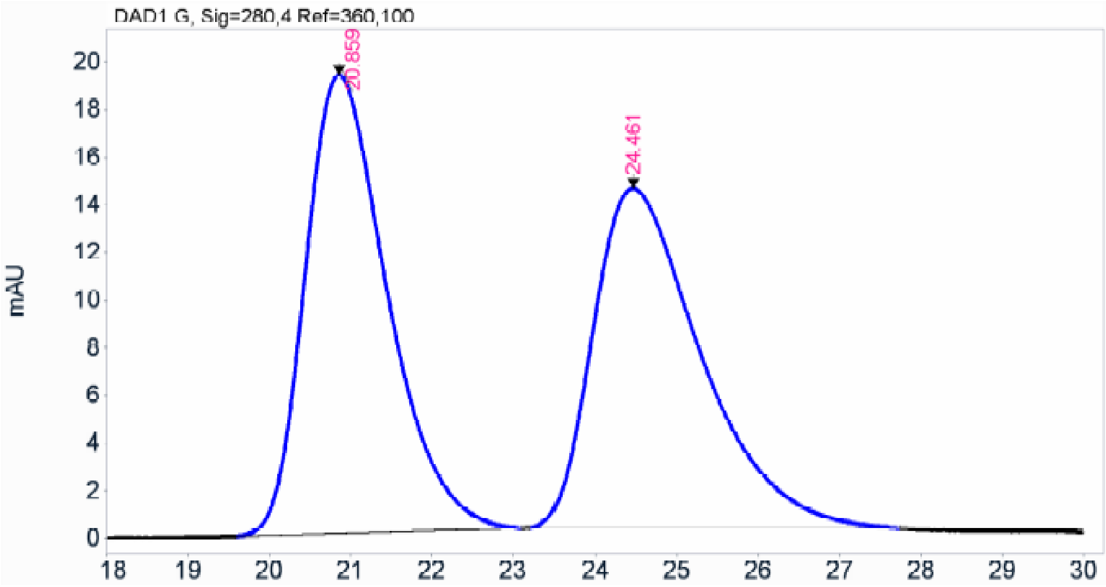

Signal: DAD1 G, Sig=280,4 Ref=360,100

RT [min]	Type	Width [min]	Area	Height	Area%
20.859	BB	1.0235	1348.112	19.2382	50.68
24.461	BB	1.1473	1311.834	14.2591	49.32

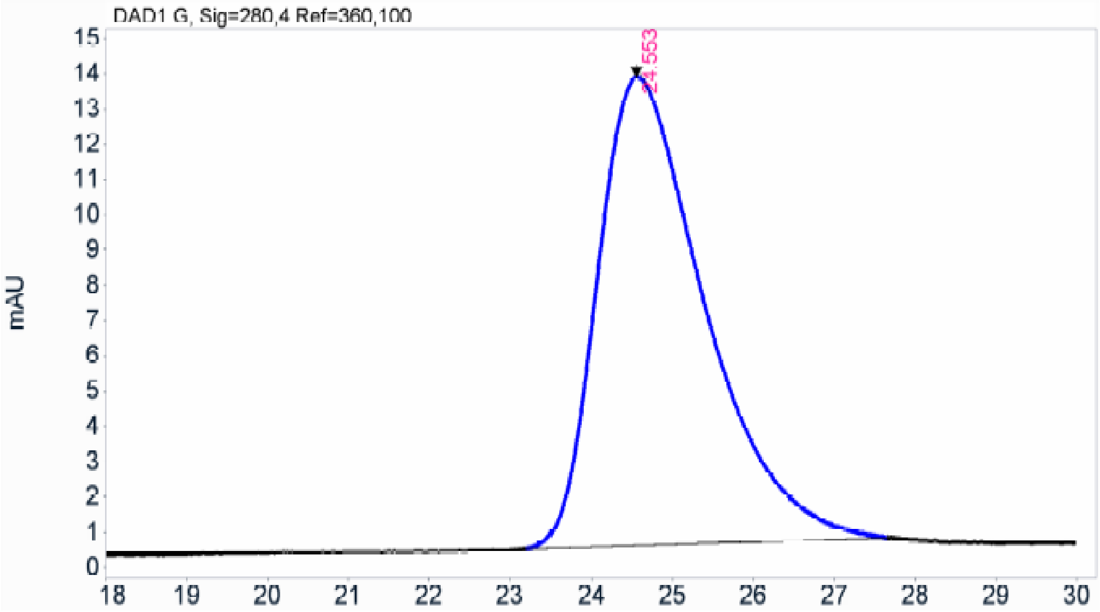

Signal: DAD1 G, Sig=280,4 Ref=360,100

RT [min]	Type	Width [min]	Area	Height	Area%
24.553	BB	1.1090	1218.466	13.2733	100.00

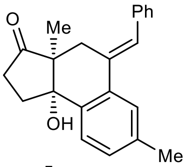

5m

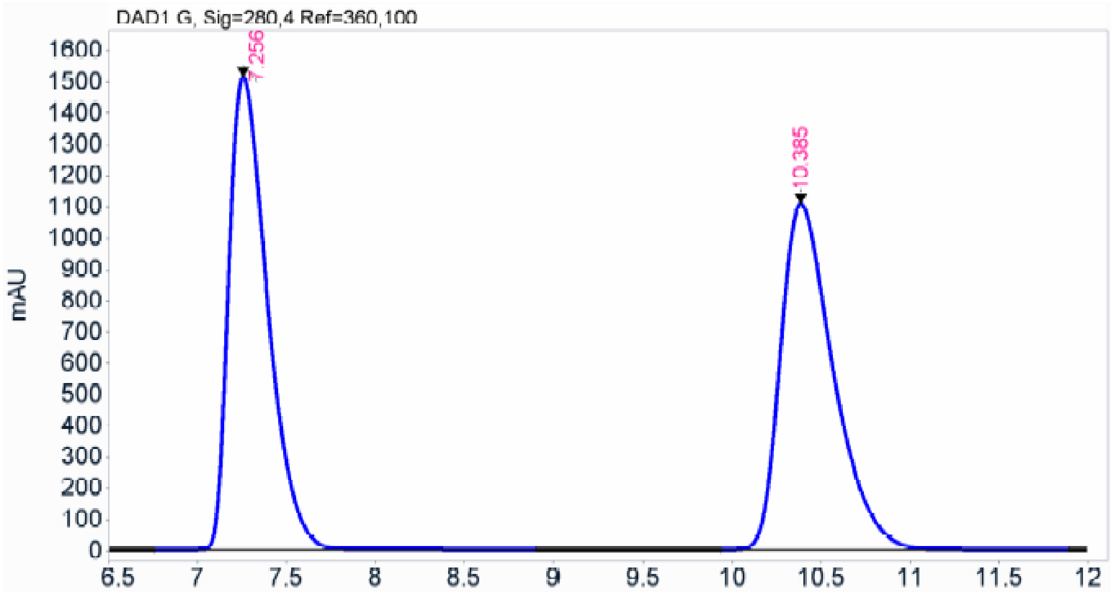

Signal: DAD1 G, Sig=280,4 Ref=360,100

RT [min]	Type	Width [min]	Area	Height	Area%
7.256	BB	0.2338	22785.963	1512.0844	49.98
10.385	BB	0.3145	22801.002	1106.3059	50.02

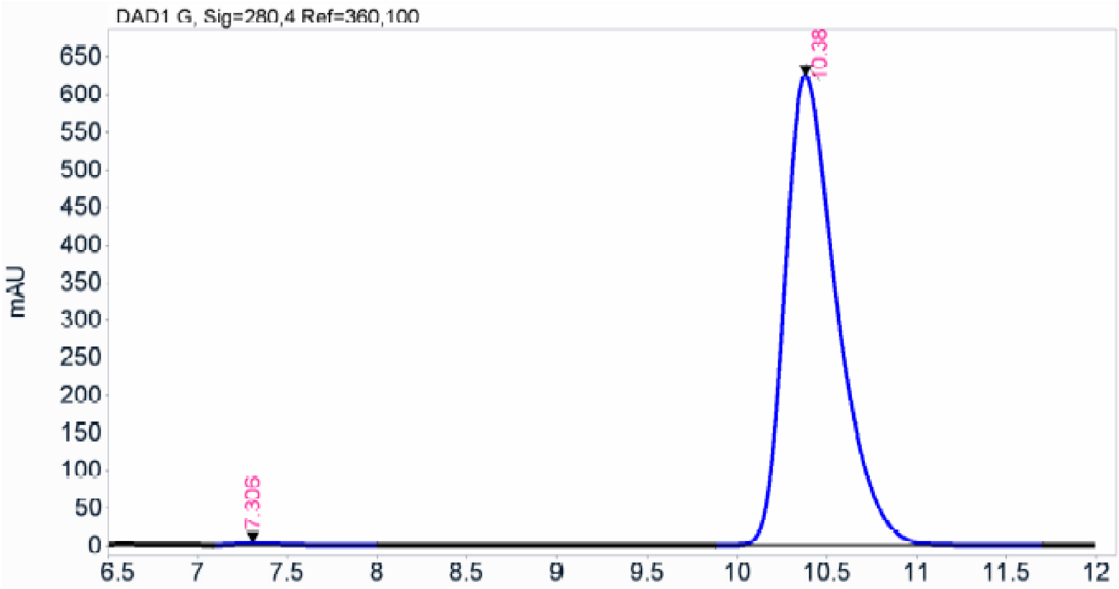

Signal: DAD1 G, Sig=280,4 Ref=360,100

RT [min]	Type	Width [min]	Area	Height	Area%
7.306	BB	0.2051	36.582	2.6831	0.29
10.380	BB	0.3049	12578.919	624.8822	99.71

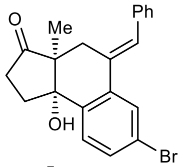

5n

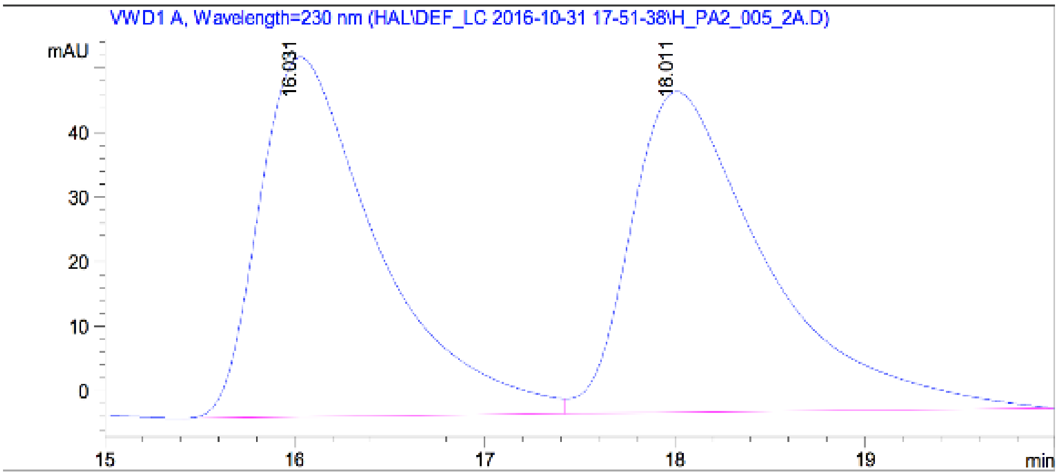

Meas. R	Area %	Width	Symmetr.
16.031	49.628	0.645	0.491
18.011	50.372	0.732	0.482

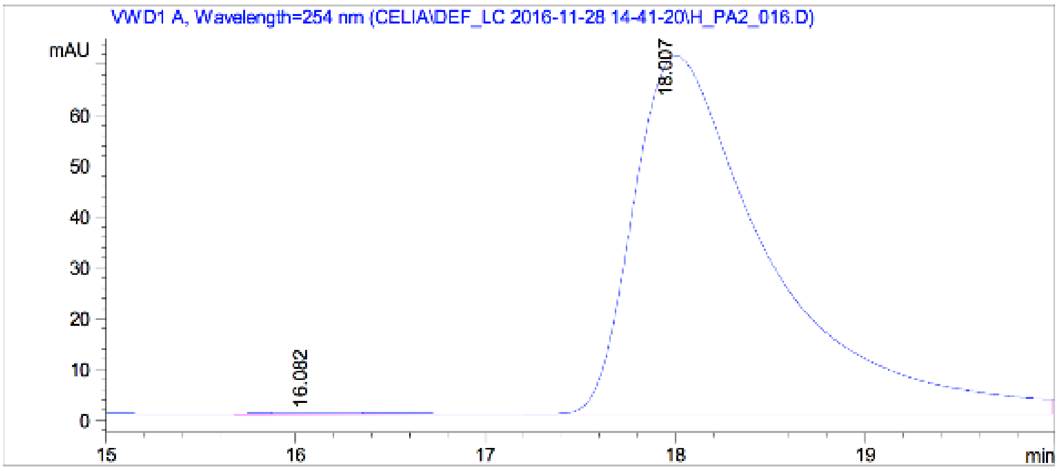

Meas. R	Area %	Width	Symmetr.
16.082	0.377	0.672	0.723
18.007	99.623	0.724	0.425

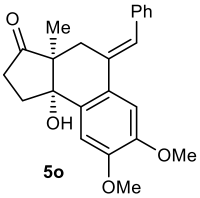

5o

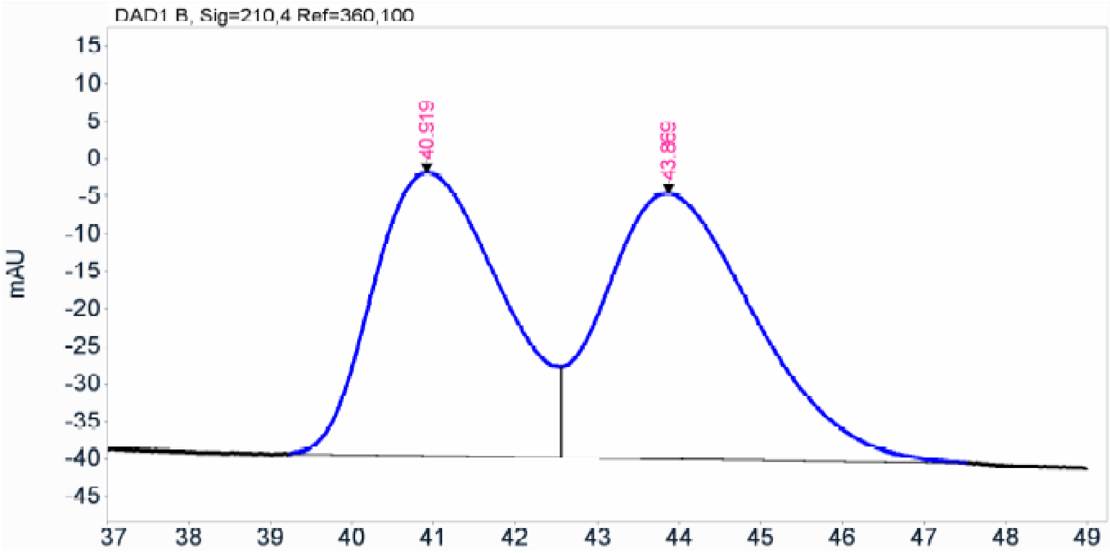

Signal:	DAD1 B, Sig=210,4 Ref=360,100				
RT [min]	Type	Width [min]	Area	Height	Area%
40.919	BV	1.2876	4124.541	37.7838	47.39
43.869	VB	1.5132	4578.141	35.4374	52.61

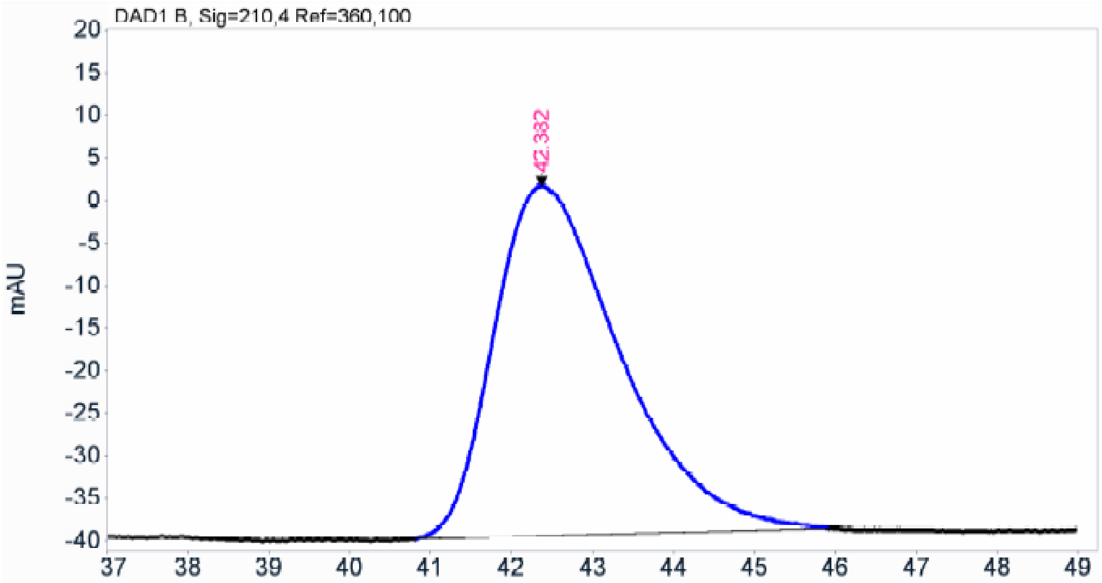

Signal:	DAD1 B, Sig=210,4 Ref=360,100				
RT [min]	Type	Width [min]	Area	Height	Area%
42.382	BB	1.2630	4419.429	41.0826	100.00

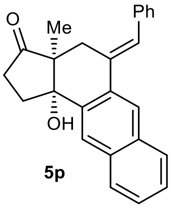

5p

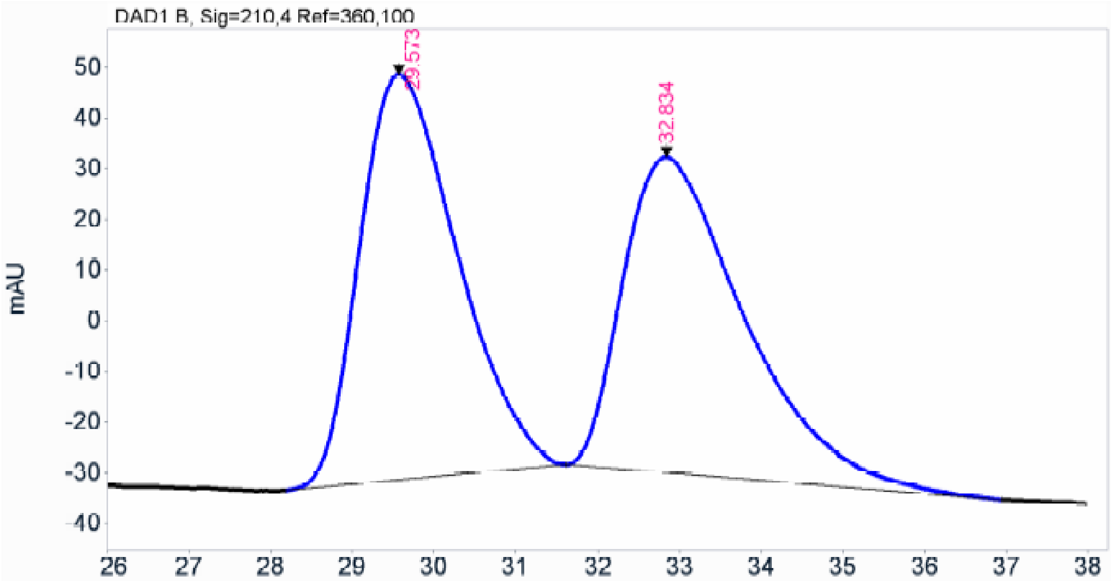

Signal:	DAD1 B, Sig=210,4 Ref=360,100				
RT [min]	Type	Width [min]	Area	Height	Area%
29.573	BB	1.2102	6634.788	79.9463	50.75
32.834	BB	1.2768	6439.603	62.2555	49.25

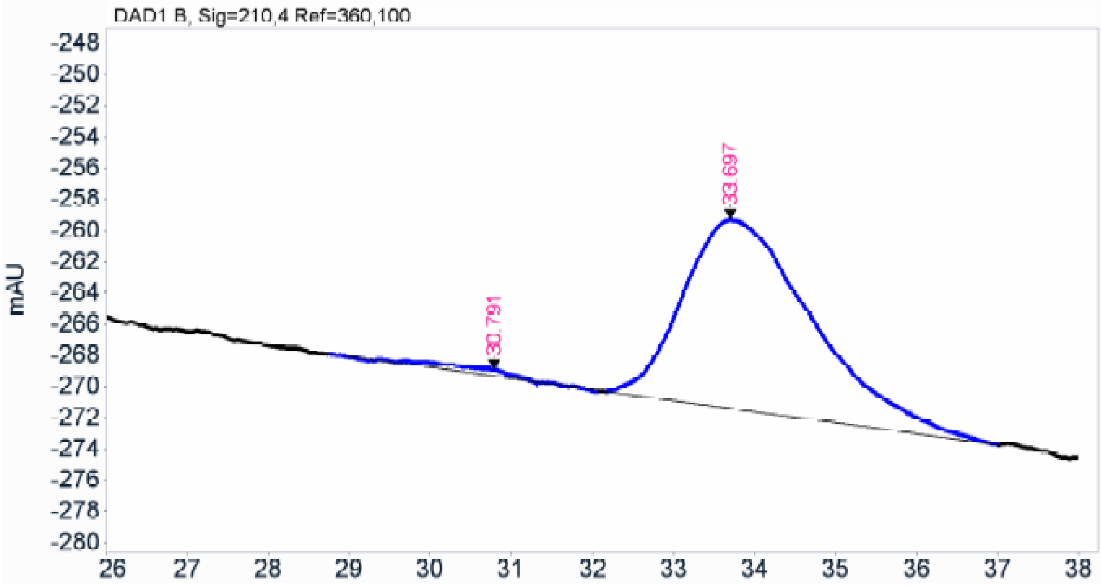

Signal:	DAD1 B, Sig=210,4 Ref=360,100				
RT [min]	Type	Width [min]	Area	Height	Area%
30.791	MM	1.1531	29.371	0.4245	2.12
33.697	BB	1.3145	1353.284	12.1217	97.88

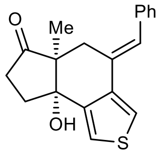

5ra

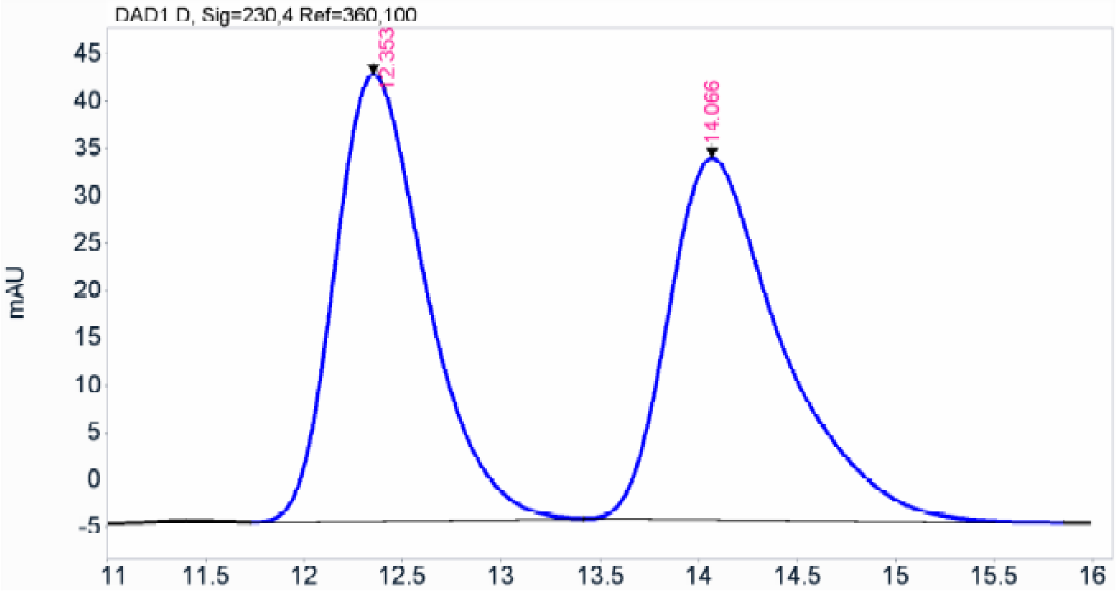

Signal:	DAD1 D, Sig=230,4 Ref=360,100				
RT [min]	Type	Width [min]	Area	Height	Area%
12.353	BB	0.4920	1520.132	47.1697	49.84
14.066	BB	0.5873	1530.195	38.2051	50.16

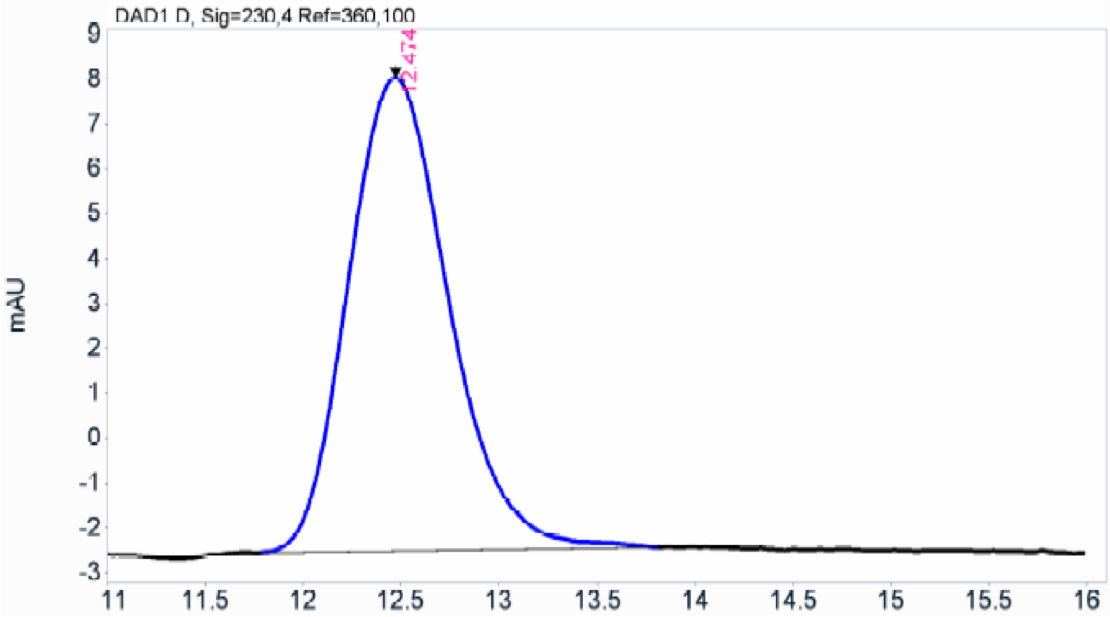

Signal:	DAD1 D, Sig=230,4 Ref=360,100				
RT [min]	Type	Width [min]	Area	Height	Area%
12.474	BB	0.5566	376.502	10.5426	100.00

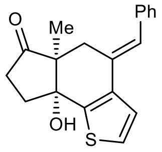

5rb

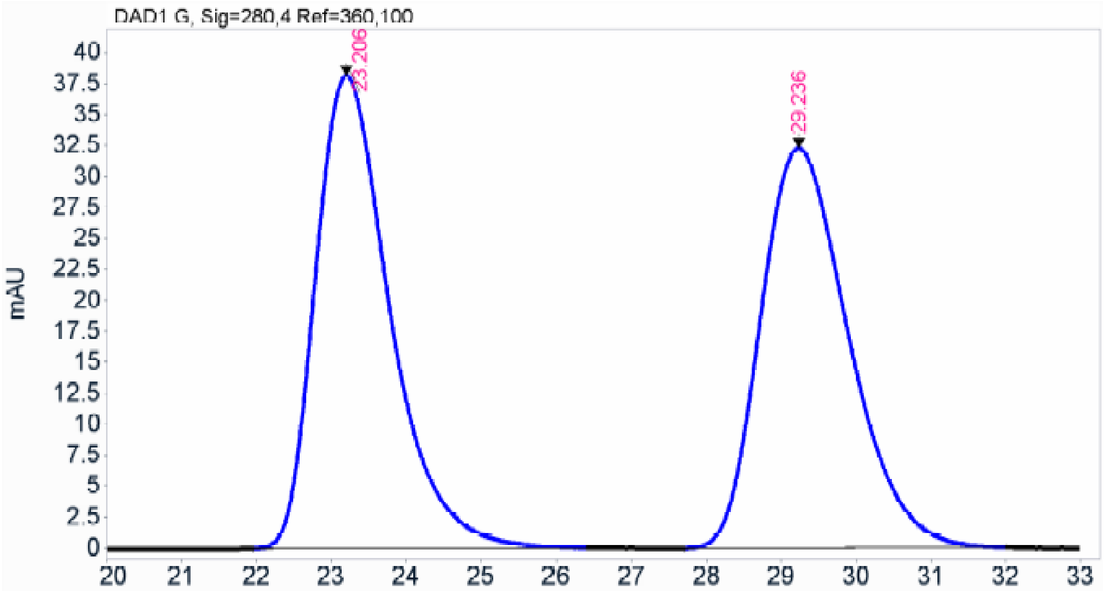

Signal: DAD1 G, Sig=280,4 Ref=360,100

RT [min]	Type	Width [min]	Area	Height	Area%
23.206	BB	1.0229	2632.945	38.1632	50.03
29.236	BB	1.2222	2629.708	32.2889	49.97

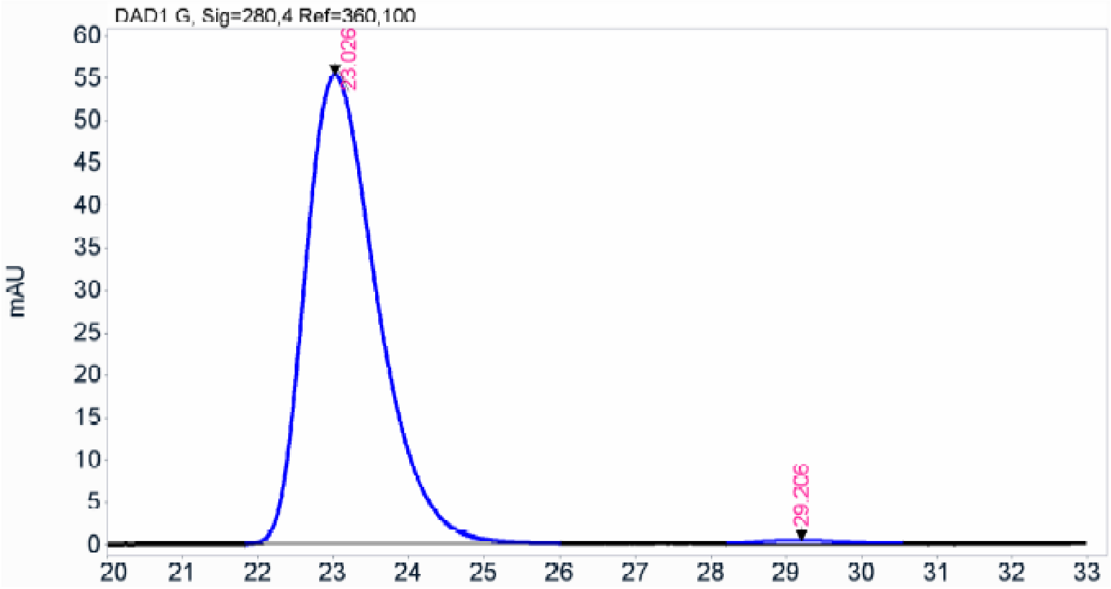

Signal: DAD1 G, Sig=280,4 Ref=360,100

RT [min]	Type	Width [min]	Area	Height	Area%
23.026	BB	1.0204	3679.825	55.2973	98.87
29.206	MM	1.4456	42.053	0.4848	1.13

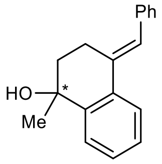

7

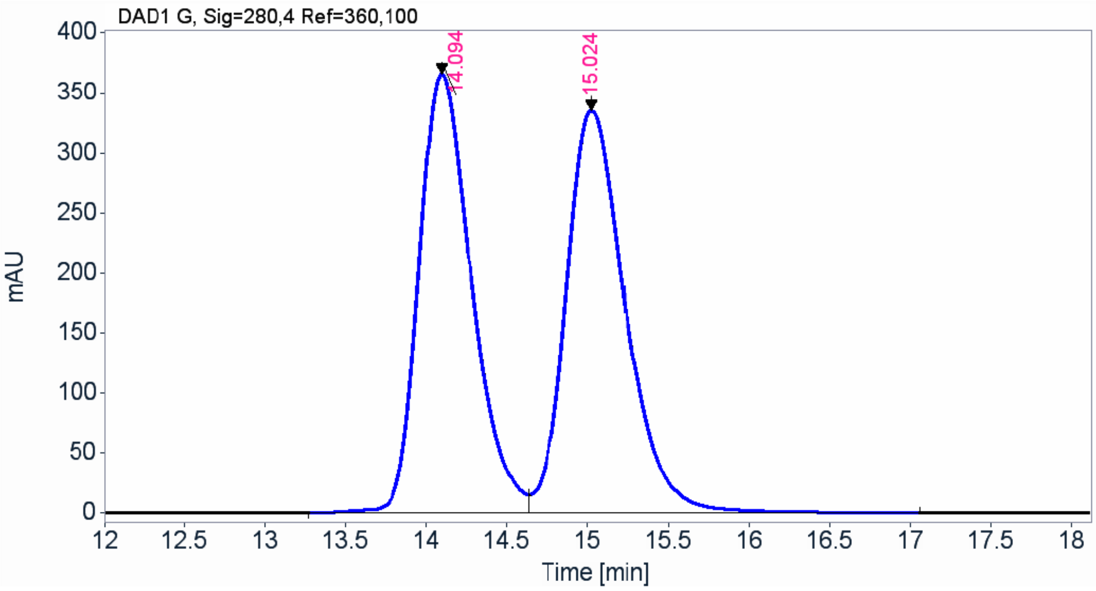

Signal:	DAD1 G, Sig=280,4 Ref=360,100				
RT [min]	Type	Width [min]	Area	Height	Area%
14.094	BV	0.3462	8285.378	365.8550	49.48
15.024	VB	0.3865	8459.277	335.0882	50.52

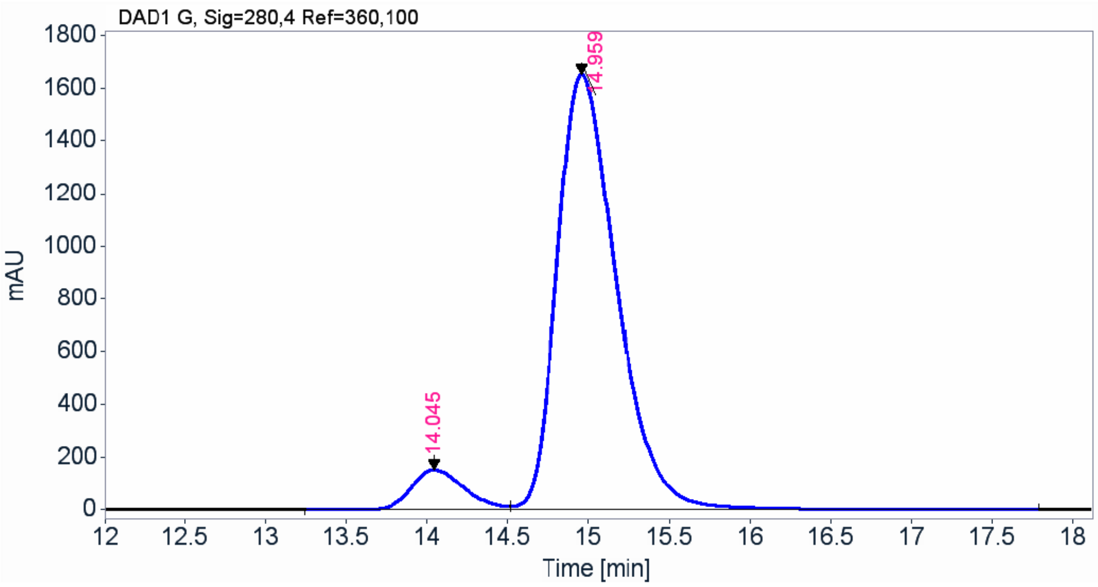

Signal:	DAD1 G, Sig=280,4 Ref=360,100				
RT [min]	Type	Width [min]	Area	Height	Area%
14.045	BV	0.3458	3446.738	152.4387	7.59
14.959	VB	0.3901	41937.324	1652.3798	92.41

References

1. B. M. Partridge, J. Solana González, H. W. Lam, *Angew. Chem., Int. Ed.* **2014**, *53*, 6523-6527.
2. S. Kallepu, K. K. Gollapelli, J. B. Nanubolu, R. Chegondi, *Chem. Commun.* **2015**, *51*, 16840-16843.
3. Prepared according to:A. I. Oliva, U. Christmann, D. Font, F. Cuevas, P. Ballester, H. Buschmann, A. Torrens, S. Yenes, M. A. Pericàs, *Org. Lett.* **2008**, *10*, 1617-1619.
4. M. Kenny, J. Christensen, S. J. Coles, V. Franckevičius, *Org. Lett.* **2015**, *17*, 3926-3929.
5. Prepared according to: I. Misztalewska, A. Z. Wilczewska, O. Wojtasik, K. H. Markiewicz, P. Kuchlewski, A. M. Majcher, *RSC Adv.* **2015**, *5*, 100281-100289.
6. Prepared according to: A. K. Ghosh, D. D. Anderson, *Org. Lett.* **2012**, *14*, 4730-4733.
7. Prepared according to: D. Kalaitzakis, J. D. Rozzell, S. Kambourakis, I. Smonou, *Eur. J. Org. Chem.* **2006**, 2309-2313.
8. Prepared according to: Z. He, H. Li, Z. Li, *J. Org. Chem.* **2010**, *75*, 4636-4639.
9. Prepared according to: C. Görl, H. G. Alt, *J. Organomet. Chem.* **2007**, *692*, 5727-5753. However, propargyl bromide (80% wt. in toluene) was used in place of propargyl chloride.
10. J.-i. Ito, K. Fujii, H. Nishiyama, *Chem. Eur. J.* **2013**, *19*, 601-605.
11. J. Yan, N. Yoshikai, *ACS Catal.* **2016**, *6*, 3738-3742.
